# General Synthetic Methodologies for Building Blocks to Construct Molecular Motors

**DOI:** 10.1021/acs.joc.4c02619

**Published:** 2025-02-28

**Authors:** Daniel Doellerer, John Y. de Boer, Ben L. Feringa

**Affiliations:** Stratingh Institute for Chemistry, Center for Systems Chemistry and Zernike Institute for Advanced Materials, Faculty of Mathematics and Natural Sciences, University of Groningen, Nijenborgh 3, 9747 AG Groningen, The Netherlands

## Abstract

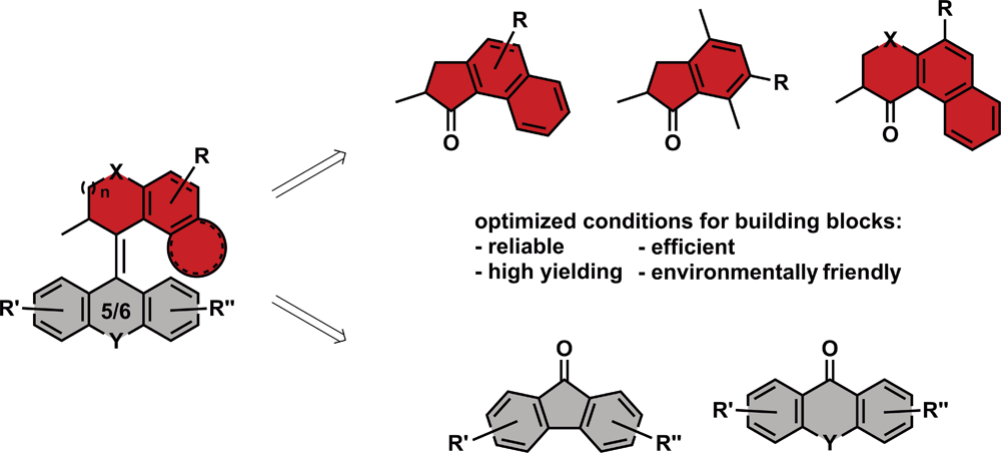

Here we present a collection of reliable, high yielding, and efficient synthetic methods for the preparation of commonly used building blocks to construct molecular rotary motors based on overcrowded alkenes. The easy access and robust synthesis procedures facilitate new endeavors to employ molecular motors in recently emerging fields toward dynamic chemical systems and responsive materials.

## Introduction

The field of light activated switchable molecules has its origin with the discovery of the photoisomerization process of azobenzene by G. S. Hartley in 1937,^1^ although the molecule azobenzene was initially reported more than 100 years earlier by E. Mitscherlich.^2^ Since then, the area rapidly advanced and chemists identified and designed an impressive family of molecular photoactuators.^3−5^ However, it was not until 1999, that our group discovered the first light-driven rotary molecular motor based on overcrowded alkenes that could also act as a multistage chiroptical switch.^6^ These molecular constructs feature four distinct states and have the special ability to perform unidirectional and repetitive rotation at the nanoscale level.^7^

**Figure 1:**
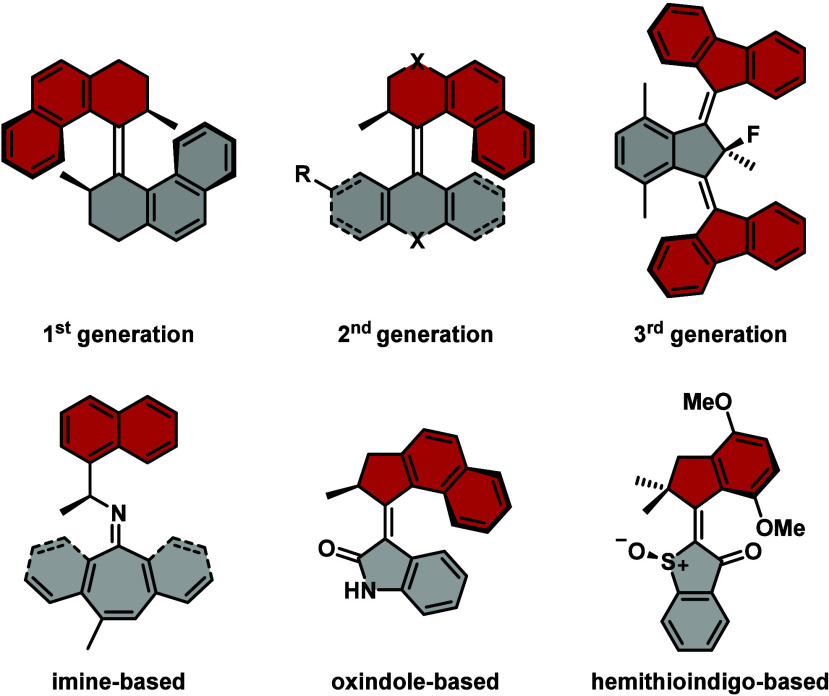
Illustration of various overcrowded alkene-based molecular motors, top: 1st generation (left), 2nd generation (middle; X = CH_2_, O, S; R = H, O-alkyl, NO_2_) and 3rd generation (right) motors; bottom: imine-based (left) oxindole-based (middle) and hemithioindigo-based (right) motors.

Since then, three generations of molecular motors have been developed (see Figure 1 for structures),^7^ which differ in terms of their number of stereocenters. The first generation features two identical halves with two stereocenters,^6^ while the second generation molecular motors usually consist of a symmetric (beside the functional groups) bottom part and most commonly feature a single stereocenter bearing top half.^8^ Recent discoveries by our group provide insight into rotation speed modulation and direction reversion by introduction of a β- instead of an α-methyl or a combination of them and improvement of motor efficiency.^9^ Additionally, the third generation exhibits a pseudoasymmetric center, originating from merging two second generation molecular motors together, rendering the molecule achiral.^10^ Besides the three generations, also other asymmetric types of imine-^11^ and overcrowded alkene-based molecular motors have been developed, such as oxindole-^12^ or hemithioindigo-based systems^13,14^ (see Figure 1).

Molecular motors based on overcrowded alkenes possess a myriad of applications, ranging from smart materials,^15,16^ to liquid crystals,^17^ catalysis,^14,18^ supramolecular systems^19,20^ and control of cell and membrane functions.^21,22^ Due to the rapid increasing interest using this class of photoactive molecules over the past few years, we here present a collection of reliable, high yielding, and efficient synthesis procedures to generate a diversity of substituted building blocks that form the basis for a range of overcrowded alkene-based molecular switches and motors. We envision that these synthetic methodologies will facilitate access to new interesting and highly functionalized molecules based on overcrowded alkene molecular switches and motors.

## Results and Discussion

Here we present an overview of how to efficiently synthesize the most commonly used molecular motor building blocks for overcrowded alkene-based structures. The synthetic pathways and procedures (see Supporting Information for detailed reaction conditions, general procedures and recommendations for molecular motor syntheses) are highly optimized concerning their yields or greener purification methods and tested regarding their reliability. Additionally, the scalability for most compounds was investigated, and the procedures can be executed on scales equal to or larger than 1 g.

### 5-Membered (Naphthalene-Based) Top Half Ketones

**Scheme 1:**
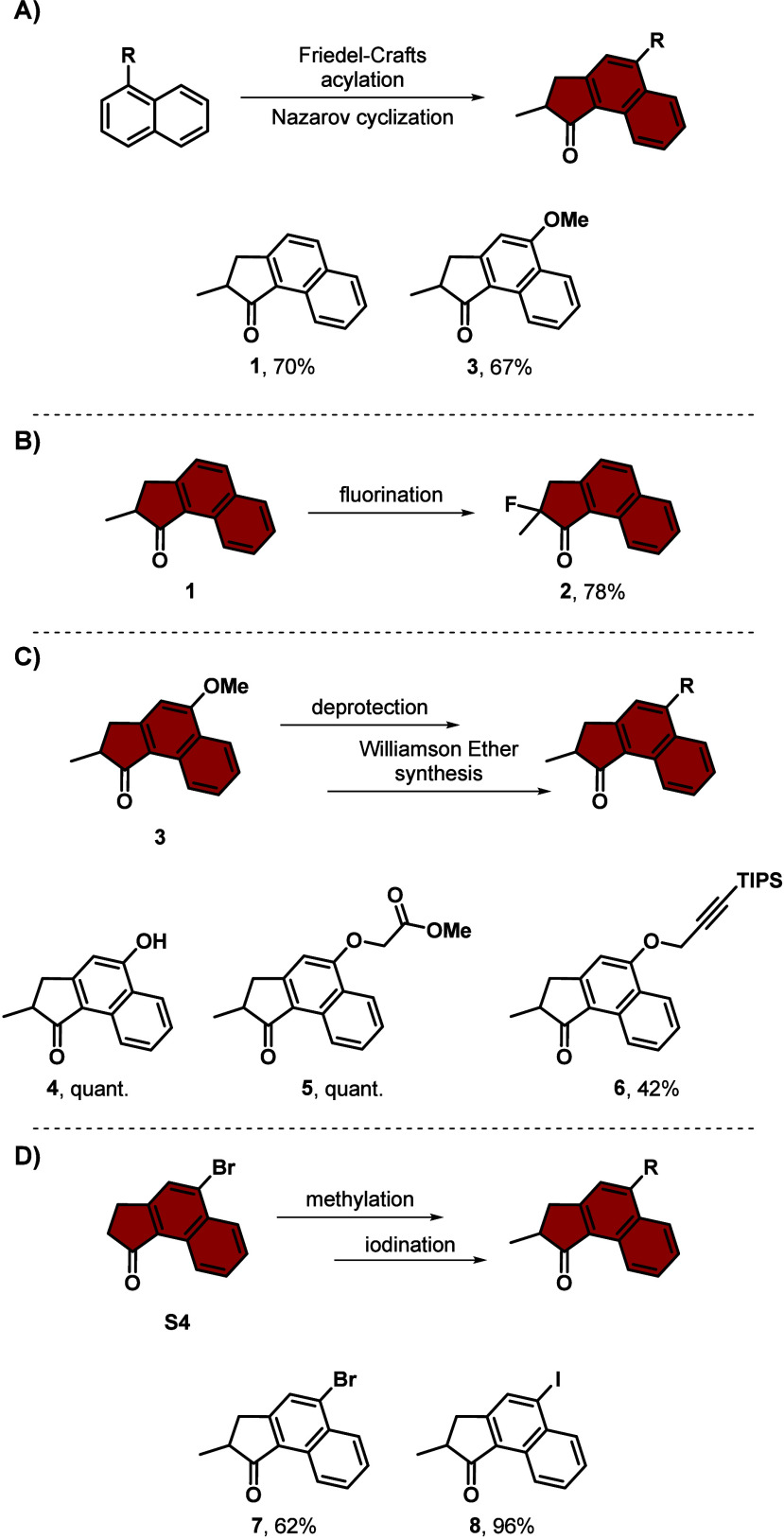
General Synthesis Schemes for 5-Membered Top Halves Based on Naphthalene. **A)** Synthesis of Unsubstituted (**1**) and Methoxy-Substituted (**3**) Naphthalene Top Halves *via* Friedel–Crafts Acylation Followed by a Nazarov Cyclization; **B)** Fluorination of **1** to Obtain the Fluorinated Top Half **2**; **C)** Deprotection of **3** Resulting in the Phenolic Top Half (**4**) Which Can Further React *via* a Williamson Ether Synthesis to **5** and **6**; and **D)** Methylation of **S4** to Form Bromo Top Half Ketone (**7**) Which Can Further Be Iodinated *via* a Copper Catalyzed Finkelstein Reaction to **8** Naphthalene, methacryloyl chloride, AlCl_3_, CH_2_Cl_2_, −78 °C to rt, 16 h, 70%. 1-Methoxynaphthalene, methacrylic acid, PPA 115%, 80 °C, 3 h, 67%. NFSI, LiHMDS, toluene, −78 °C to rt, 24 h, 78%. pyr·HCl, 180 to 210 °C, 3 h, quant. 2-Bromoacetate, K_2_CO_3_, MeCN, 75 °C, 16 h, quant. **S1**, K_2_CO_3_, DMF, 55 °C, 16 h, 42%. MeI, LiHMDS, THF, 0 °C to rt, 30 min, 62%. NaI, CuI, DMEDA, 1,4-dioxane, 140 °C, 72 h, 96%.

5-Membered top halves as the corresponding ketones are the most common ones used for the synthesis of molecular motors. They are mainly divided into naphthalene- and xylene-based variants or derivatives of these structures.^7^ The standard naphthalene-based top half **1** can be synthesized *via* a Friedel–Crafts acylation and consecutive Nazarov cyclization of naphthalene with methacryloyl chloride and AlCl_3_, acting as Lewis acid (see Scheme 1**A** and Supporting Information).^23^ There are different ways to purify **1**, of which the environmentally friendlier distillation results in a yellow oil while column chromatography results in a slight brown oil; however, NMR analysis shows no difference in purity. Also, worth mentioning is that the obtained oil solidifies with time or when layered with MeOH and stored in the fridge.

Top half **1** can readily undergo an electrophilic fluorination with *N*-fluorobenzenesulfonimide (NFSI) and LiHMDS as base, providing the fluorinated top half **2** (see Scheme 1**B** and Supporting Information).^24^ Fluorine-handles within molecular motor components are a powerful tool to analyze (^19^F NMR) and unravel the rotational behavior of molecular motors.^24^

Introduction of a methoxy group offers the benefits of higher electron density throughout the motor system due to its push-character but also presents a handle for further functionalization.^25,26^ Methoxy-substituted top half **3** was synthesized similarly to **1***via* a Friedel–Crafts acylation followed by a Nazarov cyclization (see Scheme 1**A** and Supporting Information).^25^ Compound **3** can be deprotected with pyridine hydrochloride, yielding the phenolic compound **4**,^26^ which can then be further converted *via* a Williamson ether synthesis with various alkyl halides. Reaction with 2-bromoacetate results in the ester modified top half **5**, which can be saponified to the free acid after motor formation, which can subsequently be coupled with alcohols or amines to form the respective esters or amides. To install a very useful click-handle, first propargyl bromide needs to be protected with triisopropylsilyl chloride (TIPSCl). **S1** can then react with **4**, resulting in top half **6** (see Scheme 1**C** and Supporting Information).^26^ Any remainders of TIPSCl or its hydrolyzed derivative can be removed *in vacuo* at 70 °C. Top half **6** can be deprotected after motor formation with TBAF, releasing the click-handle.^26^ It is noteworthy that deprotection of esters or unprotected triple bonds diminishes or completely hinders the proceeding of the Barton–Kellogg coupling of upper and lower halves (*vide infra*), being the key step in motor synthesis, due to the acidity of the protons involved.

Installing halogens on either the top or bottom halves opens up the possibility to further functionalize the structures after the molecular motor is formed. Halogen substituents can be further reacted *via* cross coupling reactions but also transformed into other functional groups like cyanides.^25,27^ The synthesis of the 5-bromo naphthalene-based top half **7** is quite challenging. The so far reported one pot synthesis between 1-bromonaphthalene and either methacrylic acid or methacryloyl chloride using polyphosphoric acid (PPA) or AlCl_3_, respectively, suffers from yields around 5% and side reactions such as dehalogenation.^28,29^ Here we present a facile synthesis involving 4 steps resulting in a roughly 50% overall yield of pure material. First, 1,4-dibromonaphthalene was lithiated with *n*-BuLi and further reacted with TMSCl to form **S2**, which was then converted *via* Friedel–Crafts acylation with 3-chloropopanoyl chloride mediated by AlCl_3_, forming precursor **S3**. Subsequently, **S3** underwent an intramolecular Friedel–Crafts alkylation, cyclizing the compound to **S4**, which was executed in the presence of AlCl_3_ and H_2_SO_4_.^30^ The final step to obtain molecule **7** was the methylation of **S4**. Therefore, we applied a published procedure by our group to methylate 6-membered ketone-based upper halves.^31^**S4** can react with MeI and freshly prepared lithium diisopropylamide (LDA) acting as a base. However, this methylation suffers from low yields due to the generation of various compounds. Next to residual **S4** (∼30%), the desired compound **7** (∼20%) and the double methylated compound **7b** (∼25–30%) were isolated (see Scheme 1**D** and Supporting Information). The addition of hexamethylphosphoramide (HMPA) as described in the literature did not push the reaction to the desired product in this case.^31,32^ Drawing inspiration from a literature report of the mid 90s on α-methylations of ketones *via* manganese enolates mitigated the polyalkylation problems.^33^ Formation of the manganese enolate, however, did not result in the desired selectivity; therefore, ketone **S4** was reacted with LiHMDS, forming the corresponding lithium enolate. Subsequently, the formed lithium enolate was reacted under slow addition to a solution of MeI, forming **7** as the main component and resulting in a yield of 62% compared with the prior applied LDA procedure.

Having different types of reactive groups within a molecule is crucial to be able to introduce a high degree of complexity by functionalization of different parts when needed. Substituting the bromide of top half **7** with an iodide *via* a copper catalyzed Finkelstein reaction introduces a more reactive moiety in particular for Sonogashira cross couplings, when in combination with, for example, bottom half **20** (*vide infra*). The iodination reaction takes place when **7** reacts with NaI, CuI and *N*,*N*′-dimethylethylenediamine (DMEDA) at elevated temperatures, yielding iodinated molecule **8** (see Scheme 1**D** and Supporting Information).

### 5-Membered (Xylene-Based) Top Half Ketones

**Scheme 2:**
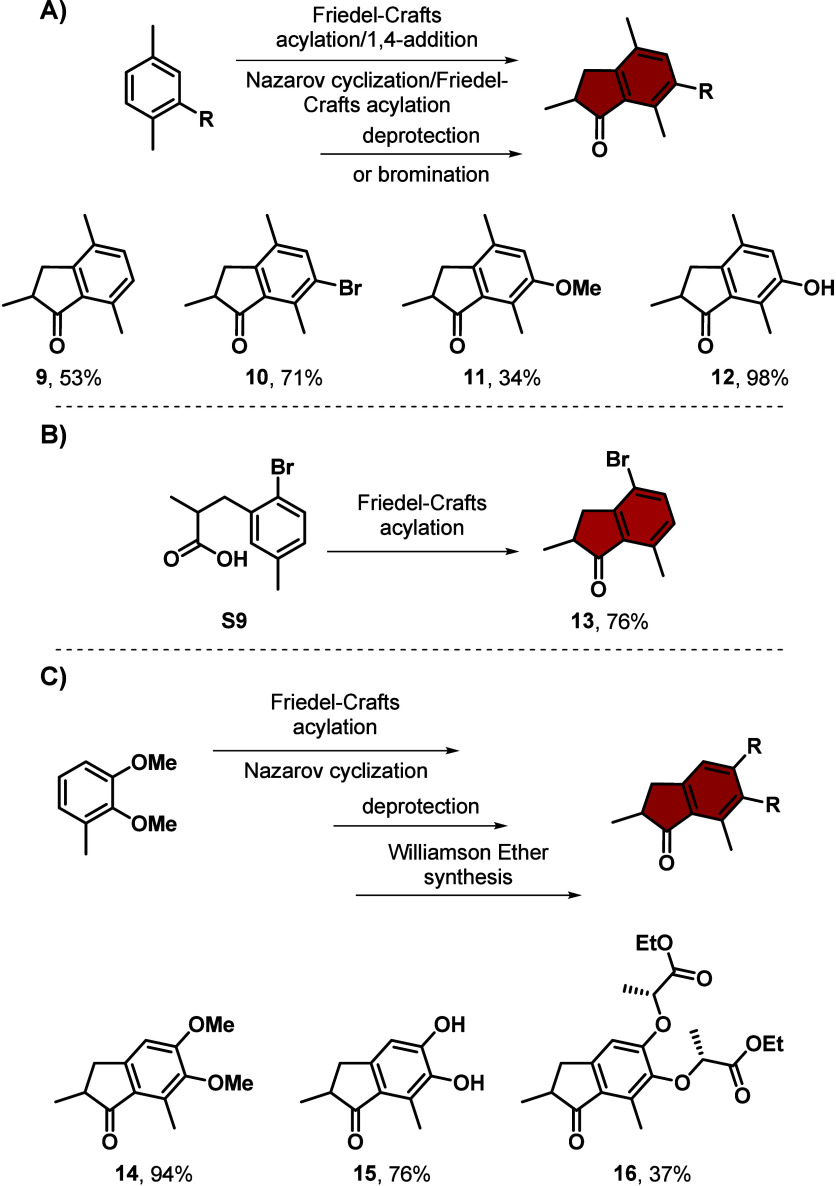
General Synthesis Schemes for 5-Membered Top Halves Based on Xylene. Synthesis of **A)** Unsubstituted (**9**) and Methoxy-Substituted (**11**) Top Half Based on Xylene *via* Friedel–Crafts Acylation Followed by a Nazarov Cyclization or 1,4-Addition Followed by a Friedel–Crafts Acylation, Respectively, Which Can Further Undergo Bromination (**10**) or Deprotection (**12**); **B)** 4-Bromo Indanone Based Top Half (**13**) *via* Friedel–Crafts Acylation; and **C)** Dimethoxy Indanone Top Half *via* Friedel–Crafts Acylation Followed by a Nazarov Cyclization (**14**) Which Can Further Be Deprotected (**15**) and React *via* a Williamson Ether Synthesis to **16** *p*-Xylene, methacryloyl chloride, AlCl_3_, CH_2_Cl_2_, −78 °C to rt, 16 h, 53%. 2-Methoxy-1,4-dimethylbenzene, methacrylic acid, Eaton’s reagent, 80 °C, 2 h, 34%. **9**, NBS, H_2_SO_4aq_, MeCN, 70 °C, 16 h and NaI, TMSCl, MeCN, rt, 16 h, 71%. **11**, AlCl_3_, toluene, 100 °C, 4 h, 98%. **S9**, H_2_SO_4_, rt, 16 h, 76%. 1,2-Dimethoxy-3-methylbezene, methacrylic acid, PPA 115%, 70 °C, 3 h, 94%. BBr_3_, CH_2_Cl_2_, 0 °C to rt, 16 h, 76%. (*S*)-2-Hydroxypropanoate, PPh_3_, DIAD, THF, 0 °C to rt, 16 h, 37%.

Besides the 5-membered top half ketones based on naphthalene, the second major class is based on xylene, very often employed within first generation molecular motors.^7^ The unsubstituted top half **9** was obtained by direct Friedel–Crafts acylation of *p*-xylene with methacryloyl chloride facilitated by AlCl_3_ followed by a Nazarov cyclization (see Scheme 2**A** and Supporting Information). The crude product was distilled instead of purified *via* column chromatography to make the workup more scalable and greener.

Top half **9** can be brominated with the aid of NBS and H_2_SO_4_ in an electrophilic substitution reaction, where the α-position of the carbonyl gets brominated involving acid catalysis. The bromide introduced in the α-position was reduced with NaI and TMSCl to provide top half **10**.^34^ Among others, compound **10** can undergo enantioselective protonation, by utilizing a chiral gold complex, (*R*)- or (*S*)-BINAP(AuCl)_2_ (2,2′-bis(diphenylphosphino)-1,1′-binaphthyl), and AgBF_4_. This sequence proceeds *via* the silyl enol ether formation of the respective compounds,^35^ resulting in enantiomerically pure **(*****R*****)-** or **(*****S*****)-10**,^36^ respectively (see Scheme 2**A** and Supporting Information). It is worth mentioning that the enantiomerically excess is even further increased using a McMurry coupling in the motor formation, where exclusively homo coupling of the same enantiomer occurs, to the respective (*R*,*R*)- or (*S*,*S*)-molecular motors.^36^

On the other hand, reacting 2-methoxy-1,4-dimethoxybenzene with methacrylic acid in Eaton’s reagent protonates the acid and activates it toward the 1,4-addition, which is followed by an intramolecular Friedel–Crafts acylation reaction, yielding compound **11**.^37^ It can be readily deprotected to the free phenol and compound **12**(36) similar to the related phenol compounds, which can further react with various alkyl halides (see Scheme 2**A** and Supporting Information).

Top half **13**, where one of the methyl groups is substituted by a bromide, provides the possibility for further functionalization after motor formation, as the substituent is not in direct conjugation with the double bond. Starting off with a reduction of methyl 2-(2-bromo-5-methylphenyl)acetate with LiAlH_4_ yielded **S5**,^38^ which was recrystallized from *n*-heptane instead of purification by column chromatography. **S5** can then be tosylated with TsCl and KOH as base, resulting in molecule **S6**. Compound **S6** can be further converted *via* a nucleophilic substitution with diethyl 2-methylmalonate and NaH as base, yielding diester **S7**. Molecule **S7** can be saponified with NaOH to acid **S8**,^39^ which at elevated temperatures decarboxylates to compound **S9**.^39^ Under the influence of concentrated sulfuric acid, **S9** undergoes an intramolecular Friedel–Crafts reaction resulting in bromo-substituted top half **13** (see Scheme 2**B** and Supporting Information).^39^

Enantiomerically pure top halves are critical to ensure directional rotation, which can, for instance, be applied for winding and unwinding of supramolecular aggregates, polymers or liquid crystal materials and controlled actuation of the systems they are embedded in.^40^ So far, the established methods are scarce, such as the enantioselective protonation,^36^ utilization of enzymatic resolution^41^ or procedures using enantiomerically pure starting materials.^9^ Introduction of enantiomerically pure lactate substituents as functional groups, as first shown by Giuseppone et al., which can be further coupled, can be applied to separate the (*R*)- and (*S*)-diastereomers of the episulfide during motor formation with 6-membered bottom halves, providing either the (*R*,*R*,*R*) or (*S*,*R*,*R*) molecular motor.^40^ To obtain the precursor for the lactate substituted top half, first, compound **14** was synthesized *via* Friedel–Crafts acylation and Nazarov cyclization of 1,2-dimethoxy-3-methylbenzene and methacrylic acid in PPA.^42^ The reaction is very robust and can be performed on scales exceeding 20 g. Double dimethoxy-substituted compound **14** can readily be deprotected with BBr_3_, resulting in dihydroxy-substituted compound **15**.^42^ Compound **15** can undergo a double Mitsunobu reaction with the enantiomerically pure ethyl (*S*)-2-hydroxypropanoate, facilitated by diisopropyl azodicarboxylate (DIAD) and PPh_3_, resulting in the bis-lactate substituted top half **16** (see Scheme 2**C** and Supporting Information).^43^

### 6-Membered Top Half Ketones

**Scheme 3:**
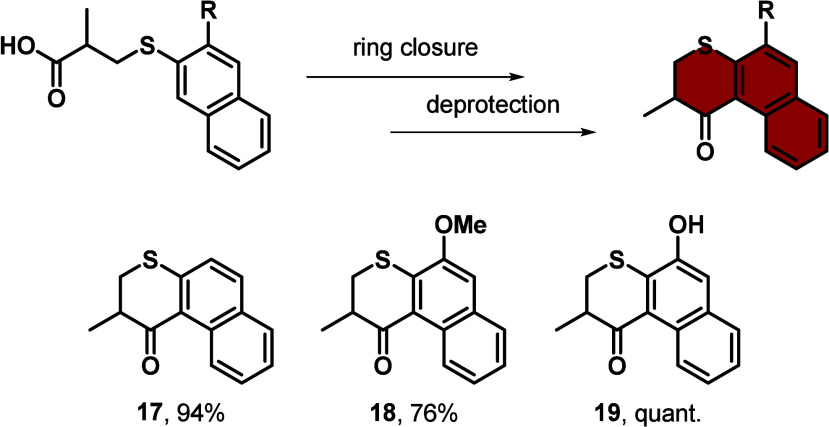
General Synthesis Schemes for 6-Membered Top Halves Based on Naphthalene. Synthesis of Unsubstituted (**17**) and Methoxy-Substituted (**18**) Top Half Based on Naphthalene *via* an Intramolecular Cyclization Which Can Further Be Deprotected (**19**) **S10**, Eaton’s reagent, 70 °C, 2 h, 94%. **S11**, Eaton’s reagent, 80 °C, 2 h, 76%. pyr·HCl, 180 to 210 °C, 3 h, quant.

6-Membered ketone-based top halves show more steric hindrance and a higher thermal helix inversion (THI) barrier compared to 5-membered ones, and as a consequence result in reduced rotation speed.^7^ One of the most commonly used top halves is based on a sulfur containing heterocyclic ring, i.e. a benzothiochromanone.^7^ To form molecule **17**, naphthalene-2-thiol was first converted using a thia-Michael addition reaction with methacrylic acid facilitated by Et_3_N, generating **S10**,^24^ which can readily undergo an intramolecular cyclization induced by Eaton’s reagent forming ketone **17** (see Scheme 3 and Supporting Information). In the case of **18**, first, a lithiation with *n*-BuLi on 2-methoxynaphthalene was performed which was followed by an electrophilic aromatic substitution, also known as thiolation, with elemental sulfur, yielding 3-methoxynaphthalene-2-thiol, whose commercial availability is limited. 3-Methoxynaphthalene-2-thiol was then transformed into **S11** using the same reaction sequence as for **17**.^44^ The cyclic ketone was obtained after intramolecular cyclization with Eaton’s reagent. The use of Eaton’s reagent, instead of the formation of the acid chloride and subsequent Friedel–Crafts acylation for intramolecular ring closure of the molecules, results in an easier workup and increased yields. Compound **18** was then deprotected with pyridine hydrochloride, forming the free phenolic upper half **19** (see Scheme 3 and Supporting Information),^44^ which can undergo various coupling reactions with alkyl halides.

### 5-Membered Bottom Half Ketones

Various 5-membered bottom halves based on fluorenones with substitutions at the 2,7-positions are commercially available. On the other hand, desymmetrized molecules, bearing one functional group, or fluorenones with substitutions at the 3,6-positions are either less commonly available, hard to purify or more expensive. Therefore, reliable procedures are required for these compounds especially. 3,6-Substituted 5-membered fluorenone-based bottom halves are very versatile when it comes to further red-shift the absorbance of molecular motors, due to their direct conjugation with the top half.^25^ Additionally, halide-substituted fluorenones open up further reaction pathways, especially cross coupling reactions. It is important to note that substitutions such as Suzuki or Sonogashira reactions before formation of the molecular motor should be avoided due to the extension of the pi-system of the core. In our experience, extending the pi-system usually decreases the reactivity within the Barton–Kellogg coupling, leading to significantly reduced yields.

**Scheme 4:**
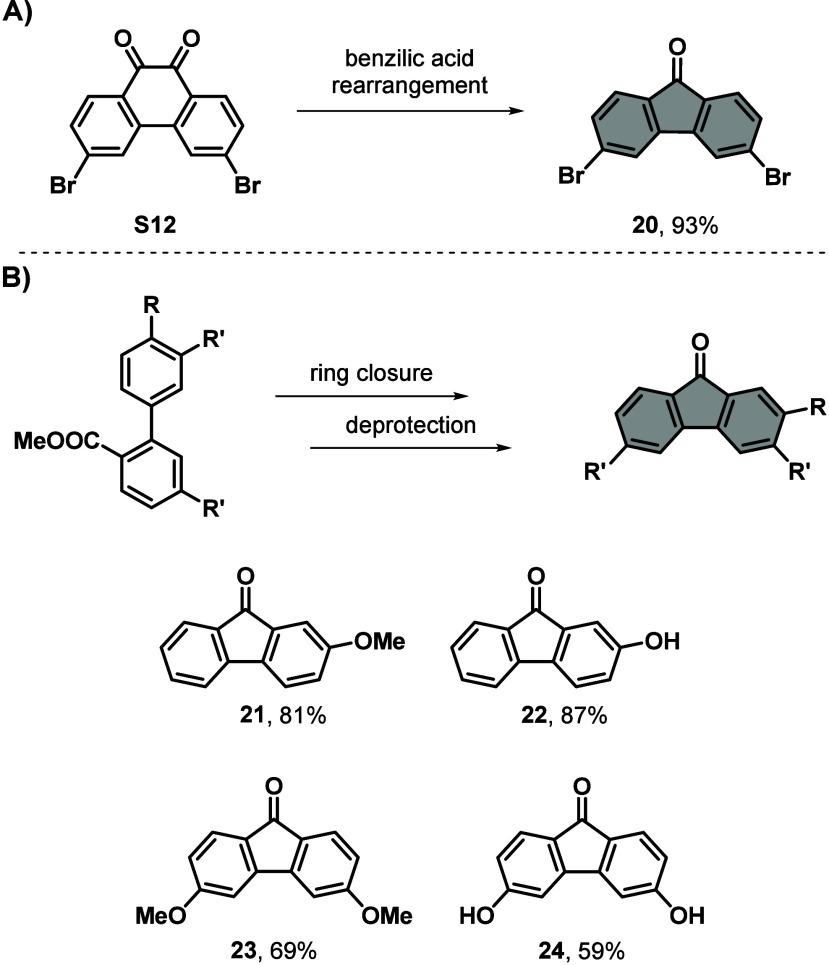
General Synthesis Schemes for 5-Membered Bottom Halves Based on Fluorenone. Synthesis of **A)** 3,6-Dibromofluorenone (**20**) *via* Benzilic Acid Rearrangement and **B)** Ring Closure to 2-Methoxyfluorenone (**21**) and 3,6-Dimethoxyfluorenone (**23**) *via* an Acid Catalyzed Friedel–Crafts Cyclization Which Can Be Further Deprotected to Form (**22**) and (**24**) **S12**, KOH, KMNO_4_, H_2_O, 120 °C, 3 h, 93%. **S13**, PPA 115%, 80 °C, 2 h, 81%. **S15**, PPA 115%, 80 °C, 2 h, 69%. AlCl_3_, toluene, 100 °C, 4 h, 87%. AlCl_3_, toluene, 100 °C, 16 h, 59%.

Molecular motors based on the 3,6-dibromo-substituted bottom half **20** and further derivates thereof have found applications for surfaces anchoring^27^ or drug delivery.^16^ Nevertheless, molecule **20** comes with an inherently tedious synthesis, attributed to the chemicals used and reduced solubility in organic solvents. Starting from the radical bromination of phenanthrene-9,10-dione with bromine and benzoyl peroxide as the initiator, **S12** was obtained. During the next step, a benzylic acid type rearrangement (oxidative decarboxylation and cyclization in this case)^45^ facilitated by KMnO_4_ was performed, yielding **20** (see Scheme 4**A** and Supporting Information).^46^ It is worth mentioning that, due to the vast generation of CO_2_, the vessel for the reaction should be significantly larger than the amount of water, which is the solvent, used. Furthermore, to work up the reaction mixture, H_2_SO_4_ and Na_2_SO_3_ were used to quench remainders of KMnO_4_, acidifying the reaction mixture and transforming any excess KMnO_4_ and MnO_2_ into soluble manganese salts, respectively. Other reducing agents like, for example, S_2_O_3_^2–^ did not work as well.

The asymmetric bottom half **21** was synthesized starting *via* a Suzuki cross coupling of (4-methoxyphenyl)boronic acid and 2-iodobenzoate in a water/MeCN mixture, where Na_2_CO_3_ acted as a base and Pd(OAc)_2_ as catalyst, yielding **S13**.^47^ The reaction proceeds quantitatively, and the product is obtained pure after removal of the combined organic layers. **S13** was then further reacted in PPA *via* a Friedel–Crafts cyclization, resulting in compound **21**.^48^ Deprotection of **21** to **22** takes place with AlCl_3_, generating a phenol handle often used for motors applied in liquid crystals (see Scheme 4**B** and Supporting Information).^17^

Bottom half **23** was previously synthesized by our group in 4 steps in an overall yield of 40%.^25^ Here we present a greener synthesis comprising 3 steps and an improved overall yield of 56%. First, 2-iodo-4-methoxybenzoid acid was transformed into its ester analog with MeOH, catalyzed by H_2_SO_4_ (**S14**).^49^ Compound **S14** was then cross coupled using a Suzuki reaction, similar to the formation of **21**, with (3-methoxyphenyl)boronic acid, yielding **S15**. Disubstituted fluorenone **23** was then formed in the same way as **21** using PPA. The reduced yield for this ring closure reaction originates from the possibility to undergo cyclization at the other *ortho*-position, generating a different isomer. Additionally, the number of steps can even be reduced to 2 steps when proceeding immediately to the Suzuki reaction from the acid precursor or starting from the commercial ester (the overall yield excluding the ester formation improves to 66% over two steps). Deprotection of **23** with AlCl_3_ in toluene results in the diol **24** (see Scheme 4**B** and Supporting Information).

### 6-Membered Bottom Half Ketones

**Scheme 5:**
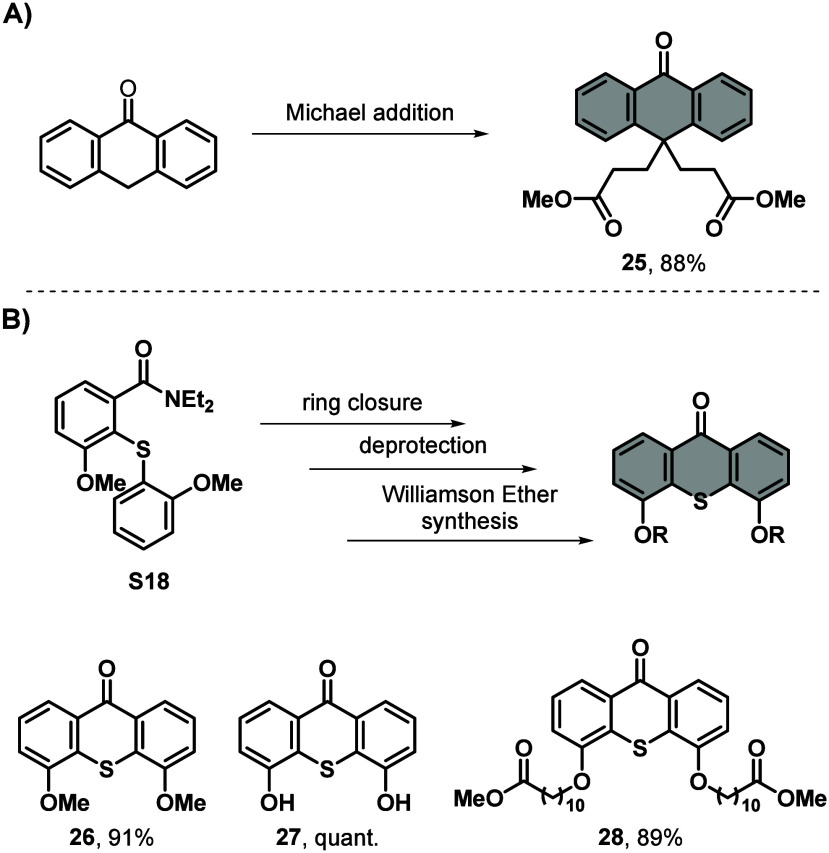
General Synthesis Schemes for 6-Membered Bottom Halves Based on Anthrone and Thioxanthone. Synthesis of **A)** 9,9-Substituted 6-Membered Bottom Half Based on Anthrone (**25**) *via* Michael Addition and **B)** Ring Closure to 4,5-Dimethoxy Substituted 6-Membered Bottom Half Based on Thioxanthone (**26**) Which Can Further Be Deprotected (**27**) and Reacted *via* a Williamson Ether Synthesis (**28**) Anthrone, methyl acrylate, NaOMe, THF/MeOH, rt, 4 h, 88%. **S18**, DIPA, *n*-BuLi, THF, 0 °C to rt, 2 h, 91%. BBr_3_, CH_2_Cl_2_, 0 °C to rt, 16 h, quant. 11-Bromoundeanoate, Cs_2_CO_3_, DMF, 100 °C, 16 h, 89%.

6-Membered bottom halves, often based on anthrone or thioxanthone, are highly represented within ultrafast molecular motors when coupled with a 5-membered top half, due to a reduced THI barrier.^7^ Molecule **25**, an anthrone-based 6-membered bottom half, is a valuable building block representing a facile anchoring unit for surface modifications with molecular motors.^50,51^ Compound **25** was synthesized *via* a Michael addition by reacting anthrone with methyl acrylate in a mixture of tetrahydrofuran (THF)/MeOH facilitated by NaOMe at room temperature (see Scheme 5**A** and Supporting Information).^50^ Possible modifications after motor formation include deprotection and subsequent substitution reactions or reduction.^51^

The second group of 6-membered bottom halves is based on a thioxanthone core and used for fast motors in responsive amphiphiles^52^ as well as surface anchoring of rotary motors.^53^ First, an oxidation of 2-methoxybenzenethiol with KMnO_4_ and CuSO_4_ in dichloromethane (DCM) at room temperature to the disulfide **S16** was performed, generating the first precursor needed for the thioxanthone-based bottom half **26**. The other precursor was synthesized *via* formation of the acid chloride of 3-methoxybenzoic acid with thionyl chloride in dichloromethane at elevated temperatures followed by a Schotten–Baumann reaction with diethylamine, yielding **S17**. Subsequent *ortho*-lithiation of **S17** by *t*-BuLi at −78 °C, in the presence of *N*,*N*,*N*′,*N*′-tetramethylethylenediamine (TMEDA) and addition of **S16** yielded **S18***via* a disulfide bond cleavage. Here, a more environmentally friendly recrystallization method from EtOAc was applied compared to purification *via* flash column chromatography. Thioxanthone **26** was synthesized *via* an intramolecular LDA (freshly prepared) promoted cyclization of **S18**. Also in this case, greener recrystallization from methanol was used compared to column chromatography. Compound **26** can readily be deprotected with BBr_3_ in DCM, resulting in thioxanthone **27**.^44^ Bottom half **27** can undergo a Williamson ether synthesis with various alkyl halides and bases, for example in the case of methyl 11-bromoundeacanoate and Cs_2_CO_3_, resulting in disubstituted thioxanthone bottom half **28**, which was purified *via* recrystallization from methanol (see Scheme 5**B** and Supporting Information).^52^ Possible further reactions are similar for **28** as for **25**.

## Conclusions

In summary, we present an overview of reliable, high yielding, and efficient synthetic procedures for the generation of the most commonly used molecular motor building blocks. The synthesis steps have been revisited, the number of steps for several of them reduced, and their workups optimized, making the processes more environmentally friendly. Easy access to quantities of pure motor building blocks facilitates construction of further functionalized molecular motors while paving the way toward new applications.

## Data Availability

The data underlying this study are available in the published article and its online Supporting Information.

## Supporting Information Available

Experimental procedures, ^1^H, ^13^C{^1^H} and ^19^F NMR spectroscopic data (PDF)
