# Supplementary material for: General Synthetic Methodologies for Building Blocks to Construct Molecular Motors
Source: J Org Chem. 2025 Feb 28;90(10):3519–26. doi: 10.1021/acs.joc.4c02619 (PMC11915373; doi:10.1021/acs.joc.4c02619)
Supplement: Supplementary file 1 — jo4c02619_si_001.pdf [file jo4c02619_si_001.pdf]

Supporting Information

General Synthetic Methodologies for Building Blocks to Construct Molecular Motors

Daniel Doellerer‡, John Y. de Boer‡ & Ben L. Feringa*

Stratingh Institute for Chemistry, Center for Systems Chemistry and Zernike Institute for Advanced Materials, Faculty of Mathematics and Natural Sciences, University of Groningen, Nijenborgh 3, 9747 AG Groningen, The Netherlands

‡ contributed equally
* corresponding author: b.l.feringa@rug.nl

Table of Contents

GENERAL INFORMATION **S3**
SYNTHESIS **S7**
REFERENCES **S36**
APPENDIX **S38**
NMR SPECTRA S38

General Information

Chemicals were purchased from commercial sources, Sigma-Aldrich (PPA 115%), Fluorochem, TCI (Eaton's reagent), BLDpharm and used without further purification. Dried solvents were obtained from Acros Organics, Alfa Aesar or from a solvent purification system (MBraun SPS-800). Unless stated otherwise, all reactions were carried out in oven-dried glassware under a nitrogen atmosphere using standard Schlenk techniques. Solids were added in a counter flow of nitrogen or before crimping reaction vials and cycled three times between vacuum and nitrogen before addition of liquids. Solutions and reagents were added with nitrogen-flushed disposable syringes/needles. Glassware in heated reactions was placed in customized aluminum heating blocks (crimp top vials as well as round bottom flasks) and oil baths (Schlenk tubes).

Analytical thin layer chromatography (TLC) was performed on silica gel 60 G/UV265 aluminum sheets from Merck (0.25 mm). Flash column chromatography was performed on silica gel Davisil LC60A (Merck type 9385, 230–400 mesh) or a Biotage Selekt system (MPLC) using the indicated solvents. NMR spectra were recorded on a Varian Mercury-Plus 400, a Varian Unity Plus 500 or a Bruker 600 MHz NMR spectrometer at 298 K unless stated otherwise. Chemical shifts are reported in parts per million (ppm) and referenced to the residual solvent signal ($CDCl_3$: δ = 7.26 for ^{1}H and 77.2 for ^{13}C{^{1}H}; $(CD_3)_2SO$: δ = 2.50 for ^{1}H and 39.5 for ^{13}C{^{1}H}) and thereby relatively stated to TMS. The resonance multiplicity is indicated as s = singlet, d = doublet, t = triplet, q = quartet, p = pentet, h = hextet, m = multiplet, dd (doublet of doublets), td (triplet of doublets), ddd (doublet of doublets of doublets), br = broad and the coupling constant values (*J*) are given in hertz (Hz). High resolution mass spectra (HRMS) were recorded on a LTQ Orbitrap XL spectrometer.

Melting points were determined using an Eclipse LV100N-POL polarized optical microscope from Nikon equipped with a heating stage HS82 from Mettler Toledo.

Molecule **7** suffers from significant side reactions during the one-pot reaction, to address this, we adopted a multistep approach.

We do not use PPA 105% in our current procedures. From our experience, PPA 105% exhibits greater variability between batches, often leading to undesired regioselectivity. In contrast, PPA 115% (supplier indicated) has shown more consistent performances, and we did not encounter these issues. To address these concerns, we re-optimized the reactions with Eaton's reagent (ER), which is more reliable, easier to handle and effectively resolves regioselectivity issues. All acids were stored in acid cabinets.

In the case compound **11**, the regioselectivity is dictated by the concentration of the reactant in ER. When the reactants are dissolved in ER (1M), both isomers are formed. While dissolving it in ER (5M) leads to the selective formation of **11**. Mechanistically, in the high acid amount (1M) scenario, two pathways occur. One, where the methacrylic acid is protonated and converted slowly into an acylium ion and one, where the acid is activated towards the 1,4-addition, resulting in the generation of both regioisomers. Reducing the amount of acidic medium further, (5M) scenario, just the methacrylic acid is protonated, therefore activating the double bond towards the 1,4-addition, ensuring regioselective product formation, due to the acylium ion formation slowed down even further.

For compounds **3** and **14**, achieving the opposite regioselectivity required dissolving the reagents in PPA 115%.

Compounds **13**, **17**, **18**, **21** and **23**, which require an intramolecular ring closing reaction, the strength of the acid appeared to correlate with the ring size and strain of the product. For

instance, indanones work with sulfuric acid (**13**), thiochroman-4-ones work with ER (**17**, **18**) and fluorenones work with PPA 115% (**21**, **23**).

All spectra of the synthesized compounds can be found in the appendix of the Supporting Information.

Table S1: Overview of optimized conditions.

Entry	Compound	Optimization
1	**1**[1]	change in work-up: distillation/column chromatography to remove non-NMR active reagents; solidifying with layered MeOH at -4 ºC
2	**2**[2]	in-house optimized
3	**3**[3]	milder conditions and change in work-up: quenching overnight; wash of organic layer with aq. $NaHCO_3$; no column/recrystallization
4	**4**[4]	change in work-up: no column/recrystallization; quantitative yield
5	**5**	new compound
6	**S1**[4]	adopted from literature
7	**6**[4]	change in work-up: column with pentane/CH_2Cl_2; dried *in vacuo* at 70 ºC
8	**S2**[5]	supply of procedure
9	**S3**[5]	supply of procedure; improved yields
10	**S4**[5]	supply of procedure; improved yields
11	**7**	overall yield improved from 5% to roughly 50% through 4-step procedure
12	**7b**	new compound
13	**8**	new compound
14	**9**	new procedure; change in conditions and work-up: use of methacryloyl chloride and $AlCl_3$; distillation; big scale; improved yields
15	**10**	new procedure; change in conditions: debromination with NaI and TMSCl; big scale; improved yields
16	**11**	new procedure; milder conditions; big scale
17	**12**[6]	change in work-up: trituration with *n*-pentane instead of column; improved yields
18	**S5**[7]	change in work-up: recrystallization instead of column
19	**S6**	new compound
20	**S7**	new compound
21	**S8**[8]	supply of procedure
22	**S9**[8]	change in work-up: no recrystallization
23	**13**[8]	change in conditions and work-up: no acid chloride formation; direct ring closure in H_2SO_4; no recrystallization
24	**14**[9]	change in conditions: PPA 115%; big scale; improved yields
25	**15**[9]	change in work-up: quenched with MeOH; washed with H_2O and dissolved acetone and dried over $MgSO_4$
26	**16**[10]	added for completeness
27	**S10**[2]	change in work-up: addition of 10% Et_2O in *n*-pentane and sonication with subsequent filtration
28	**17**	new procedure; change in conditions and work-up: no acid chloride formation; distillation
29	**S11**[11]	change in work-up: use of 1M HCl; addition of 10% Et_2O in *n*-pentane and sonication with subsequent filtration; big scale; improved yields
30	**18**	new procedure; change in conditions and work-up: no acid chloride formation; distillation; big scale; improved yields
31	**19**[11]	change in work-up: no wash with 1M HCl; report of analysis data
32	**S12**[12]	adopted from literature
33	**20**[12]	details to conditions and work-up to avoid accidents while performing the reaction
34	**S13**[13]	change in work-up: no column
35	**21**[14]	change in work-up: wash of organic layer with aq. $NaHCO_3$; filtration over silica plug; no column

36	**22**	new procedure; normally synthesized *via* different routes
37	**S14**[15]	change in conditions: use of H_2SO_4 and MeOH
38	**S15**	new procedure; improved yields
39	**23**	improved overall yield; less steps
40	**24**	new procedure
41	**25**[16]	change in work-up: column
42	**S16**[11]	change in work-up: trituration with MeOH; big scale
43	**S17**[11]	adopted from literature
44	**S18**[11]	change in conditions and work-up: *t*-BuLi instead of *s*-BuLi; recrystallization from EtOAc; no column
45	**26**[11]	change in work-up recrystallization; no column
46	**27**[11]	change in work-up: quenched with MeOH; washed with H_2O and dissolved acetone and dried over $MgSO_4$
47	**28**	new procedure; change in work-up to similar molecule: quench with 1M HCl and wash with aqueous LiCl (10% w/v); recrystallized from MeOH

Synthesis

5-Membered (Naphthalene-based) Top Half Ketones

2-methyl-2,3-dihydro-1*H*-cyclopenta[*a*]naphthalen-1-one (1)[1]

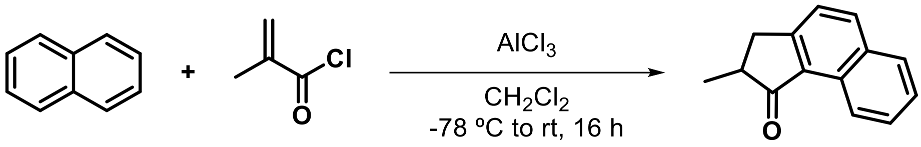

1

A flame-dried Schlenk tube equipped with a magnetic stirring egg was charged with CH_2Cl_2 (165 mL), $AlCl_3$ (13.7 g, 103 mmol, 2.0 eq.) and methacryloyl chloride (5.10 mL, 5.49 g, 52.5 mmol, 1.0 eq.). The mixture was cooled down to -78 °C (dry ice/EtOH) and naphthalene (6.52 g, 50.9 mmol, 1.0 eq.) was added portionwise. The resulting mixture was stirred and allowed to warm up to room temperature overnight. The mixture was hydrolyzed on ice/H_2O, extracted with CH_2Cl_2 (3x), washed with aqueous K_2CO_3 and the combined organic layers dried over Na_2SO_4. The solvent was removed under reduced pressure and the crude product purified *via* distillation (Kugelrohr, 5 – 6 mbar, 230 – 240 °C), yielding **1** as a yellow oil (7.03 g, 35.8 mmol, 70%).

The crude product can also be purified *via* a silica plug (*n*-pentane), yielding **1** as slight brown oil (quant.).

When the oil of **1** is layered with MeOH and stored at -4 °C, the oil transforms into an off-white solid.

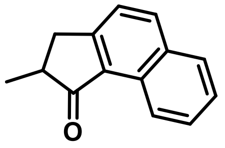

1
$C_{14}H_{12}O$
Mw = 196.25 g/mol

^{1}H NMR (400 MHz, $CDCl_3$): δ = 9.16 (d, *J* = 8.4 Hz, 1H), 8.05 (d, *J* = 8.4 Hz, 1H), 7.89 (d, *J* = 8.4 Hz, 1H), 7.67 (ddd, *J* = 8.3, 6.9, 1.4 Hz, 1H), 7.56 (ddd, *J* = 8.2, 6.9, 1.3 Hz, 1H), 7.50 (d, *J* = 8.3 Hz, 1H), 3.49 (dd, *J* = 18.1, 8.0 Hz, 1H), 2.87 – 2.80 (m, 2H), 1.38 (d, *J* = 7.3 Hz, 3H).
^{13}C{^{1}H} NMR (101 MHz, $CDCl_3$): δ = 210.2, 156.8, 135.9, 132.9, 130.4, 129.7, 129.0, 128.2, 126.7, 124.2, 124.1, 42.6, 35.5, 16.8.
HRMS-ESI (ESI+): calculated for $C_{14}H_{12}OH^+$ $[M+H]^+$ 196.0961, found 196.0957.

2-fluoro-2-methyl-2,3-dihydro-1*H*-cyclopenta[*a*]naphthalen-1-one (2)[2]

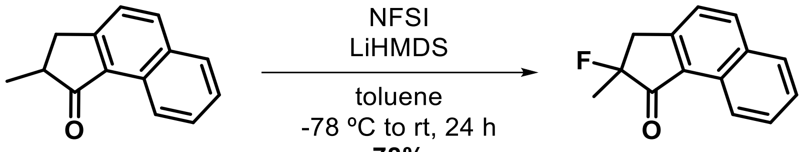

1 **2**

A flame-dried Schlenk tube equipped with a magnetic stirring egg was charged with toluene (20 mL) and LiHMDS (1M in THF, 7.6 mL, 1.27 g, 7.6 mmol, 2.2 eq.). The mixture was cooled down to -78 °C (dry ice/EtOH) and **1** (671 mg, 3.42 mmol, 1.0 eq.) dissolved in toluene (2.5 mL) was added dropwise. After stirring for 30 min at -78 °C, NFSI (2.49 g, 7.89 mmol, 2.3 eq.) was added portionwise and the resulting mixture stirred and allowed to warm up to room temperature overnight. The mixture was quenched with aqueous 1M HCl and extracted with CH_2Cl_2 (3x), washed with brine and dried over $MgSO_4$. The solvents were removed under reduced pressure and the crude product purified *via* flash column chromatography (*n*-pentane/EtOAc 19:1), yielding **2** as an orange oil (574 mg, 2.67 mmol, 78%).

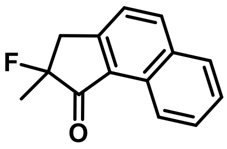

2
$C_{14}H_{11}FO$
Mw = 214.24 g/mol

^{1}H NMR (400 MHz, $CDCl_3$): δ = 9.03 (d, *J* = 8.4 Hz, 1H), 8.05 (d, *J* = 8.4 Hz, 1H), 7.85 (d, *J* = 8.1 Hz, 1H), 7.69 – 7.63 (m, 1H), 7.58 – 7.52 (m, 1H), 7.42 (d, *J* = 8.4 Hz, 1H), 3.56 – 3.44 (m, 1H), 3.39 – 3.31 (m, 1H), 1.67 (d, *J* = 22.8 Hz, 3H).
^{13}C{^{1}H} NMR (101 MHz, $CDCl_3$): δ = 201.5 (d), 153.4 (d), 137.5, 132.9, 129.6 (d), 129.5, 128.5, 127.7 (d), 127.1, 124.1, 123.7 (d), 95.7 (d), 40.8 (d), 21.9 (d).
^{19}F NMR (376 MHz, $CDCl_3$): δ = -151.2.
HRMS-ESI (ESI+): calculated for $C_{14}H_{11}FOH^+$ $[M+H]^+$ 237.0686, found 237.0686.

5-methoxy-2-methyl-2,3-dihydro-1*H*-cyclopenta[*a*]naphthalen-1-one (3)[3]

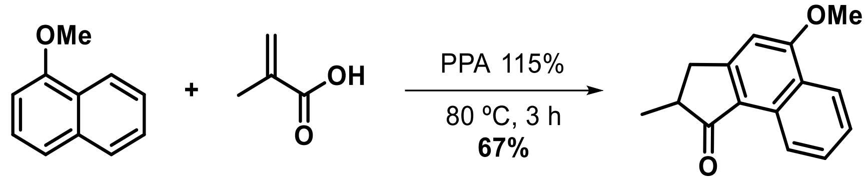

3

A round bottom flask equipped with a mechanical stirrer was charged with PPA 115% (~40 mL) and heated to 80 ºC. 1-methoxynaphthalene (6.5 mL, 7.12 g, 45.0 mmol, 1.0 eq.) was added and after mixing occurred, methacrylic acid (6.5 mL, 6.63 g, 77.0 mmol, 1.7 eq.) was added and stirring of the mixture was continued at 80 ºC for 3 h. The mixture was quenched on ice/H_2O, left stirring for 16 h and extracted with EtOAc. The organic layer was washed with H_2O and aqueous $NaHCO_3$, dried over $MgSO_4$ and the solvent removed under reduced pressure, yielding **3** as yellow/gold solid (6.79 g, 30.0 mmol, 67%).

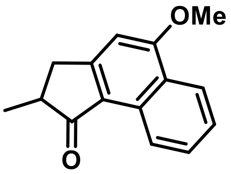

3
$C_{15}H_{14}O_2$
Mw = 226.28 g/mol

^{1}H NMR (600 MHz, $CDCl_3$): δ = 9.13 (d, *J* = 8.3 Hz, 1H), 8.25 (d, *J* = 8.4 Hz, 1H), 7.66 (ddd, *J* = 8.3, 6.8, 1.3 Hz, 1H), 7.52 (ddd, *J* = 8.3, 6.9, 1.3 Hz, 1H), 6.78 (s, 1H), 4.08 (s, 3H), 3.42 (dd, J = 18.0, 8.0 Hz, 1H), 2.81 – 2.73 (m, 2H), 1.36 (d, *J* = 7.3 Hz, 3H).
^{13}C{^{1}H} NMR (151 MHz, $CDCl_3$): δ = 208.5, 161.8, 159.3, 130.8, 129.4, 126.1, 125.3, 124.0, 123.6, 122.6, 101.6, 56.1, 42.3, 36.0, 17.1.
HRMS-ESI (APCI+): calculated for $C_{15}H_{14}O_2H^+$ $[M+H]^+$ 227.1067, found 227.1067.

5-hydroxy-2-methyl-2,3-dihydro-1*H*-cyclopenta[*a*]naphthalen-1-one (4)[4]

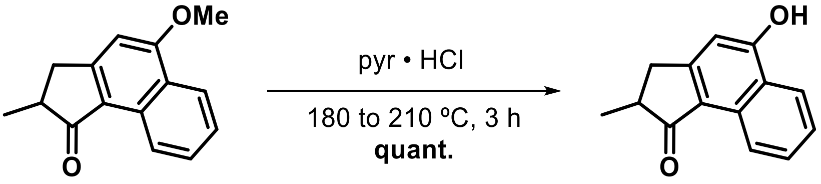

3 **4**

A round bottom flask equipped with a magnetic stirring egg was charged with pyridine HCl (53.5 g, 463 mmol, 29.9 eq.) and the mixture heated to 180 ºC. Ketone **3** (3.50 g, 15.5 mmol, 1.0 eq.) was added and the resulting mixture heated at 210 ºC for 3 h. The mixture was poured onto ice/H_2O, extracted with EtOAc, washed with H_2O (5x) and the solvent removed under reduced pressure, yielding **4** as a pale brown solid (3.28 g, 15.5 mmol, quant.).

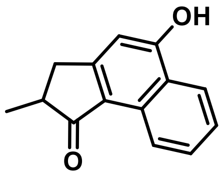

4
$C_{14}H_{12}O_2$
Mw = 212.25 g/mol

^{1}H NMR (600 MHz, $(CD_3)_2SO$): δ = 11.41 (s, 1H), 8.96 (d, *J* = 8.3 Hz, 1H), 8.20 (d, *J* = 8.3 Hz, 1H), 7.67 (ddd, *J* = 8.3, 6.9, 1.3 Hz, 1H), 7.53 (ddd, *J* = 8.3, 6.9, 1.3 Hz, 1H), 6.92 (s, 1H), 3.37 (dd, *J* = 17.6, 7.5 Hz, 1H), 2.74 – 2.64 (m, 2H), 1.21 (d, *J* = 7.4 Hz, 3H).
^{13}C{^{1}H} NMR (151 MHz, $(CD_3)_2SO$): δ = 207.0, 160.4, 159.8, 130.5, 129.2, 125.4, 124.1, 122.9, 122.8, 121.1, 105.7, 41.4, 34.9, 16.6.
HRMS-ESI (ESI-): calculated for $C_{14}H_{11}O_2^-$ $[M\text{-}H]^-$ 211.0765, found 211.0764.

Methyl 2-((2-methyl-1-oxo-2,3-dihydro-1*H*-cyclopenta[*a*]naphthalen-5-yl)oxy)acetate (5)

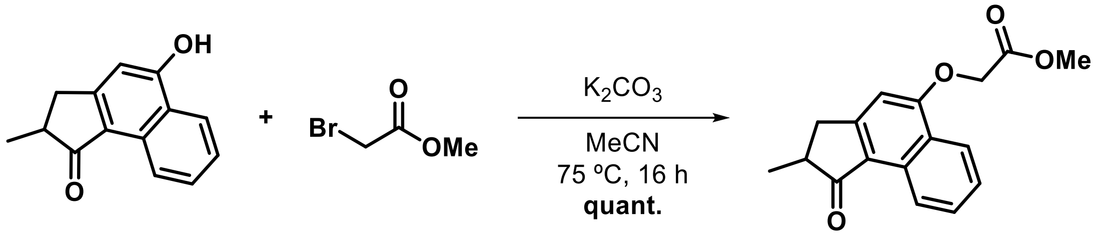

4 **5**

An oven-dried crimp top vial equipped with a magnetic stirring bar was charged with **4** (1.04 g, 4.90 mmol, 1.0 eq.) and K_2CO_3 (0.86 g, 6.24 mmol, 1.25 eq.). The vial was crimped, flushed with nitrogen and methyl 2-bromoacetate (0.88 mL, 1.09 g, 7.11 mmol, 1.45 eq.) and dry MeCN (25 mL) were added. The mixture was heated at 75 °C for 16 h, followed by heating at 85 °C for 1 h. The mixture was allowed to cool down to room temperature, H_2O was added and the mixture extracted with CH_2Cl_2 (3x). The combined organic layers were dried over Na_2SO_4, the solvent removed under reduced pressure and the crude product purified *via* flash column chromatography (gradient *n*-pentane to EtOAc), yielding **5** as an off-white solid (1.39 g, 4.89 mmol, quant.).

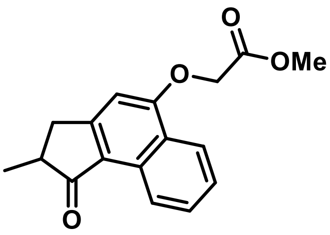

5
$C_{17}H_{16}O_4$
Mw = 284.31 g/mol

^{1}H NMR (400 MHz, $CDCl_3$): δ = 9.13 (d, *J* = 8.3 Hz, 1H), 8.35 (d, *J* = 8.3 Hz, 1H), 7.69 (ddd, *J* = 8.3, 6.9, 1.3 Hz, 1H), 7.56 (ddd, *J* = 8.3, 6.9, 1.3 Hz, 1H), 6.65 (s, 1H), 4.90 (s, 2H), 3.86 (s, 3H), 3.41 (dd, *J* = 18.0, 8.0 Hz, 1H), 2.84 – 2.70 (m, 2H), 1.35 (d, *J* = 7.3 Hz, 3H).
^{13}C{^{1}H} NMR (101 MHz, $CDCl_3$): δ = 208.5, 168.6, 159.7, 158.6, 130.8, 129.7, 126.4, 125.2, 124.6, 124.0, 122.7, 102.4, 65.6, 52.7, 42.4, 35.9, 16.9.
HRMS-ESI (ESI+): calculated for $C_{17}H_{16}O_4H^+$ $[M+H]^+$ 285.1121, found 285.1118.
Mp: 145 – 146 °C.

(3-bromoprop-1-yn-1-yl)triisopropylsilane (S1)[4]

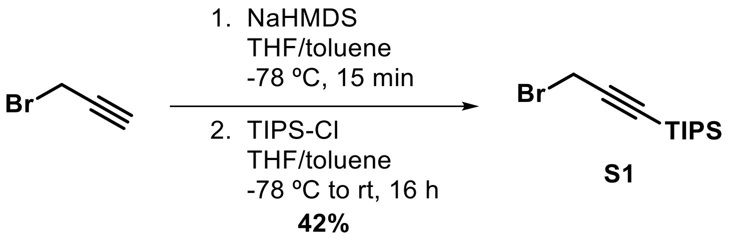

A flame-dried Schlenk tube equipped with a magnetic stirring egg was charged with THF (97 mL) and NaHMDS (1M in THF, 45.0 mL, 8.25 g, 45.0 mmol, 1.0 eq.) The mixture was cooled down to -78 °C (dry ice/EtOH) and propargyl bromide (80% solution in toluene, 4.9 mL, 7.82 g, 52.6 mmol, 1.2 eq.) was added dropwise. After 10 min, TIPS-Cl (9.5 mL, 8.56 g, 44.4 mmol, 1.0 eq.) was added dropwise and the resulting mixture was allowed to warm up to room temperature after 15 min and stirred overnight. The mixture was quenched with aqueous NH_4Cl and extracted with CH_2Cl_2 (3x), the combined organic layers dried over Na_2SO_4 and the solvent removed under reduced pressure. The crude product was purified *via* flash column chromatography with (*n*-pentane), yielding **S1** as slight yellow oil (5.10 g, 18.5 mmol, 42%)

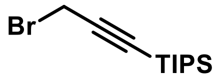

S1
$C_{12}H_{23}BrSi$
Mw = 275.31 g/mol

^{1}H NMR (400 MHz, $CDCl_3$): δ = 3.95 (s, 2H), 1.07 (s, 21H).
^{13}C{^{1}H} NMR (101 MHz, $CDCl_3$): δ = 101.8, 89.3, 18.7, 15.1, 11.3.

2-methyl-5-((3-(triisopropylsilyl)prop-2-yn-1-yl)oxy)-2,3-dihydro-1*H*-cyclopenta[a]naphthalen-1-one (6)[4]

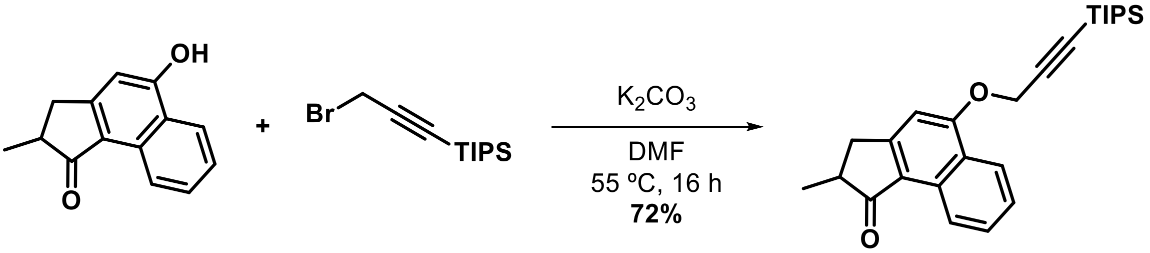

4 **S1** **6**

An oven-dried crimp top vial equipped with a magnetic stirring bar was charged with **4** (1.31 g, 6.17 mmol, 1.0 eq.), **S1** (2.02 g, 7.35 mmol, 1.2 eq.) and K_2CO_3 (3.59 g, 26.0 mmol, 4.2 eq.). The vial was crimped, flushed with nitrogen and DMF (30 mL) added. The mixture was heated at 55 °C for 16 h. The mixture was allowed to cool down to room temperature, the solvent removed under reduced pressure, H_2O added and extracted with CH_2Cl_2 (3x). The solvent was removed under reduced pressure, the crude product purified *via* flash column chromatography (gradient *n*-pentane to CH_2Cl_2) and dried *in vacuo* at 70 °C, yielding **6** as a colorless oil which solidifies over time (1.80 g, 4.43 mmol, 72%).

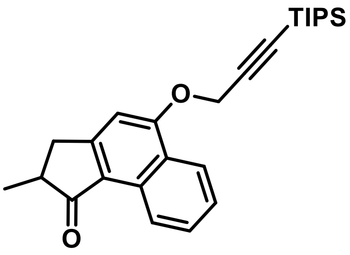

6
$C_{26}H_{34}O_2Si$
Mw = 406.64 g/mol

^{1}H NMR (400 MHz, $CDCl_3$): δ = 9.13 (d, *J* = 8.3 Hz, 1H), 8.26 (d, *J* = 8.4 Hz, 1H), 7.67 (ddd, *J* = 8.3, 6.9, 1.4 Hz, 1H), 7.53 (ddd, *J* = 8.3, 6.9, 1.3 Hz, 1H), 7.01 (s, 1H), 5.01 (s, 2H), 3.45 – 3.34 (m, 1H), 2.84 – 2.69 (m, 2H), 1.35 (d, *J* = 7.3 Hz, 3H), 1.04 (s, 21H).
^{13}C{^{1}H} NMR (101 MHz, $CDCl_3$): δ = 208.6, 159.6, 158.8, 130.8, 129.4, 126.1, 125.4, 123.9, 122.7, 103.7, 100.9, 90.8, 57.4, 42.4, 35.9, 18.6, 17.1, 11.2.
HRMS-ESI (ESI+): calculated for $C_{26}H_{34}O_2SiH^+$ $[M+H]^+$ 407.2401, found 407.2396.

(4-bromonaphthalen-1-yl)trimethylsilane (S2)[5]

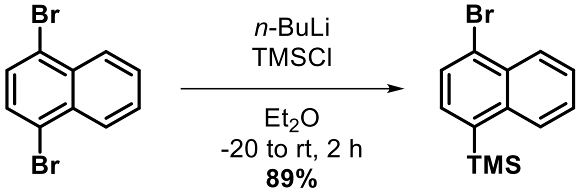

S2

A flame-dried Schlenk tube equipped with a magnetic stirring egg was charge with 1,4-dibromonaphthalene (18.6 g, 65.0 mmol, 1.0 eq.), Et_2O (195 mL) and the solution was cooled down -20 ºC (NaCl/ice/H_2O). *n*-BuLi (1.6M in hexanes, 43.7 mL, 4.48 g, 69.9 mmol, 1.1 eq.) was added dropwise and the mixture stirred at -20 ºC for 1 h. TMSCl (14.2 mL, 12.2 g, 112 mmol, 1.7 eq.) was added dropwise and the mixture allowed to warm up to room temperature and stirred for 30 min. H_2O was added and the mixture extracted with Et_2O (2x), the combined organic layers dried over $MgSO_4$ and the solvent removed under reduced pressure, yielding **S2** as slight brown crystals (16.1 g, 57.3 mmol, 89%).

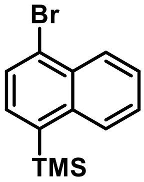

S2
$C_{13}H_{15}BrSi$
Mw = 279.25 g/mol

^{1}H NMR (600 MHz, $CDCl_3$): δ = 8.34 – 8.30 (m, 1H), 8.09 (dd, *J* = 8.0, 0.9 Hz, 1H), 7.75 (d, *J* = 7.4 Hz, 1H), 7.61 – 7.54 (m, 2H), 7.50 (d, *J* = 7.4 Hz, 1H), 0.46 (s, 9H).
^{13}C{^{1}H} NMR (151 MHz, $CDCl_3$): δ = 138.7, 138.3, 133.5, 132.0, 129.5, 128.7, 128.3, 126.9, 126.6, 125.4, 0.3.

1-(4-bromonaphthalen-1-yl)-3-chloropropan-1-one (S3)[5]

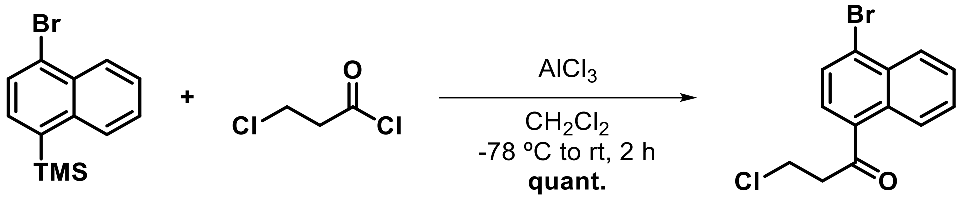

S2 **S3**

A flame-dried Schlenk tube equipped with a magnetic stirring bar was charged with CH_2Cl_2 (350 mL), $AlCl_3$ (8.51 g, 63.8 mmol, 1.2 eq.) and 3-chloropropanoyl chloride (5.6 mL, 7.49 g, 59.0 mmol, 1.1 eq.) and was cooled down to -78 °C (dry ice/EtOH). **S2** (15.0 g, 53.7 mmol, 1.0 eq.) dissolved in CH_2Cl_2 (50 mL) was added and the mixture allowed to warm up to room temperature and stirred for 2 h. A mixture of ice/H_2O/HCl was added and extracted with CH_2Cl_2. The organic layer was dried over Na_2SO_4 and the solvent removed under reduced pressure, yielding **S3** as brown oil (15.9 g, 53.4 mmol, quant.).

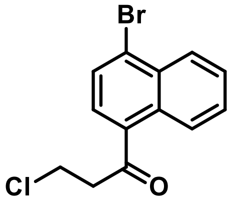

S3
$C_{13}H_{10}BrClO$
Mw = 297.58 g/mol

^{1}H NMR (600 MHz, $CDCl_3$): δ = 8.64 – 8.60 (m, 1H), 8.37 – 8.31 (m, 1H), 7.85 (d, *J* = 7.8 Hz, 1H), 7.70 (d, *J* = 7.8 Hz, 1H), 7.68 – 7.64 (m, 2H), 3.98 (t, *J* = 6.5 Hz, 2H), 3.51 (t, *J* = 6.5 Hz, 2H).
^{13}C{^{1}H} NMR (151 MHz, $CDCl_3$): δ = 200.1, 135.2, 132.6, 131.3, 129.1, 128.8, 128.7, 128.2, 127.8, 127.8, 126.3, 44.6, 39.1.
HRMS-ESI (ESI+): calculated for $C_{13}H_{10}BrClOH^+$ $[M+H]^+$ 296.9676, found 296.9675.

5-bromo-2,3-dihydro-1*H*-cyclopenta[*a*]naphthalen-1-one (S4)[5]

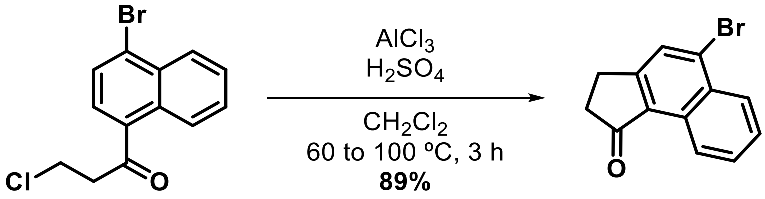

S3 **S4**

A round bottom flask equipped with a magnetic stirring egg was charge with **S3** (15.9 g, 53.4 mmol, 1.0 eq.) and CH_2Cl_2 (15 mL). $AlCl_3$ (8.54 g, 64.1 mmol, 1.2 eq.) and H_2SO_4 (80 mL) were added slowly and consecutively and the mixture was heated at 60 °C for 30 min (until no gas formation was observed). The mixture was heated at 100 °C for 2½ h before being allowed to cool down to room temperature. The mixture was quenched on ice/H_2O and extracted with CH_2Cl_2. The organic layer was washed with aqueous $NaHCO_3$ and dried over $MgSO_4$, yielding **S4** as a pale brown solid (12.4 g, 47.4 mmol, 89%).

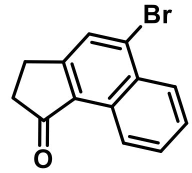

S4
$C_{13}H_9BrO$
Mw = 261.12 g/mol

^{1}H NMR (600 MHz, $CDCl_3$): δ = 9.21 (d, *J* = 8.0 Hz, 1H), 8.31 (d, *J* = 8.4 Hz, 1H), 7.90 (s, 1H), 7.72 (ddd, *J* = 8.3, 6.9, 1.3 Hz, 1H), 7.66 (ddd, *J* = 8.3, 6.9, 1.3 Hz, 1H), 3.24 – 3.20 (m, 2H), 2.83 – 2.80 (m, 2H).
^{13}C{^{1}H} NMR (151 MHz, $CDCl_3$): δ = 206.7, 158.2, 131.8, 131.2, 130.8, 130.3, 129.9, 128.6, 128.0, 127.7, 124.4, 37.1, 26.0.
HRMS-ESI (ESI+): calculated for $C_{13}H_9BrOH^+$ $[M+H]^+$ 260.9910, found 260.9908.

5-bromo-2-methyl-2,3-dihydro-1*H*-cyclopenta[*a*]naphthalen-1-one (7)

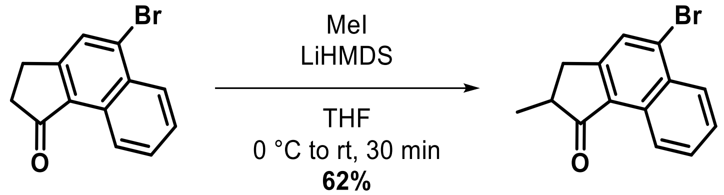

S4 **7**

A flame-dried Schlenk tube equipped with a magnetic stirring egg was charged with THF (20 mL), **S4** (1.00 g, 3.83 mmol, 1.0 eq.) and the mixture cooled down to 0 ºC (ice/H_2O). LiHMDS (1M in THF, 4.2 mL, 0.71 g, 4.21 mmol, 1.1 eq.) was added dropwise and the mixture stirred for 20 min at 0 ºC (ice/H_2O). MeI (11.9 mL, 27.2 g, 192 mmol, 50.0 eq.) dissolved in THF (20 mL) was cooled down to 0 ºC (ice/H_2O) and the first mixture added dropwise to it *via* a canula. After addition, the mixture was allowed to warm up to room temperature and was stirred for 30 min. The mixture was quenched with saturated aqueous NH_4OH, extracted with Et_2O (3x) and the combined organic layers dried over $MgSO_4$. The solvents were removed under reduced pressure and the crude product purified *via* flash column chromatography (gradient *n*-pentane to *n*-pentane/EtOAc 9:1), yielding **7** as an off-white solid (0.65 g, 2.36 mmol, 62%).

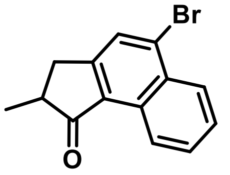

7
$C_{14}H_{11}BrO$
Mw = 275.15 g/mol

^{1}H NMR (400 MHz, $CDCl_3$): δ = 9.16 (d, *J* = 8.2 Hz, 1H), 8.26 (d, *J* = 8.4 Hz, 1H), 7.82 (s, 1H), 7.69 (t, *J* = 7.6 Hz, 1H), 7.63 (t, *J* = 7.7 Hz, 1H), 3.42 (dd, *J* = 18.2, 7.9 Hz, 1H), 2.77 (d, *J* = 14.9 Hz, 2H), 1.36 (d, *J* = 7.3 Hz, 3H).
^{13}C{^{1}H} NMR (101 MHz, $CDCl_3$): δ = 209.2, 156.4, 131.7, 131.2, 130.3, 129.9, 129.7, 128.4, 127.9, 127.6, 124.4, 42.6, 35.1, 16.7.
Mp: 88 – 89 °C.

5-bromo-2,2-dimethyl-2,3-dihydro-1H-cyclopenta[a]naphthalen-1-one (7b)

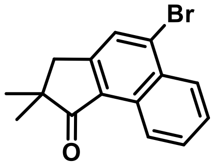

7b
$C_{15}H_{13}BrO$
Mw = 289.17 g/mol

When LDA was used as a base, **7b** was obtained as a side product with similar yields during the methylation of **S4** to obtain **7**.

^{1}H NMR (400 MHz, $CDCl_3$): δ = 9.19 (d, *J* = 8.2 Hz, 1H), 8.29 (d, *J* = 8.3 Hz, 1H), 7.84 (s, 1H), 7.75 – 7.60 (m, 2H), 3.07 (s, 2H), 1.29 (s, 6H).
^{13}C{^{1}H} NMR (101 MHz, $CDCl_3$): δ = 211.2, 155.1, 131.9, 131.4, 130.7, 129.7, 128.8, 128.5, 127.9, 127.6, 124.5, 46.1, 43.0, 25.6.
HRMS-ESI (ESI+): calculated for $C_{15}H_{13}BrOH^+$ $[M+H]^+$ 289.0223, found 289.0223.
Mp: 96 – 98 °C.

5-iodo-2-methyl-2,3-dihydro-1*H*-cyclopenta[*a*]naphthalen-1-one (8)

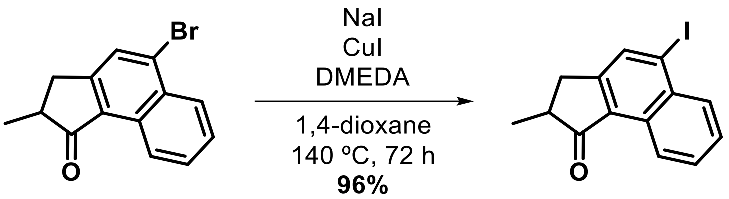

7 **8**

An oven-dried crimp top vial equipped with a magnetic stirring bar was charged with **7** (105 mg, 0.38 mmol, 1.0 eq.), NaI (229 mg, 1.53 mmol, 4.0 eq.) and CuI (14.5 mg, 76.1 µmol, 20 mol%). The vial was crimped, flushed with nitrogen and 1,4-dioxane (degassed by N_2; 10 mL) and DMEDA (40 µL, 32.7 mg, 0.37 mmol, 1.0 eq.) added. The mixture was heated at 140 °C for 72 h. The mixture was allowed to cool down to room temperature, H_2O and brine was added and the mixture extracted with EtOAc (3x). The combined organic layers were dried over $MgSO_4$ and the solvent was removed under reduced pressure. The crude product was purified *via* recrystallization (*n*-heptane), yielding **8** as an off-white solid (118 mg, 0.37 mmol, 96%).

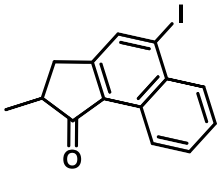

8
$C_{14}H_{11}IO$
Mw = 322.15 g/mol

^{1}H NMR (400 MHz, $CDCl_3$): δ = 9.13 (d, *J* = 8.2 Hz, 1H), 8.20 (s, 1H), 8.15 (d, *J* = 8.4 Hz, 1H), 7.69 (ddd, *J* = 8.3, 6.9, 1.3 Hz, 1H), 7.62 (ddd, *J* = 8.3, 6.9, 1.4 Hz, 1H), 3.43 (dd, *J* = 18.2, 8.1 Hz, 1H), 2.84 – 2.74 (m, 2H), 1.37 (d, *J* = 7.3 Hz, 3H).
^{13}C{^{1}H} NMR (101 MHz, $CDCl_3$): δ = 209.4, 156.6, 136.1, 133.6, 132.6, 130.8, 129.8, 129.6, 128.3, 124.5, 110.1, 42.7, 34.8, 16.7.
HRMS-ESI (ESI+): calculated for $C_{14}H_{11}IOH^+$ $[M+H]^+$ 322.9927, found 322.9925.
Mp: 99 – 101 °C.

5-Membered (Xylene-based) Top Half Ketones

2,4,7-trimethyl-2,3-dihydro-1*H*-inden-1-one (9)

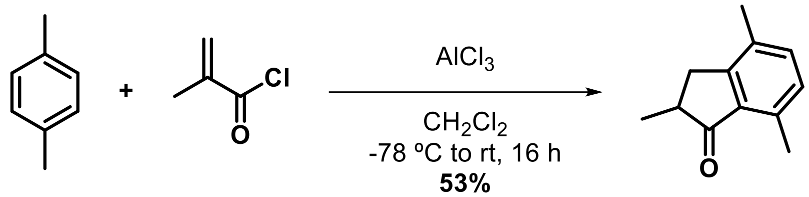

9

A flame-dried Schlenk tube equipped with a magnetic stirring egg was charged with CH_2Cl_2 (750 mL), $AlCl_3$ (101 g, 754 mmol, 2.0 eq.) and the mixture cooled down to -78 °C (dry ice/EtOH). Methacryloyl chloride (36.5 mL, 39.4 g, 377 mmol, 1.0 eq.) was added dropwise and the suspension stirred for 10 min at -78 °C. *p*-Xylene (46.2 mL, 40.0 g, 377 mmol, 1.0 eq.) was added dropwise. The resulting mixture was stirred and allowed to warm up to room temperature overnight. The mixture was hydrolyzed on ice/H_2O, extracted with CH_2Cl_2 (2x), washed with aqueous $NaHCO_3$, brine and the combined organic layers dried over $MgSO_4$. The solvent was removed under reduced pressure and the crude product purified *via* distillation (Kugelrohr, 1 mbar, 140 – 150 °C), yielding **9** as a slightly yellow oil (35.0 g, 201 mmol, 53%).

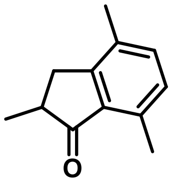

9
$C_{12}H_{14}O$
Mw = 174.24 g/mol

^{1}H NMR (400 MHz, $CDCl_3$): δ = 7.24 (d, *J* = 7.5 Hz, 1H), 7.02 (d, *J* = 7.5 Hz, 1H), 3.24 (dd, *J* = 17.1, 8.0 Hz, 1H), 2.71 – 2.63 (m, 1H), 2.60 (s, 3H), 2.54 (dd, *J* = 17.1, 4.1 Hz, 1H), 2.29 (s, 3H), 1.30 (d, *J* = 7.4 Hz, 3H).
^{13}C{^{1}H} NMR (101 MHz, $CDCl_3$): δ = 210.9, 153.1, 136.3, 134.6, 133.6, 132.7, 129.4, 42.2, 33.6, 18.1, 17.6, 16.7.
HRMS-ESI (ESI+): calculated for $C_{12}H_{14}OH^+$ $[M+H]^+$ 175.1117, found 175.1117.

6-bromo-2,4,7-trimethyl-2,3-dihydro-1*H*-inden-1-one (10)

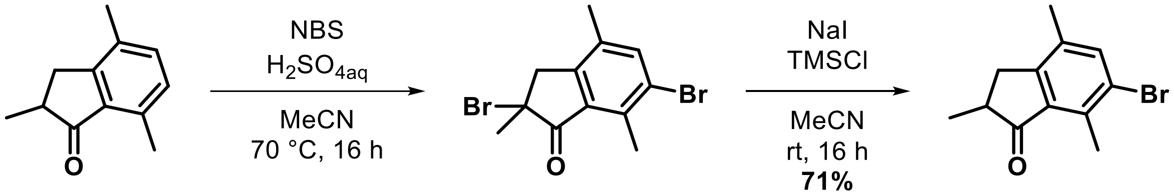

9 **10**

A round bottom flask equipped with a magnetic stirring egg was charged with **9** (23.1 g, 133 mmol, 1.0 eq.) and MeCN (400 mL). Subsequently, aqueous H_2SO_4 (10% v/v, 450 mL) and NBS (59.0 g, 331 mmol, 2.5 eq.), portionwise, were added and the mixture heated to 70 ºC overnight. The mixture was allowed to cool down to room temperature, poured on H_2O, extracted with EtOAc (3x) and the solvent removed under reduced pressure. The crude product was used without further purification in the next reaction.

The crude product was dissolved in MeCN (270 mL), NaI (140 g, 931 mmol, 7.0 eq.) and TMSCl (50.8 g, 466 mmol, 3.5 eq.) were added and the mixture stirred at room temperature overnight. H_2O was added, the mixture extracted with EtOAc (3x) and the combined organic layers washed with saturated aqueous $Na_2S_2O_3$ and brine. The solvent was removed under reduced pressure and the crude product redissolved in MeCN and crystallized at -25 ºC, yielding **10** as an off-white solid.

A second batch was obtained by concentrating the mother liquor and purifying it *via* flash column chromatography (gradient *n*-pentane to 4% CH_2Cl_2), yielding **10** as an off-white solid (24.0 g, 94.8 mmol, 71% over two steps).

To obtain the (*R*)- or (*S*)-enantiomer,[6] a flame-dried Schlenk flask equipped with a magnetic stirring egg was charged THF and DIPA (1.1 eq.) and cooled down to 0 ºC. *n*-BuLi (1.1 eq.) was added dropwise and the mixture stirred for 15 min. The mixture was cooled down to -78 ºC (dry ice/EtOH) and added to a solution of **10** (1.0 eq.) in THF at -78 ºC and stirred for 2 h. TMSCl (1.2 eq.) was added and after addition the mixture was allowed to warm up to room temperature and stirred for an additional 2 h. The mixture was hydrolyzed with aqueous $NaHCO_3$ and extracted with Et_2O (3x), the combined organic layers washed with brine, filtered over a plug of silica, dried over Na_2SO_4 and concentrated under reduced pressure.

(*R*)- or (*S*)-BINAP$(AuCl)_2$ (0.03 eq.) dissolved in CH_2Cl_2 and $AgBF_4$ (0.03 eq.) in EtOH were mixed at room temperature and stirred for 45 min. The mixture was cooled down to 0 ºC (ice/H_2O) and added to the prior concentrated solution in Et_2O at 0 ºC (ice/H_2O). The mixture was allowed to warm up to room temperature and stirred overnight. The mixture was hydrolyzed with aqueous $NaHCO_3$ and extracted with Et_2O (3x), the combined organic layers dried over Na_2SO_4 and the solvent removed under reduced pressure. The crude product was purified *via* flash column chromatography (gradient *n*-pentane to EtOAc), yielding **(*R*)-** or **(*S*)-10** (chiral HPLC: Chiralpak AS-H, *n*-heptane/*i*PrOH 98:2, 0.5 mL/min, 40 ºC; 98% *ee*).

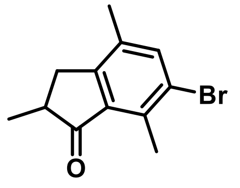

10
$C_{12}H_{13}BrO$
Mw = 253.14 g/mol

^{1}H NMR (400 MHz, $CDCl_3$): δ = 7.54 (s, 1H), 3.17 (dd, *J* = 17.2, 8.0 Hz, 1H), 2.68 (s, 4H), 2.47 (dd, *J* = 17.3, 4.1 Hz, 1H), 2.28 (s, 3H), 1.30 (d, *J* = 7.4 Hz, 3H).
^{13}C{^{1}H} NMR (101 MHz, $CDCl_3$): δ = 209.7, 152.5, 138.0, 135.9, 134.8, 134.4, 125.1, 42.4, 32.9, 17.3, 16.7, 16.7.
HRMS-ESI (ESI+): calculated for $C_{12}H_{13}BrOH^+$ [M+H]$^+$ 253.0223, found 253.0223.

6-methoxy-2,4,7-trimethyl-2,3-dihydro-1*H*-inden-1-one (11)

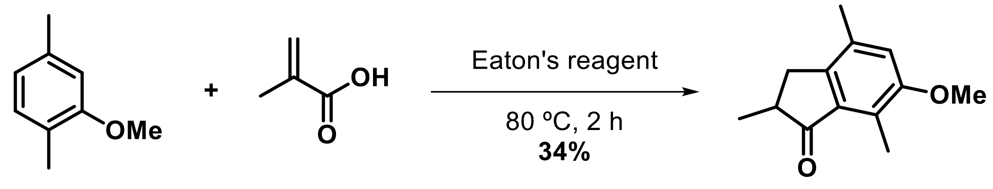

11

A round bottom flask equipped with a magnetic stirring egg was charged with 2-methoxy-1,4-dimethylbenzene (39.3 g, 288 mmol, 1.0 eq.), methacrylic acid (48.9 mL, 49.7 g, 577 mmol, 2.0 eq.) and Eaton's reagent (58 mL) and the mixture heated at 80 ºC and stirred for 2 h. The mixture was allowed to cool down to room temperature, poured on ice/H_2O, aqueous 4M NaOH added and the mixture extracted with EtOAc (3x). The combined organic layers were washed with aqueous 1M NaOH, dried over $MgSO_4$, the solvent removed under reduced pressure and the crude product purified *via* recrystallization (MeOH), yielding **11** as an off-white solid (20.0 g, 97.9 mmol, 34%).

Smaller scale reactions tend to have higher yields.

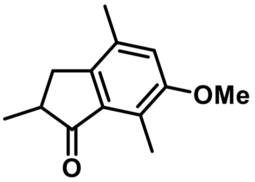

11
$C_{13}H_{16}O_2$
Mw = 204.27 g/mol

^{1}H NMR (400 MHz, $CDCl_3$): δ = 6.91 (s, 1H), 3.84 (s, 3H), 3.16 (dd, *J* = 16.7, 8.0 Hz, 1H), 2.72 – 2.59 (m, 1H), 2.50 (s, 3H), 2.46 (dd, *J* = 17.0, 4.4 Hz, 1H), 2.29 (s, 3H), 1.29 (d, *J* = 7.4 Hz, 3H).
^{13}C{^{1}H} NMR (101 MHz, $CDCl_3$): δ = 211.2, 157.1, 144.5, 134.3, 132.9, 124.2, 117.9, 56.5, 42.9, 32.6, 18.0, 16.7, 9.8.
HRMS-ESI (ESI+): calculated for $C_{13}H_{16}O_2H^+$ [M+H]$^+$ 205.1223, found 205.1223.

6-hydroxy-2,4,7-trimethyl-2,3-dihydro-1*H*-inden-1-one (12)[6]

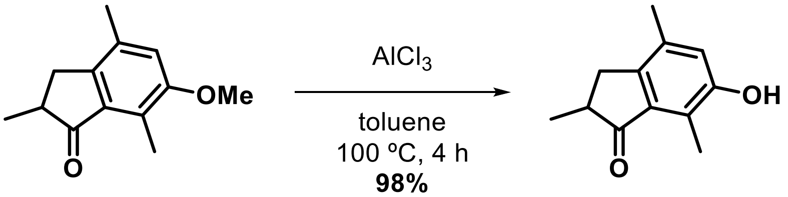

11 **12**

A flame-dried 3-neck round bottom flask equipped with a magnetic stirring egg was charged with toluene (100 mL) and **11** (5.00 g, 24.5 mmol, 1.0 eq.). $AlCl_3$ (9.79 g, 73.4 mmol, 3.0 eq.) was added slowly and the suspension heated at 100 ºC and stirred for 4 h. The mixture was allowed to cool down to room temperature, poured slowly onto ice and extracted with EtOAc (3x). The combined organic layers were washed with brine, dried over $MgSO_4$, the solvent removed under reduced pressure and the obtained solid triturated with *n*-pentane, yielding **12** as a red solid (4.55 g, 23.9 mmol, 98%).

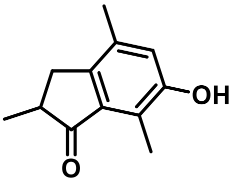

12
$C_{12}H_{14}O_2$
Mw = 190.24 g/mol

^{1}H NMR (400 MHz, $CDCl_3$): δ = 6.91 (s, 1H), 5.14 (s, 1H), 3.15 (dd, *J* = 16.8, 7.9 Hz, 1H), 2.72 – 2.62 (m, 1H), 2.53 (s, 3H), 2.45 (dd, *J* = 16.7, 3.9 Hz, 1H), 2.25 (s, 3H), 1.29 (d, *J* = 7.4 Hz, 3H).
^{13}C{^{1}H} NMR (101 MHz, $CDCl_3$): δ = 211.4, 153.3, 145.8, 134.4, 133.5, 122.5, 120.2, 42.9, 32.6, 17.7, 16.7, 9.4.
HRMS-ESI (ESI+): calculated for $C_{12}H_{14}O_2H^+$ $[M+H]^+$ 191.1067, found 191.1067.

(2-bromo-5-methylphenyl)methanol (S5)[7]

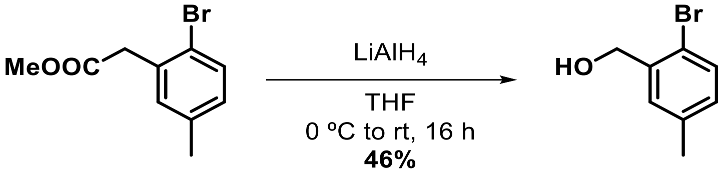

S5

A flame-dried Schlenk tube equipped with a magnetic stirring egg was charged with methyl-2-(2-bromo-5-methylphenyl)acetate (6.87 g, 30.0 mmol, 1.0 eq.) and THF (45 mL). The mixture was cooled down to 0 °C (ice/water) and $LiAlH_4$ (1M in THF, 18 0mL, 0.68 g, 18.0 mmol, 0.6 eq.) added. The mixture was allowed to warm up to room temperature and stirred overnight. EtOAc was added and the mixture quenched by slow addition of H_2O. The mixture was stirred until it turned white, the solids filtered off, the organic layer separated and dried over $MgSO_4$. The crude product purified *via* recrystallization (*n*-heptane), yielding **S5** as a colorless solid (2.80 g, 13.9 mmol, 46%).

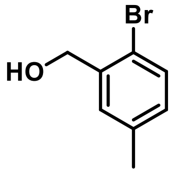

S5
C_8H_9BrO
Mw = 201.06 g/mol

^{1}H NMR (400 MHz, $CDCl_3$): δ = 7.36 (d, *J* = 8.1 Hz, 1H), 7.24 (d, *J* = 2.3 Hz, 1H), 6.92 (dd, *J* = 8.2, 2.3 Hz, 1H), 4.63 (s, 2H), 3.28 (br s, 1H), 2.28 (s, 3H).
^{13}C{^{1}H} NMR (101 MHz, $CDCl_3$): δ = 139.2, 137.5, 132.2, 129.7, 129.4, 119.0, 64.6, 21.0.

2-bromo-5-methylbenzyl 4-methylbenzenesulfonate (S6)

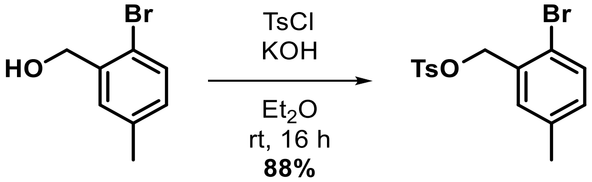

S5 **S6**

A round bottom flask equipped with a magnetic stirring bar was charged with **S5** (2.60 g, 12.9 mmol, 1.0 eq.), Et_2O (65 mL) and TsCl (2.96 g, 15.5 mmol, 1.2 eq.). KOH (8.54 g, 129 mmol, 10.0 eq.) was added slowly and the mixture stirred at room temperature overnight. The mixture was filtered, extracted with H_2O, washed with brine and the organic layer dried over $MgSO_4$. The solvent was removed under reduced pressure and the compound triturated with *n*-pentane, yielding **S6** as a colorless solid (4.05 g, 11.4 mmol, 88%).

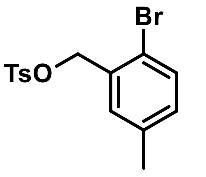

S6
$C_{15}H_{15}BrO_3S$
Mw = 355.25 g/mol

^{1}H NMR (400 MHz, $CDCl_3$): δ = 7.81 (d, *J* = 8.3 Hz, 2H), 7.37 – 7.29 (m, 3H), 7.14 (d, *J* = 2.2 Hz, 1H), 6.97 (dd, *J* = 8.2, 2.2 Hz, 1H), 5.09 (s, 2H), 2.43 (s, 3H), 2.25 (s, 3H).
^{13}C{^{1}H} NMR (101 MHz, $CDCl_3$): δ = 145.0, 137.7, 133.0, 132.6, 132.4, 131.3, 131.2, 129.9, 128.1, 120.0, 71.2, 21.7, 20.8.
HRMS-ESI (ESI+): calculated for $C_{15}H_{15}BrO_3SNa^+$ $[M+Na]^+$ 376.9817, found 376.9816.

Diethyl 2-(2-bromo-5-methylbenzyl)-2-methylmalonate (S7)

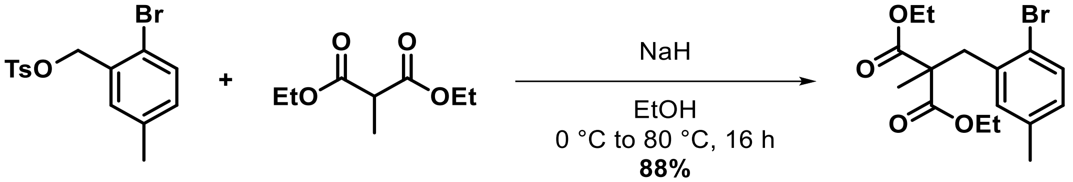

S6 **S7**

A flame-dried Schlenk tube equipped with a magnetic stirring egg was charged with EtOH (35 mL) and cooled down to 0 ºC (ice/water). NaH (60% dispersion in mineral oil, 0.36 g, 9.15 mmol, 1.3 eq.) was added portionwise and the suspension allowed to warm up to room temperature. Diethyl 2-methylmalonate (1.3 mL, 1.31 g, 7.39 mmol, 1.05 eq.) was added and the mixture stirred for 30 min. Subsequently, **S6** (2.50 g, 7.04 mmol, 1.0 eq.) was added, the mixture heated at 80 ºC and stirred overnight. The mixture was allowed to cool down to room temperature, EtOAc added and the mixture quenched by slow addition of H_2O and extracted. The organic layer was washed with brine, dried over $MgSO_4$. The solvent was removed under reduced pressure and the crude product purified *via* flash column chromatography (gradient *n*-pentane to 10% EtOAc), yielding **S7** as a colorless oil (2.20 g, 6.16 mmol, 88%).

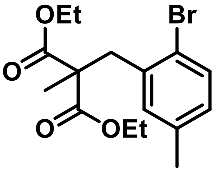

S7
$C_{16}H_{21}BrO_4$
Mw = 357.24 g.mol

^{1}H NMR (400 MHz, $CDCl_3$): δ = 7.35 (d, *J* = 8.1 Hz, 1H), 6.92 (d, *J* = 2.3 Hz, 1H), 6.84 (dd, *J* = 8.1, 2.3 Hz, 1H), 4.26 – 4.10 (m, 4H), 3.42 (s, 2H), 2.19 (s, 3H), 1.34 (s, 3H), 1.23 (t, *J* = 7.2 Hz, 6H).
^{13}C{^{1}H} NMR (101 MHz, $CDCl_3$): δ = 171.8, 136.9, 135.9, 132.7, 132.0, 129.3, 122.8, 61.4, 55.1, 39.2, 20.8, 19.2, 14.0.
HRMS-ESI (ESI+): calculated for $C_{16}H_{21}BrO_4Na^+$ $[M+Na]^+$ 379.0515, found 379.0513.

2-(2-bromo-5-methylbenzyl)-2-methylmalonic acid (S8)[8]

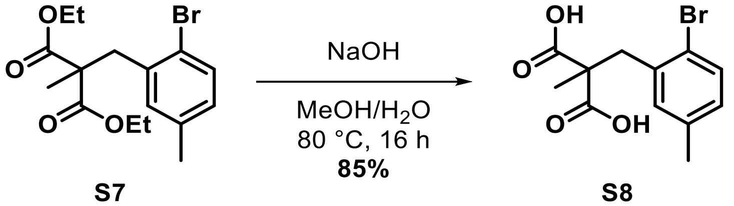

S7 **S8**

A round bottom flask equipped with a magnetic stirring egg and reflux condenser was charged with **S7** (4.41 g, 12.4 mmol, 1.0 eq.) and NaOH (9.86 g, 247 mmol, 20.0 eq.) dissolved in a mixture of $MeOH/H_2O$ (1:1, 1M, 250 mL) and the mixture heated at 80 ºC and stirred overnight. The mixture was allowed to cool down to room temperature, the pH adjusted to 2, followed by extraction with EtOAc (2x) and the combined organic layers washed with brine and dried over $MgSO_4$. The solvent was removed under reduced pressure and the obtained solid washed with *n*-pentane and CH_2Cl_2, yielding **S8** as colorless solid (3.16 g, 10.5 mmol, 85%).

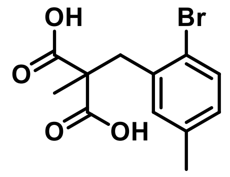

S8
$C_{12}H_{13}BrO_4$
Mw = 301.14 g/mol

^{1}H NMR (400 MHz, $(CD_3)_2SO$): δ = 7.46 (d, *J* = 8.1 Hz, 1H), 7.05 (d, *J* = 2.2 Hz, 1H), 6.98 (dd, *J* = 8.2, 2.2 Hz, 1H), 3.29 (s, 2H), 2.20 (s, 3H), 1.16 (s, 3H).
^{13}C{^{1}H} NMR (101 MHz, $(CD_3)_2SO$): δ = 173.2, 137.0, 136.1, 132.5, 131.8, 129.6, 122.4, 54.3, 38.7, 20.6, 18.8.
HRMS-ESI (ESI+): calculated for $C_{12}H_{13}BrO_4Na^+$ $[M+Na]^+$ 322.9889, found 322.9889.

3-(2-bromo-5-methylphenyl)-2-methylpropanoic acid (S9)[8]

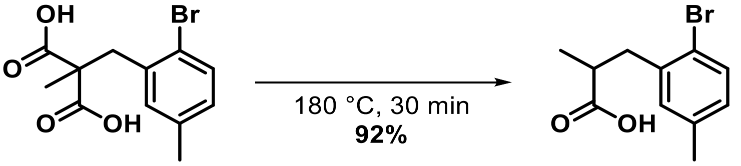

S8 **S9**

An oven-dried crimp top vial equipped with a magnetic stirring bar was charged with **S8** (480 mg, 1.59 mmol, 1.0 eq.) and the vial heated at 180 ºC for 30 min. The vial was allowed to cool down to room temperature, yielding **S9** as a slight brown oil (378 mg, 1.47 mmol, 92%).

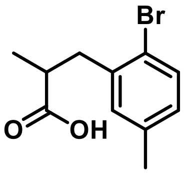

S9
$C_{11}H_{13}BrO_2$
Mw = 257.13 g/mol

^{1}H NMR (400 MHz, $CDCl_3$): δ = 9.54 (br s, 1H), 7.41 (d, *J* = 8.1 Hz, 1H), 7.03 (d, *J* = 2.2 Hz, 1H), 6.90 (dd, *J* = 8.2, 2.2 Hz, 1H), 3.15 (dd, *J* = 13.4, 6.7 Hz, 1H), 2.91 (h, *J* = 7.1 Hz, 1H), 2.77 (dd, *J* = 13.5, 7.8 Hz, 1H), 2.28 (s, 3H), 1.22 (d, *J* = 7.0 Hz, 3H).
^{13}C{^{1}H} NMR (101 MHz, $CDCl_3$): δ = 182.5, 138.2, 137.3, 132.8, 132.2, 129.2, 121.5, 39.6, 39.3, 21.0, 16.8.
HRMS-ESI (ESI+): calculated for $C_{11}H_{13}BrO_2Na^+$ $[M+Na]^+$ 278.9991, found 278.9992.

4-bromo-2,7-dimethyl-2,3-dihydro-1*H*-inden-1-one (13)[8]

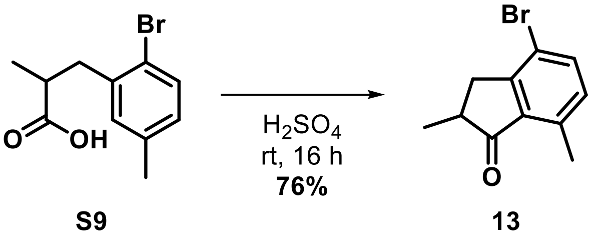

S9 **13**

An oven-dried crimp top vial equipped with a magnetic stirring bar was charged with **S9** (110 mg, 0.43 mmol, 1.0 eq.) and concentrated H_2SO_4 (0.5M, 0.85 mL) and the mixture stirred at room temperature overnight. The mixture was quenched on ice, extracted with EtOAc (3x) and the organic layer washed with saturated aqueous $NaHCO_3$ and brine, dried over $MgSO_4$ and the solvent removed under reduced pressure, yielding **13** as slight brown oil (77.5 mg, 0.32 mmol, 76%).

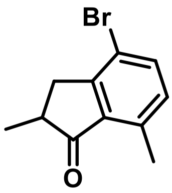

13
$C_{11}H_{11}BrO$
Mw = 239.11 g/mol

^{1}H NMR (400 MHz, $CDCl_3$): δ = 7.58 (d, *J* = 7.9 Hz, 1H), 7.01 (d, *J* = 7.9 Hz, 1H), 3.30 (dd, *J* = 17.5, 8.0 Hz, 1H), 2.76 – 2.61 (m, 2H), 2.59 (s, 3H), 1.32 (d, *J* = 7.4 Hz, 3H).
$^{13}C\{^1H\}$ NMR (101 MHz, $CDCl_3$): δ = 209.8, 153.6, 138.3, 136.7, 135.8, 131.2, 119.0, 42.3, 35.8, 18.0, 16.5.
HRMS-ESI (ESI+): calculated for $C_{11}H_{11}BrOH^+$ $[M+H]^+$ 239.0066, found 239.0065.

5,6-dimethoxy-2,7-dimethyl-2,3-dihydro-1*H*-inden-1-one (14)[9]

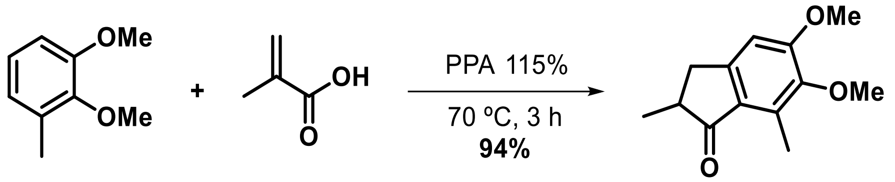

14

A round bottom flask equipped with a mechanical stirrer was charged with PPA 115% (500 mL) and heated to 70 °C. 1,2-Dimethoxy-3-methylbenzene (25.0 g, 164 mmol, 1.0 eq.) was added, the content stirred and after mixing occurred, methacrylic acid (23.7 mL, 24.0 g, 279 mmol, 1.7 eq.) was added and the mixture stirred at 70 °C for 3 h. The mixture was quenched on ice/H_2O, left stirring for 16 h and extracted with EtOAc (3x). The combined organic layers were dried over $MgSO_4$, the solvent removed under reduced pressure and the crude product purified *via* recrystallization (*n*-heptane), yielding **14** as off-white solid (32.1 g, 155 mmol, 94%).

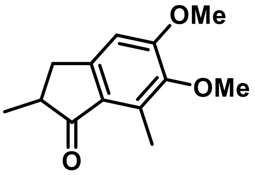

14
$C_{13}H_{16}O_3$
Mw = 220.27 g/mol

^{1}H NMR (400 MHz, $CDCl_3$): δ = 6.74 (s, 1H), 3.93 (s, 3H), 3.76 (s, 3H), 3.25 (dd, *J* = 16.6, 7.5 Hz, 1H), 2.70 – 2.53 (m, 5H), 1.27 (d, *J* = 7.2 Hz, 3H).
$^{13}C\{^1H\}$ NMR (101 MHz, $CDCl_3$): δ = 209.1, 158.2, 152.0, 146.9, 132.2, 127.3, 106.0, 60.5, 55.9, 42.7, 34.6, 16.7, 10.8.
HRMS-ESI (APCI+): calculated for $C_{13}H_{16}O_3H^+$ $[M+H]^+$ 221.1172, found 221.1167.

5,6-dihydroxy-2,7-dimethyl-2,3-dihydro-1H-inden-1-one (15)[9]

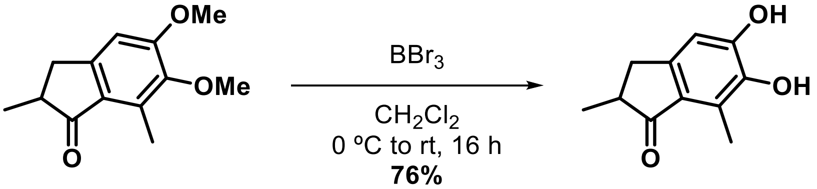

14 **15**

A flame-dried Schlenk tube equipped with a magnetic stirring egg was charged with CH_2Cl_2 (5 mL) and **14** (100 mg, 0.48 mmol, 1.0 eq.) and the mixture cooled down to 0 °C (ice/H_2O). BBr_3 (0.23 mL, 604 mg, 2.41 mmol, 5.0 eq.) was added slowly and the mixture allowed to warm up to room temperature and stirred overnight. The mixture was cooled down to 0 °C (ice/H_2O) again, MeOH slowly added under vigorous stirring until no smoke generation occurred anymore and the mixture stirred for 3 h. The solvent was removed under reduced pressure, the obtained solid washed with H_2O, dissolved in acetone, dried over $MgSO_4$ and the solvent removed under reduced pressure, yielding **15** as dark red solid (65.6 mg, 0.37 mmol, 76%).

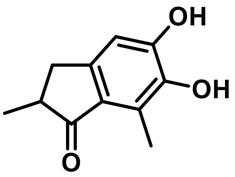

15

$C_{11}H_{12}O_3$

Mw = 192.21 g/mol

^{1}H NMR (400 MHz, $(CD_3)_2SO$): δ = 10.35 (br s, 1H), 8.40 (br s, 1H), 6.68 (s, 1H), 3.11 (dd, *J* = 16.6, 7.7 Hz, 1H), 2.57 – 2.45 (m, 1H), 2.43 (dd, *J* = 16.6, 4.1 Hz, 1H), 2.38 (s, 3H), 1.11 (d, *J* = 7.3 Hz, 3H).
^{13}C{^{1}H} NMR (101 MHz, $(CD_3)_2SO$): δ = 208.0, 151.8, 147.2, 143.0, 125.3, 123.2, 108.9, 41.9, 33.4, 16.6, 10.3.
HRMS-ESI (ESI+): calculated for $C_{11}H_{12}O_3H^+$ $[M+H]^+$ 193.0859, found 193.0857.

Diethyl 2,2'-((2,7-dimethyl-1-oxo-2,3-dihydro-1*H*-indene-5,6-diyl)bis(oxy))(2*R*,2'*R*)-dipropionate (16)[10]

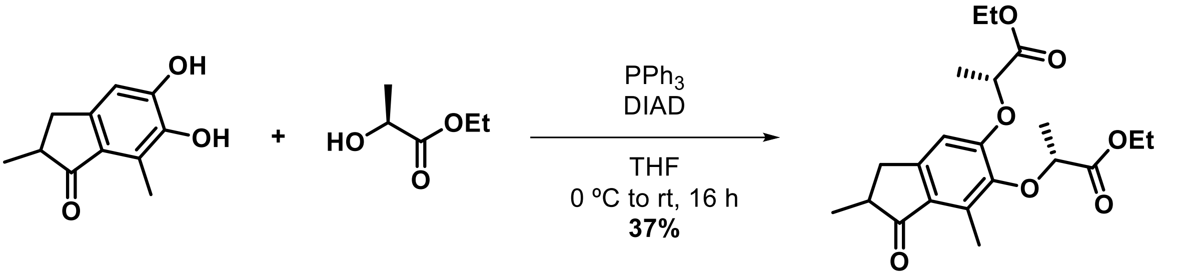

15 **16**

A flame-dried Schlenk tube equipped with a magnetic stirring egg was charged with THF (180 mL), **15** (9.50 g, 53.0 mmol, 1.0 eq.), PPh_3 (69.5 g, 265 mmol, 5.0 eq.) and ethyl (*S*)-2-hydroxypropanoate (30.4 mL, 31.3 g, 265 mmol, 5.0 eq.). DIAD (52.0 mL, 53.6 g, 265 mmol, 5.0 eq.) in THF (180 mL) was added slowly at 0 °C (ice/H_2O) and the mixture allowed to warm up to room temperature and stirred overnight. The solvent was removed under reduced pressure, the material redissolved in a mixture of Et_2O/*n*-pentane (1:2), sonicated and solids filtered off. The solids were washed with Et_2O/*n*-pentane (3x, 1:2), the organic layers combined and the solvents removed under reduced pressure. The crude product was purified *via* flash column chromatography (slow gradient *n*-pentane to 20% EtOAc), yielding **16** as a slight green/yellow oil (7.50 g, 19.8 mmol, 37%).

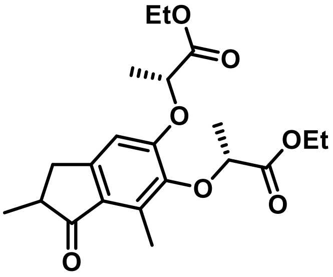

16

$C_{21}H_{28}O_7$

Mw = 392.45 g/mol

^{1}H NMR (400 MHz, $CDCl_3$): δ = 6.52 (s, 1H), 4.83 (q, *J* = 6.8 Hz, 2H), 4.25 – 4.08 (m, 4H), 3.16 (ddd, *J* = 16.8, 7.7, 3.3 Hz, 1H), 2.65 – 2.45 (m, 5H), 1.61 (dd, *J* = 24.4, 6.8 Hz, 6H), 1.22 (t, *J* = 7.0 Hz, 9H).
^{13}C{^{1}H} NMR (101 MHz, $CDCl_3$): δ = 208.9 (d), 172.3 (d), 171.2 (d), 155.0 (d), 151.2 (d), 145.1 (d), 133.1 (d), 128.0 (d), 106.8 (d), 77.1 (d), 72.7, 61.6, 61.0, 42.7, 34.4, 18.8, 18.4, 16.6, 14.2 (d), 11.5.
HRMS-ESI (ESI+): calculated for $C_{21}H_{28}O_7Na^+$ $[M+Na]^+$ 415.1727, found 415.1720.

6-Membered Top Half Ketones

2-methyl-3-(naphthalen-2-ylthio)propanoic acid (S10)[2]

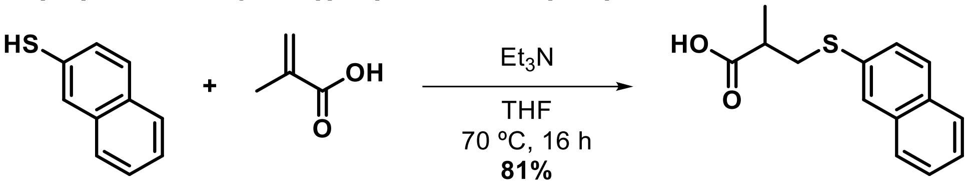

S10

A flame-dried Schlenk tube equipped with a magnetic stirring egg was charged with naphthalene-2-thiol (2.36 g, 14.7 mmol, 1.0 eq.), THF (28 mL), Et_3N (5.8 mL, 4.25 g, 42.0 mmol, 3.0 eq.) and methacrylic acid (2.4 mL, 2.41 g, 28.0 mmol, 2.0 eq.). The mixture was heated to 70 °C and stirred overnight under an inert atmosphere. The mixture was allowed to cool down to room temperature, the solvent removed under reduced pressure and aqueous 1M HCl added and the mixture extracted with EtOAc (3x). The combined organic layers were dried over $MgSO_4$ and the solvent removed under reduced pressure. Subsequently, 10% Et_2O in *n*-pentane was added, the mixture sonicated and stored at 4 °C for 2 h. The solid was filtered off and washed with *n*-pentane, yielding **S10** as a colorless solid (2.81 g, 11.4 mmol, 81%).

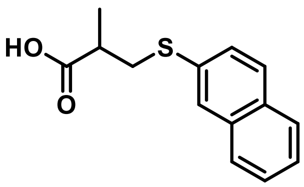

S10

$C_{14}H_{14}O_2S$

Mw = 246.32 g/mol

^{1}H NMR (400 MHz, $CDCl_3$): δ = 7.85 – 7.74 (m, 4H), 7.52 – 7.42 (m, 3H), 3.40 (dd, *J* = 13.5, 6.9 Hz, 1H), 3.03 (dd, *J* = 13.4, 7.1 Hz, 1H), 2.77 (h, *J* = 7.0 Hz, 1H), 1.34 (d, *J* = 7.0 Hz, 3H).
^{13}C{^{1}H} NMR (101 MHz, $CDCl_3$): δ = 181.4, 133.8, 132.9, 132.1, 128.7, 128.4, 128.0, 127.8, 127.3, 126.7, 126.1, 39.7, 37.0, 16.7.
HRMS-ESI (ESI+): calculated for $C_{14}H_{14}O_2SNa^+$ $[M+Na]^+$ 269.0607, found 269.0606.

2-methyl-2,3-dihydro-1*H*-benzo[*f*]thiochromen-1-one (17)

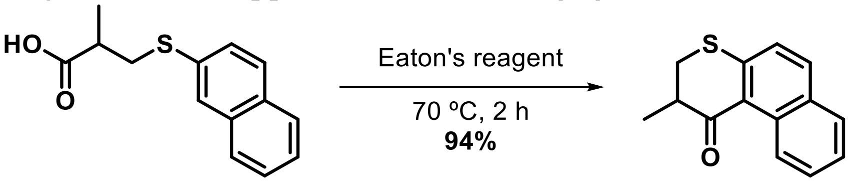

S10 **17**

A round bottom flask equipped with a magnetic stirring egg was charged with **S10** (2.75 g, 11.1 mmol, 1.0 eq.) and Eaton's reagent (12 mL) and the mixture heated at 70 °C and stirred for 2 h. The mixture was allowed to cool down to room temperature, poured on ice and extracted with EtOAc (3x), the combined organic layers washed with saturated aqueous $NaHCO_3$ and brine. The solvent was removed under reduced pressure and the crude product purified *via* distillation (Kugelrohr, 1 mbar, 220 – 230 °C), yielding **17** as a yellow oil (2.39 g, 10.5 mmol, 94%).

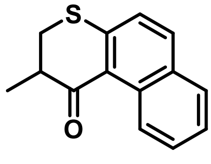

17

$C_{14}H_{12}OS$

Mw = 228.31 g/mol

^{1}H NMR (400 MHz, $CDCl_3$): δ = 9.07 (d, *J* = 8.8 Hz, 1H), 7.75 (t, *J* = 9.0 Hz, 2H), 7.58 (t, *J* = 7.8 Hz, 1H), 7.44 (t, *J* = 7.5 Hz, 1H), 7.24 (d, *J* = 8.7 Hz, 1H), 3.30 – 3.04 (m, 3H), 1.40 (d, *J* = 6.5 Hz, 3H).
^{13}C{^{1}H} NMR (101 MHz, $CDCl_3$): δ = 199.4, 144.1, 133.4, 132.6, 131.8, 129.1, 128.6, 125.8, 125.7, 125.5, 125.2, 43.0, 33.0, 15.5.
HRMS-ESI (ESI+): calculated for $C_{14}H_{12}OSH^+$ $[M+H]^+$ 229.0682, found 229.0682.

3-((3-methoxynaphthalen-2-yl)thio)-2-methylpropanoic acid (S11)[11]

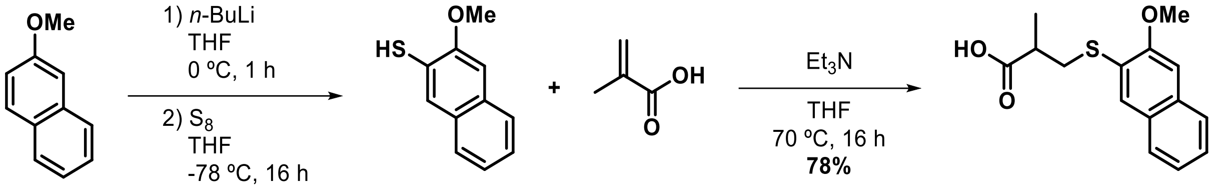

S11

A flame-dried 3-neck round bottom flask equipped with a magnetic stirring egg was charged with THF (500 mL) and 2-methoxynaphthalene (24.0 g, 152 mmol, 1.0 eq.) and cooled down to 0 °C (ice/H_2O). *n*-BuLi (1.6M in hexanes, 123 mL, 12.6 g, 197 mmol, 1.3 eq.) was slowly added and the mixture stirred at 0 °C for 1 h. The mixture was then cooled down to -78 °C (dry ice/EtOH) and S_8 (5.84 g, 22.8 mmol, 0.15 eq.) was slowly added and stirred for 30 min at -78 °C. The mixture was allowed to warm up to room temperature overnight, aqueous 1M HCl was added and the mixture extracted with Et_2O (2x), the combined organic layers washed with brine, dried over $MgSO_4$ and the solvent removed under reduced pressure.

A flame-dried Schlenk tube equipped with a magnetic stirring egg was charged with the crude product, THF (500 mL), Et_3N (63.3 mL, 46.1 g, 455 mmol, 3.0 eq.) and methacrylic acid (25.7 mL, 26.1 g, 303 mmol, 2.0 eq.). The mixture was heated to 70 °C and stirred overnight under an inert atmosphere. The mixture was allowed to cool down to room temperature, the solvent removed under reduced pressure and aqueous 1M HCl added and the mixture extracted with EtOAc (3x). The combined organic layers were washed with brine and the solvent removed under reduced pressure. Subsequently, 10% Et_2O in *n*-pentane was added and sonicated, the filtered solid was washed with *n*-pentane, yielding **S11** as a grey solid (32.8 g, 119 mmol, 78% over two steps).

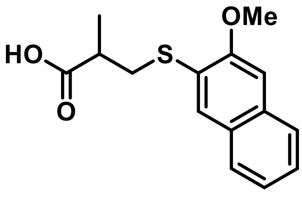

S11
$C_{15}H_{16}O_3S$
Mw = 276.35 g/mol

^{1}H NMR (400 MHz, $CDCl_3$): δ = 7.71 (d, *J* = 8.2 Hz, 3H), 7.40 (t, *J* = 7.5 Hz, 1H), 7.34 (t, *J* = 7.6 Hz, 1H), 7.11 (s, 1H), 4.01 (s, 3H), 3.39 (dd, J = 13.1, 6.5 Hz, 1H), 2.99 (dd, *J* = 13.1, 7.3 Hz, 1H), 2.79 (h, *J* = 7.0 Hz, 1H), 1.36 (d, *J* = 7.0 Hz, 3H).
^{13}C{^{1}H} NMR (101 MHz, $CDCl_3$): δ = 180.5, 155.6, 133.3, 129.1, 128.6, 127.0, 126.6, 126.3, 126.0, 124.3, 105.6, 56.1, 39.3, 35.1, 16.9.
HRMS-ESI (ESI+): calculated for $C_{15}H_{16}O_3SNa^+$ $[M+Na]^+$ 299.0712, found 299.0714.

5-methoxy-2-methyl-2,3-dihydro-1*H*-benzo[*f*]thiochromen-1-one (18)

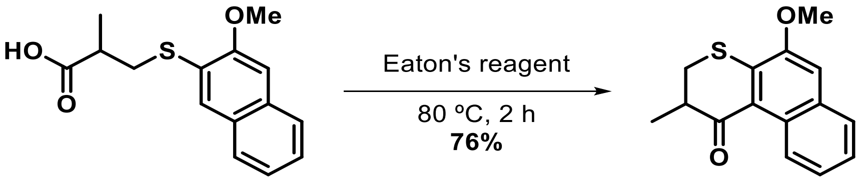

S11 **18**

A round bottom flask equipped with a magnetic stirring egg was charged with **S11** (22.0 g, 79.6 mmol, 1.0 eq.) and Eaton's reagent (80 mL) and the mixture heated to 80 °C and stirred for 2 h. The mixture was allowed to cool down to room temperature, poured on ice and extracted with EtOAc (3x), the combined organic layers washed with aqueous 1M NaOH (2x) and brine. The solvent was removed under reduced pressure and the crude product purified *via* distillation (Kugelrohr, 1 mbar, 250 °C), yielding **18** as a yellow solid (15.7 g, 60.9 mmol, 76%).

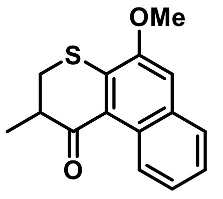

18
$C_{15}H_{14}O_2S$
Mw = 258.34 g/mol

^{1}H NMR (400 MHz, $CDCl_3$): δ = 8.96 (d, *J* = 7.7 Hz, 1H), 7.67 (d, *J* = 7.6 Hz, 1H), 7.43 (p, *J* = 7.0 Hz, 2H), 7.18 (s, 1H), 4.02 (s, 3H), 3.30 – 3.01 (m, 3H), 1.38 (d, *J* = 6.2 Hz, 3H).
^{13}C{^{1}H} NMR (101 MHz, $CDCl_3$): δ = 199.6, 152.4, 137.8, 132.0, 127.9, 127.2, 126.9, 126.5, 126.0, 125.6, 109.7, 56.3, 42.3, 31.9, 15.2.
HRMS-ESI (ESI+): calculated for $C_{15}H_{14}O_2SH^+$ $[M+H]^+$ 259.0787, found 259.0786.

5-hydroxy-2-methyl-2,3-dihydro-1*H*-benzo[*f*]thiochromen-1-one (19)[11]

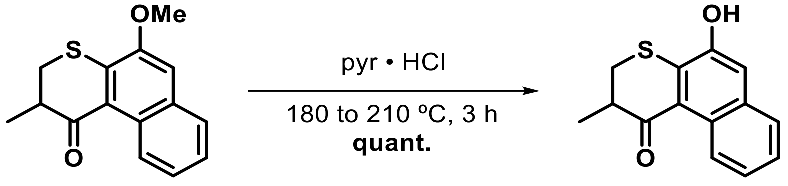

18 **19**

A round bottom flask equipped with a magnetic stirring egg was charged with pyridine HCl (76.5 g, 662 mmol, 30.0 eq.) and heated to 180 °C. **18** (5.70 g, 22.1 mmol, 1.0 eq.) was added and the resulting mixture heated at 210 °C for 3 h. The mixture was poured onto ice/H_2O, extracted with warm EtOAc (2x), the combined organic layers were washed with H_2O and brine and the solvent removed under reduced pressure, yielding **19** as a green solid (5.37 g, 21.9 mmol, quant.).

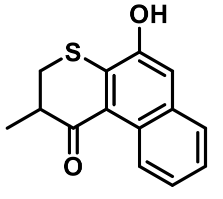

19
$C_{14}H_{12}O_2S$
Mw = 244.31 g/mol

^{1}H NMR (400 MHz, $CDCl_3$): δ = 8.91 (d, *J* = 8.5 Hz, 1H), 7.62 (d, *J* = 7.8 Hz, 1H), 7.49 – 7.36 (m, 2H), 7.31 (s, 1H), 5.62 (br s, 1H), 3.28 (t, *J* = 9.2 Hz, 1H), 3.21 – 3.06 (m, 2H), 1.42 (d, *J* = 6.2 Hz, 3H).
^{13}C{^{1}H} NMR (101 MHz, $CDCl_3$): δ = 199.5, 148.7, 135.5, 132.3, 128.2, 127.6, 127.0, 126.7, 126.2, 125.7, 114.9, 43.3, 32.7, 15.6.
HRMS-ESI (ESI-): calculated for $C_{14}H_{11}O_2S^-$ $[M-H]^-$ 243.0485, found 243.0484.

5-Membered Bottom Half Ketones

3,6-dibromophenanthrene-9,10-dione (S12)[12]

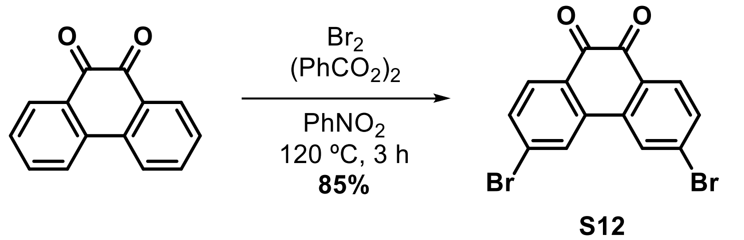

S12

A round bottom flask equipped with a magnetic stirring egg, reflux condenser and wash bottle (aqueous $Na_2S_2O_3$) was charged with nitrobenzene (44 mL), phenanthrene-9,10-dione (10.1 g, 48.4 mmol, 1.0 eq.), benzoyl peroxide (358 mg, 1.48 mmol, 3 mol%) and bromine (0.8 mL, 2.52 g, 15.7 mmol, 0.3 eq.). The mixture was heated to 120 °C and bromine (4.8 mL, 15.1 g, 94.4 mmol, 2.0 eq.) added. The mixture was stirred at 120 °C for 3 h, allowed to cool down to room temperature, filtered over a Büchner funnel and washed with EtOH, yielding **S12** as a gold/brown solid (15.1 g, 41.2 mmol, 85%).

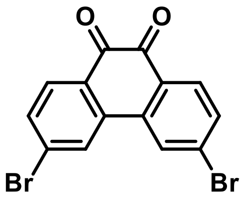

S12
$C_{14}H_6Br_2O_2$
Mw = 366.01 g/mol

^{1}H NMR (400 MHz, $CDCl_3$): δ = 8.11 (d, *J* = 1.7 Hz, 2H), 8.06 (d, *J* = 8.3 Hz, 2H), 7.66 (dd, *J* = 8.3, 1.7 Hz, 2H).
^{13}C{^{1}H} NMR (101 MHz, $CDCl_3$): δ = 179.0, 136.1, 133.6, 132.3, 132.2, 130.0, 127.1.
HRMS-ESI (ESI+): calculated for $C_{14}H_6Br_2O_2Na^+$ $[M+Na]^+$ 386.8627, found 386.8624.

3,6-dibromo-9*H*-fluoren-9-one (20)[12]

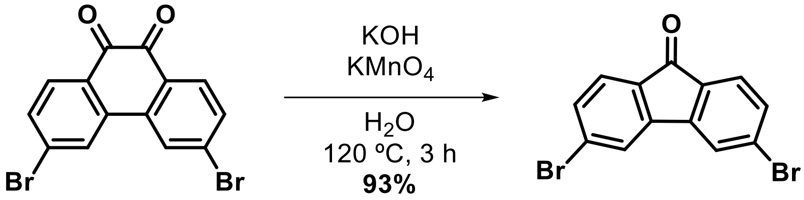

S12 **20**

A round bottom flask (25x the volume of H_2O used) equipped with a magnetic stirring egg and reflux condenser, was charge with KOH (4.98 g, 87.2 mmol, 13.0 eq.), H_2O (15 mL) and heated to 130 ºC. Subsequently, **S12** (2.50 g, 6.83 mmol, 1.0 eq.) and $KMnO_4$ (5.72 g, 36.2 mmol, 5.3 eq.) were added portionwise (harsh reaction due to CO_2 generation) and the mixture stirred for 3 h at 130 ºC. The mixture was allowed to cool down to room temperature and was slowly neutralized with concentrated H_2SO_4 and Na_2SO_3 (other reducing agents like $S_2O_3^{2-}$ did not work as good) added to the slightly acidic mixture. The precipitate was filtered off, washed with H_2O and dried at 60 ºC *in vacuo*, yielding **20** as a pale yellow solid (2.15 g, 6.37 mmol, 93%).

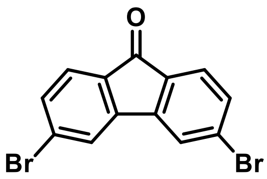

20
$C_{13}H_6Br_2O$
Mw = 338.00 g/mol

^{1}H NMR (400 MHz, $CDCl_3$): δ = 7.68 (d, *J* = 1.6 Hz, 2H), 7.55 (d, *J* = 7.9 Hz, 2H), 7.50 (dd, *J* = 7.9, 1.6 Hz, 2H).
^{13}C{^{1}H} NMR (101 MHz, $CDCl_3$): δ = 191.5, 144.9, 133.0, 132.9, 130.0, 125.9, 124.3.

Methyl 4'-methoxy-[1,1'-biphenyl]-2-carboxylate (S13)[13]

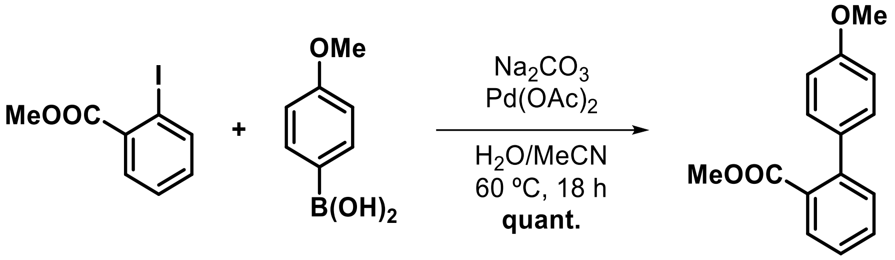

S13

An oven-dried crimp top vial equipped with a magnetic stirring bar was charged with (4-methoxyphenyl)boronic acid (1.26 g, 8.30 mmol, 1.1 eq.), Na_2CO_3 (2.80 g, 26.4 mmol, 3.5 eq.) and $Pd(OAc)_2$ (21.1 mg, 94.0 µmol, 1 mol%). The vial was crimped, flushed with nitrogen and MeCN (16 mL), H_2O (6.5 mL) and methyl 2-iodobenzoate (1.1 mL, 1.96 g, 7.49 mmol, 1.0 eq.) were added. The mixture was heated at 60 °C for 18 h. Subsequently, the mixture was allowed to cool down to room temperature, H_2O added and extracted with CH_2Cl_2 (2x). The combined organic layers were dried over $MgSO_4$, the solvent removed under reduced pressure, yielding **S13** colorless oil (1.81 g, 7.46 mmol, quant.).

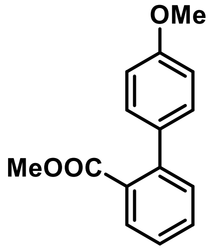

S13
$C_{15}H_{14}O_3$
Mw = 242.27 g/mol

^{1}H NMR (600 MHz, $CDCl_3$): δ = 7.79 (dd, *J* = 7.7, 1.4 Hz, 1H), 7.51 (td, *J* = 7.5, 1.4 Hz, 1H), 7.40 – 7.34 (m, 2H), 7.26 – 7.23 (m, 2H), 6.95 – 6.92 (m, 2H), 3.85 (s, 3H), 3.67 (s, 3H).
^{13}C{^{1}H} NMR (151 MHz, $CDCl_3$): δ = 169.6, 159.1, 142.2, 133.8, 131.3, 131.0, 130.9, 129.9, 129.6, 126.9, 113.7, 55.4, 52.1.
HRMS-ESI (APCI+): calculated for $C_{15}H_{14}O_3H^+$ $[M+H]^+$ 243.1016, found 243.1015.

2-methoxy-9*H*-fluoren-9-one (21)[14]

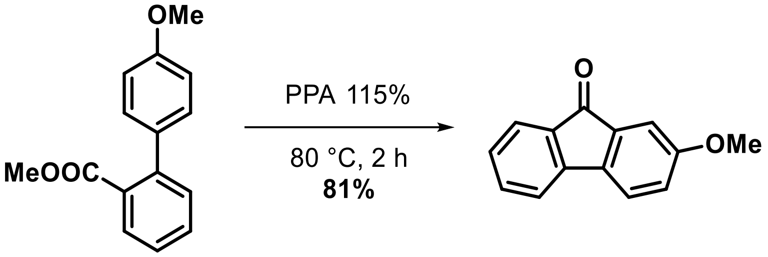

S13 **21**

A round bottom flask equipped with a mechanical stirrer was charged with **S13** (2.04 g, 8.42 mmol, 1.0 eq.) and PPA 115% (40 mL). The mixture was heated to 80 °C and stirred for 2 h. The mixture was quenched with ice and EtOAc and left stirring overnight at room temperature. The layers were separated and the aqueous phase extracted with EtOAc (2x). The combined organic layers were washed with aqueous $NaHCO_3$, brine, dried over $MgSO_4$ and the solvent removed under reduced pressure. The crude product was purified *via* filtration over a silica plug (*n*-pentane/CH_2Cl_2 3:7), yielding **21** as yellow solid (1.43 g, 6.80 mmol, 81%).

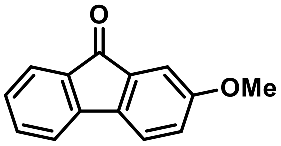

21
$C_{14}H_{10}O_2$
Mw = 210.23 g/mol

^{1}H NMR (600 MHz, $CDCl_3$): δ = 7.60 (dt, *J* = 7.3, 0.9 Hz, 1H), 7.45 – 7.42 (m, 1H), 7.43 – 7.38 (m, 2H), 7.21 – 7.18 (m, 2H), 6.99 (dd, *J* = 8.1, 2.5 Hz, 1H), 3.86 (s, 3H).
$^{13}C\{^1H\}$ NMR (151 MHz, $CDCl_3$): δ = 194.0, 161.0, 145.1, 137.2, 136.1, 135.0, 134.5, 128.0, 124.5, 121.5, 120.5, 119.7, 109.5, 55.9.
HRMS-ESI (ESI+): calculated for $C_{14}H_{10}O_2^+$ $[M+]^+$ 210.0675, found 210.0665.

2-hydroxy-9*H*-fluoren-9-one (22)

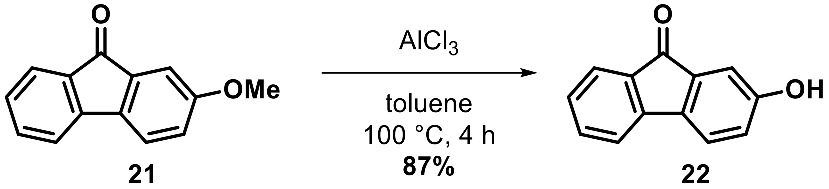

A flame-dried Schlenk tube equipped with a magnetic stirring egg was charged with toluene (100 mL) and **21** (1.40 g, 6.66 mmol, 1.0 eq.). $AlCl_3$ (2.66 g, 20.0 mmol, 3.0 eq.) was added slowly and the mixture heated to 100 °C and stirred for 4 h. The mixture was allowed to cool down to room temperature, poured on ice and extracted with EtOAc (3x). The combined organic layers were washed with brine, dried over $MgSO_4$, the solvent removed under reduced pressure and the compound triturated with *n*-pentane, yielding **22** as red solid (1.13 g, 5.76 mmol, 87%).

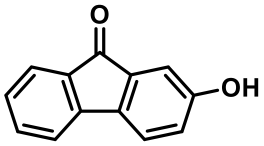

22
$C_{13}H_8O_2$
Mw = 196.21 g/mol

^{1}H NMR (400 MHz, $(CD_3)_2SO$): δ = 10.05 (s, 1H), 7.56 – 7.42 (m, 4H), 7.19 (td, *J* = 7.4, 1.1 Hz, 1H), 6.96 (d, *J* = 2.3 Hz, 1H), 6.92 (dd, *J* = 8.0, 2.4 Hz, 1H).
$^{13}C\{^1H\}$ NMR (101 MHz, $(CD_3)_2SO$): δ = 193.3, 158.9, 144.8, 135.3, 135.2, 134.7, 133.4, 127.7, 123.8, 122.3, 121.0, 119.9, 111.0.
HRMS-ESI (ESI-): calculated for $C_{13}H_7O_2^-$ $[M-H]^-$ 195.0452, found 195.0451.

Methyl 2-iodo-4-methoxybenzoate (S14)[15]

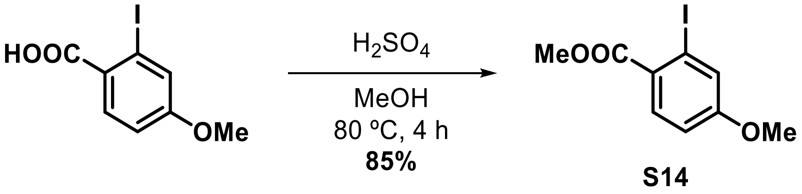

An oven-dried crimp top vial equipped with a magnetic stirring bar was charged with 2-iodo-4-methoxybenzoic acid (2.49 g, 8.97 mmol, 1.0 eq.). The vial was crimped, flushed with nitrogen and MeOH (24 mL) and concentrated H_2SO_4 (2.4 mL) were added. The mixture was heated at 80 °C for 4 h. The mixture was allowed to cool down to room temperature, the solvent removed under reduced pressure, the residue was diluted with Et_2O and extracted with H_2O (2x), aqueous $Na_2S_2O_3$, aqueous $NaHCO_3$ and dried over Na_2SO_4. The solvent was removed under reduced pressure and the crude product purified *via* flash column chromatography (gradient *n*-pentane to EtOAc), yielding **S14** as colorless oil (2.23 g, 7.65 mmol, 85%).

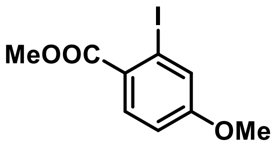

S14
$C_9H_9IO_3$
Mw = 292.07 g/mol

^{1}H NMR (400 MHz, $CDCl_3$): δ = 7.86 (d, *J* = 8.8 Hz, 1H), 7.53 (d, *J* = 2.6 Hz, 1H), 6.91 (dd, *J* = 8.8, 2.6 Hz, 1H), 3.89 (s, 3H), 3.83 (s, 3H).
^{13}C{^{1}H} NMR (101 MHz, $CDCl_3$): δ = 166.0, 162.0, 132.6, 127.0, 126.1, 113.7, 95.7, 55.6, 52.2.
HRMS-ESI (ESI+): calculated for $C_9H_9IO_3Na^+$ $[M+Na]^+$ 314.9489, found 314.9487.

Methyl 3',5-dimethoxy-[1,1'-biphenyl]-2-carboxylate (S15)

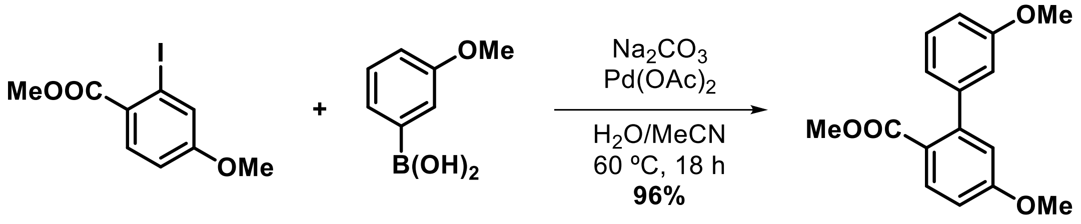

S14 **S15**

An oven-dried crimp top vial equipped with a magnetic stirring bar was charged with **S14** (1.00 g, 3.45 mmol, 1.0 eq.), (3-methoxyphenyl)boronic acid (569 mg, 3.74 mmol, 1.1 eq.), Na_2CO_3 (1.35 g, 12.8 mmol, 3.7 eq.) and $Pd(OAc)_2$ (12.9 mg, 57.5 μmol, 2 mol%). The vial was crimped, flushed with nitrogen and MeCN (8 mL) and H_2O (3 mL) were added. The mixture was heated at 60 °C for 18 h. The mixture was allowed to cool down to room temperature, H_2O added and extracted with CH_2Cl_2 (2x). The combined organic layers were dried over Na_2SO_4, the solvent removed under reduced pressure and the crude product purified *via* flash column chromatography (gradient *n*-pentane to EtOAc), yielding **S15** as a colorless oil (904 mg, 3.32 mmol, 96%).

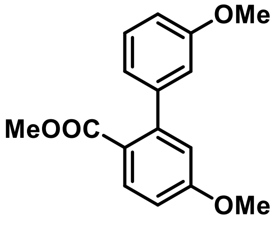

S15
$C_{16}H_{16}O_4$
Mw = 272.30 g/mol

^{1}H NMR (400 MHz, $CDCl_3$): δ = 7.86 (d, *J* = 8.6 Hz, 1H), 7.30 (t, *J* = 7.9 Hz, 1H), 6.95 – 6.82 (m, 5H), 3.86 (s, 3H), 3.83 (s, 3H), 3.63 (s, 3H).
^{13}C{^{1}H} NMR (101 MHz, $CDCl_3$): δ = 168.3, 161.8, 159.3, 145.3, 143.2, 132.4, 129.0, 122.8, 121.0, 116.2, 114.0, 112.9, 112.8, 55.6, 55.4, 51.9.
HRMS-ESI (ESI+): calculated for $C_{16}H_{16}O_4Na^+$ $[M+Na]^+$ 295.0941, found 295.0939.

3,6-dimethoxy-9*H*-fluoren-9-one (23)

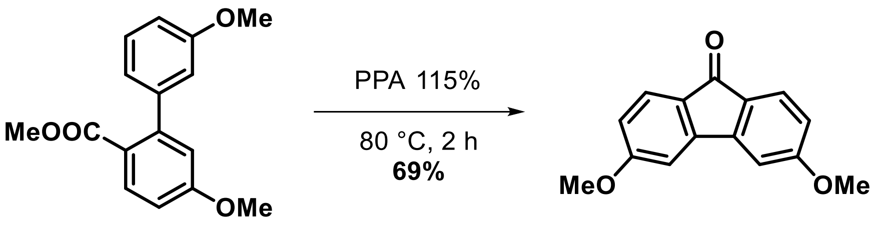

S15 **23**

A round bottom flask equipped with a mechanical stirrer was charged with **S15** (2.18 g, 8.02 mmol, 1.0 eq.) and PPA 115% (20 mL). The mixture was heated to 80 ºC and stirred for 2 h. The mixture was quenched with ice and EtOAc and left stirring overnight at room temperature. The layers were separated and the aqueous phase extracted with EtOAc (2x). The combined organic layers were washed with aqueous $NaHCO_3$, brine, dried over $MgSO_4$ and the solvent removed under reduced pressure. The crude product was purified *via* flash column chromatography (gradient CH_2Cl_2 to 4% MeOH in CH_2Cl_2), yielding **23** as yellow solid (1.33 g, 5.52 mmol, 69%).

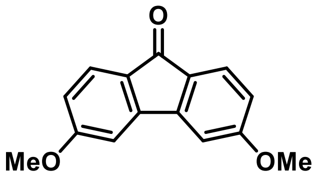

23
$C_{15}H_{12}O_3$
Mw = 240.26 g/mol

^{1}H NMR (400 MHz, $CDCl_3$): δ = 7.55 (d, *J* = 8.2 Hz, 2H), 6.95 (d, *J* = 1.9 Hz, 2H), 6.71 (dd, *J* = 8.2, 1.4 Hz, 2H), 3.88 (s, 6H).
^{13}C{^{1}H} NMR (101 MHz, $CDCl_3$): δ = 191.5, 165.1, 146.0, 128.4, 125.8, 113.1, 107.1, 55.8.
HRMS-ESI (ESI+): calculated for $C_{15}H_{12}O_3Na^+$ $[M+Na]^+$ 263.0679, found 263.0676.

3,6-dihydroxy-9*H*-fluoren-9-one (24)

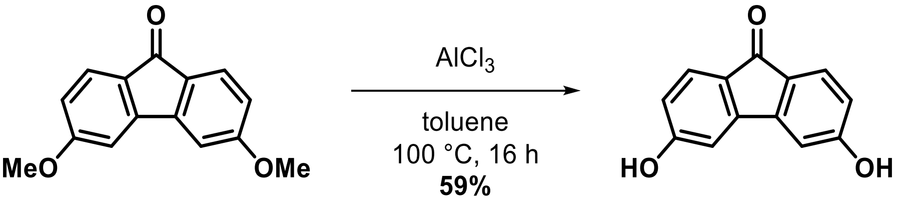

23 **24**

A flame-dried Schlenk tube equipped with a magnetic stirring egg was charged with toluene (100 mL) and **21** (1.14 g, 4.75 mmol, 1.0 eq.). $AlCl_3$ (6.33 g, 47.5 mmol, 10.0 eq.) was added slowly and the mixture heated to 100 ºC and stirred for 16 h. The mixture was allowed to cool down to room temperature, poured on ice and extracted with EtOAc (3x). The combined organic layers were washed with brine, dried over $MgSO_4$, the solvent removed under reduced pressure and the crude compound purified *via* flash column chromatography (gradient *n*-pentane/EtOAc 5:1 to EtOAc) and subsequent recrystallization (acetone) yielding **24** as brown solid (594 mg, 2.80 mmol, 59%).

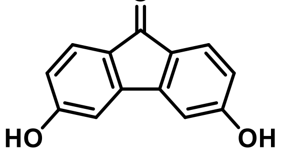

24
$C_{13}H_8O_3$
Mw = 212.20 g/mol

^{1}H NMR (400 MHz, $(CD_3)_2SO$): δ = 10.53 (br s, 2H), 7.38 (d, *J* = 8.0 Hz, 2H), 7.05 (s, 2H), 6.67 (d, *J* = 8.0 Hz, 2H).
^{13}C{^{1}H} NMR (101 MHz, $(CD_3)_2SO$): δ = 190.3, 163.7, 145.8, 126.0, 125.5, 115.0, 108.5.
HRMS-ESI (ESI-): calculated for $C_{13}H_7O_3^-$ $[M-H]^-$ 211.0401, found 211.0402.

6-Membered Bottom Half Ketones

Dimethyl 3,3'-(10-oxo-9,10-dihydroanthracene-9,9-diyl)dipropionate (25)[16]

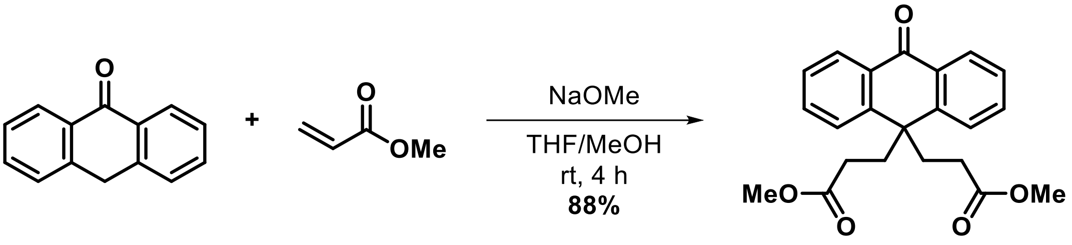

25

A round bottom flask equipped with a magnetic stirring egg was charged with anthrone (5.00 g, 25.7 mmol, 1.0 eq.), methyl acrylate (10.3 mL, 9.74 g, 113 mmol, 4.4 eq.), NaOMe (141 mg, 2.61 mmol, 0.1 eq.), THF (50 mL) and MeOH (25 mL). The resulting mixture was stirred at room temperature for 4 h, quenched with aqueous 0.1M HCl and extracted with EtOAc. The solvents were removed under reduced pressure and the crude product purified *via* flash column chromatography (gradient *n*-pentane to EtOAc), yielding **25** as a pale yellow solid (8.31 g, 22.7 mmol, 88%).

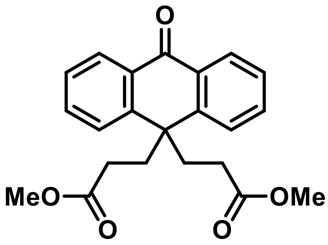

25
$C_{22}H_{22}O_5$
Mw = 366.41 g/mol

^{1}H NMR (600 MHz, $CDCl_3$): δ = 8.38 (dd, *J* = 7.9, 1.5 Hz, 2H), 7.71 – 7.67 (m, 2H), 7.64 (d, *J* = 8.0 Hz, 2H), 7.50 – 7.44 (m, 2H), 3.43 (s, 6H), 2.62 – 2.58 (m, 4H), 1.59 – 1.55 (m, 4H).
^{13}C{^{1}H} NMR (151 MHz, $CDCl_3$): δ = 183.2, 173.3, 145.0, 134.4, 132.8, 127.8, 127.6, 126.0, 51.6, 44.9, 39.9, 29.2.
HRMS-ESI (ESI+): calculated for $C_{22}H_{22}O_5Na^+$ $[M+Na]^+$ 389.1260, found 389.1351.

1,2-bis(2-methoxyphenyl)disulfane (S16)[11]

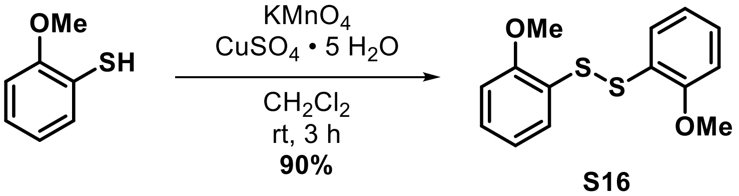

S16

A round bottom flask equipped with a mechanical stirrer was charged with 2-methoxybenzenethiol (60.0 g, 428 mmol, 1.0 eq.), potassium permanganate (156 g, 984 mmol, 2.3 eq.), copper(II) sulfate pentahydrate (150 g, 599 mmol, 1.4 eq.) and CH_2Cl_2 (1 L). The resulting mixture was stirred at room temperature for 3 h, filtered over celite and the solvent removed under reduced pressure. The crude product was triturated with MeOH, yielding **S16** as off-white solid (53.8 g, 193 mmol, 90%).

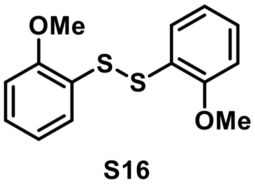

S16
$C_{14}H_{14}O_2S_2$
Mw = 278.38 g/mol

^{1}H NMR (400 MHz, $CDCl_3$): δ = 7.54 (d, *J* = 7.8 Hz, 2H), 7.19 (t, *J* = 7.7 Hz, 2H), 6.92 (t, *J* = 7.6 Hz, 2H), 6.86 (d, *J* = 8.2 Hz, 2H), 3.90 (s, 6H).
^{13}C{^{1}H} NMR (101 MHz, $CDCl_3$): δ = 156.7, 127.9, 127.7, 124.7, 121.5, 110.6, 56.0.
HRMS-ESI (ESI+): calculated for $C_{14}H_{14}O_2S_2Na^+$ $[M+Na]^+$ 301.0328, found 301.0330.

***N*,*N*-diethyl-3-methoxybenzamide (S17)**[11]

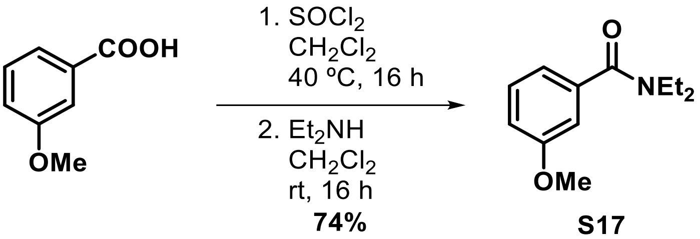

S17

A flame-dried Schlenk tube equipped with a magnetic stirring egg was charged with 3-methoxybenzoic acid (25.0 g, 165 mmol, 1.0 eq.) and CH_2Cl_2 (200 mL). $SOCl_2$ (28.7 mL, 47.0 g, 395 mmol, 2.4 eq.) was slowly added and the mixture stirred at 40 °C overnight. The mixture was allowed to cool down to room temperature, solvent and volatiles removed under reduced pressure and the mixture redissolved in CH_2Cl_2 (200 mL). The mixture was added to a solution of Et_2NH (68.1 mL, 48.2 g, 659 mmol, 4.0 eq.) in CH_2Cl_2 at 0 °C (ice/H_2O), allowed to warm up to room temperature and stirred overnight. The mixture was extracted with aqueous 1M HCl (2x) and aqueous $NaHCO_3$ (2x) and the solvent removed under reduced pressure, yielding **S17** as a yellow oil (25.4 g, 122 mmol, 74%).

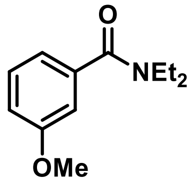

S17
$C_{12}H_{17}NO_2$
Mw = 207.27 g/mol

^{1}H NMR (400 MHz, $CDCl_3$): δ = 7.29 (t, J = 7.8 Hz, 1H), 6.96 – 6.87 (m, 3H), 3.82 (s, 3H), 3.54 (br s, 2H), 3.26 (br s, 2H), 1.24 (br s, 3H), 1.11 (br s, 3H).
^{13}C{^{1}H} NMR (101 MHz, $CDCl_3$): δ = 171.1, 159.7, 138.7, 129.7, 118.5, 115.1, 111.8, 55.4, 43.4, 39.3, 14.4, 13.0.
HRMS-ESI (ESI+): calculated for $C_{12}H_{17}NO_2Na^+$ $[M+Na]^+$ 230.1152, found 230.230.1149.

***N*,*N*-diethyl-3-methoxy-2-((2-methoxyphenyl)thio)benzamide (S18)**[11]

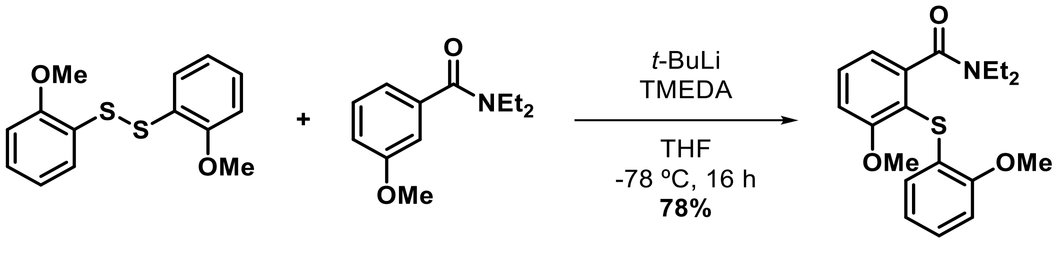

S16 **S17** **S18**

A flame-dried 3-neck round bottom flask equipped with a magnetic stirring egg was charged with THF (950 mL) and cooled down to -78 °C (dry ice/EtOH). *t*-BuLi (1.7M in pentane, 99.6 mL, 10.9 g, 169 mmol, 1.2 eq.) and TMEDA (25.2 mL, 19.7 g, 169 mmol, 1.2 eq.) were slowly and subsequently added and the mixture stirred for 30 min. **S17** (29.0 g, 140 mmol, 1.0 eq.) dissolved in THF (70 mL) was added dropwise and the mixture stirred at -78 °C for 1 h. **S16** (51.4 g, 185 mmol, 1.3 eq.) was added portionwise and the mixture allowed to warm up to room temperature overnight. The mixture was quenched by slow addition of ice-cold H_2O, extracted with Et_2O and the organic layer washed with aqueous 1M NaOH. The solvent was removed under reduced pressure and the crude product purified *via* recrystallization (EtOAc), yielding **S18** as cottonlike yellow solid (37.5 g, 109 mmol, 78%).

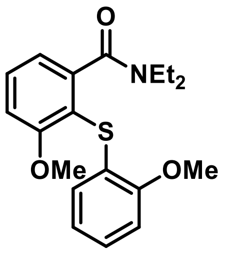

S18
$C_{19}H_{23}NO_3S$
Mw = 345.46 g/mol

^{1}H NMR (400 MHz, $CDCl_3$): δ = 7.43 (t, *J* = 7.9 Hz, 1H), 7.08 – 6.99 (m, 1H), 6.95 (s, 1H), 6.93 (s, 1H), 6.79 (d, *J* = 8.2 Hz, 1H), 6.77 – 6.64 (m, 2H), 3.87 (s, 3H), 3.75 (s, 3H), 3.72 – 3.60 (m, 1H), 3.41 – 3.29 (m, 1H), 3.17 – 3.05 (m, 1H), 3.05 – 2.94 (m, 1H), 1.18 (t, *J* = 7.1 Hz, 3H), 0.98 (t, *J* = 7.1 Hz, 3H).
^{13}C{^{1}H} NMR (101 MHz, $CDCl_3$): δ = 168.8, 160.7, 155.7, 145.3, 131.3, 127.1, 125.9, 125.5, 121.1, 118.8, 116.2, 111.4, 110.3, 56.2, 55.9, 42.8, 38.7, 14.0, 12.6.
HRMS-ESI (ESI+): calculated for $C_{19}H_{23}NO_3SNa^+$ $[M+Na]^+$ 368.1291, found 368.1288.

4,5-dimethoxy-9*H*-thioxanthen-9-one (26)[11]

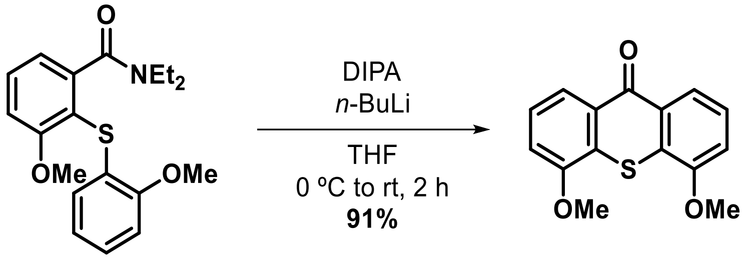

S18 **26**

A flame-dried 3-neck round bottom flask equipped with a magnetic stirring egg was charged with THF (300 mL) and DIPA (25.0 mL, 17.8 g, 177 mmol, 5.5 eq.) and was cooled down to 0 °C (dry ice/H_2O). *n*-BuLi (11M in hexanes, 14.6 mL, 10.3 g, 161 mmol, 5.0 eq.) was slowly added and the mixture stirred for 15 min. **S18** (11.1 g, 32.1 mmol, 1.0 eq.) dissolved in THF (200 mL) was added dropwise. After addition the mixture was allowed to warm up to room temperature and stirred for 2 h. The mixture was quenched by slow addition of aqueous NH_4Cl, extracted with CH_2Cl_2 (2x), dried over $MgSO_4$ and the solvent removed under reduced pressure. The crude product was purified *via* recrystallization (MeOH), yielding **26** as yellow solid (8.00 g, 29.4 mmol, 91%).

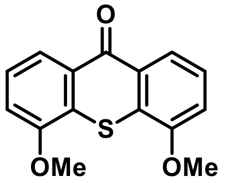

26
$C_{15}H_{12}O_3S$
Mw = 272.32 g/mol

^{1}H NMR (600 MHz, $CDCl_3$): δ = 8.24 (d, *J* = 8.1 Hz, 2H), 7.44 (t, *J* = 8.0 Hz, 2H), 7.13 (d, *J* = 7.9 Hz, 2H), 4.04 (s, 6H).
^{13}C{^{1}H} NMR (151 MHz, $CDCl_3$): δ = 180.4, 155.0, 130.1, 127.8, 126.1, 121.6, 112.2, 56.6.
HRMS-ESI (ESI+): calculated for $C_{15}H_{12}O_3SH^+$ $[M+H]^+$ 273.0580, found 273.0578.

4,5-dihydroxy-9*H*-thioxanthen-9-one (27)[11]

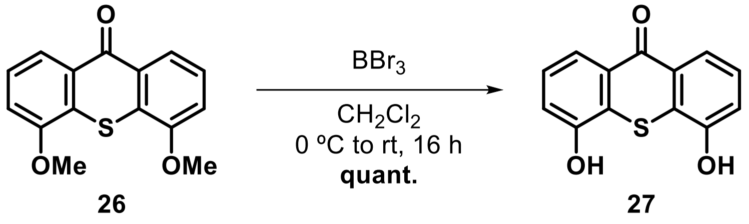

A flame-dried Schlenk tube equipped with a magnetic stirring egg was charged with **26** (2.00 g, 7.34 mmol, 1.0 eq.) and CH_2Cl_2 (100 mL). The mixture was cooled down to 0 °C (ice/H_2O), BBr_3 (3.5 mL, 9.20 g, 36.7 mmol, 5.0 eq.) added dropwise and the mixture allowed to warm up to room temperature overnight. Next, the mixture was cooled down to 0 °C (ice/H_2O) again, MeOH (100 mL) slowly added and the mixture stirred for 3 h. The solvent was removed under reduced pressure, the obtained solid washed with H_2O, dissolved in acetone, the solution dried over $MgSO_4$ and the solvent removed under reduced pressure, yielding **27** as yellow/green solid (1.79 g, 7.32 mmol, quant.).

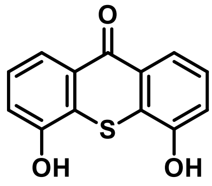

27
$C_{13}H_8O_3S$
Mw = 244.26 g/mol

^{1}H NMR (400 MHz, $(CD_3)_2SO$): δ = 11.00 (s, 2H), 7.95 (dd, *J* = 8.0, 1.3 Hz, 2H), 7.40 (t, *J* = 7.9 Hz, 2H), 7.21 (dd, *J* = 7.8, 1.3 Hz, 2H).
^{13}C{^{1}H} NMR (101 MHz, $(CD_3)_2SO$): δ = 179.4, 153.1, 129.3, 126.4, 125.3, 119.2, 116.5.
HRMS-ESI (APCI+): calculated for $C_{13}H_8O_3SH^+$ $[M+H]^+$ 245.0267, found 245.0265.

Dimethyl 11,11'-((9-oxo-9*H*-thioxanthene-4,5-diyl)bis(oxy))diundecanoate (28)

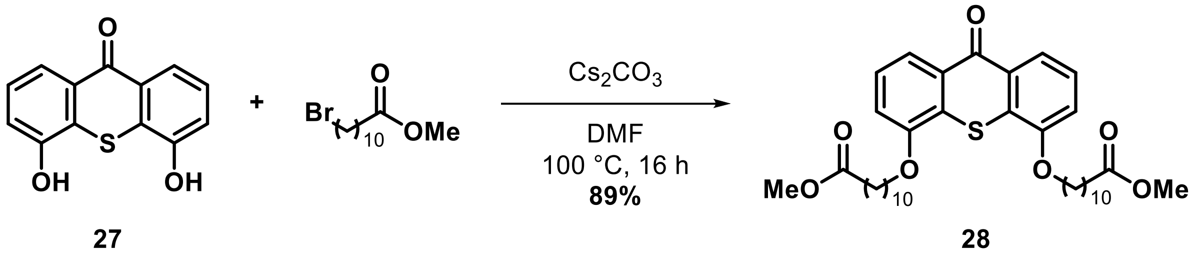

An oven-dried crimp top vial equipped with a magnetic stirring bar was charged with and **27** (0.81 g, 3.30 mmol, 1.0 eq.) and Cs_2CO_3 (5.38 g, 16.5 mmol, 5.0 eq.). The vial was crimped, flushed with nitrogen and DMF (35 mL) and methyl 11-bromoundecanoate (2.4 mL, 2.77 g, 9.91 mmol, 3.0 eq.) were added. The mixture was heated at 100 °C for 16 h. The mixture was allowed to cool down to room temperature, EtOAc and aqueous 1M HCl were added and the phases separated. The organic layer was washed with aqueous LiCl (10% w/v) and brine, dried over $MgSO_4$, the solvent removed under reduced pressure and the crude product purified *via* recrystallization (MeOH), yielding **28** as a yellow solid (1.88 g, 2.93 mmol, 89%).

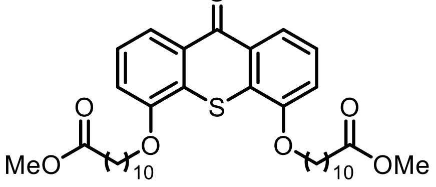

28
$C_{37}H_{52}O_7S$
Mw = 640.88 g/mol

^{1}H NMR (400 MHz, $CDCl_3$): δ = 8.22 (d, *J* = 8.2 Hz, 2H), 7.40 (t, *J* = 8.0 Hz, 2H), 7.11 (d, *J* = 8.1 Hz, 2H), 4.17 (t, *J* = 6.5 Hz, 4H), 3.65 (s, 6H), 2.28 (t, *J* = 7.5 Hz, 4H), 1.92 (p, *J* = 6.7 Hz, 4H), 1.66 – 1.50 (m, 8H), 1.30 (s, 20H).
^{13}C{^{1}H} NMR (101 MHz, $CDCl_3$): δ = 180.5, 174.4, 154.5, 130.1, 128.5, 125.9, 121.4, 113.2, 69.6, 51.5, 34.2, 29.7, 29.5, 29.4, 29.4, 29.3, 29.1, 26.1, 25.1.
HRMS-ESI (ESI+): calculated for $C_{37}H_{52}O_7SNa^+$ $[M+Na]^+$ 663.3326, found 663.3315.

General Procedures & Tips for Molecular Motors

1st Generation Molecular Motor (McMurry Reaction)

A flame-dried Schlenk flask/tube equipped with a magnetic stirring egg was charged with Zn powder (4.5 eq.) and flushed with nitrogen (3x). Dry dioxane (0.5M) was added and the reaction mixture cooled down to 0 ºC (ice/H_2O). $TiCl_4$ (2.1 eq.) was added dropwise and the resulting mixture heated at 100 ºC for 1 h. The reaction was allowed to cool down to room temperature, **ketone** (1.0 eq.) was added and the mixture heated at 100 ºC for 16 h. The reaction mixture was allowed to cool down to room temperature, was diluted with Et_2O and filtered over a glass filter frit (p3) with silica. The silica was washed with Et_2O, the organic solvent removed under reduced pressure and purified using flash column chromatography, yielding **MM**.

advice/observations:

- The *cis*-isomer can be obtained by thermal isomerization of **MM** in DMSO (0.5M) at 190 °C for 1–3 h.
- The *trans*-isomer can be obtained by irradiation of **MM** with a 310 nm LED and separated *via* flash column chromatography (very slow gradient from toluene to 10% acetone/toluene). This method primarily works for more polar first generation molecular motors, such as the di-alcohol, the di-aldehyde or the di-nitrile **MM**.

Thioketone Formation

An oven dried crimp top vial equipped with a magnetic stirring bar was charged with **ketone** (1.0 eq.) and Lawesson's reagent (1.1 eq.). The vial was crimped, flushed with nitrogen and dry toluene (0.1M) was added and the reaction mixture was heated at 115 ºC for 3 h. The reaction mixture was allowed to cool down to room temperature, concentrated and the crude purified *via* flash column chromatography (inert atmosphere), yielding **thioketone**, which was immediately further reacted.

advice/observations:

- Most of the times isolated as a colorful oil.
- The thioketone displays lower polarity than the ketone, resulting in higher retention factors TLC.

Hydrazone Formation

Procedure 1

A round bottom flask equipped with a magnetic stirring egg and a reflux condenser was charged with **fluorenone** (1.0 eq.), dry EtOH (0.05–0.1M), DMF (cat.) and hydrazine monohydrate (50–60%, 6.5 eq.). The resulting reaction mixture was stirred and heated at 95 ºC for 5 h. Subsequently, the reaction mixture was allowed to cool down to room temperature and the solvents removed under reduced pressure, yielding **hydrazone**.

Procedure 2

A flame dried Schlenk flask/tube equipped with a magnetic stirring egg was charged with **fluorenone** (1.0 eq.), dry degassed DMF (0.2M) and hydrazine monohydrate (50–60%, 6.5 eq.). The resulting reaction mixture was stirred and heated at 100 °C for 16 h. The reaction mixture was allowed to cool down to room temperature, poured on water and extracted with CH_2Cl_2 (3x). The combined organic layers were washed with aqueous LiCl (5% w/v, 2x) and brine. The solvent was removed under reduced pressure and the crude product purified *via* flash column chromatography.

advice/observations:

- Too high concentrations can lead to homocoupling.
- Too much water favors the backreaction.
- Procedure 2 is more effective on 4*H*-thiochromen-4-one based derivatives.

Diazo Formation

Under light exclusion, a flame dried Schlenk flask/tube equipped with a magnetic stirring egg was charged **hydrazone** (1.0 eq.) and dry THF (0.1M). The reaction mixture was cooled down to 0 ºC (ice/H_2O), MnO_2 (activated, 4.0 eq.) added and the reaction mixture stirred for 5 min at 0 ºC and then 3 h at room temperature. The reaction mixture was filtered over celite with CH_2Cl_2 and the solvents removed under reduced pressure, yielding **diazo**, which was immediately further reacted.

advice/observations:

- The filtration can be performed under inert atmosphere.
- For very sensitive compounds, stirring can be stopped, the MnO_2 settles then to the bottom of the flask/tube, and the reaction mixture can be filtered over a canula equipped with a Whatman filter directly into the Barton-Kellogg coupling reaction.

2nd Generation Molecular Motor (Barton-Kellogg Reaction)

An oven-dried crimp top vial equipped with a magnetic stirring bar was charged with **thioketone** (1.0 eq.) and **diazo** (1.0 eq.) dissolved in dry THF (0.1–0.15M) and stirred for 24 h at room temperature under a nitrogen atmosphere. Subsequently, hexamethylphosphorous triamide (4.0 eq.) was added and the reaction mixture stirred for another 24 h at room temperature. The solvents were removed under reduced pressure, and the crude product purified *via* flash column chromatography, yielding **MM**.

advice/observations:

- The reaction should be performed under light exclusion.
- The less toxic HEPT can be used instead of HMPT.
- Generated episulfides are primarily isolated in the case of 6-membered bottom halves, whereas PPh_3 is used as desulfuration agent.

References

(1) Dietrich, U.; Hackmann, M.; Rieger, B.; Klinga, M.; Leskelä, M. Control of Stereoerror Formation with High-Activity “Dual-Side” Zirconocene Catalysts: A Novel Strategy to Design the Properties of Thermoplastic Elastic Polypropenes. *J. Am. Chem. Soc.* **1999**, *121* (18), 4348–4355. https://doi.org/10.1021/ja9833220.

(2) Štacko, P.; Kistemaker, J. C. M.; Feringa, B. L. Fluorine-Substituted Molecular Motors with a Quaternary Stereogenic Center. *Chem. Eur. J.* **2017**, *23* (27), 6643–6653. https://doi.org/10.1002/chem.201700581.

(3) Pfeifer, L.; Scherübl, M.; Fellert, M.; Danowski, W.; Cheng, J.; Pol, J.; Feringa, B. L. Photoefficient 2nd Generation Molecular Motors Responsive to Visible Light. *Chem. Sci.* **2019**, *10* (38), 8768–8773. https://doi.org/10.1039/c9sc02150g.

(4) Pfeifer, L.; Hoang, N. V; Scherübl, M.; Pshenichnikov, M. S.; Feringa, B. L. Powering Rotary Molecular Motors with Low-Intensity near-Infrared Light. *Sci. Adv.* **2020**, *6*, eabb6165. https://doi.org/DOI:10.1126/sciadv.abb6165.

(5) Offner, J. D.; Schnakenburg, G.; Rose-Munch, F.; Rose, E.; Dötz, K. H. Heterobimetallic Dibenzoindenyl (Cr(CO)3-Re(CO)3) Complexes via Chromium-Templated [3 + 2 + 1]Benzannulation: Synthesis and Molecular Structures. *Inorg. Chem.* **2011**, *50* (17), 8153–8157. https://doi.org/10.1021/ic200628a.

(6) Neubauer, T. M.; Van Leeuwen, T.; Zhao, D.; Lubbe, A. S.; Kistemaker, J. C. M.; Feringa, B. L. Asymmetric Synthesis of First Generation Molecular Motors. *Org. Lett.* **2014**, *16* (16), 4220–4223. https://doi.org/10.1021/ol501925f.

(7) Chen, X.; Li, M.; Liu, Z.; Yang, C.; Xie, H.; Hu, X.; Su, S. J.; Jiang, H.; Zeng, W. Bimetal Cooperatively Catalyzed Arylalkynylation of Alkynylsilanes. *Org. Lett.* **2021**, *23* (17), 6724–6728. https://doi.org/10.1021/acs.orglett.1c02283.

(8) Bachmann, W. E.; Cook, J. W.; Hewett, C. L.; Iball, J. The Synthesis of Compounds Related to the Sterols, Bile Acids, and Oestrus-Producing Hormones. Part X. Ruzicka’s Hydrocarbon “C21H16” from Cholic Acid. *J. Chem. Soc.* **1936**, 54–61. https://doi.org/https://doi.org/10.1039/JR9360000054.

(9) London, G.; Carroll, G. T.; Fernández Landaluce, T.; Pollard, M. M.; Rudolf, P.; Feringa, B. L. Light-Driven Altitudinal Molecular Motors on Surfaces. *Chem. Commun.* **2009**, No. 13, 1712–1714. https://doi.org/10.1039/b821755f.

(10) Li, Q.; Foy, J. T.; Colard-Itté, J. R.; Goujon, A.; Dattler, D.; Fuks, G.; Moulin, E.; Giuseppone, N. Gram Scale Synthesis of Functionalized and Optically Pure Feringa’s Motors. *Tetrahedron* **2017**, *73* (33), 4874–4882. https://doi.org/10.1016/j.tet.2017.05.023.

(11) Van Dijken, D. J.; Chen, J.; Stuart, M. C. A.; Hou, L.; Feringa, B. L. Amphiphilic Molecular Motors for Responsive Aggregation in Water. *J. Am. Chem. Soc.* **2016**, *138* (2), 660–669. https://doi.org/10.1021/jacs.5b11318.

(12) Kobin, B.; Grubert, L.; Blumstengel, S.; Henneberger, F.; Hecht, S. Vacuum-Processable Ladder-Type Oligophenylenes for Organic-Inorganic Hybrid Structures: Synthesis, Optical and Electrochemical Properties upon Increasing Planarization as Well as Thin Film Growth. *J. Mat. Chem.* **2012**, *22* (10), 4383–4390. https://doi.org/10.1039/c2jm15868j.

(13) Usami, K.; Yamaguchi, E.; Tada, N.; Itoh, A. Transition-Metal-Free Synthesis of Phenanthridinones through Visible-Light-Driven Oxidative C–H Amidation. *Eur. J. Org. Chem.* **2020**, No. 10, 1496–1504. https://doi.org/10.1002/ejoc.201900536.

(14) Pünner, F.; Schieven, J.; Hilt, G. Synthesis of Fluorenone and Anthraquinone Derivatives from Aryl- and Aroyl-Substituted Propiolates. *Org. Lett.* **2013**, *15* (18), 4888–4891. https://doi.org/10.1021/ol4023276.

(15) Ibraheem, W.; Chaar, C.; Camiade, E.; Hervé, V.; Fouquenet, D.; Roux, A. E.; Si-Tahar, M.; Ahmed, E.; Thibonnet, J.; Thiery, E.; Petrignet, J. Synthesis, Antibacterial and Cytotoxic Evaluation of Cytosporone E and Analogs. *J. Mol. Struct.* **2022**, *1252*, 132135. https://doi.org/10.1016/j.molstruc.2021.132135.

(16) Pollard, M. M.; Lubomska, M.; Rudolf, P.; Feringa, B. L. Controlled Rotary Motion in a Monolayer of Molecular Motors. *Angew. Chem. Int. Ed.* **2007**, *46* (8), 1278–1280. https://doi.org/10.1002/anie.200603618.

Appendix

NMR Spectra

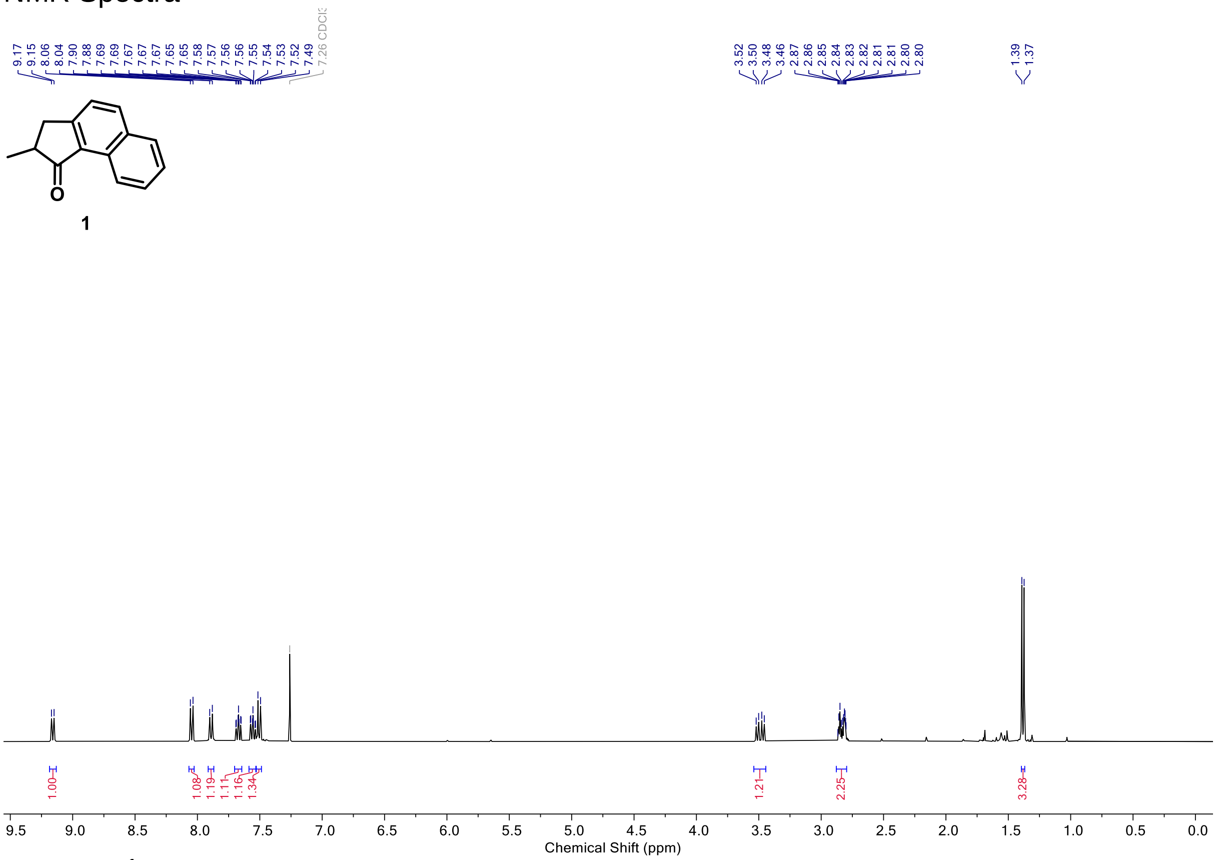

Figure S1: ¹H NMR spectrum of **1** (400 MHz, $CDCl_3$).

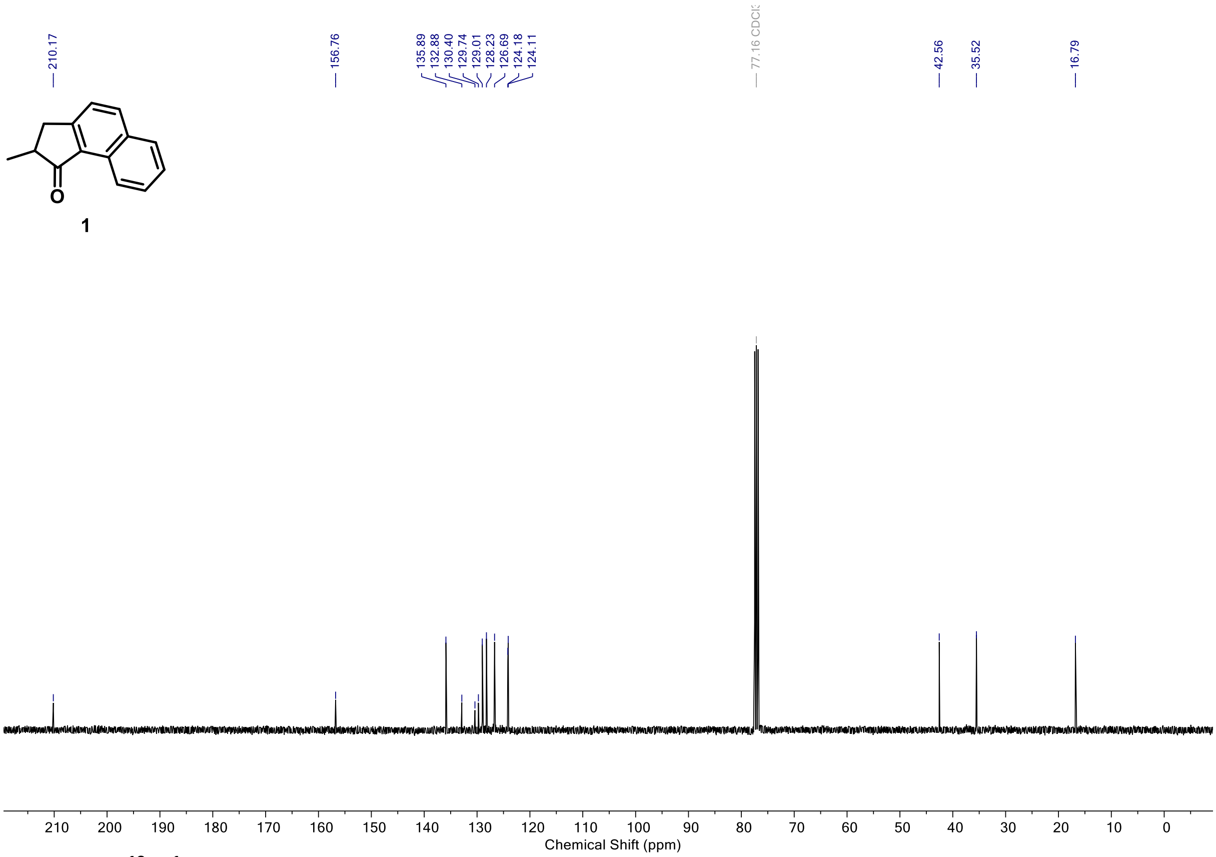

Figure S2: ¹³C{¹H} NMR spectrum of **1** (101 MHz, $CDCl_3$).

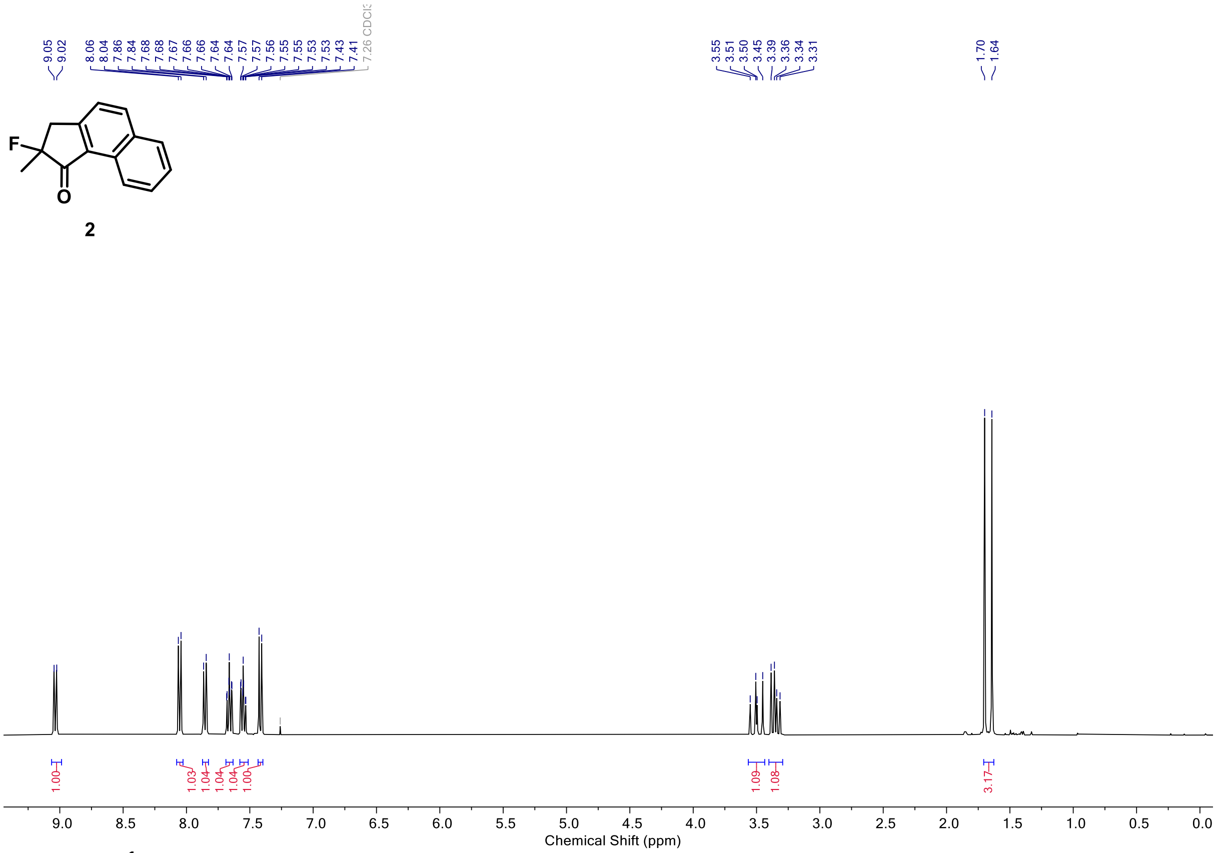

Figure S3: **^{1}H NMR** spectrum of **2** (400 MHz, $CDCl_3$).

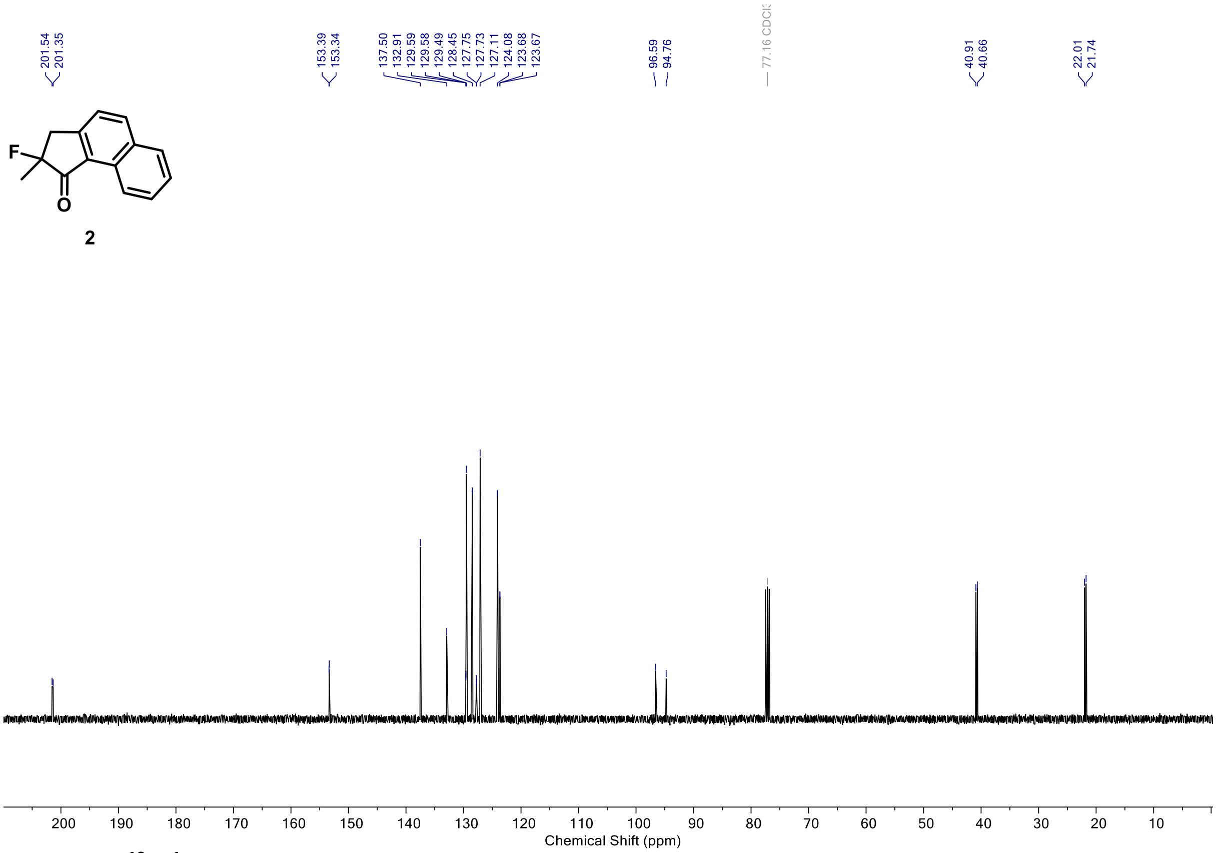

Figure S4: **^{13}C{^{1}H} NMR** spectrum of **2** (101 MHz, $CDCl_3$).

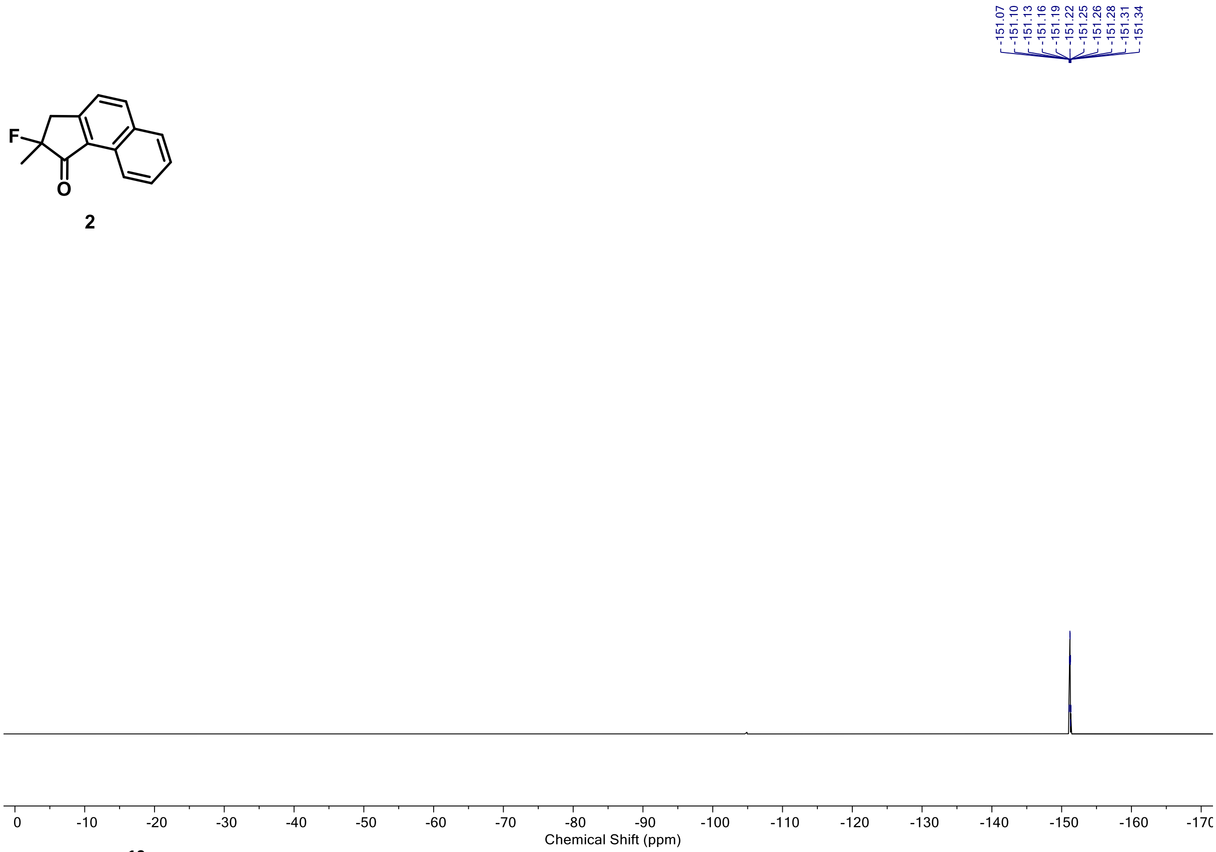

Figure S5: ^{19}F NMR spectrum of **2** (376 MHz, $CDCl_3$).

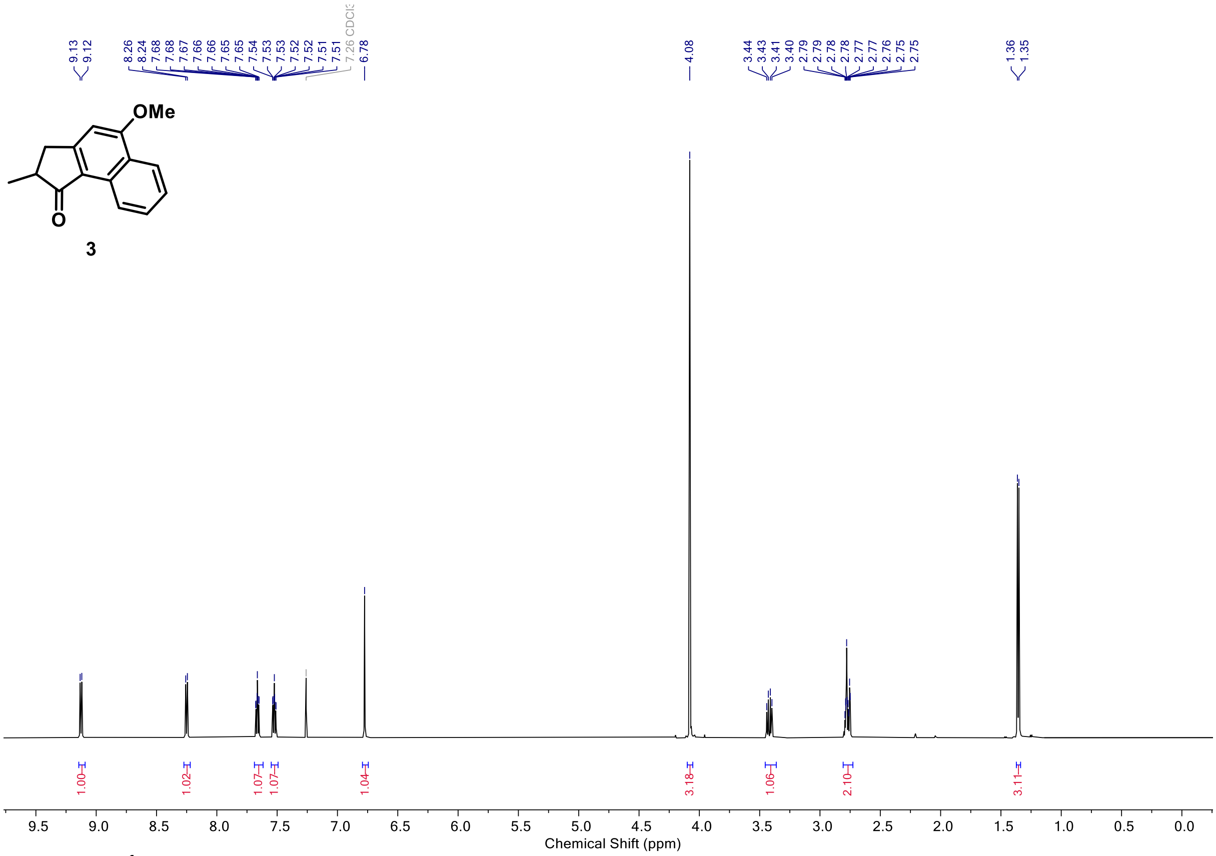

Figure S6: ^{1}H NMR spectrum of **3** (600 MHz, $CDCl_3$).

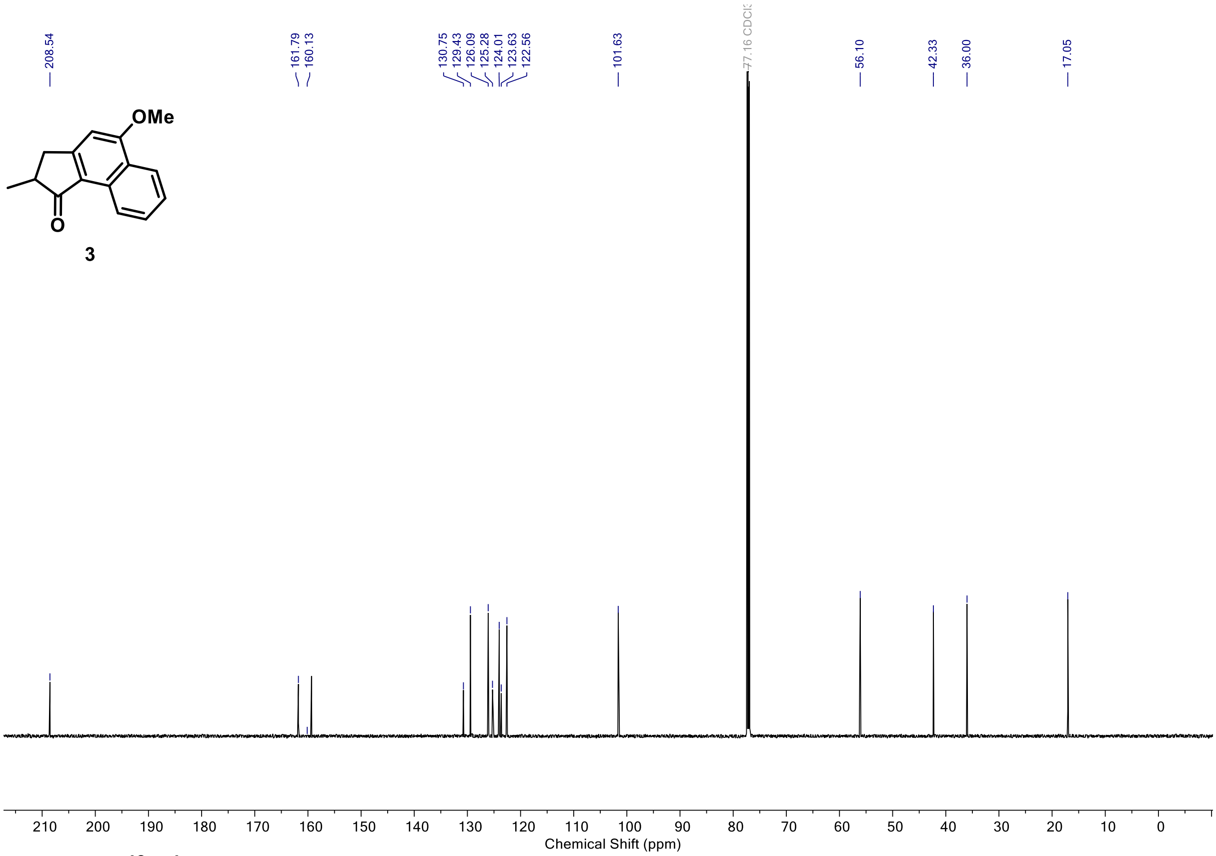

Figure S7: ^{13}C{^{1}H} NMR spectrum of **3** (151 MHz, $CDCl_3$).

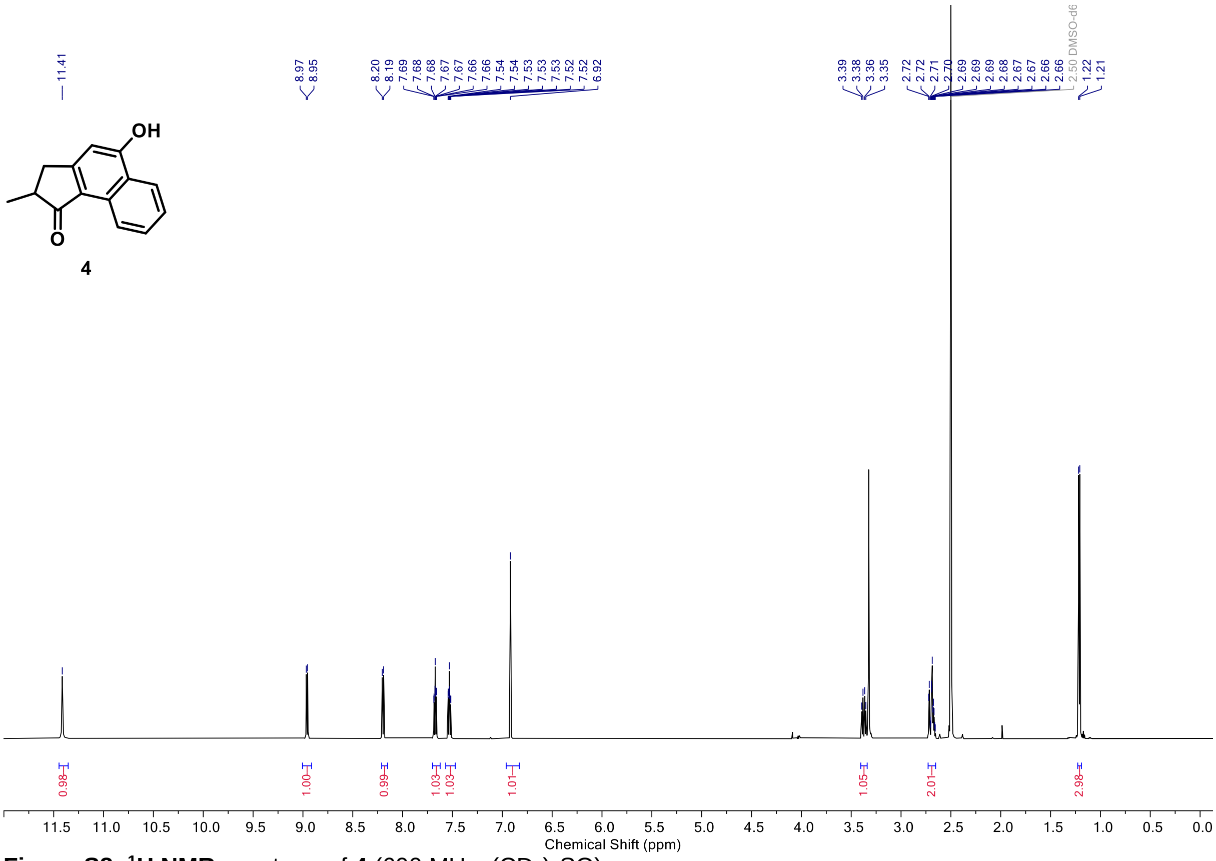

Figure S8: ^{1}H NMR spectrum of **4** (600 MHz, $(CD_3)_2SO$).

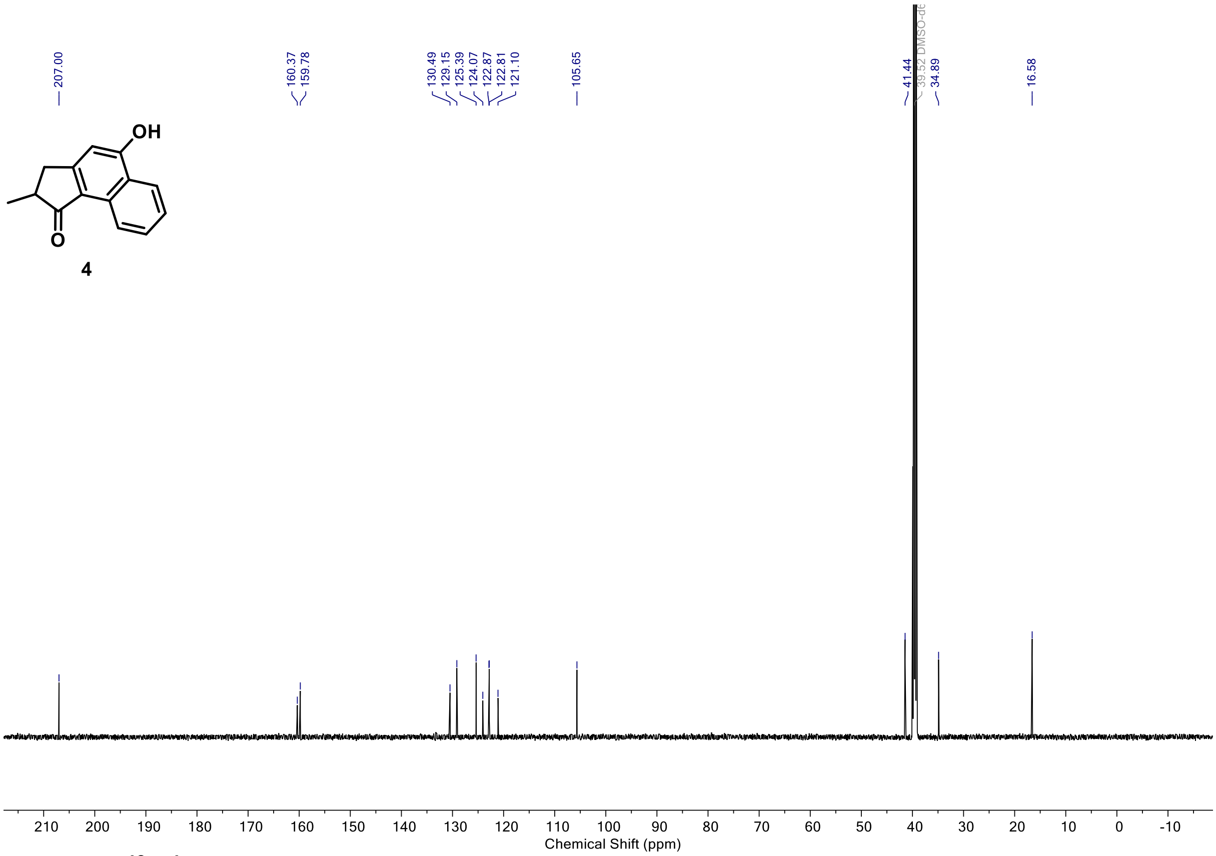

Figure S9: ^{13}**C{**^{1}**H} NMR** spectrum of **4** (151 MHz, $(CD_3)_2SO$).

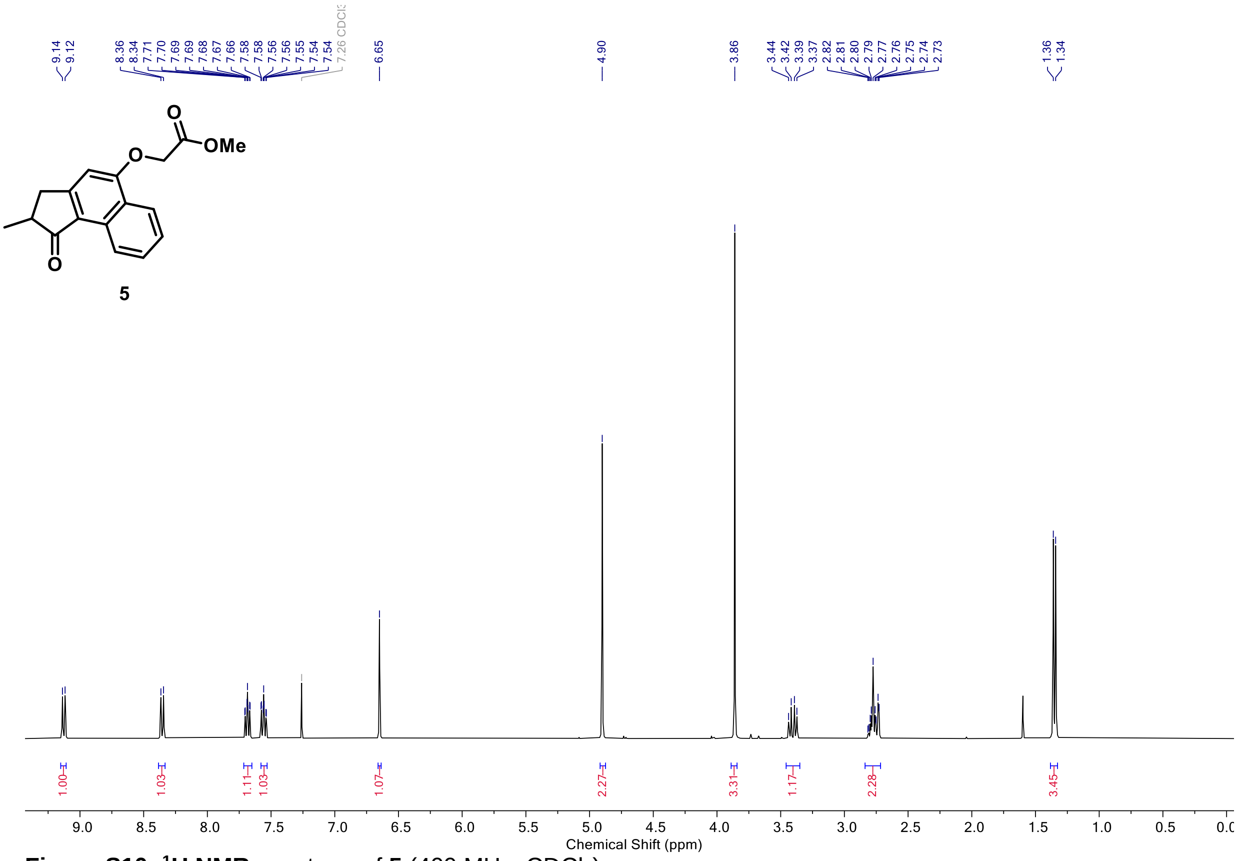

Figure S10: ^{1}**H NMR** spectrum of **5** (400 MHz, $CDCl_3$).

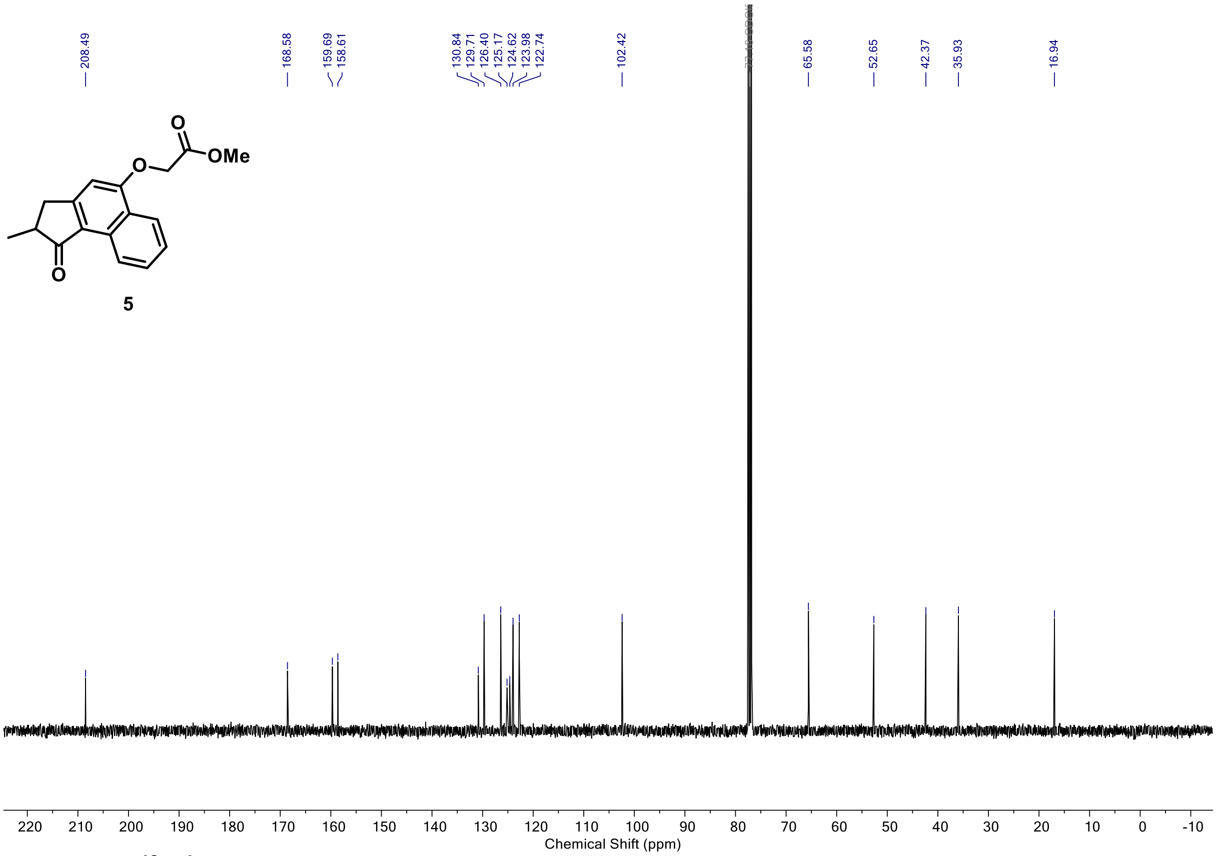

Figure S11: $^{13}C\{^{1}H\}$ NMR spectrum of **5** (101 MHz, $CDCl_3$).

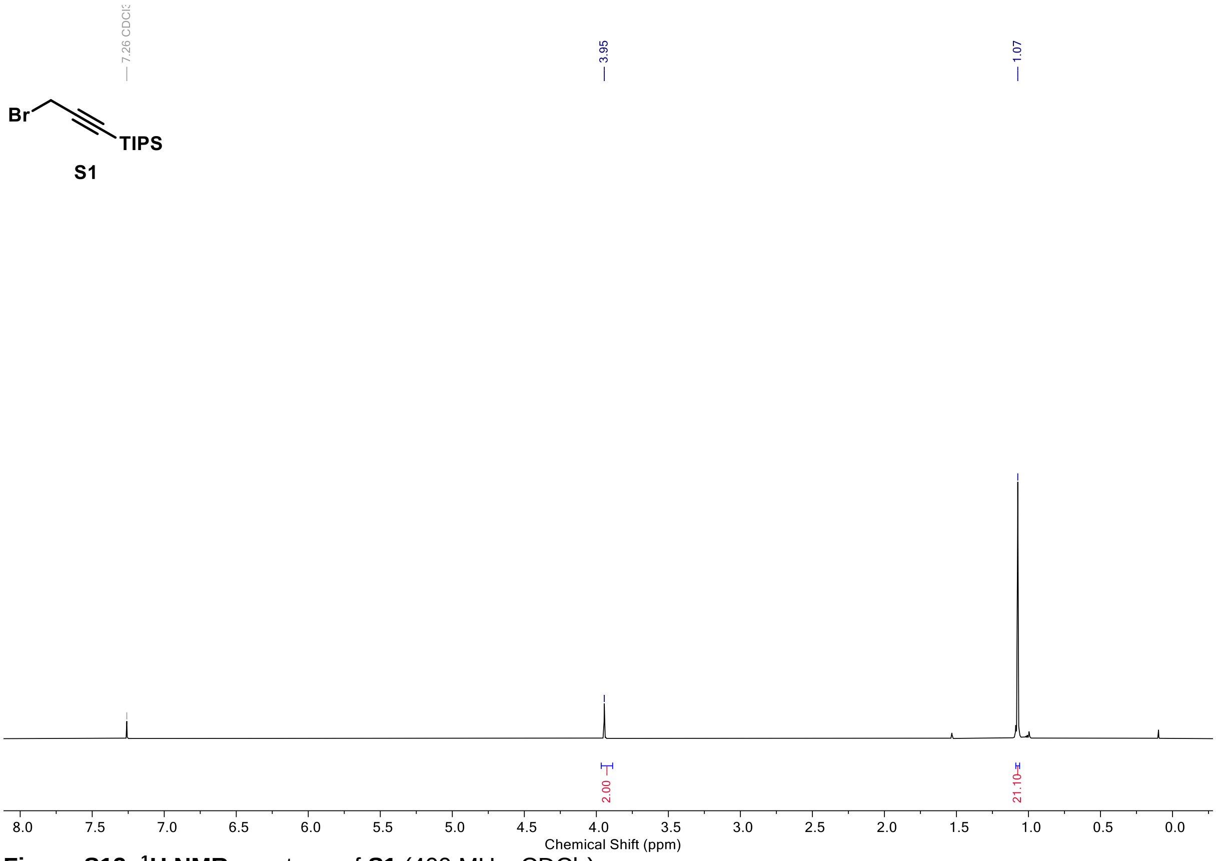

Figure S12: ^{1}H NMR spectrum of **S1** (400 MHz, $CDCl_3$).

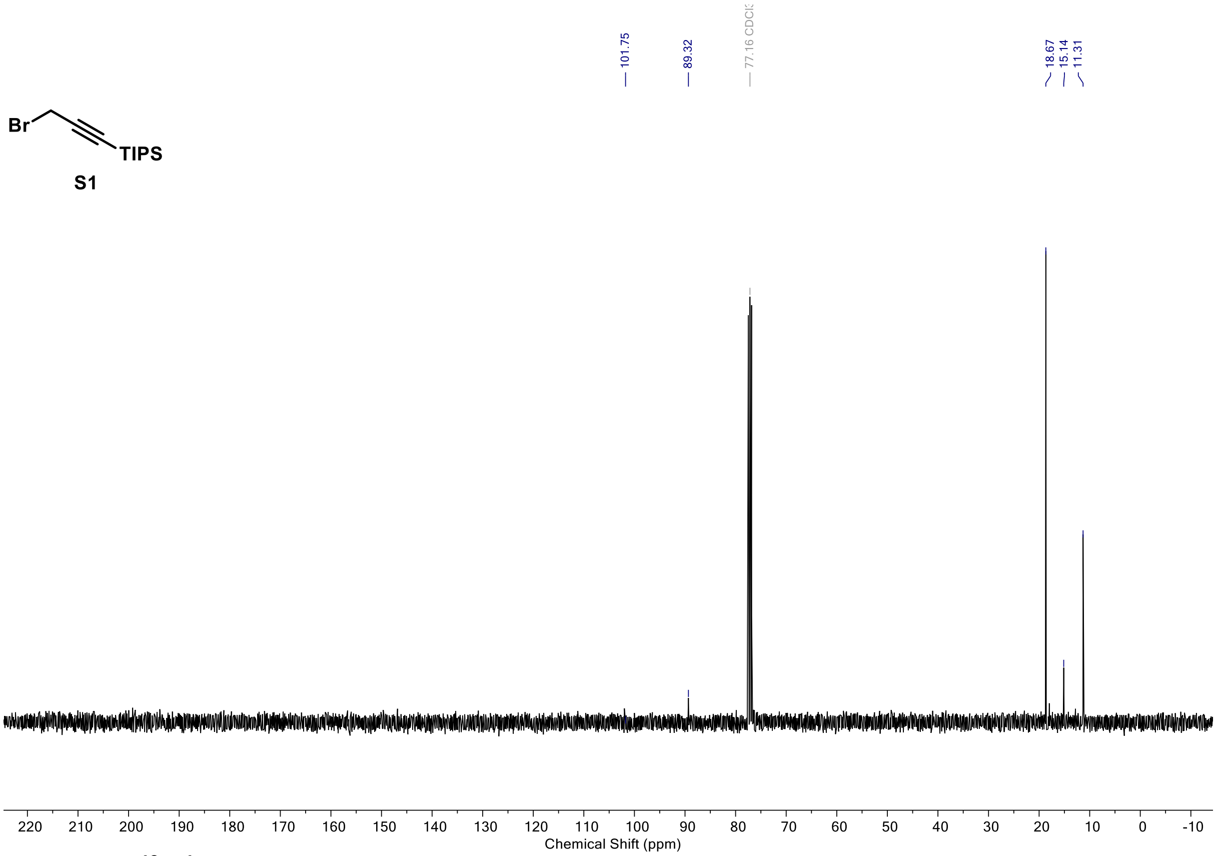

Figure S13: $^{13}C\{^{1}H\}$ NMR spectrum of S1 (101 MHz, $CDCl_3$).

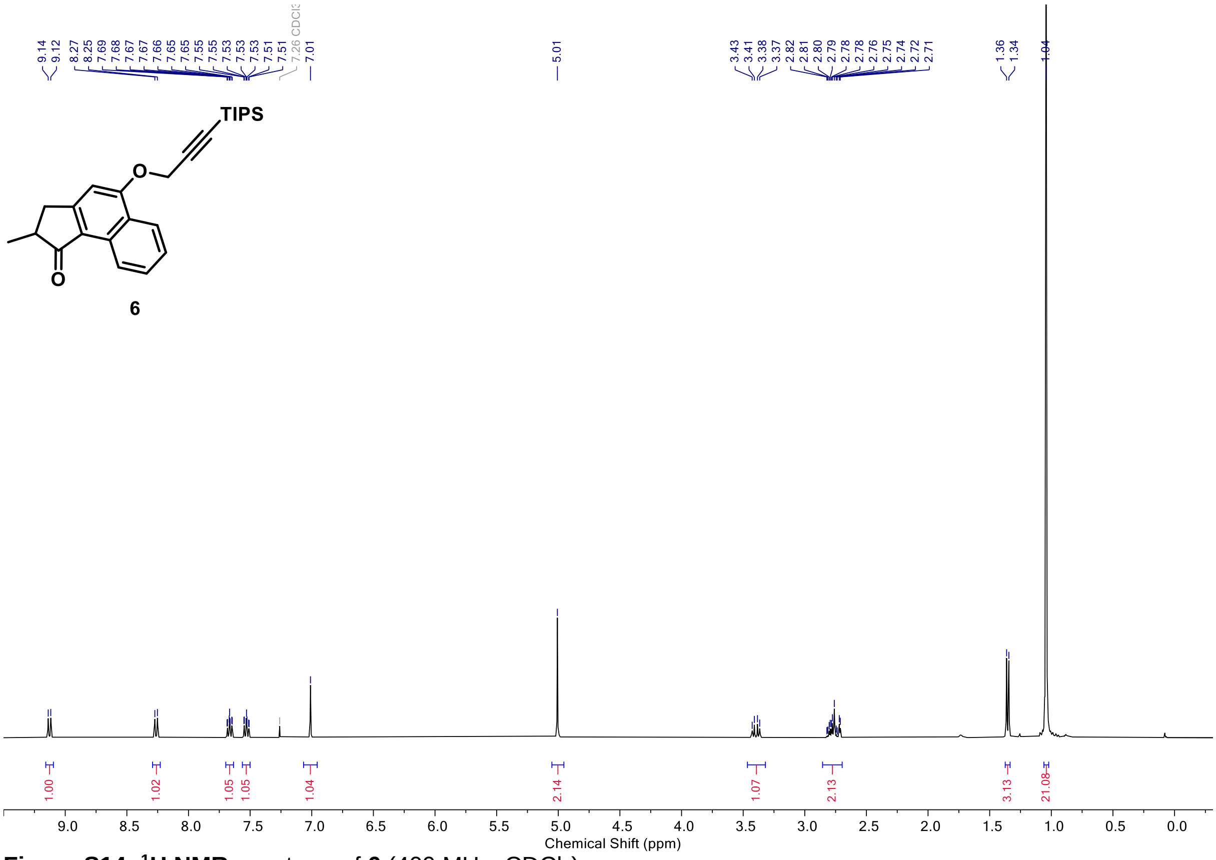

Figure S14: ^{1}H NMR spectrum of 6 (400 MHz, $CDCl_3$).

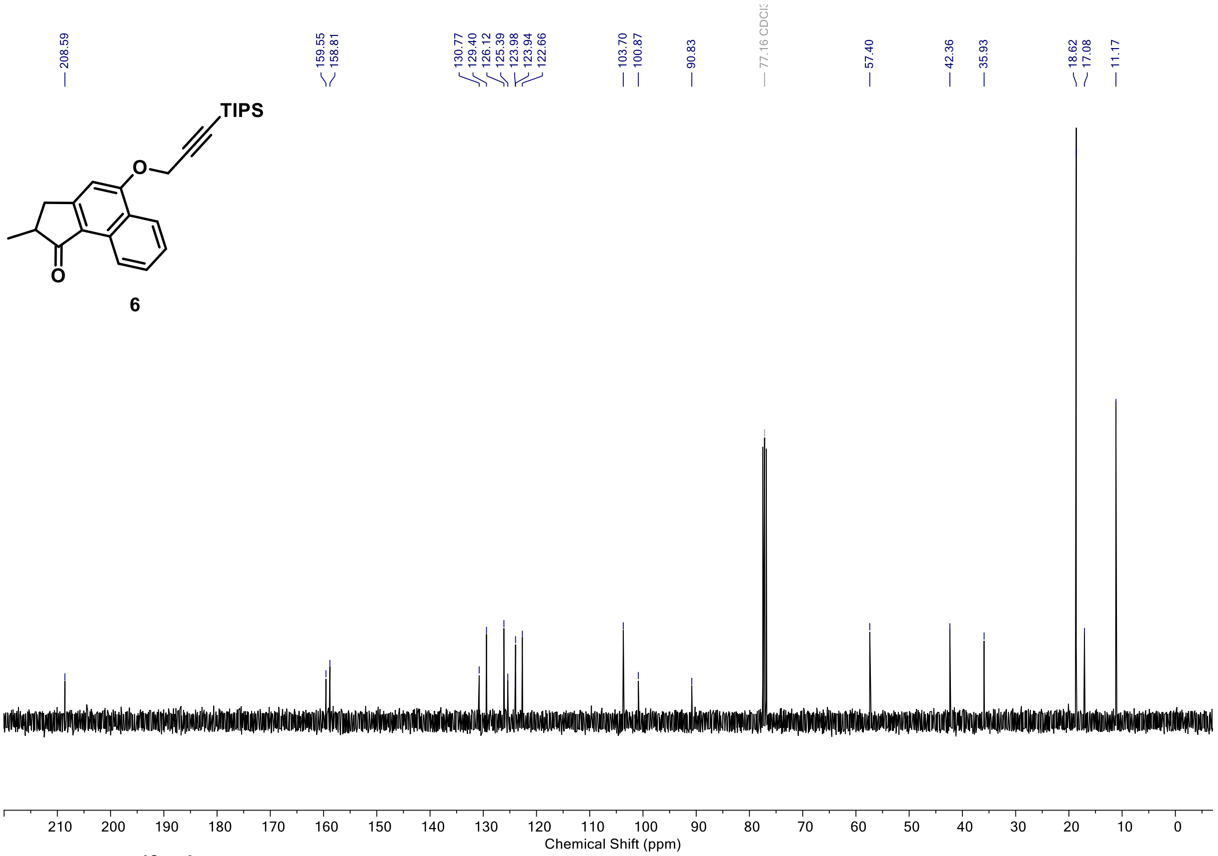

Figure S15: **$^{13}C\{^1H\}$ NMR** spectrum of **6** (101 MHz, $CDCl_3$).

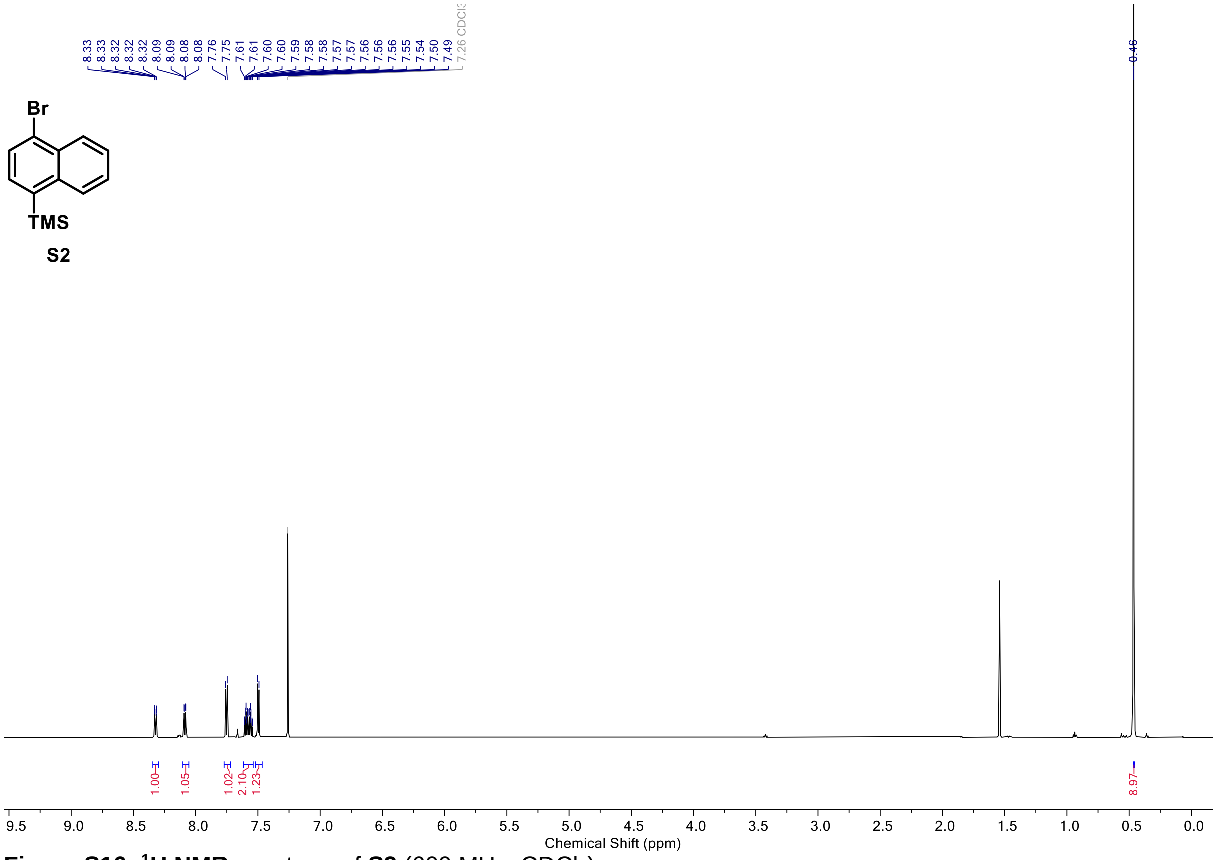

Figure S16: **^1H NMR** spectrum of **S2** (600 MHz, $CDCl_3$).

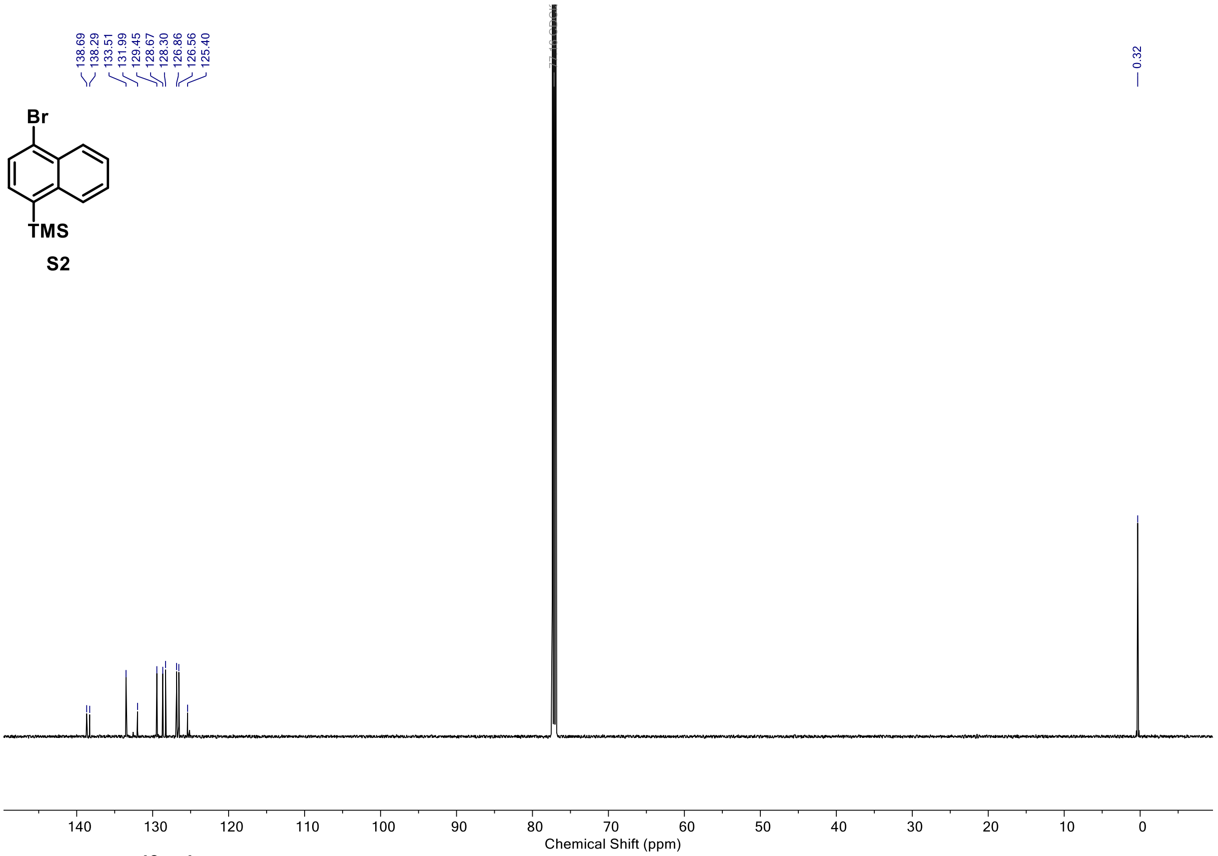

Figure S17: ^{13}C{^{1}H} NMR spectrum of **S2** (151MHz, CDCl$_3$).

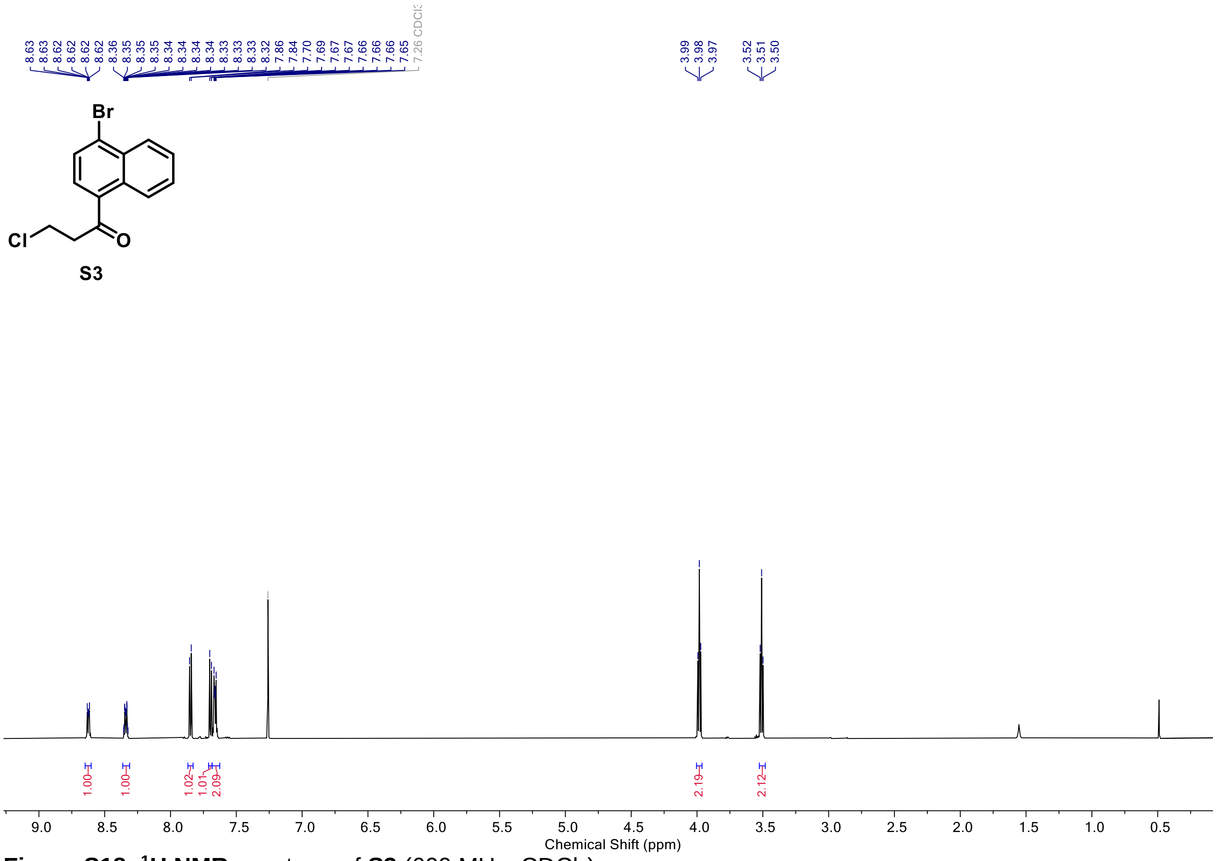

Figure S18: ^{1}H NMR spectrum of **S3** (600 MHz, CDCl$_3$).

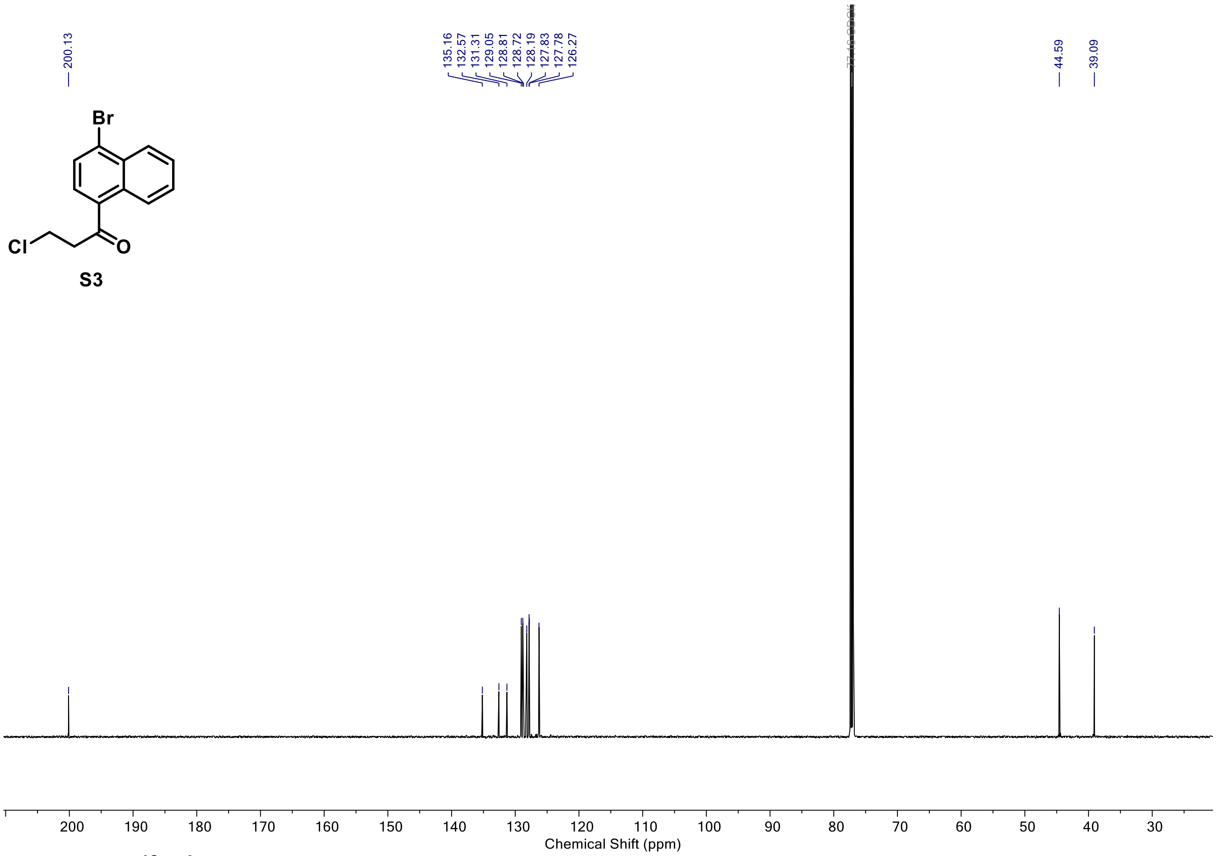

Figure S19: ^{13}C{^{1}H} NMR spectrum of **S3** (151 MHz, CDCl$_3$).

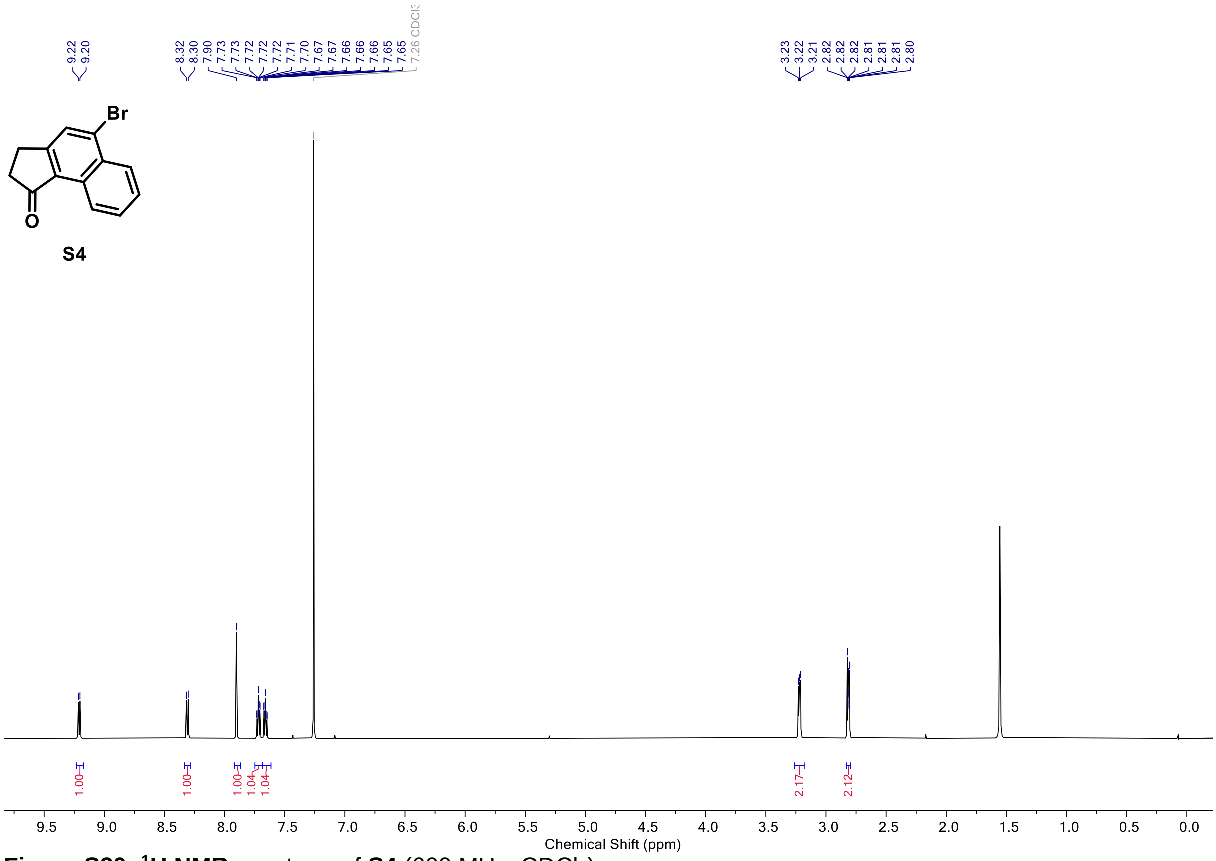

Figure S20: ^{1}H NMR spectrum of **S4** (600 MHz, CDCl$_3$).

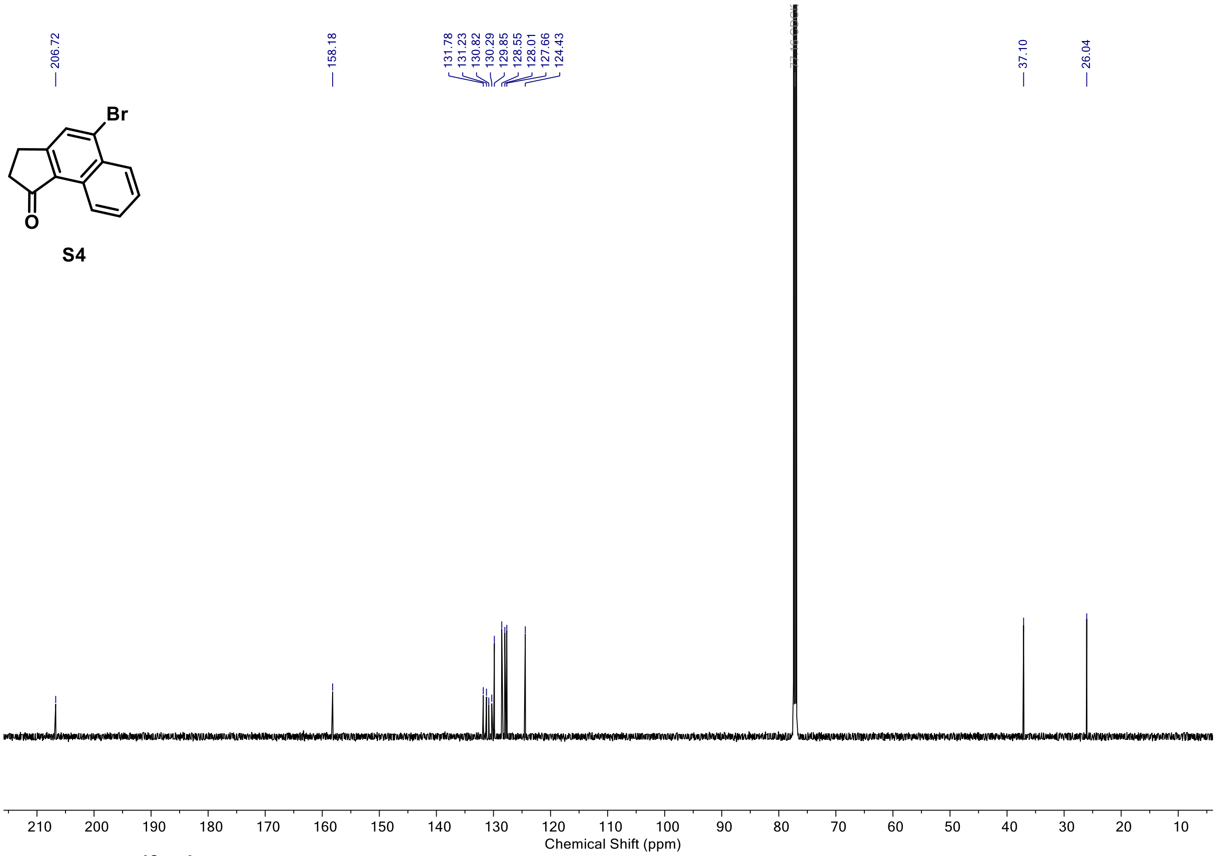

Figure S21: ^{13}C{^{1}H} NMR spectrum of **S4** (151 MHz, $CDCl_3$).

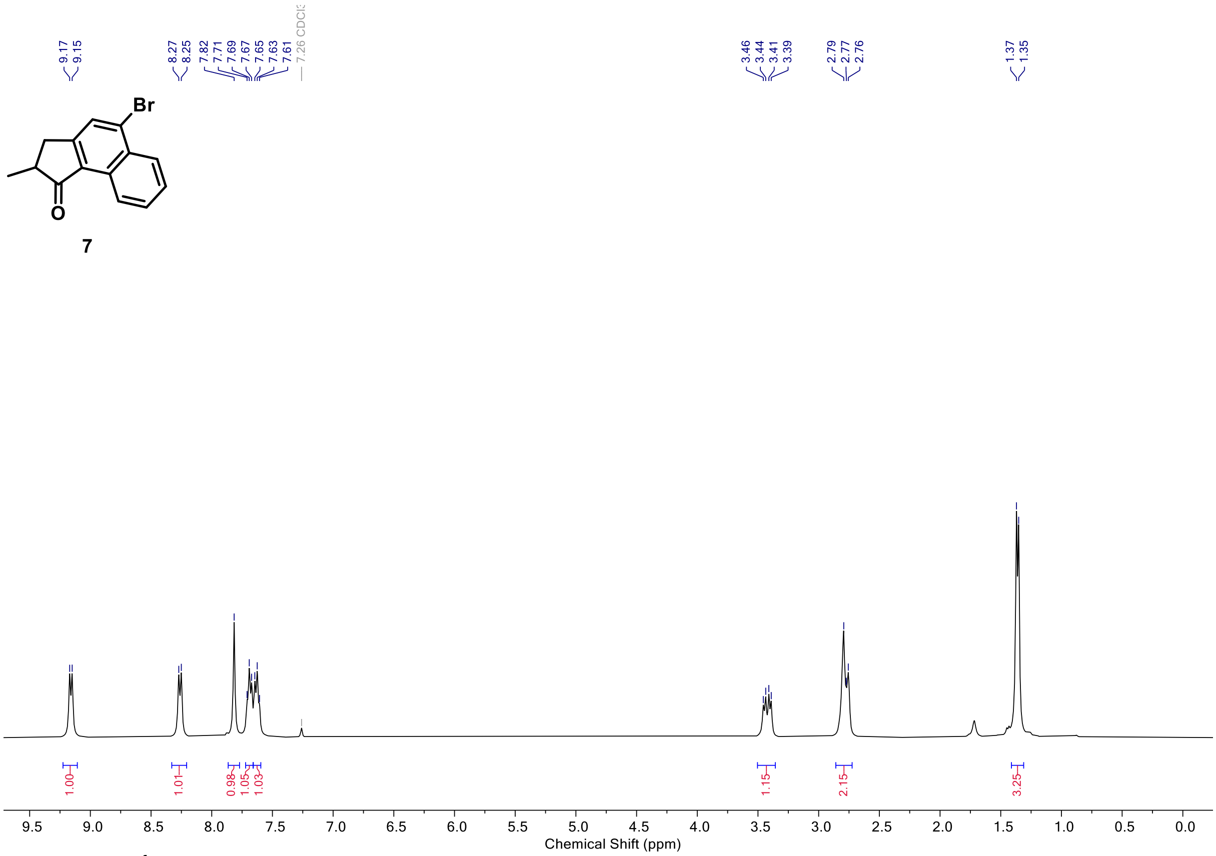

Figure S22: ^{1}H NMR spectrum of **7** (400 MHz, $CDCl_3$).

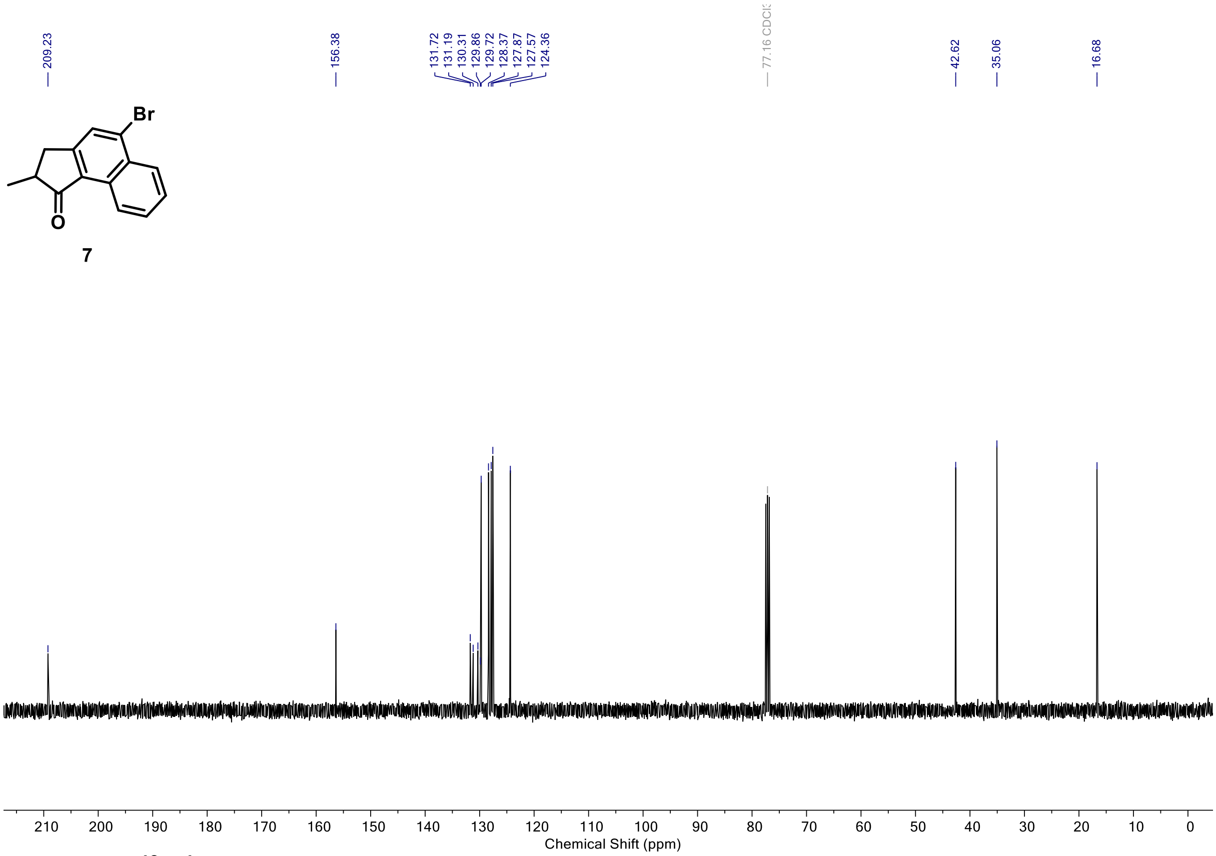

Figure S23: ^{13}C{^{1}H} NMR spectrum of **7** (101 MHz, $CDCl_3$).

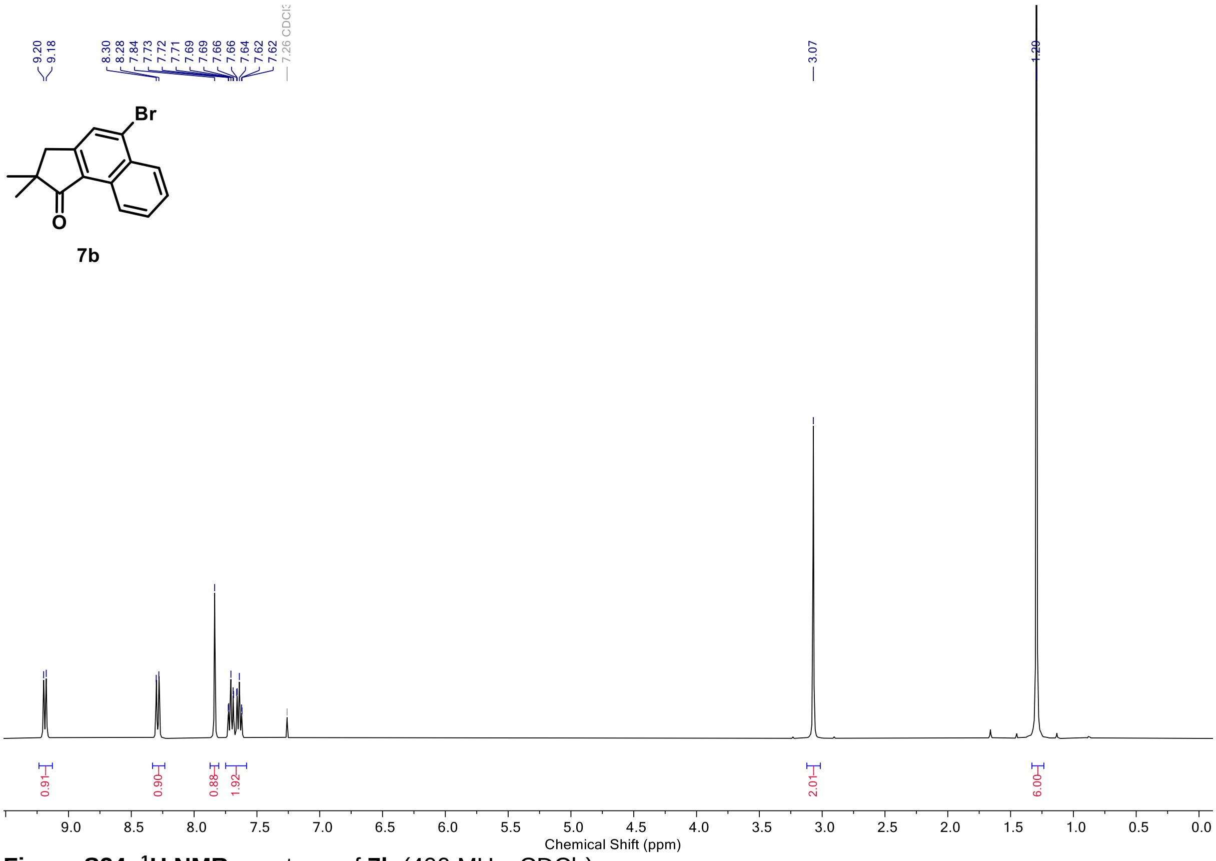

Figure S24: ^{1}H NMR spectrum of **7b** (400 MHz, $CDCl_3$).

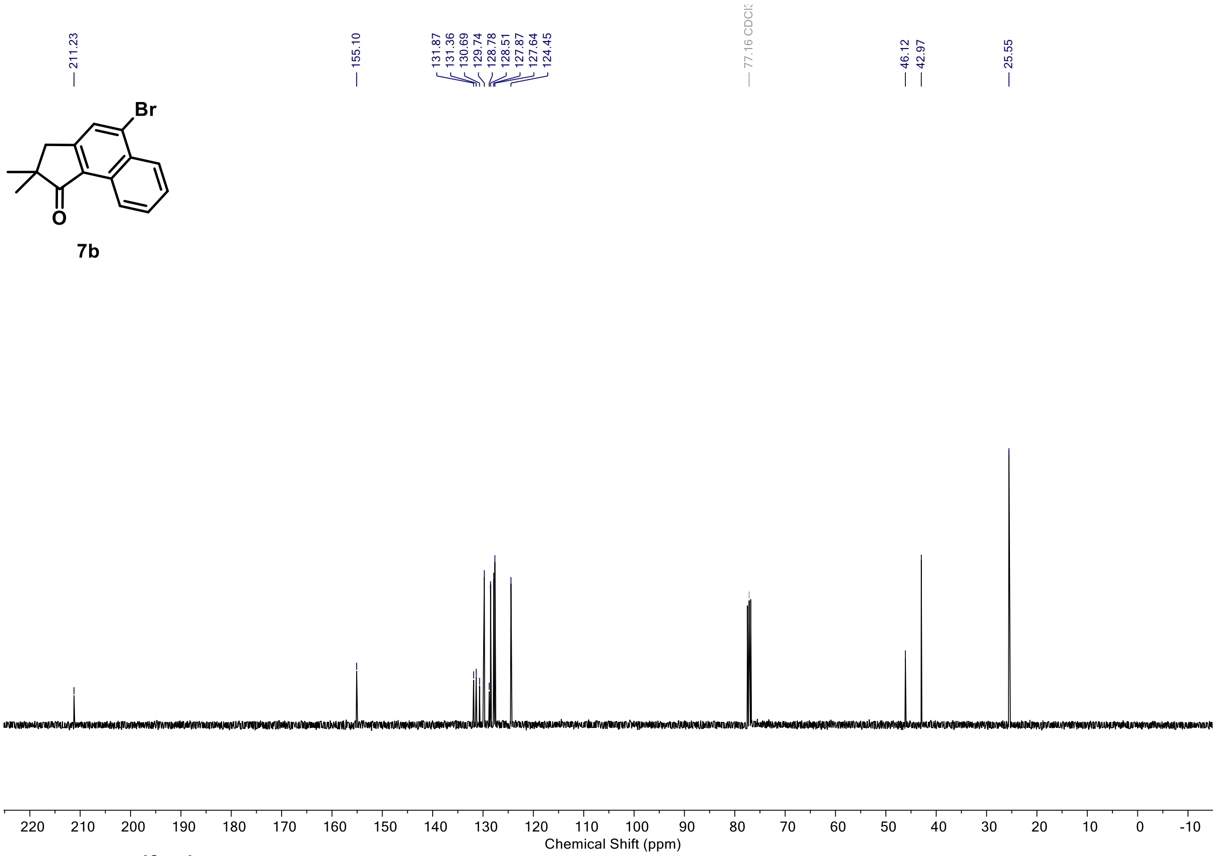

Figure S25: ^{13}C{^{1}H} NMR spectrum of **7b** (101 MHz, $CDCl_3$).

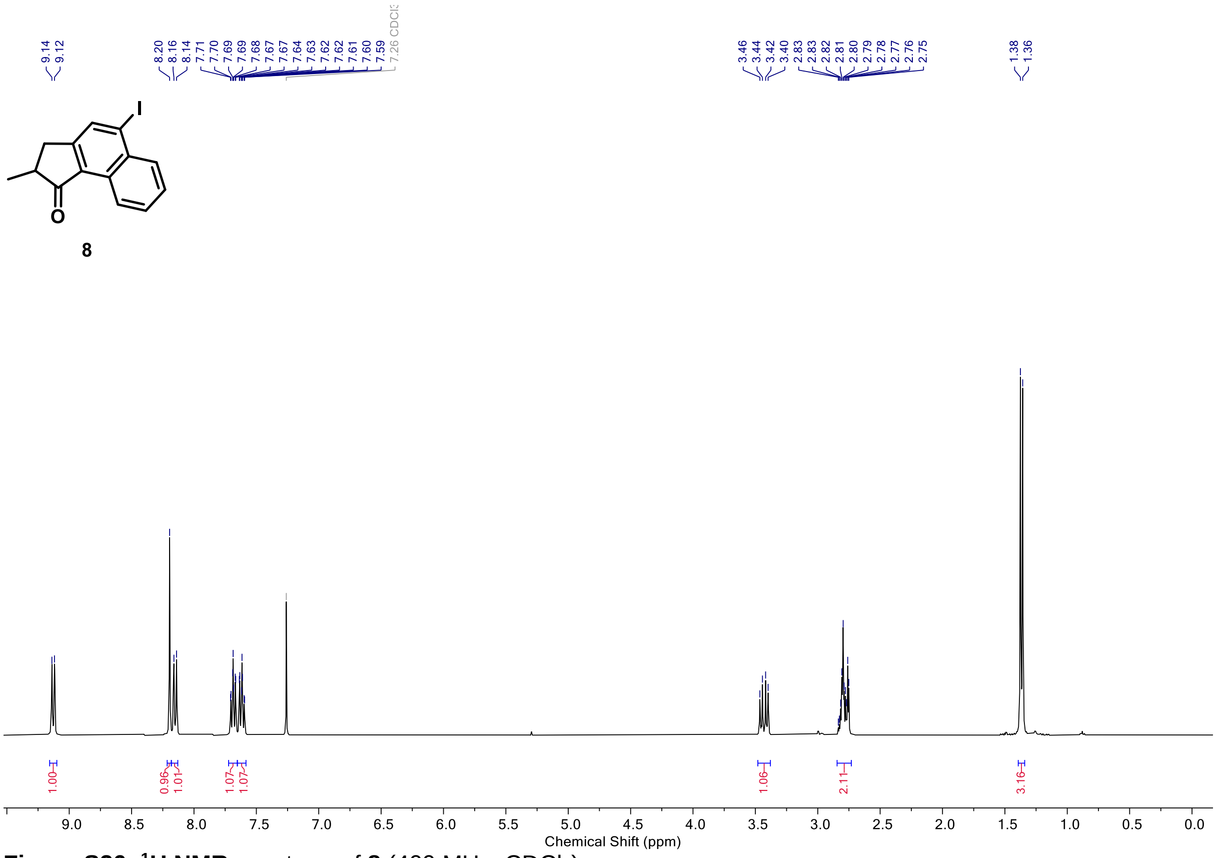

Figure S26: ^{1}H NMR spectrum of **8** (400 MHz, $CDCl_3$).

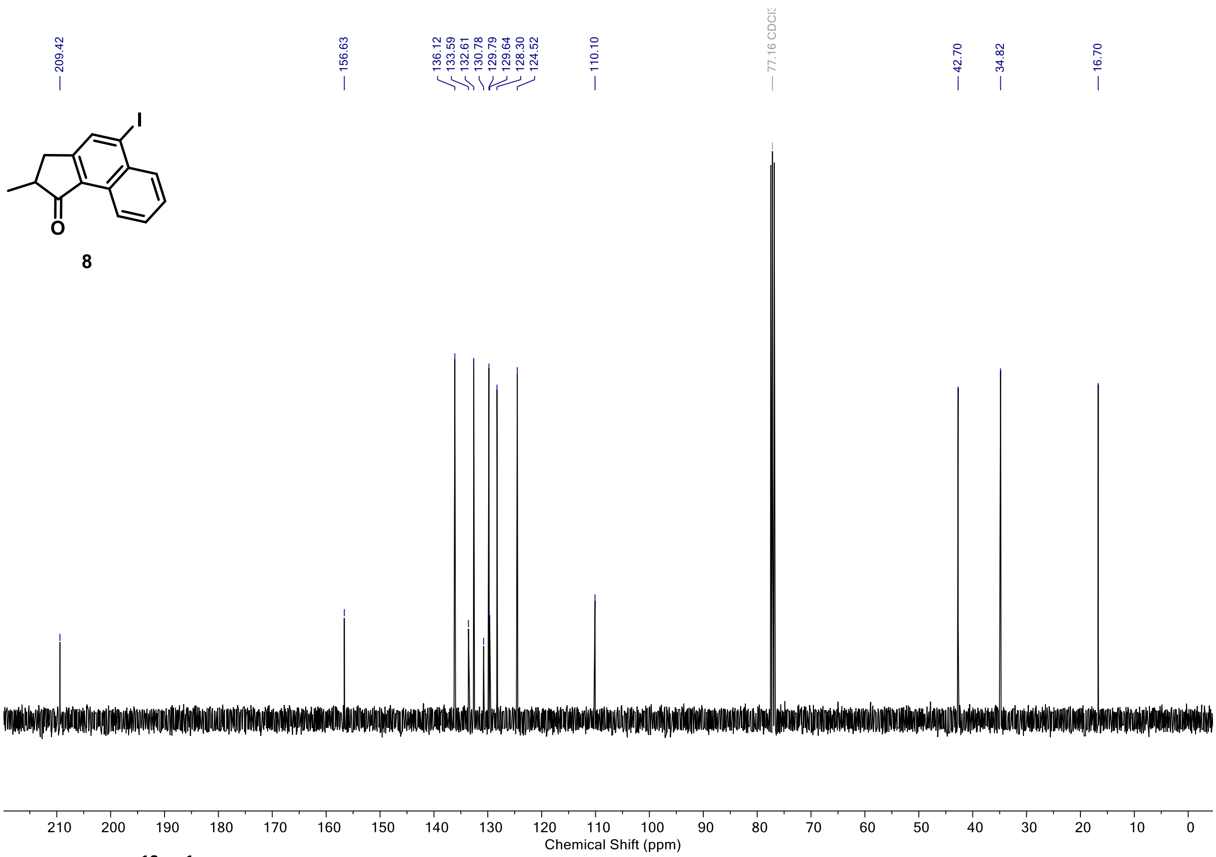

Figure S27: $^{13}C\{^{1}H\}$ NMR spectrum of **8** (101 MHz, $CDCl_3$).

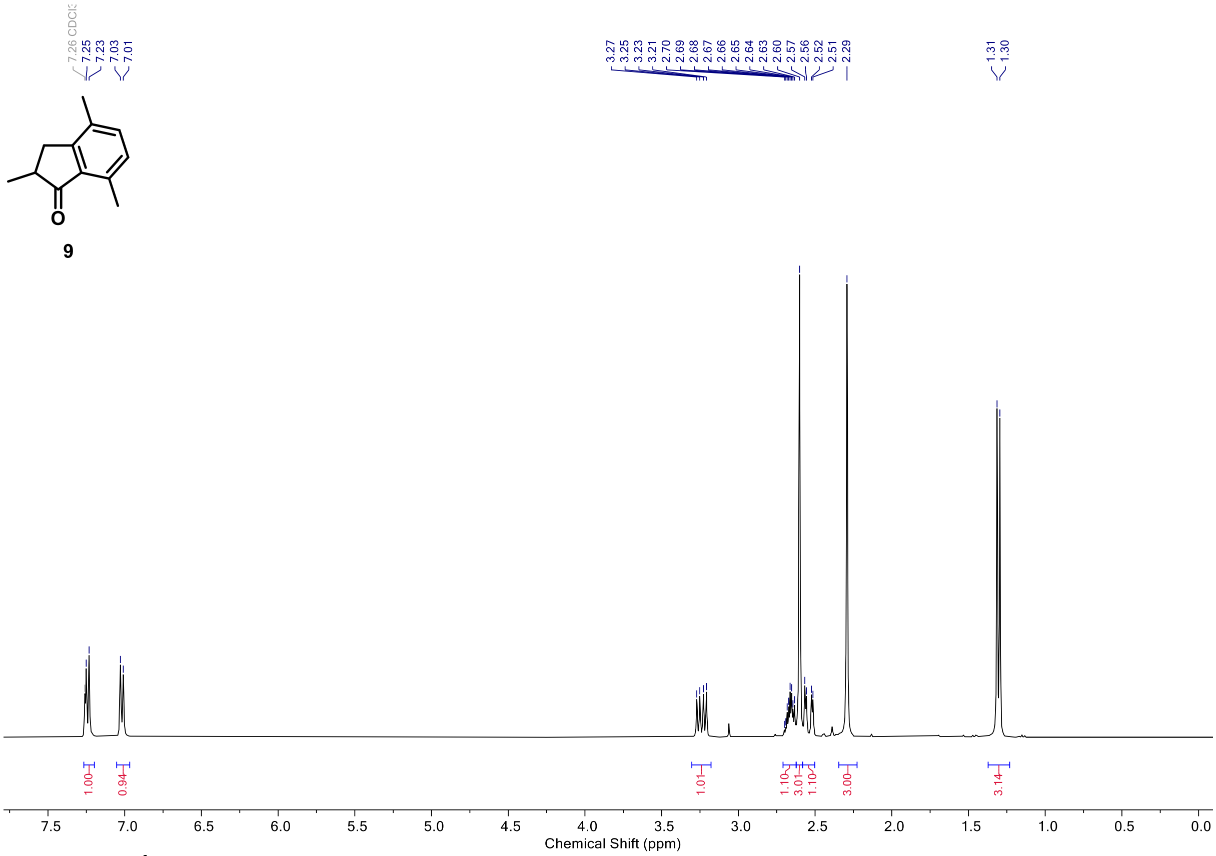

Figure S28: ^{1}H NMR spectrum of **9** (400 MHz, $CDCl_3$).

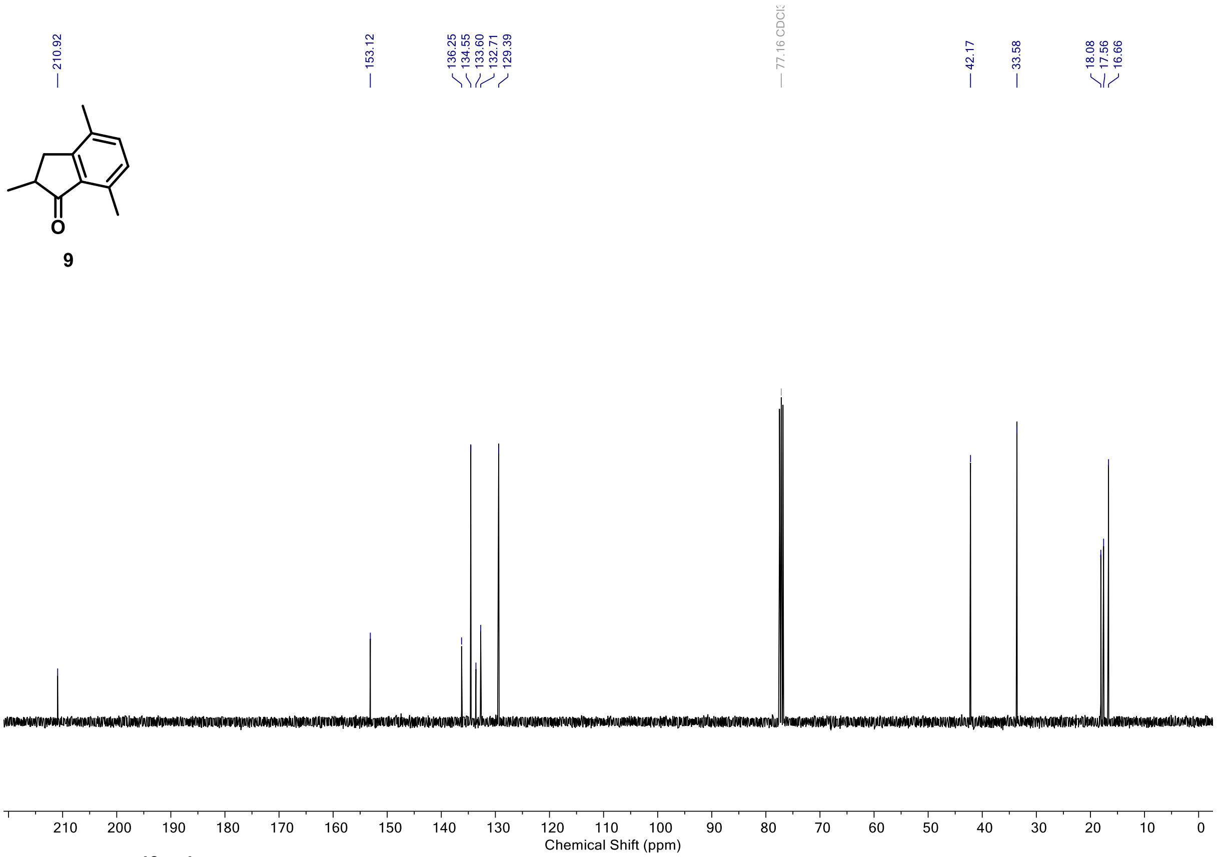

Figure S29: $^{13}C\{^{1}H\}$ NMR spectrum of **9** (101 MHz, $CDCl_3$).

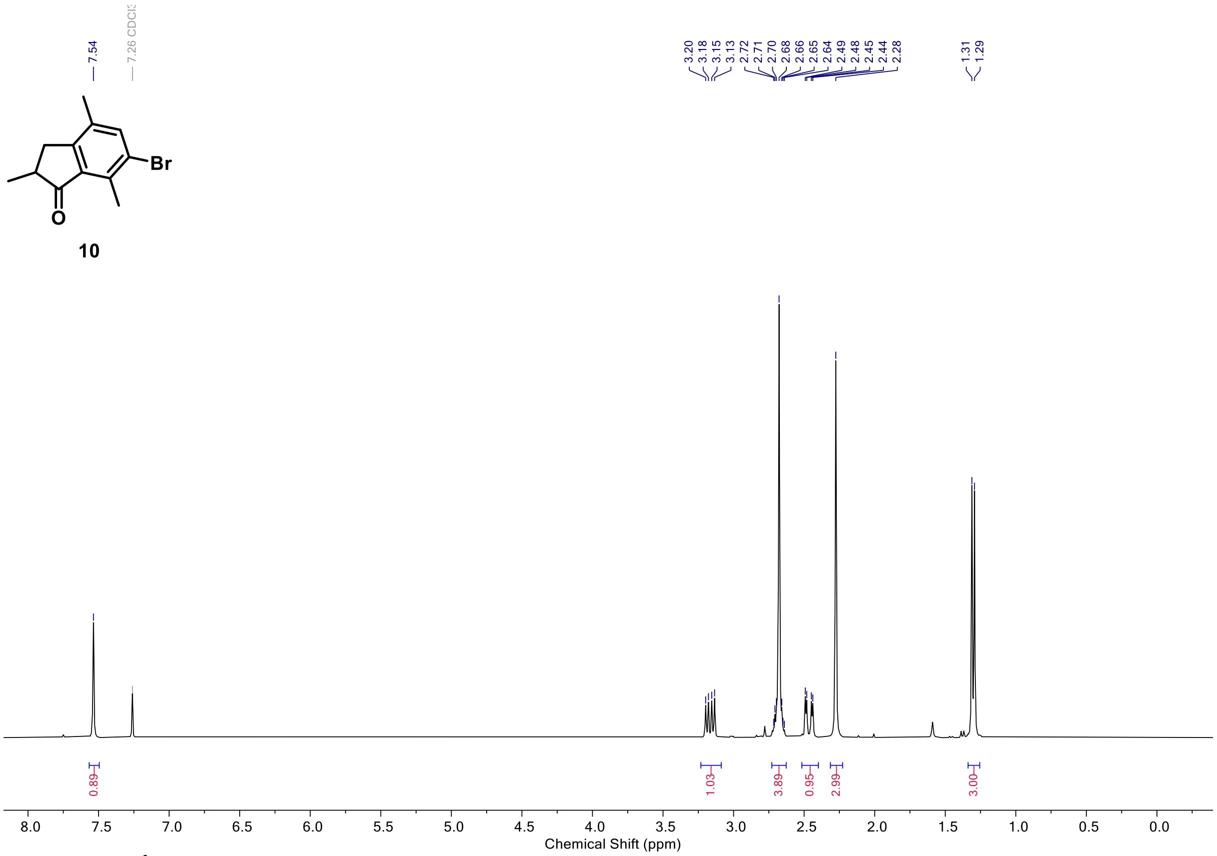

Figure S30: ^{1}H NMR spectrum of **10** (400 MHz, $CDCl_3$).

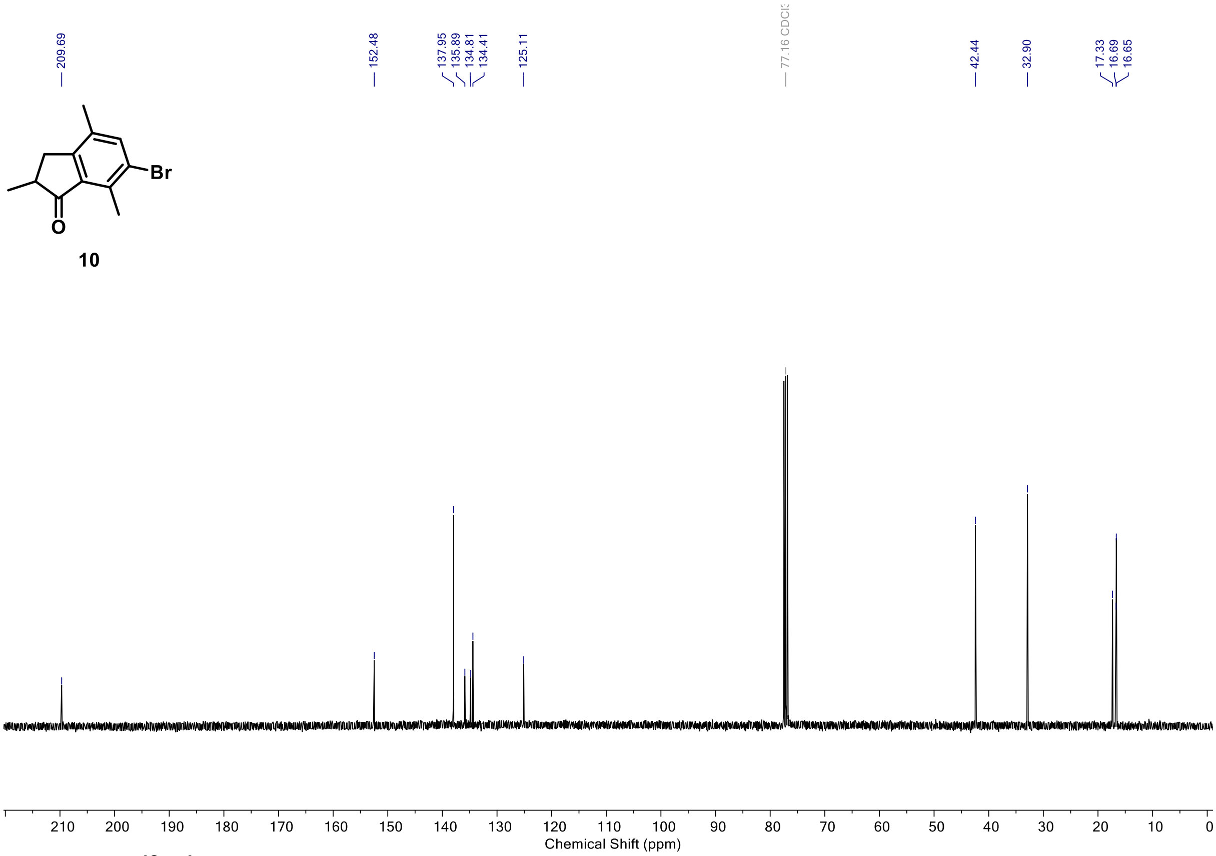

Figure S31: $^{13}C\{^{1}H\}$ **NMR** spectrum of **10** (101 MHz, $CDCl_3$).

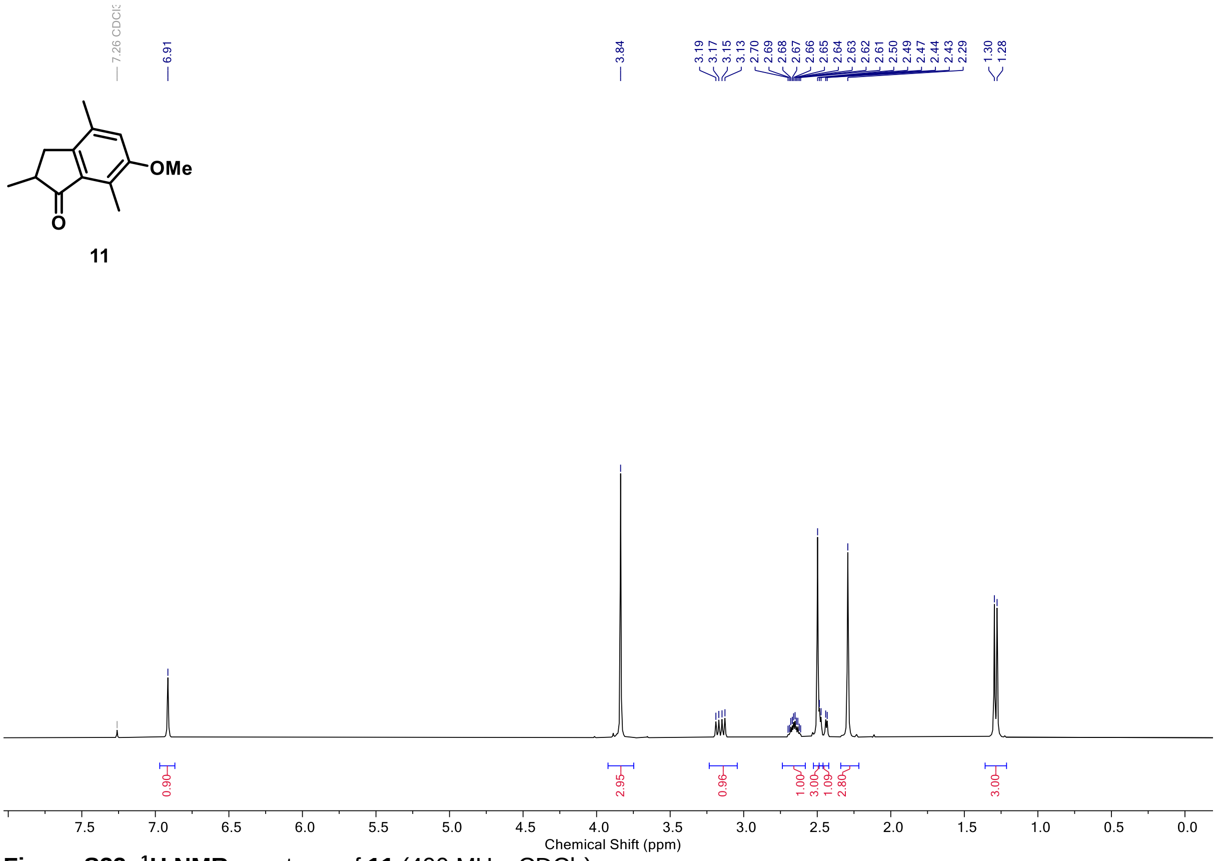

Figure S32: ^{1}**H NMR** spectrum of **11** (400 MHz, $CDCl_3$).

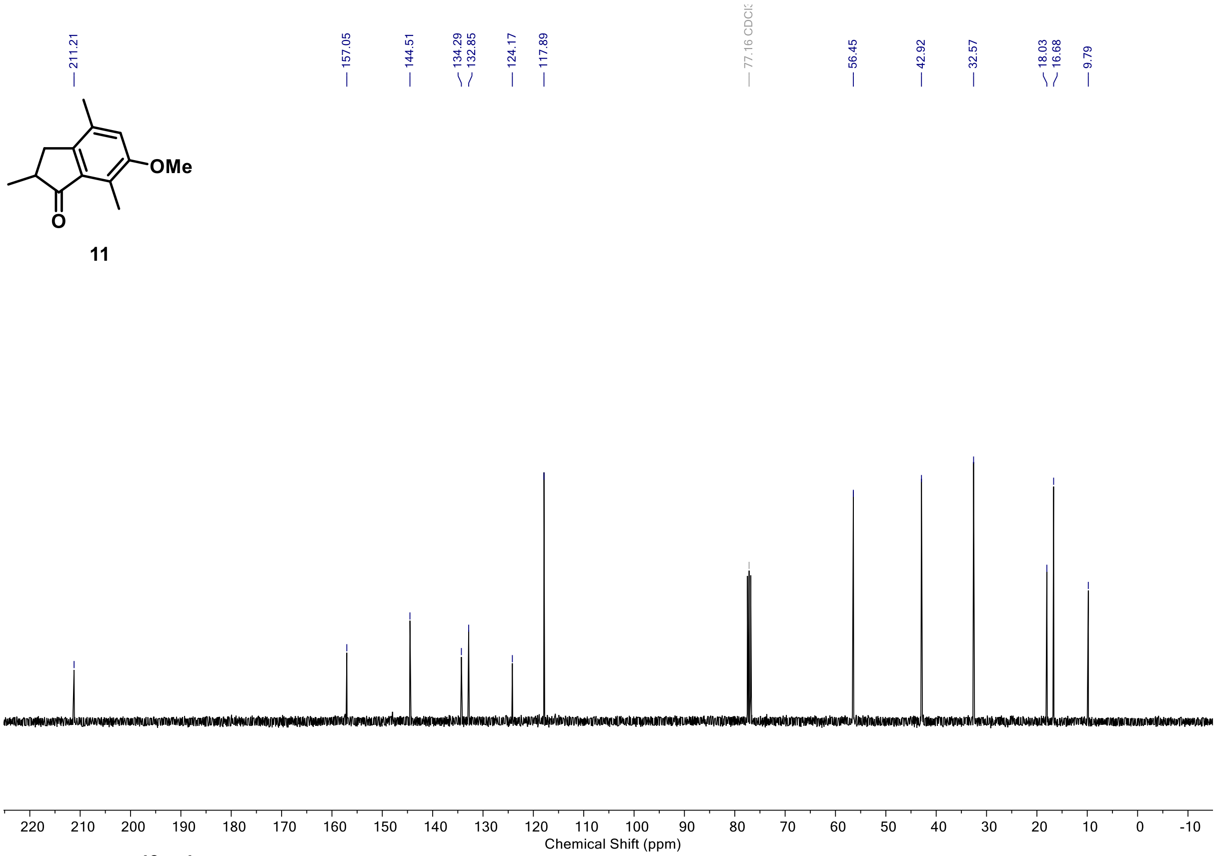

Figure S33: ^{13}C{^{1}H} NMR spectrum of **11** (101 MHz, CDCl$_3$).

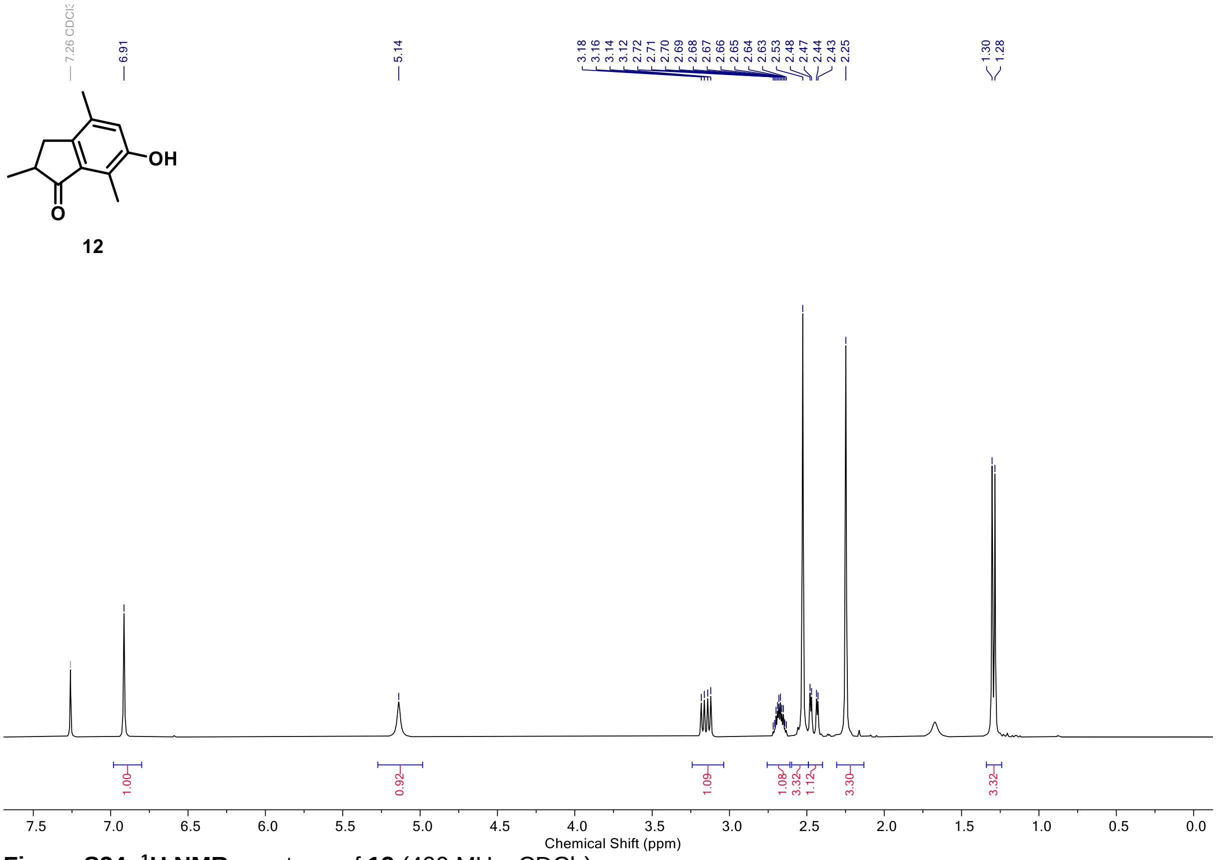

Figure S34: ^{1}H NMR spectrum of **12** (400 MHz, CDCl$_3$).

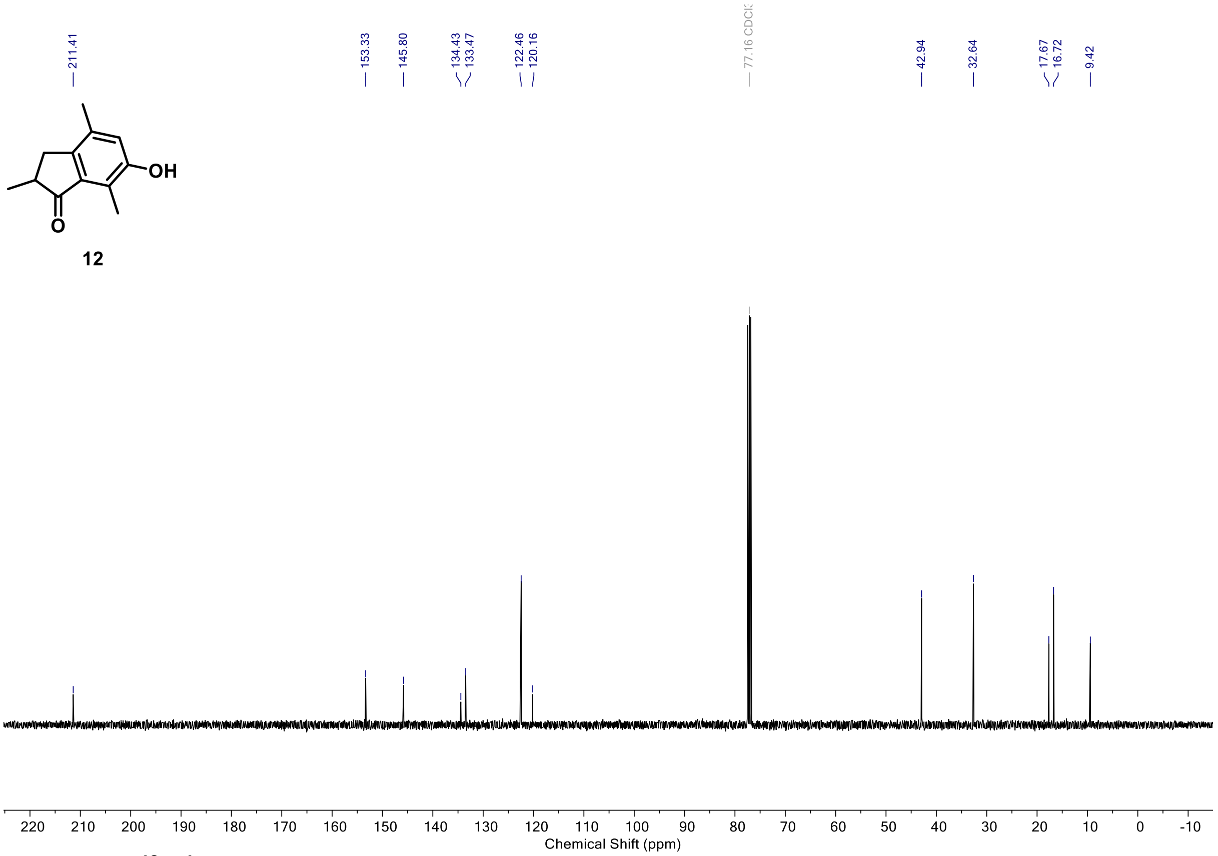

Figure S35: $^{13}C\{^{1}H\}$ NMR spectrum of **12** (101 MHz, $CDCl_3$).

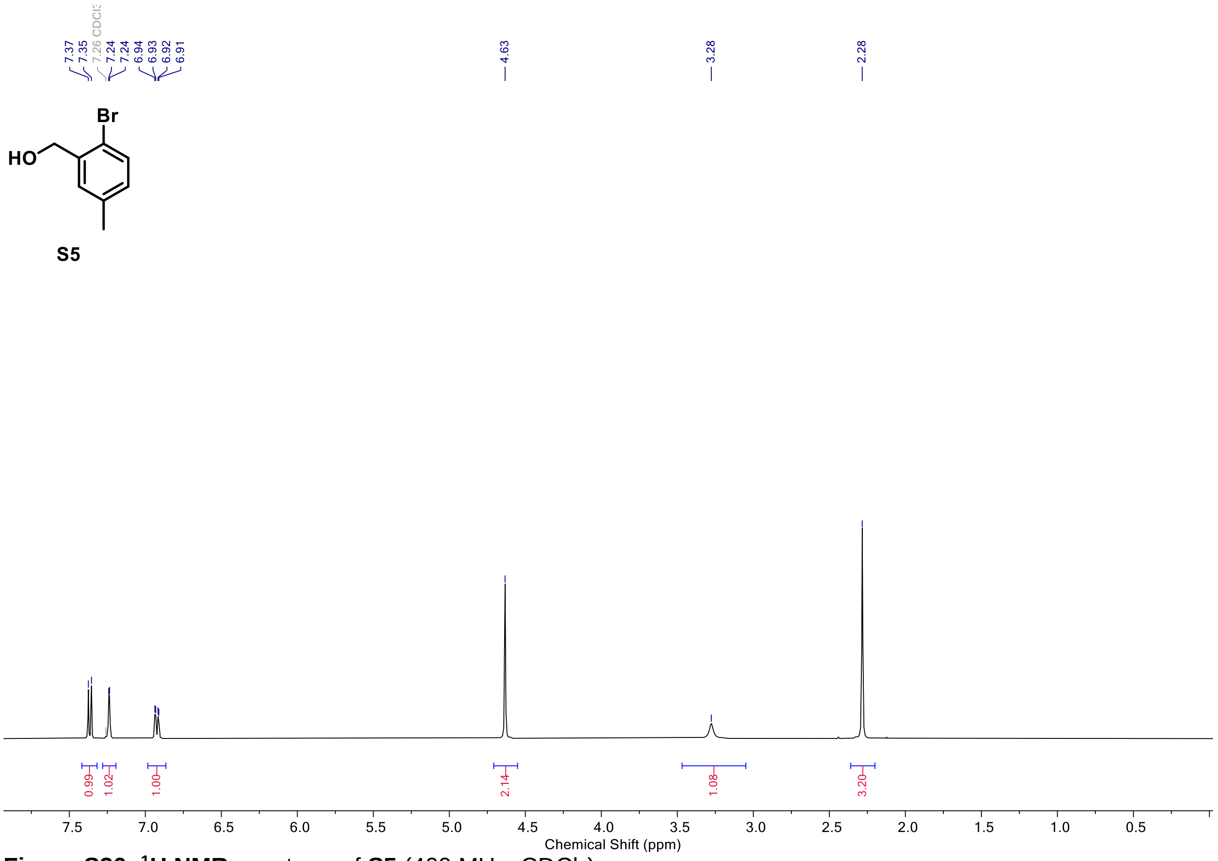

Figure S36: ^{1}H NMR spectrum of **S5** (400 MHz, $CDCl_3$).

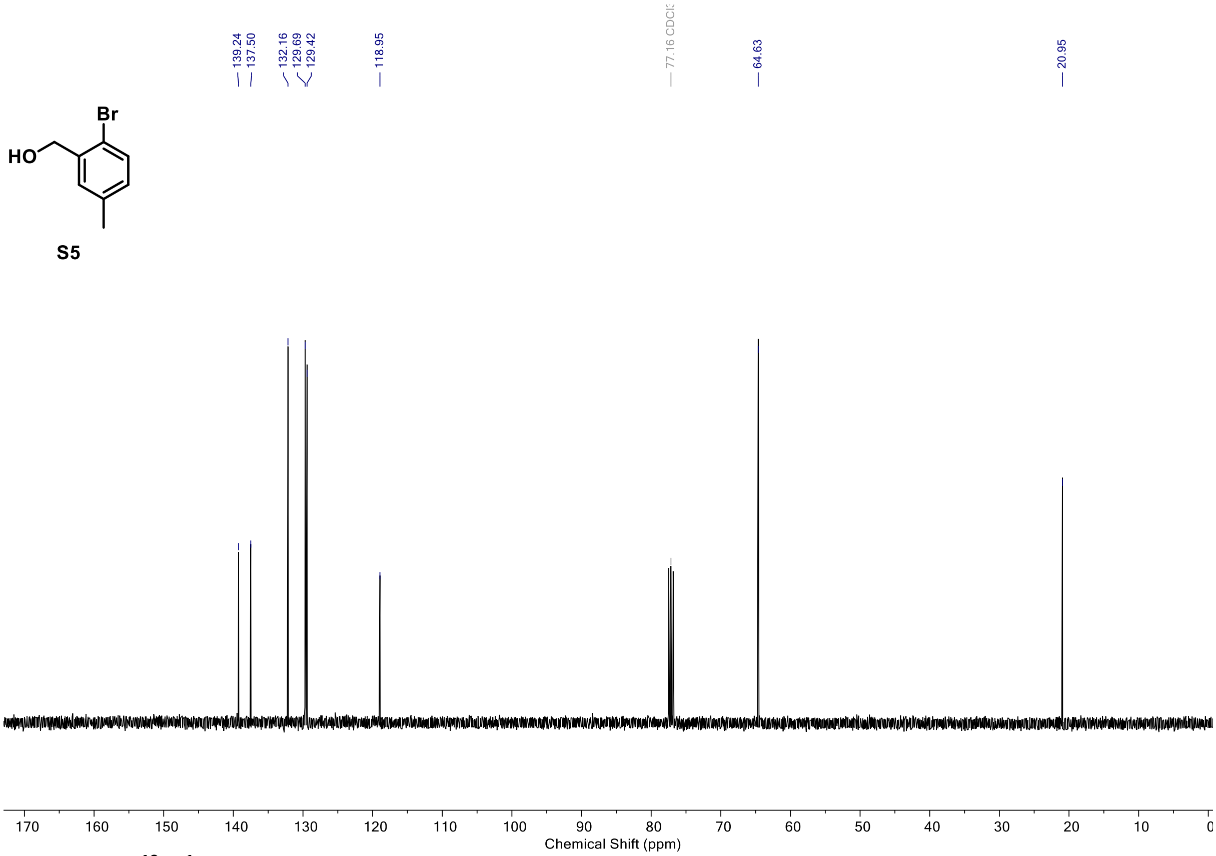

Figure S37: ^{13}C{^{1}H} NMR spectrum of **S5** (101 MHz, $CDCl_3$).

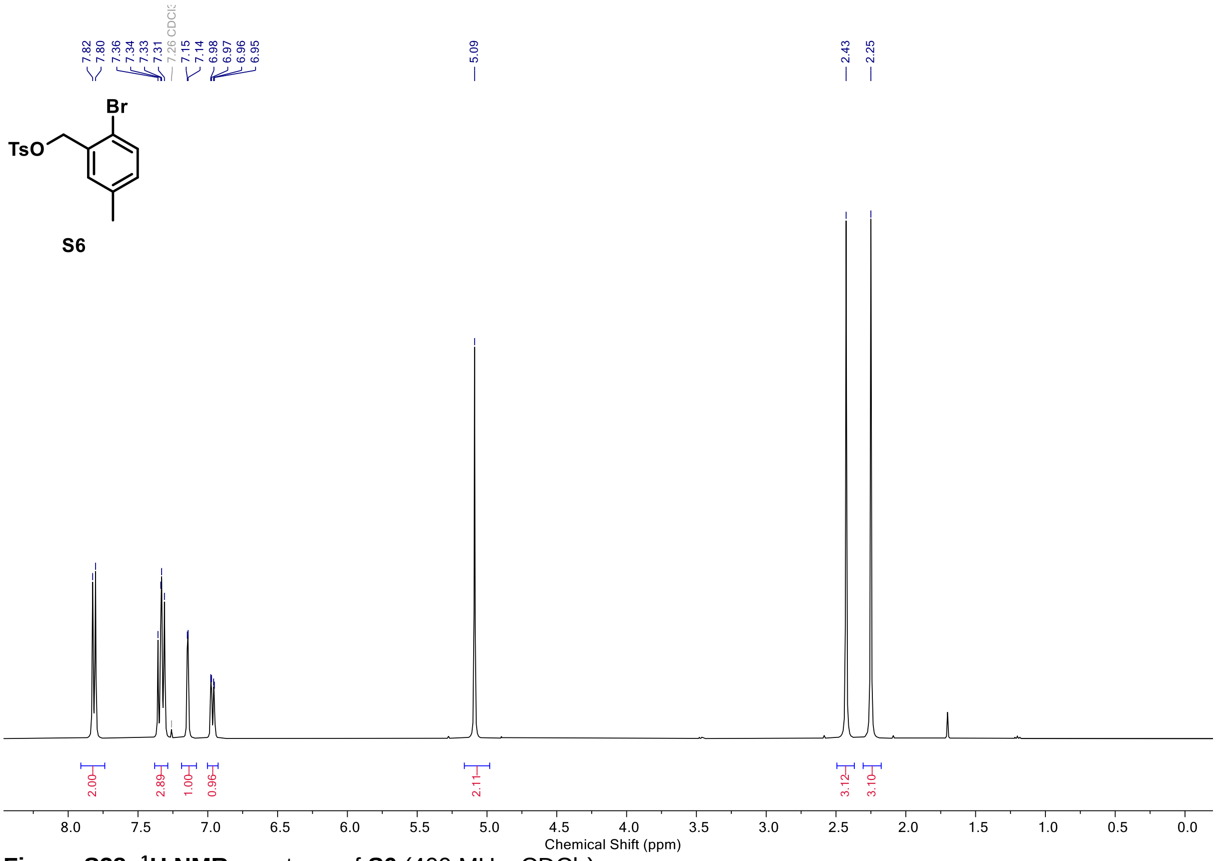

Figure S38: ^{1}H NMR spectrum of **S6** (400 MHz, $CDCl_3$).

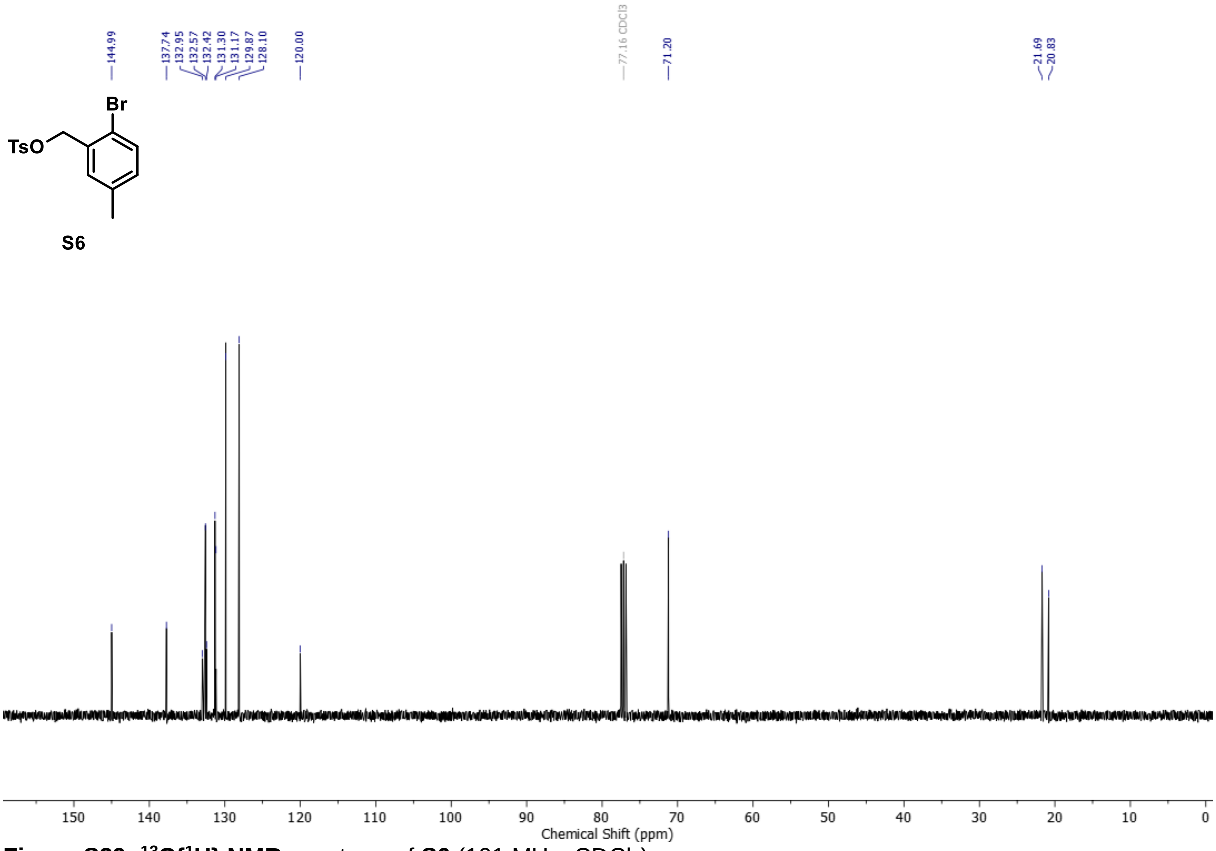

Figure S39: **^{13}C{^{1}H} NMR** spectrum of **S6** (101 MHz, $CDCl_3$).

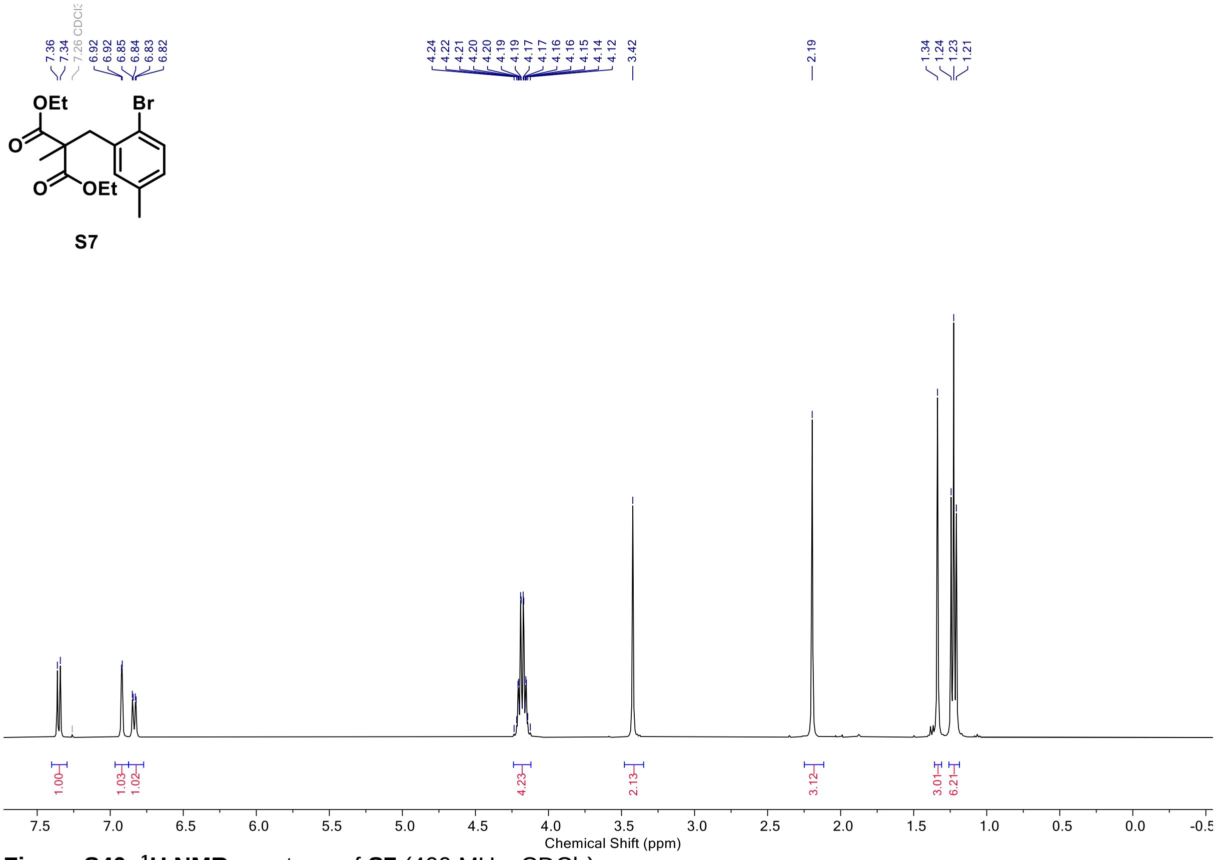

Figure S40: **^{1}H NMR** spectrum of **S7** (400 MHz, $CDCl_3$).

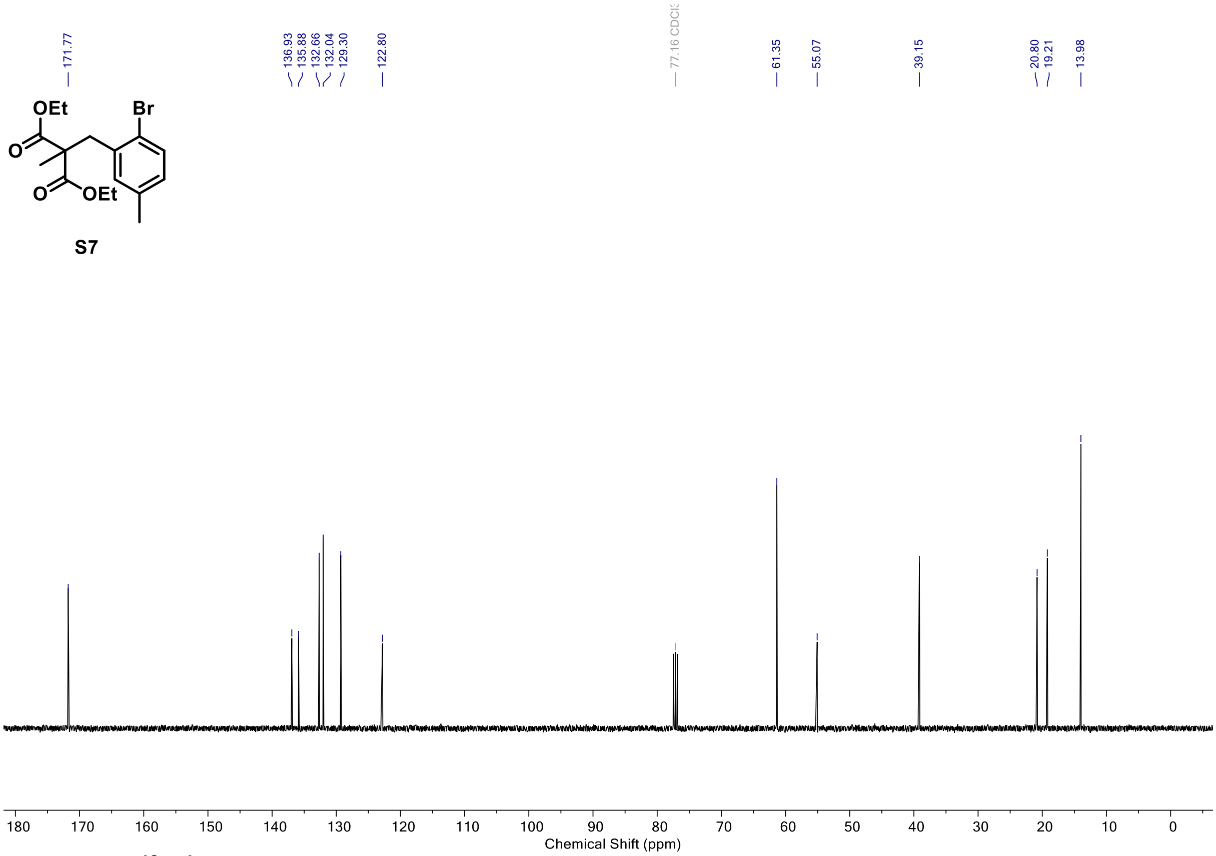

Figure S41: ^{13}**C{**^{1}**H} NMR** spectrum of **S7** (101 MHz, $CDCl_3$).

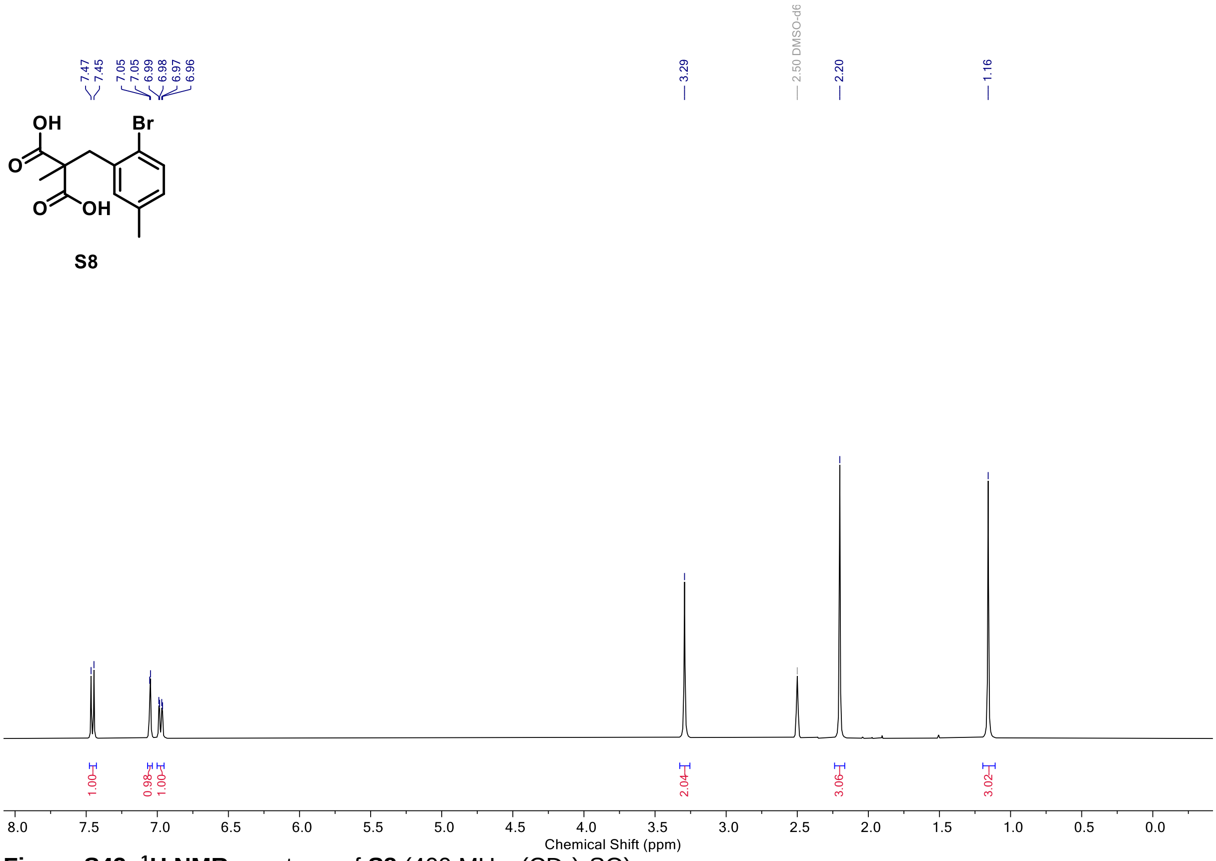

Figure S42: ^{1}**H NMR** spectrum of **S8** (400 MHz, $(CD_3)_2SO$).

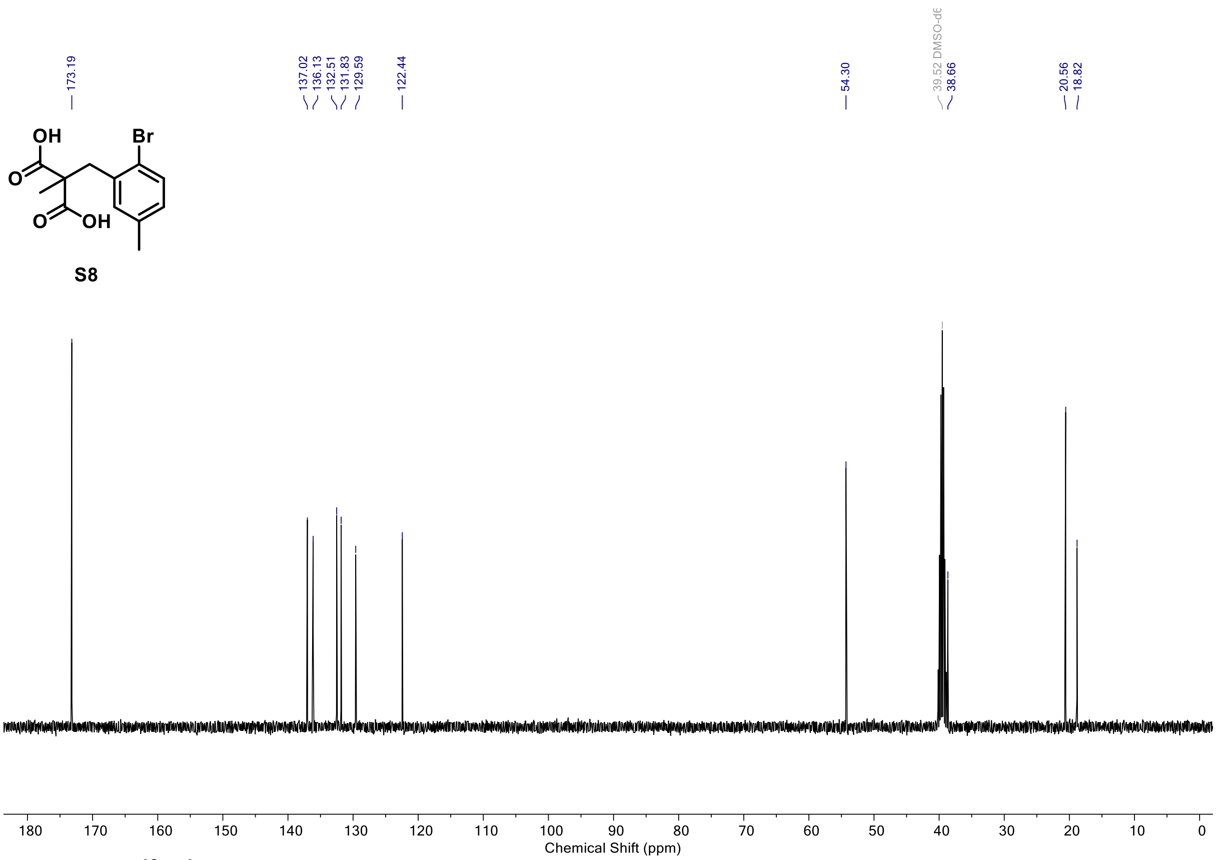

Figure S43: ^{13}**C{**^{1}**H} NMR** spectrum of **S8** (101 MHz, $(CD_3)_2SO$).

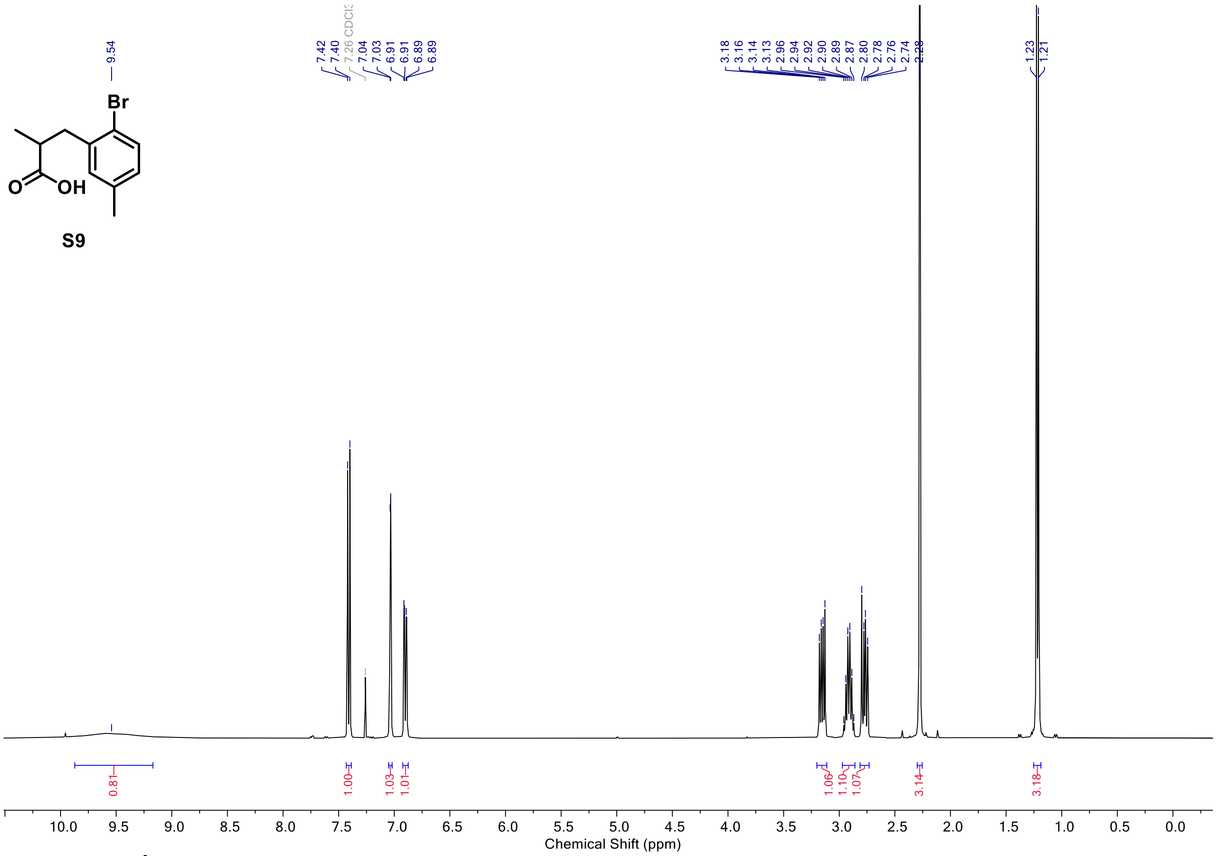

Figure S44: ^{1}**H NMR** spectrum of **S9** (400 MHz, $CDCl_3$).

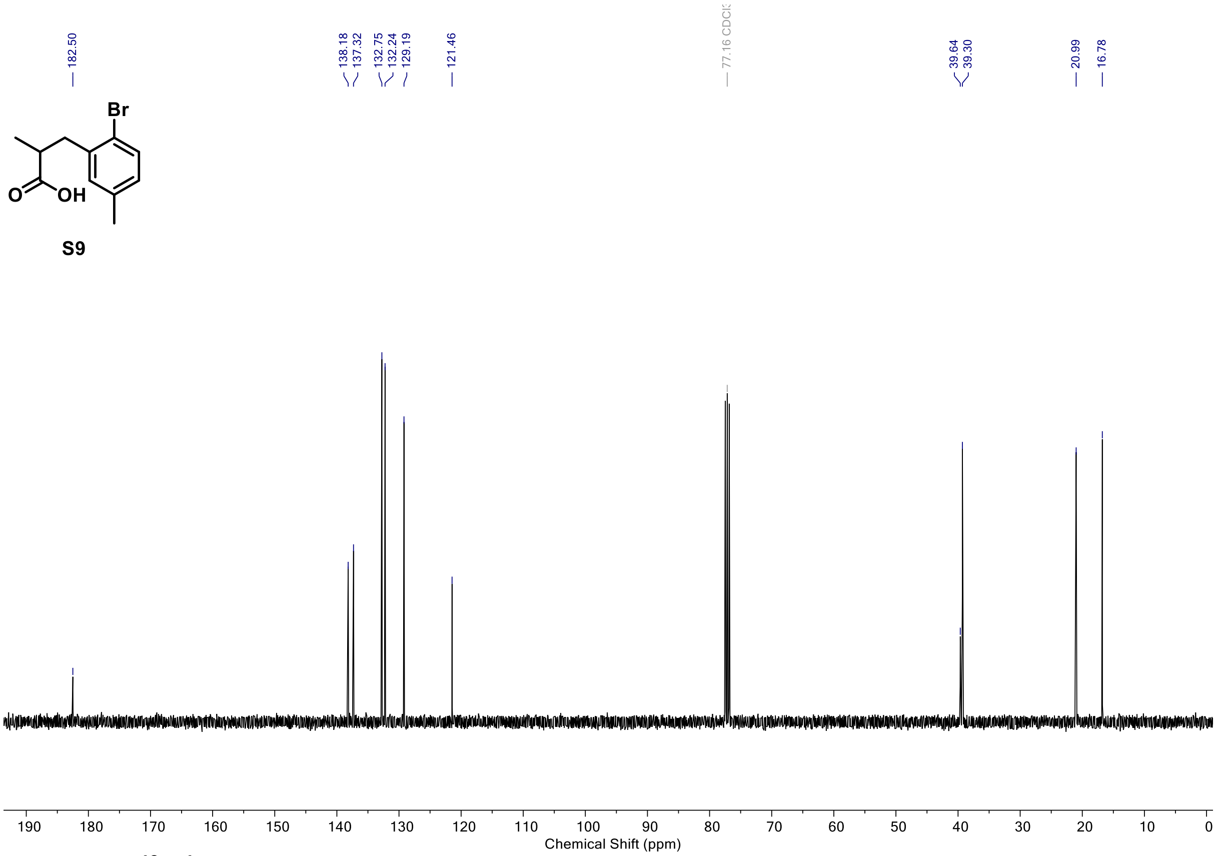

Figure S45: ^{13}**C{**^{1}**H} NMR** spectrum of **S9** (101 MHz, $CDCl_3$).

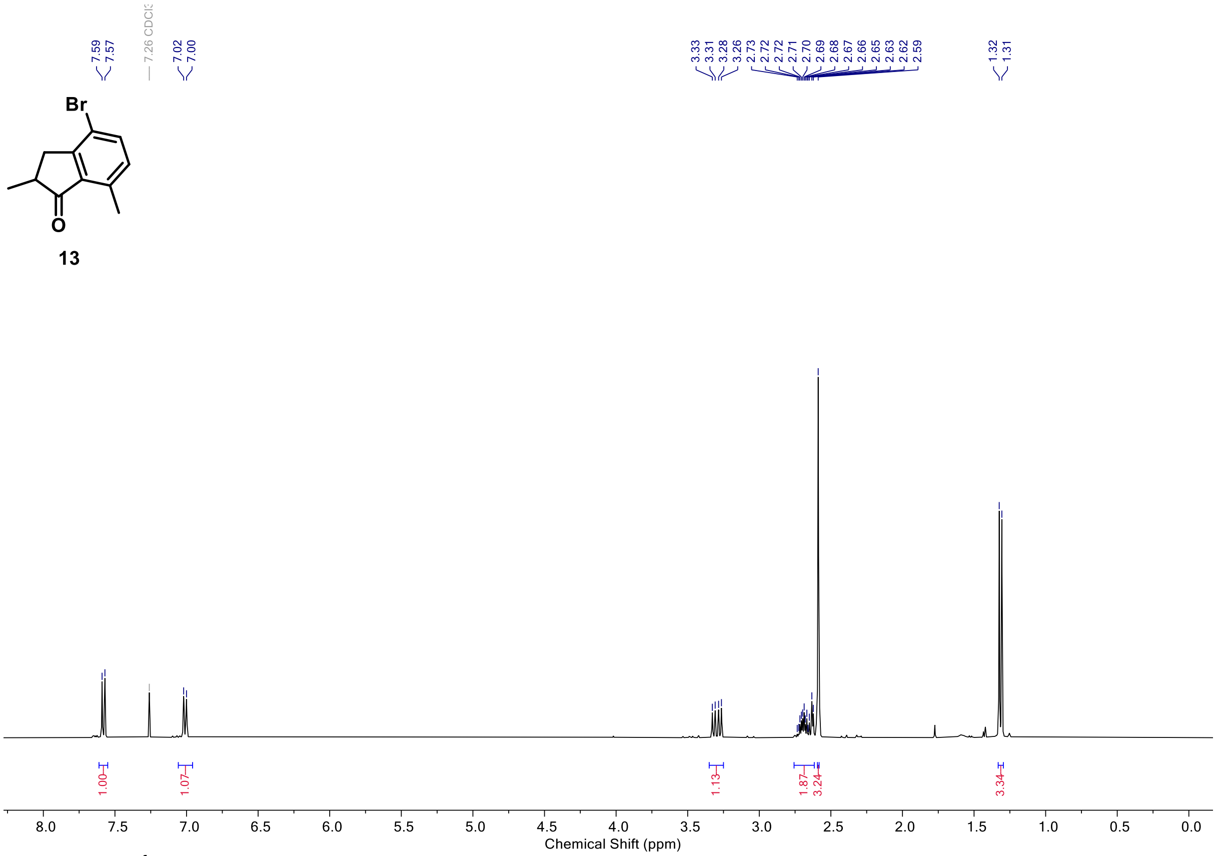

Figure S46: ^{1}**H NMR** spectrum of **13** (400 MHz, $CDCl_3$).

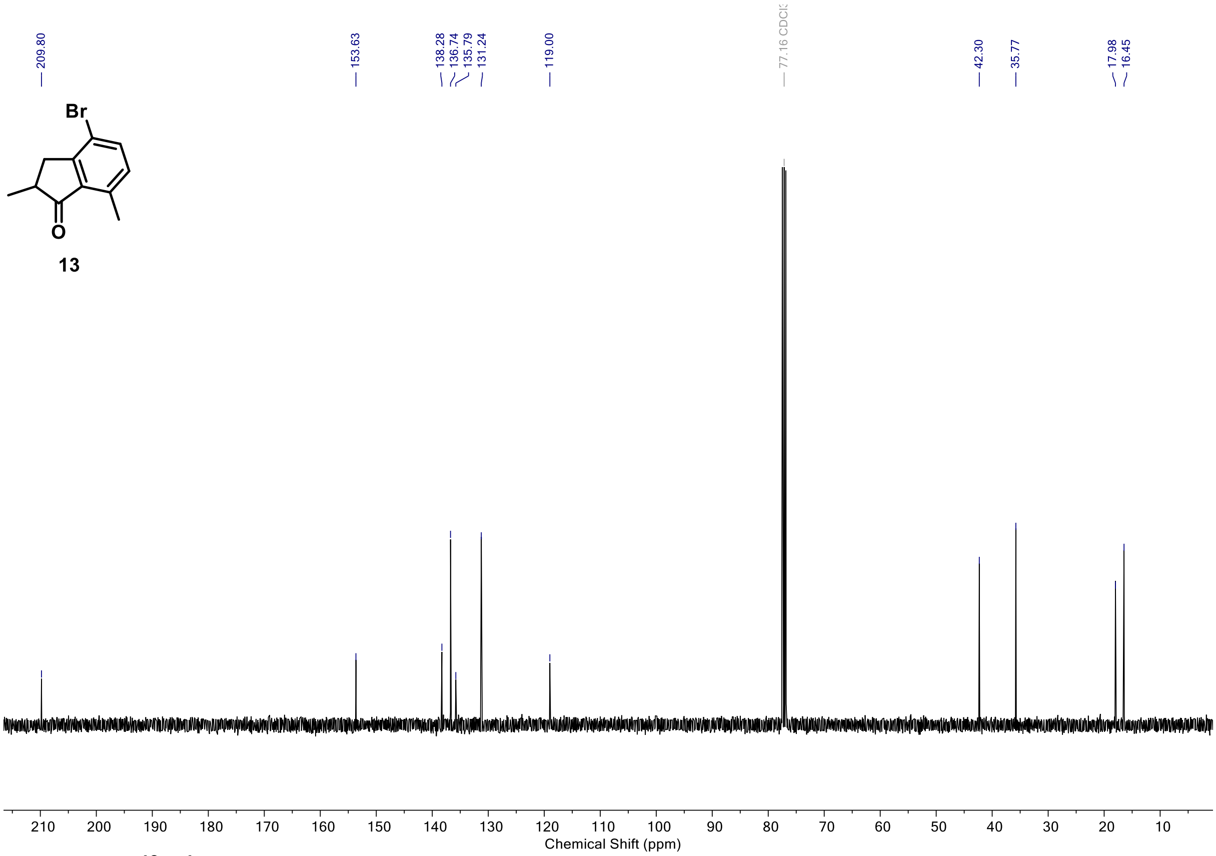

Figure S47: ^{13}**C{^{1}H} NMR** spectrum of **13** (101 MHz, $CDCl_3$).

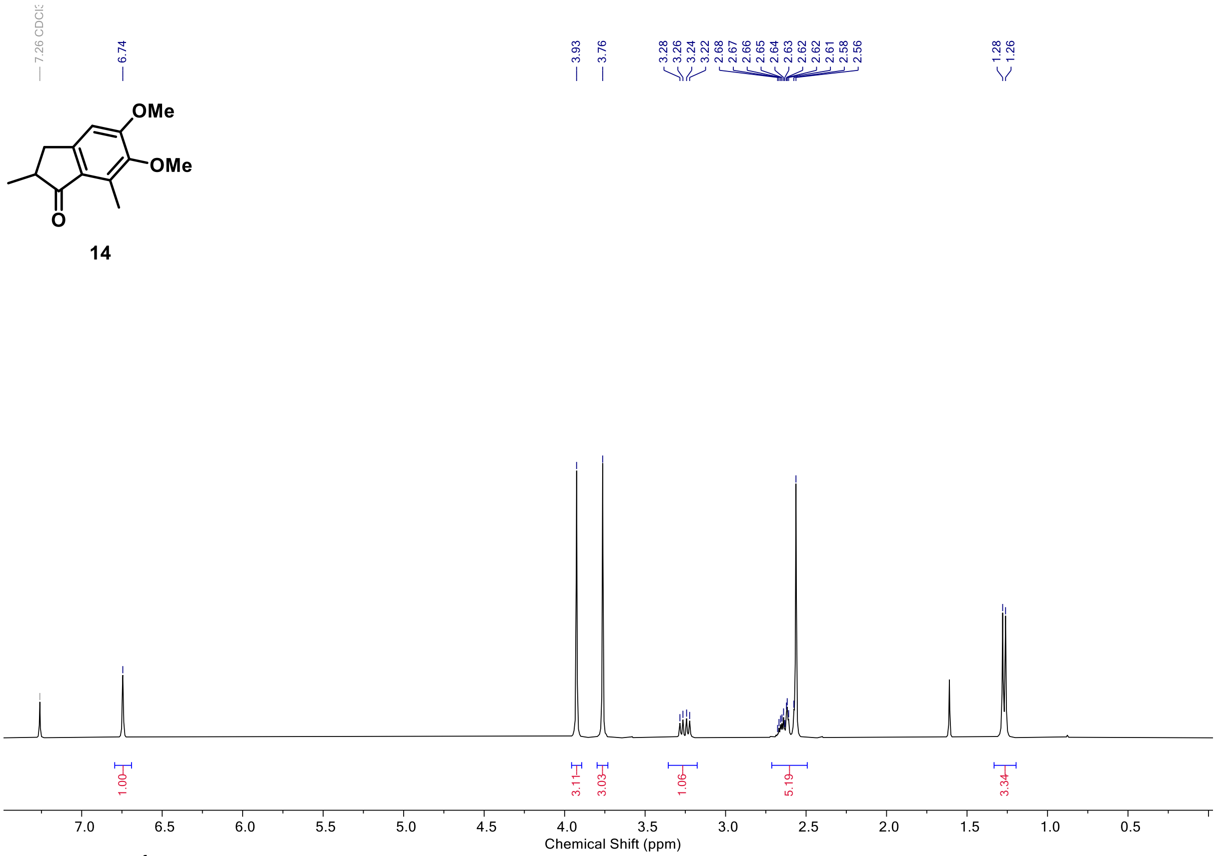

Figure S48: ^{1}**H NMR** spectrum of **14** (400 MHz, $CDCl_3$).

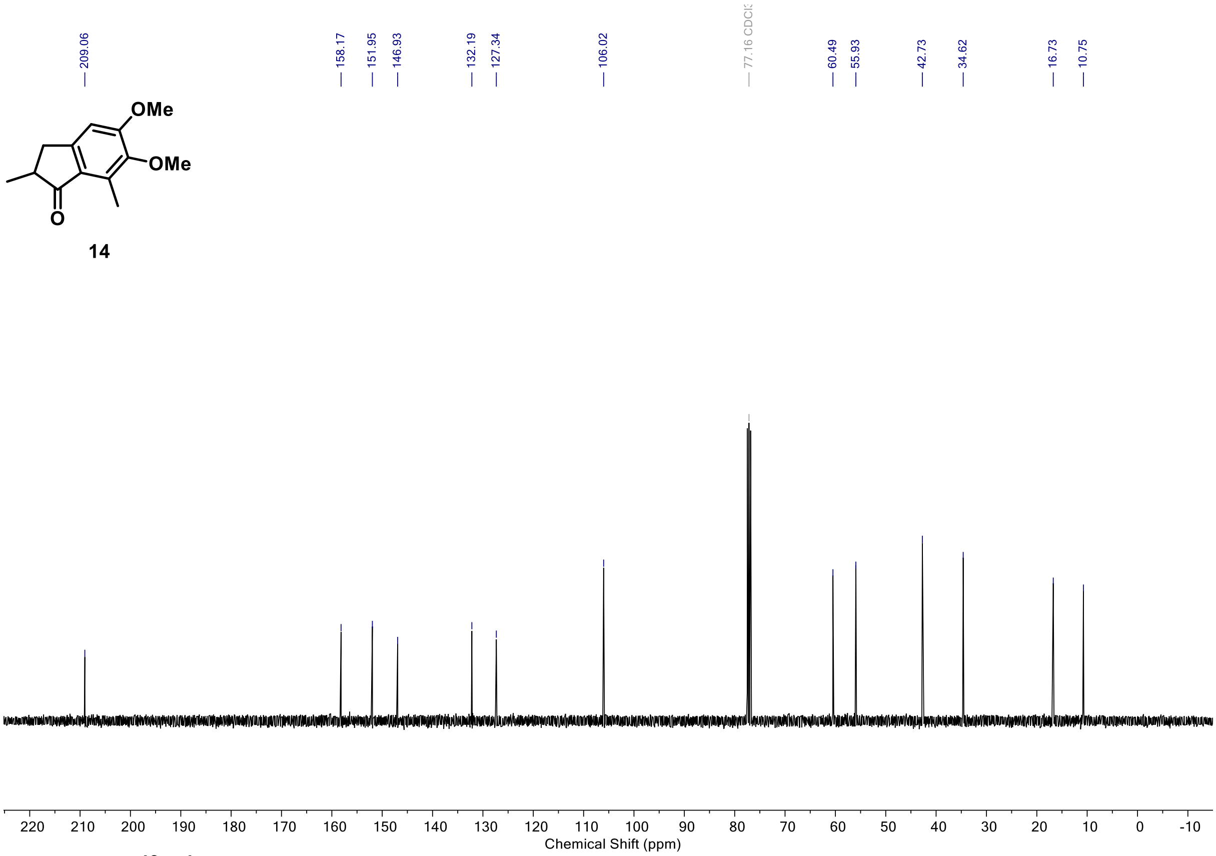

Figure S49: ^{13}C{^{1}H} NMR spectrum of **14** (101 MHz, $CDCl_3$).

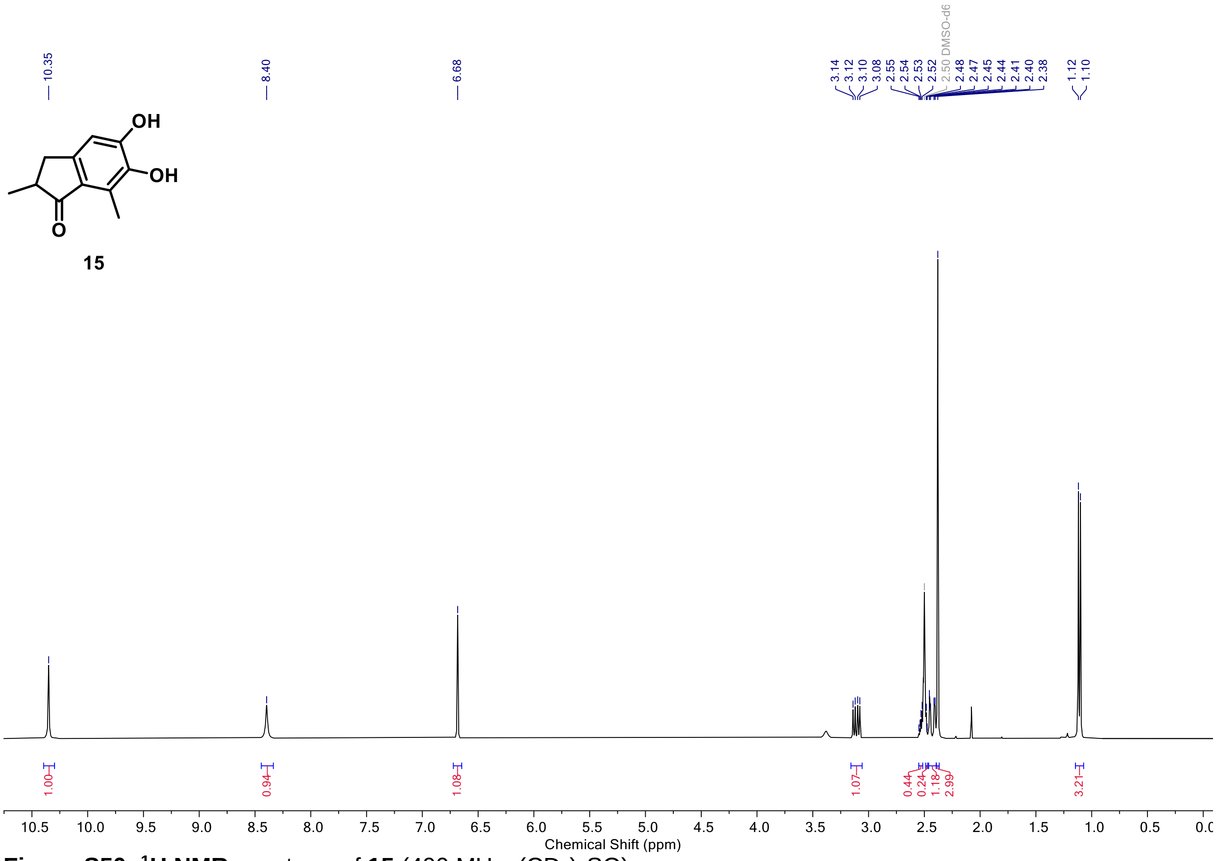

Figure S50: ^{1}H NMR spectrum of **15** (400 MHz, $(CD_3)_2SO$).

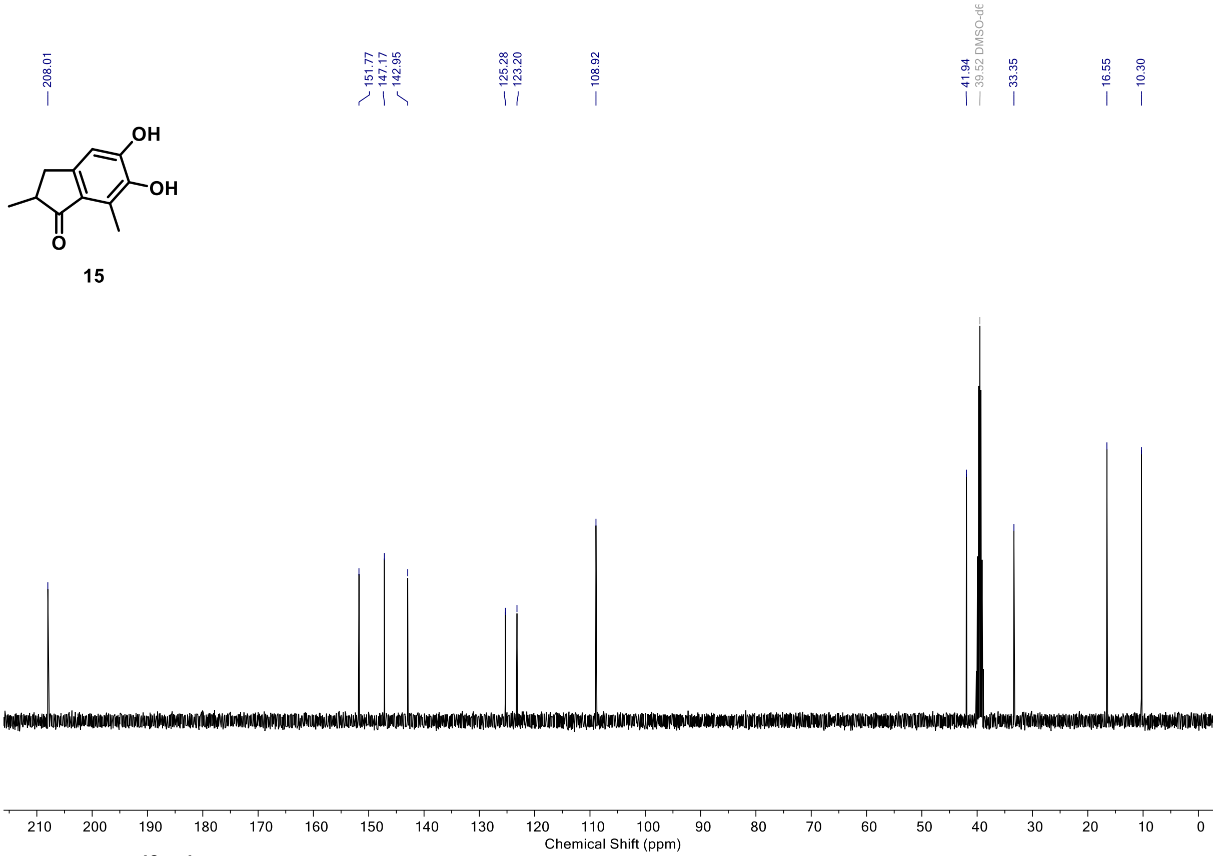

Figure S51: $^{13}C\{^{1}H\}$ NMR spectrum of 15 (101 MHz, $(CD_3)_2SO$).

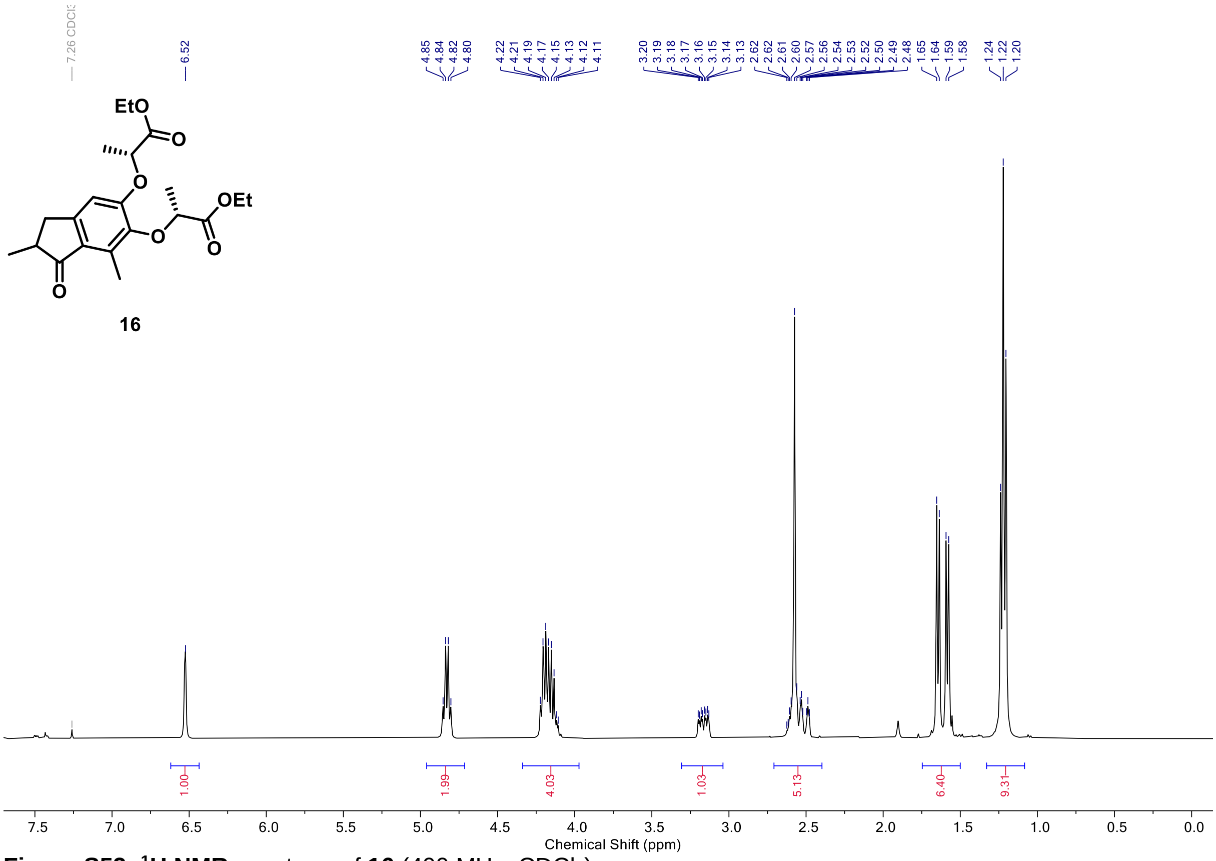

Figure S52: ^{1}H NMR spectrum of 16 (400 MHz, $CDCl_3$).

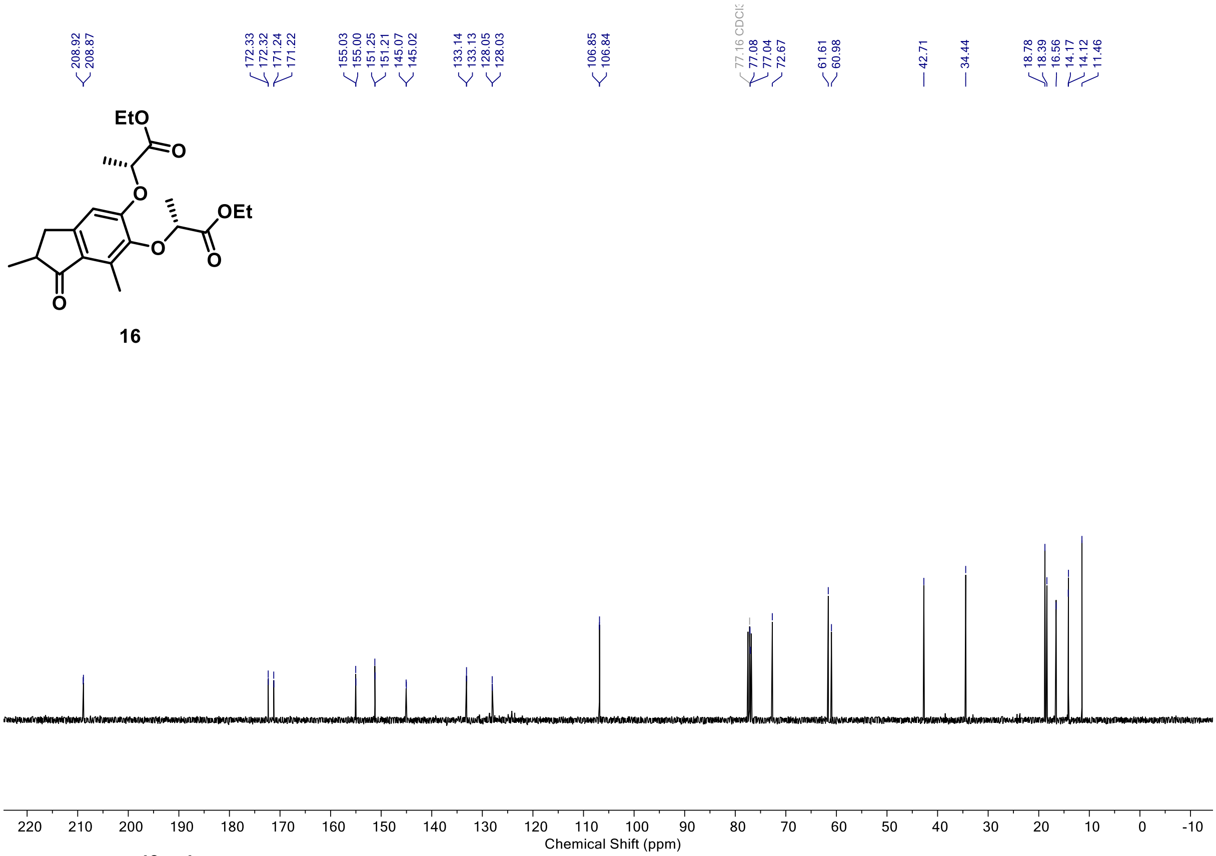

Figure S53: ^{13}**C{**^{1}**H} NMR** spectrum of **16** (101 MHz, $CDCl_3$).

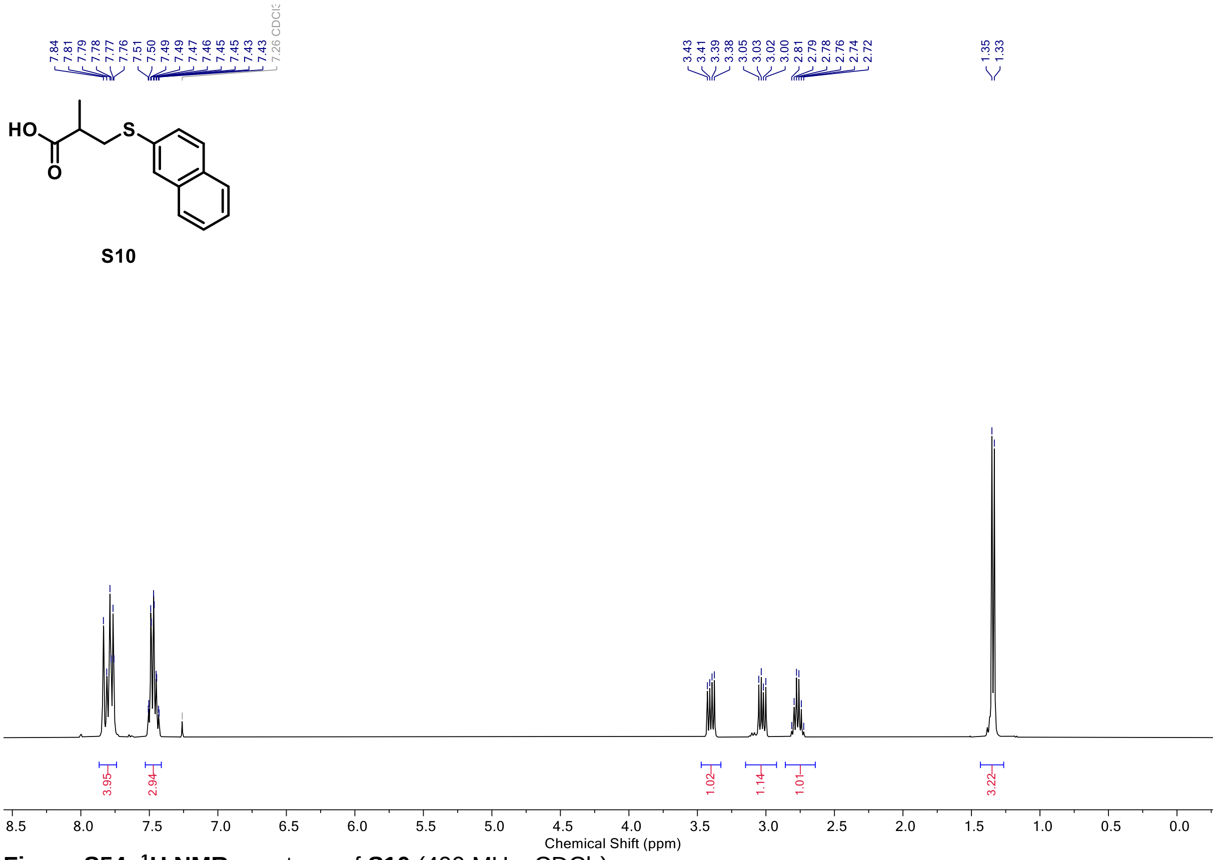

Figure S54: ^{1}**H NMR** spectrum of **S10** (400 MHz, $CDCl_3$).

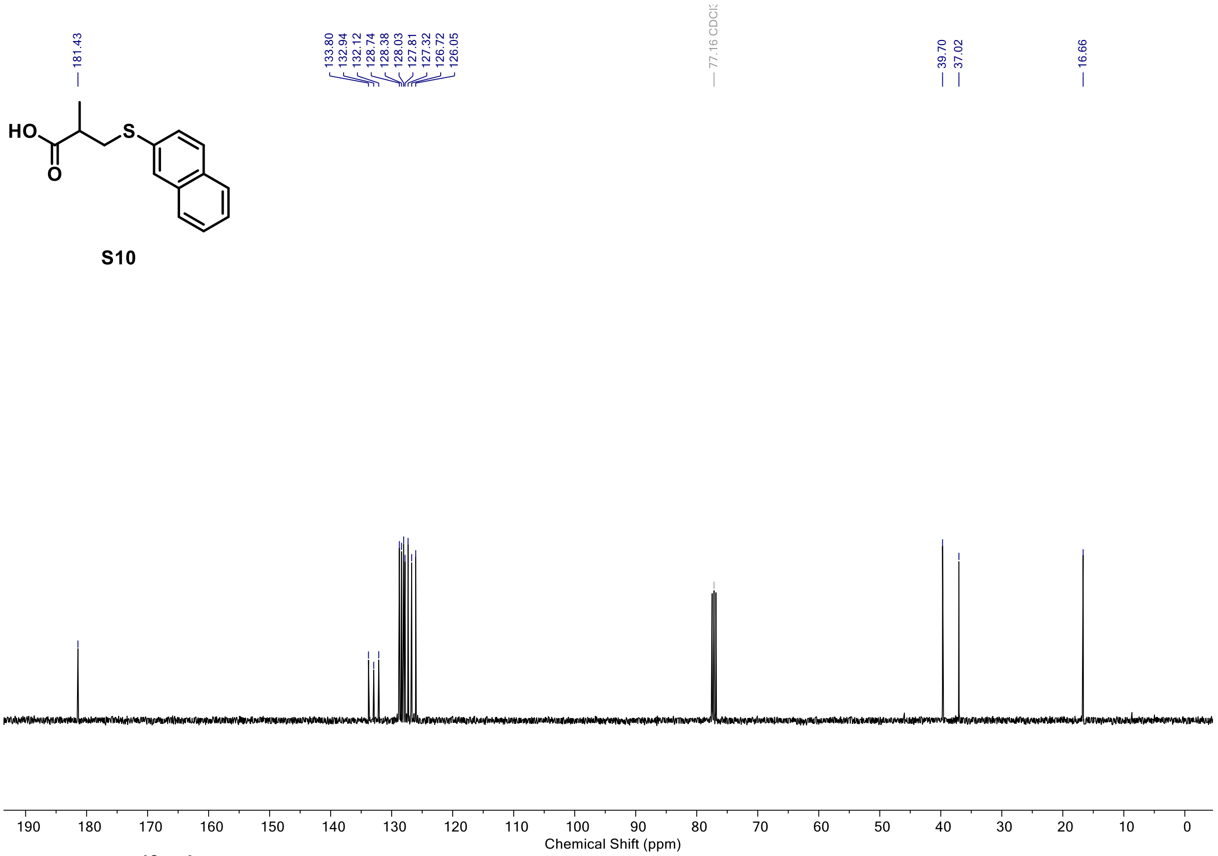

Figure S55: **$^{13}C\{^{1}H\}$ NMR** spectrum of **S10** (101 MHz, $CDCl_3$).

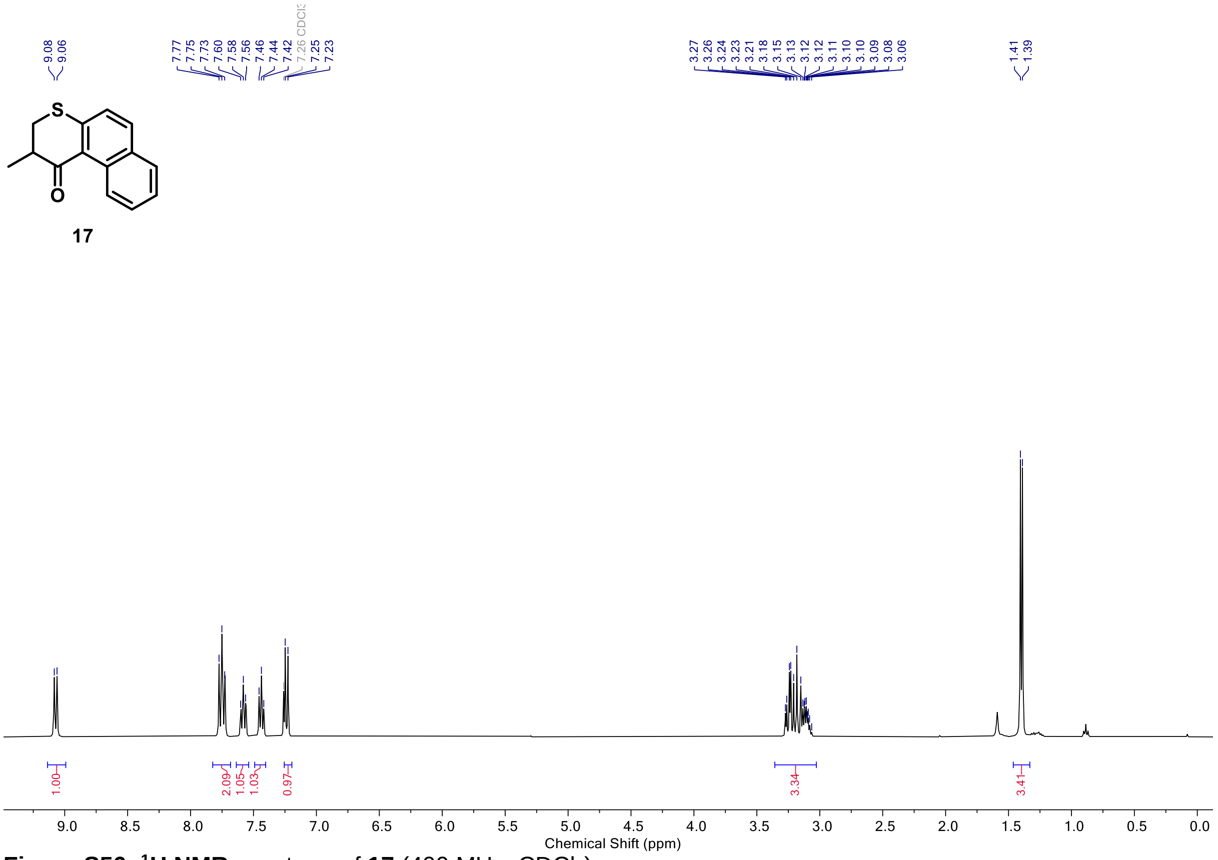

Figure S56: **^{1}H NMR** spectrum of **17** (400 MHz, $CDCl_3$).

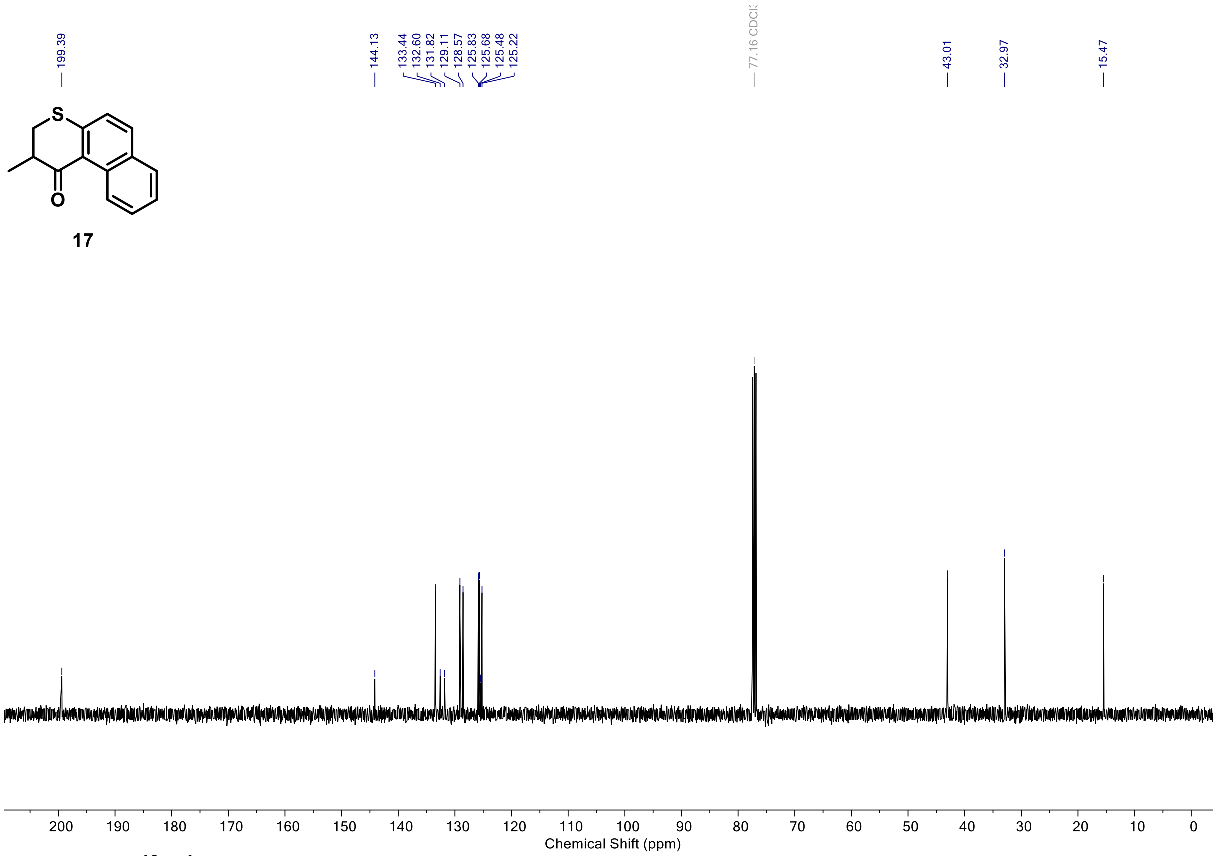

Figure S57: **$^{13}C\{^1H\}$ NMR** spectrum of **17** (101 MHz, $CDCl_3$).

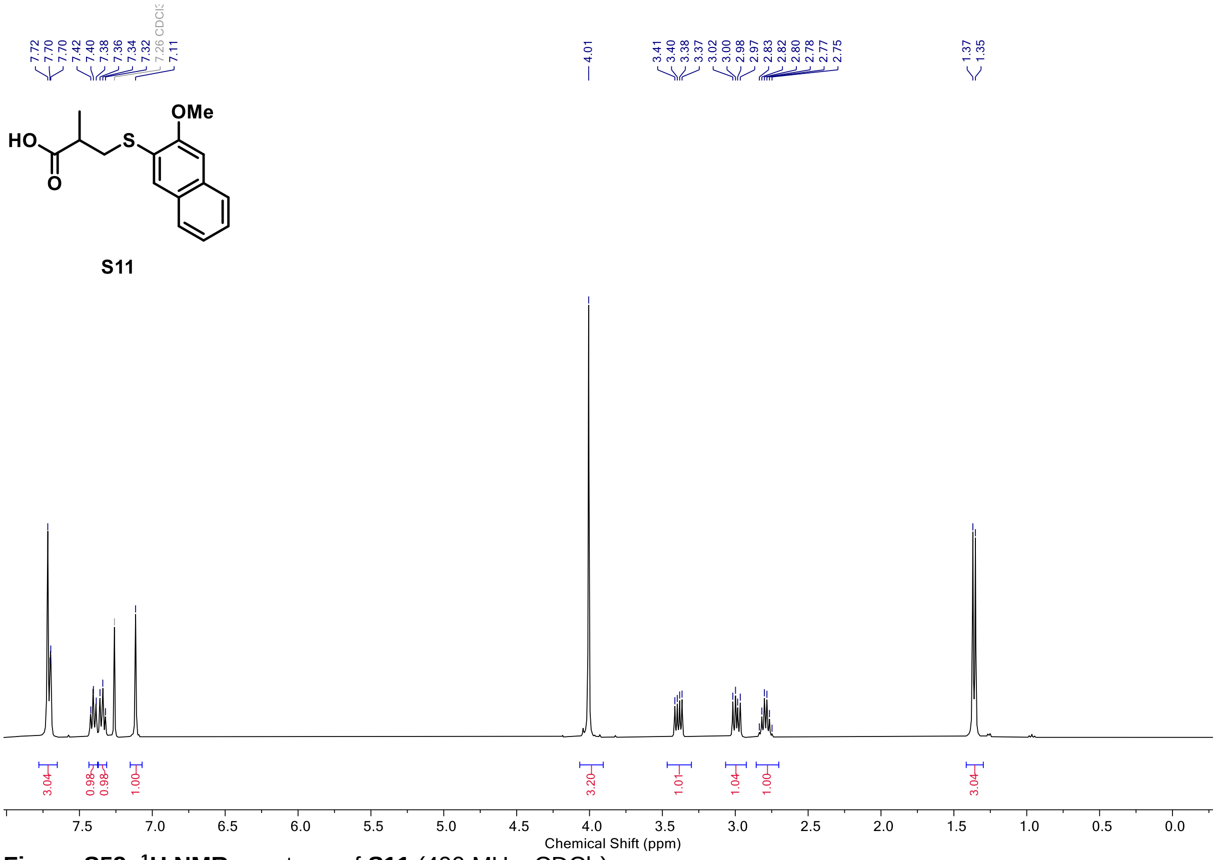

Figure S58: **^1H NMR** spectrum of **S11** (400 MHz, $CDCl_3$).

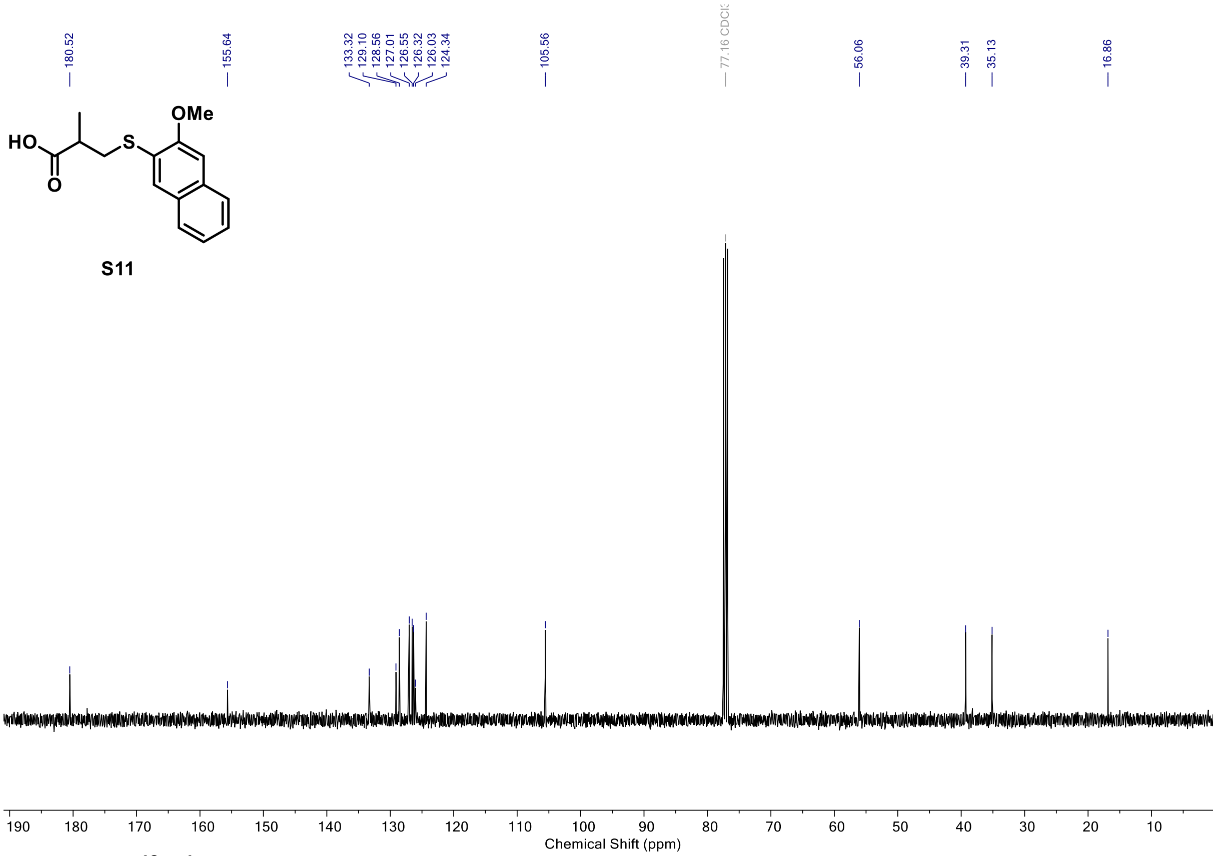

S11

Figure S59: **^{13}C{^{1}H} NMR** spectrum of **S11** (101 MHz, $CDCl_3$).

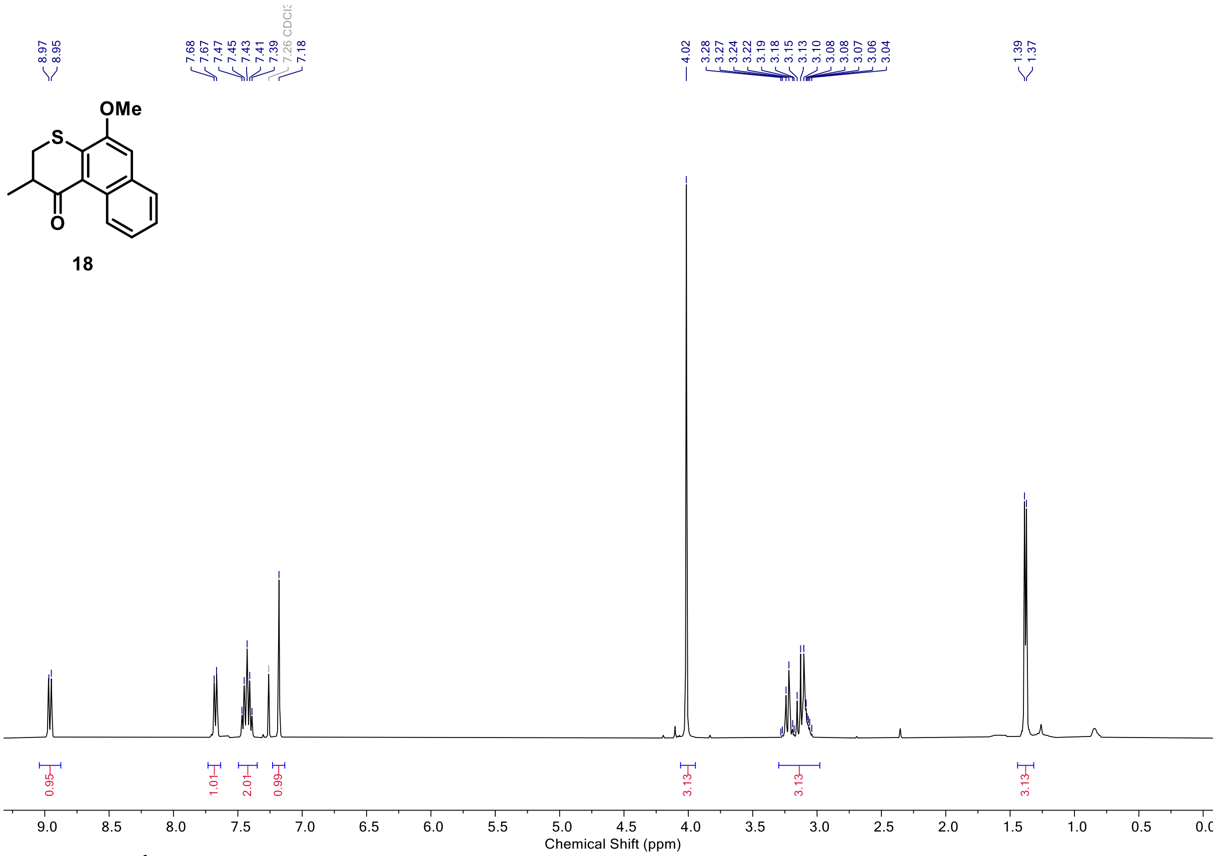

18

Figure S60: **^{1}H NMR** spectrum of **18** (400 MHz, $CDCl_3$).

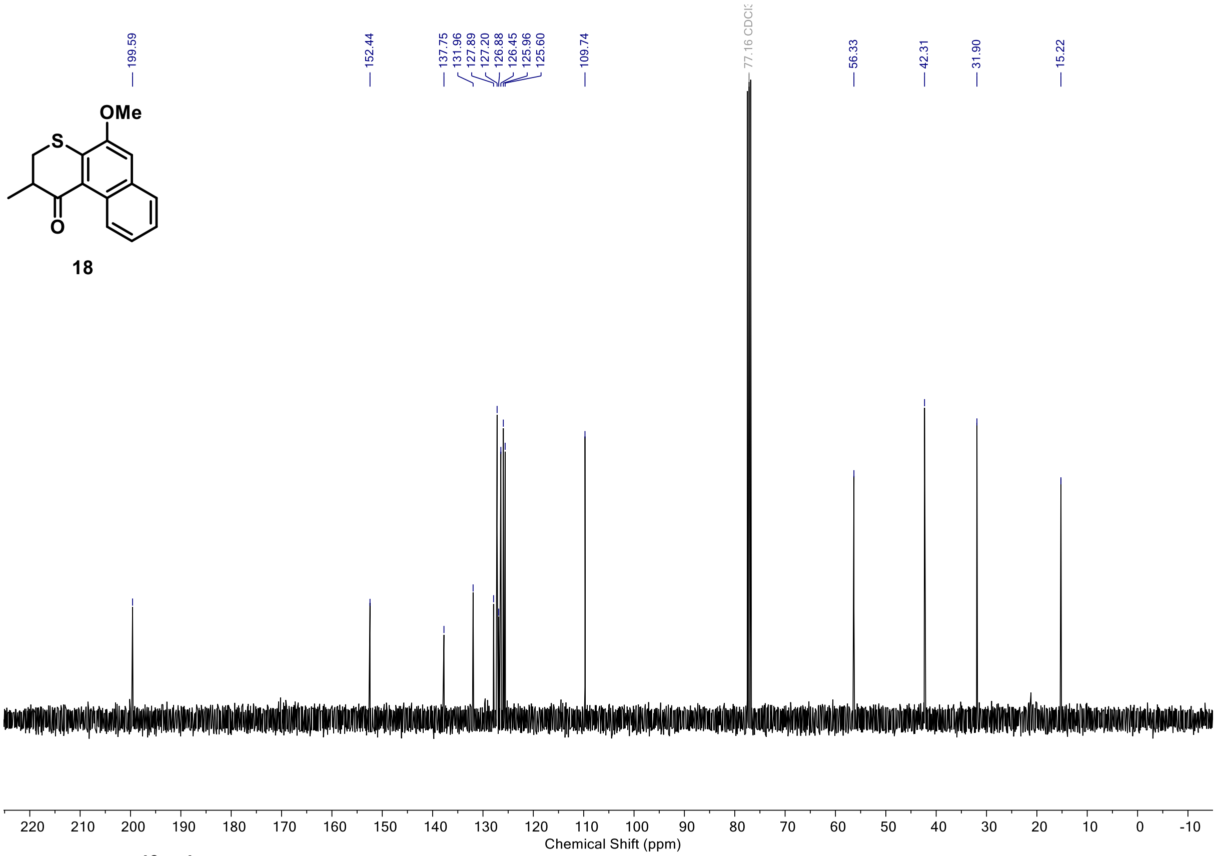

Figure S61: $^{13}C\{^{1}H\}$ NMR spectrum of **18** (101 MHz, CDCl3).

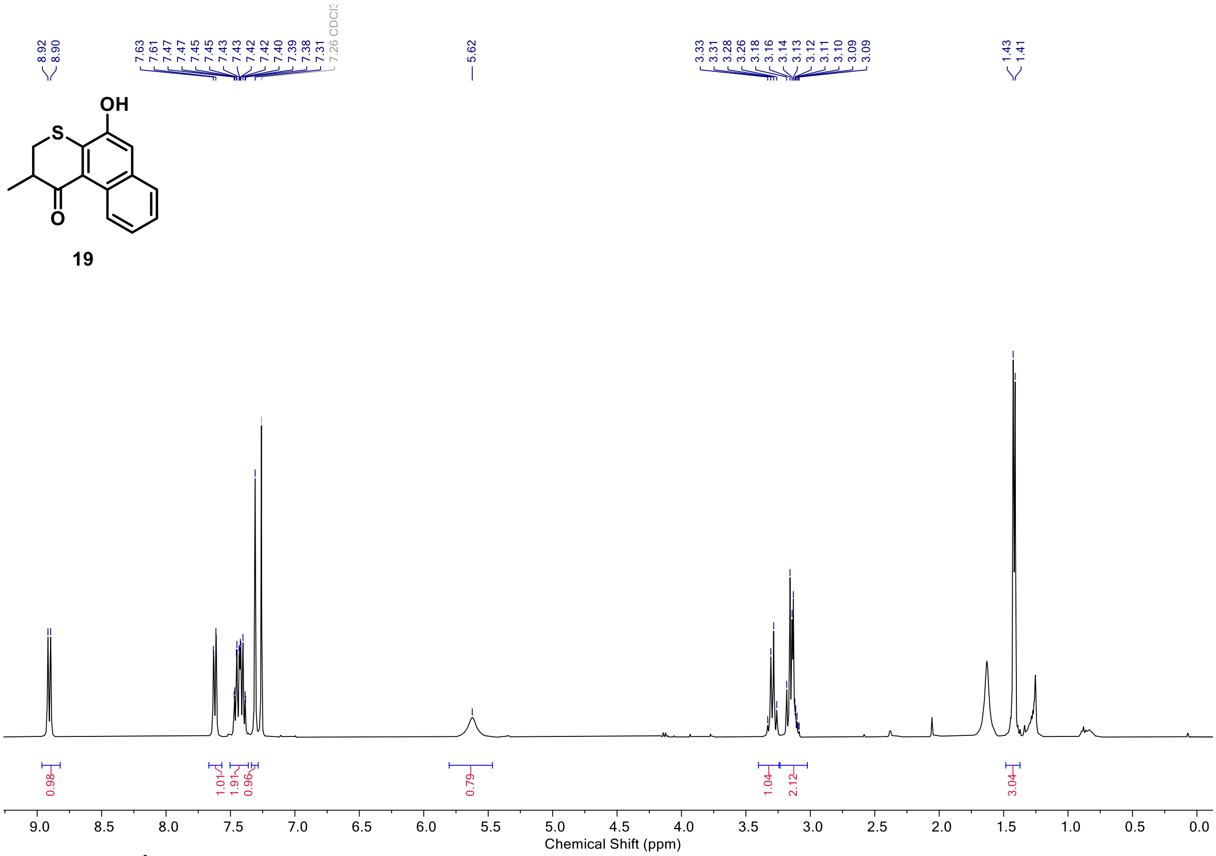

Figure S62: ^{1}H NMR spectrum of **19** (400 MHz, CDCl3).

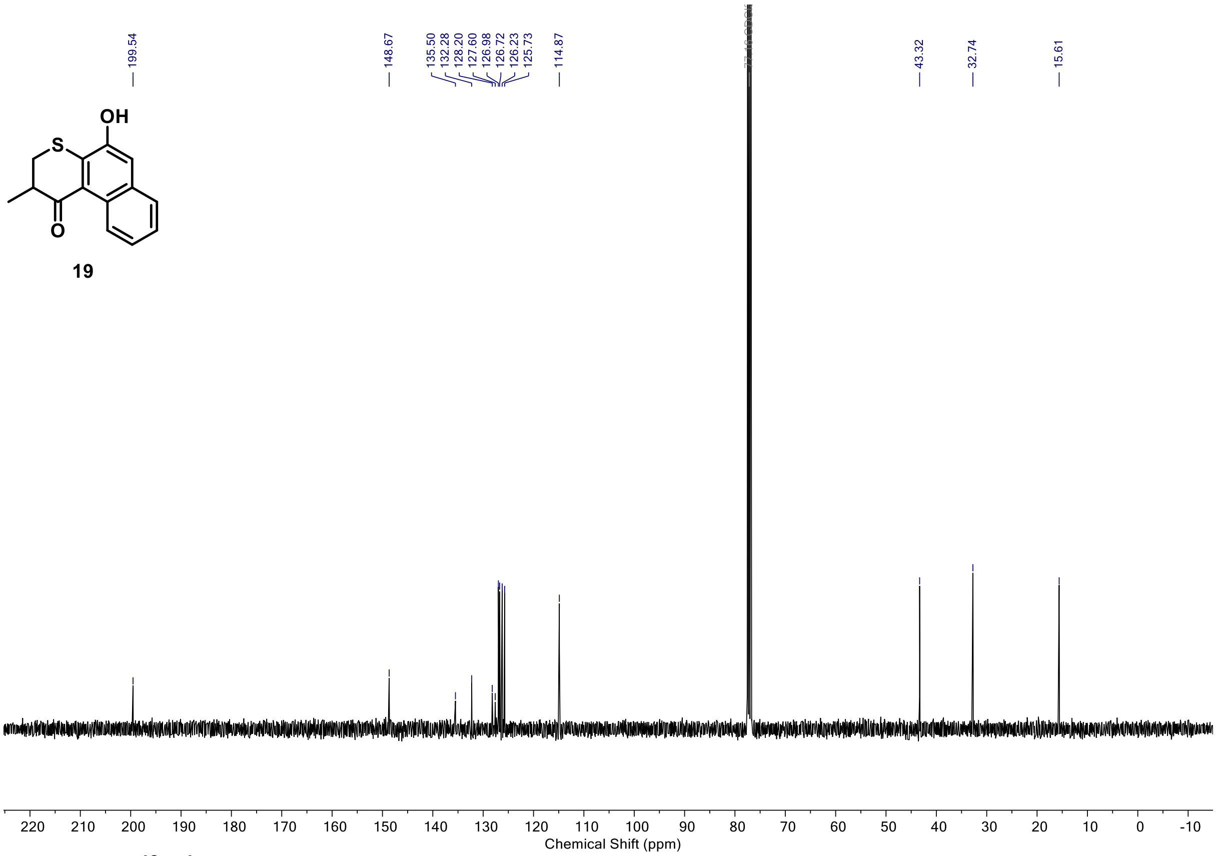

Figure S63: ^{13}C{^{1}H} NMR spectrum of **19** (101 MHz, $CDCl_3$).

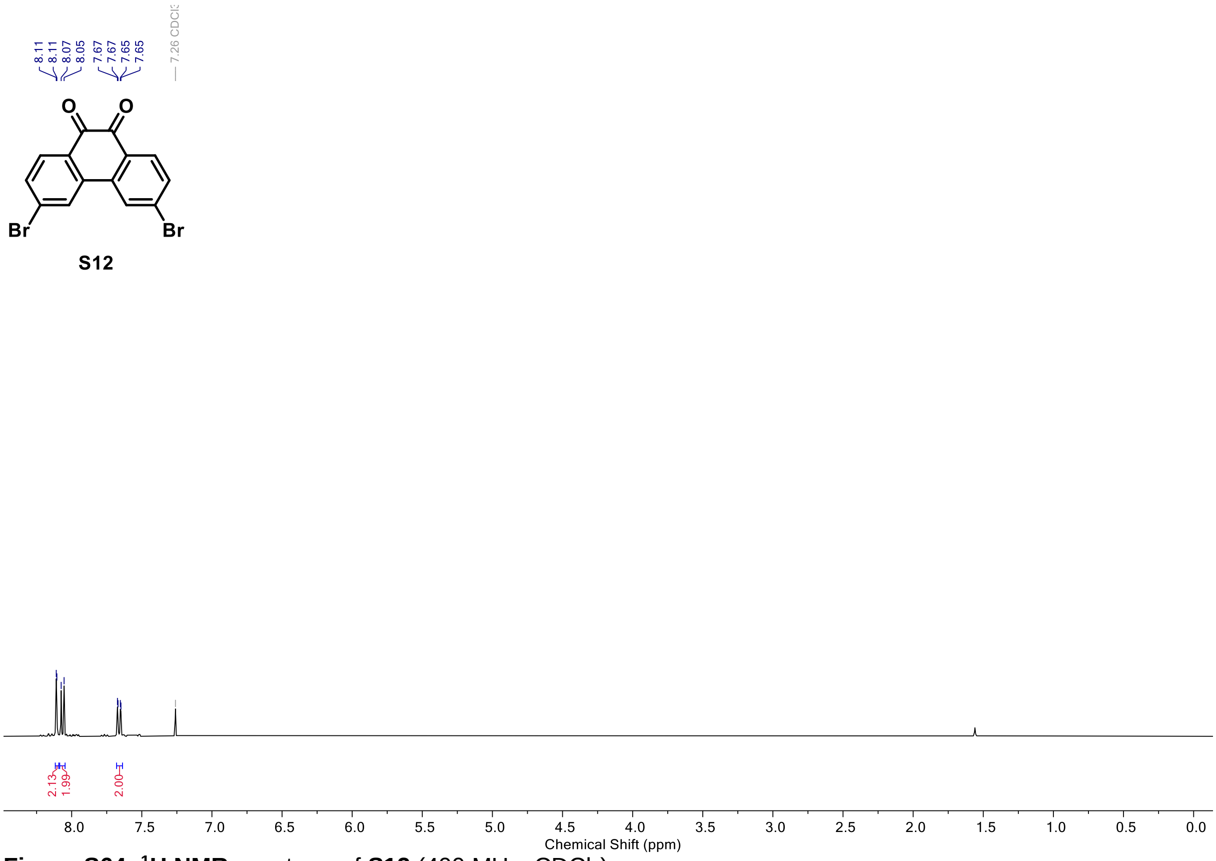

Figure S64: ^{1}H NMR spectrum of **S12** (400 MHz, $CDCl_3$).

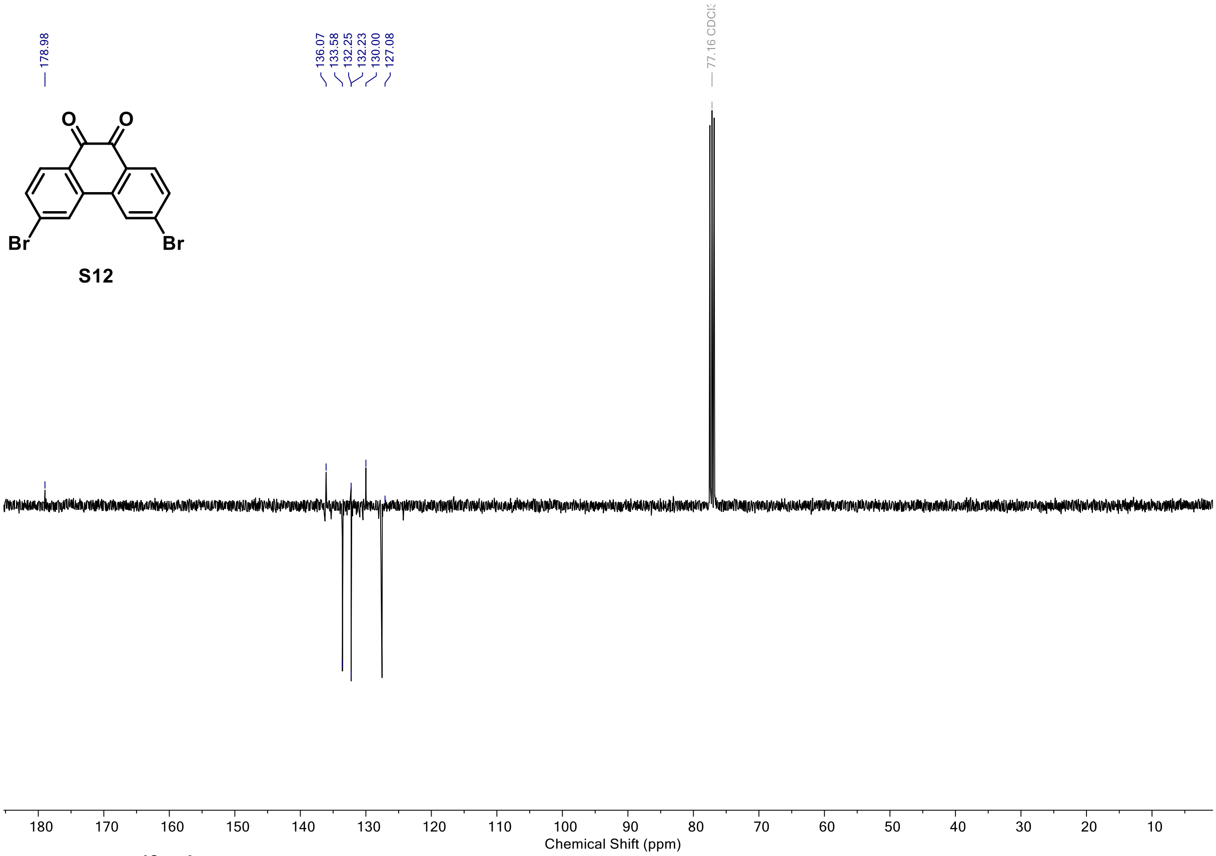

Figure S65: ^{13}**C{**^{1}**H} APT NMR** spectrum of **S12** (101 MHz, $CDCl_3$).

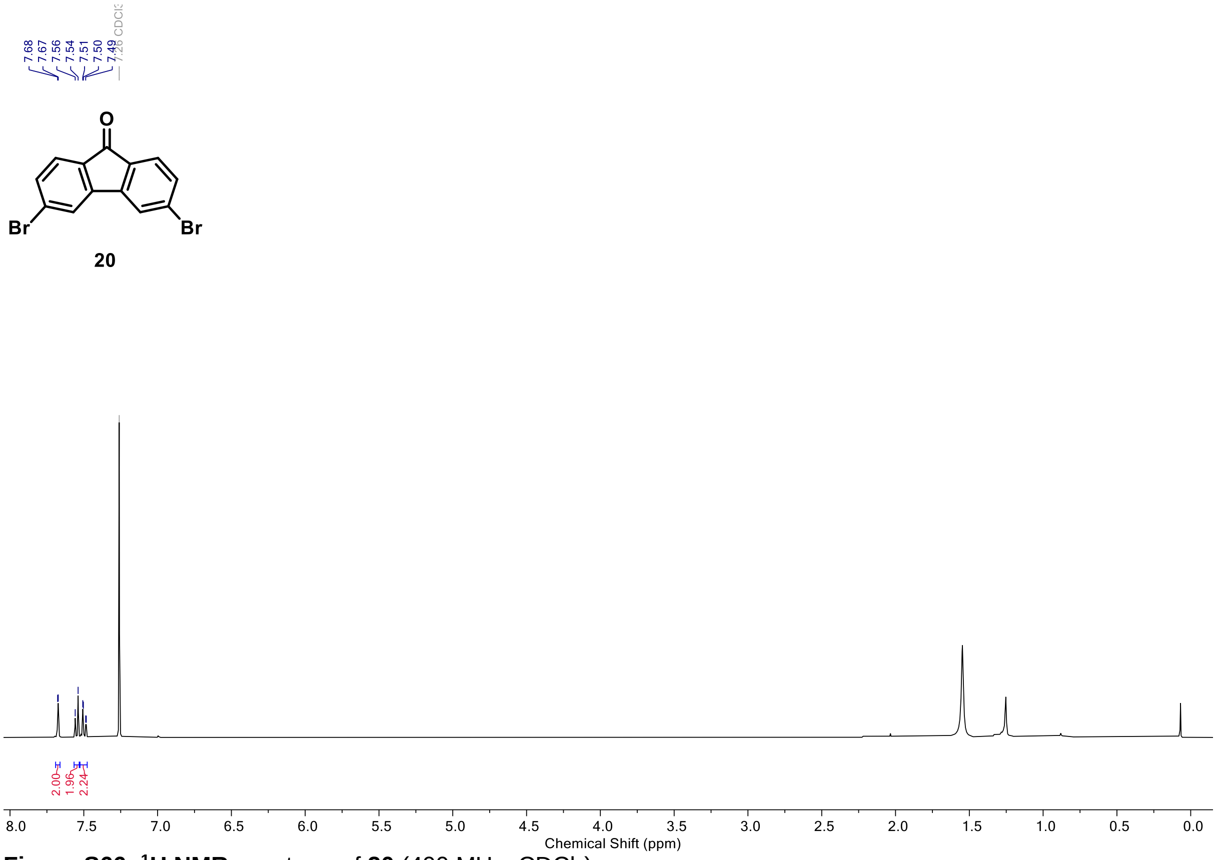

Figure S66: ^{1}**H NMR** spectrum of **20** (400 MHz, $CDCl_3$).

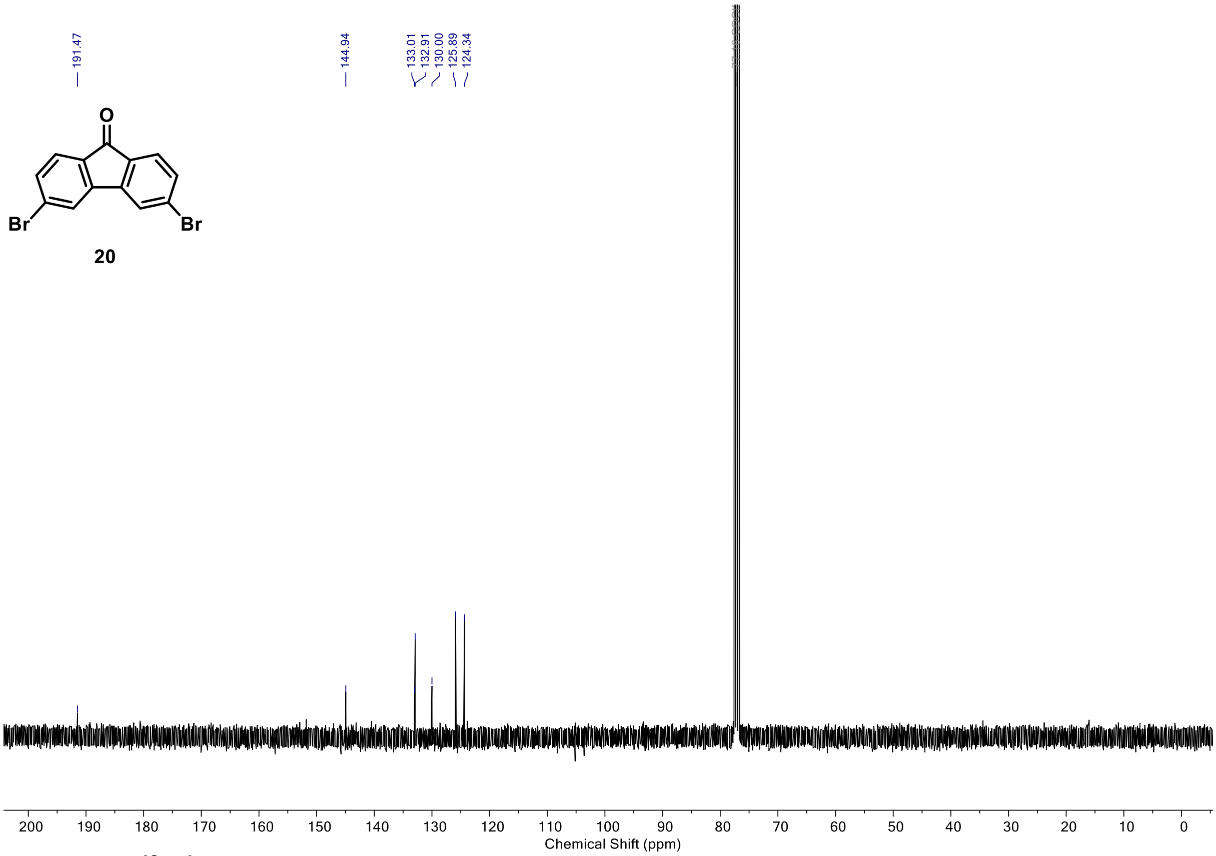

Figure S67: ^{13}C{^{1}H} NMR spectrum of **20** (101 MHz, $CDCl_3$).

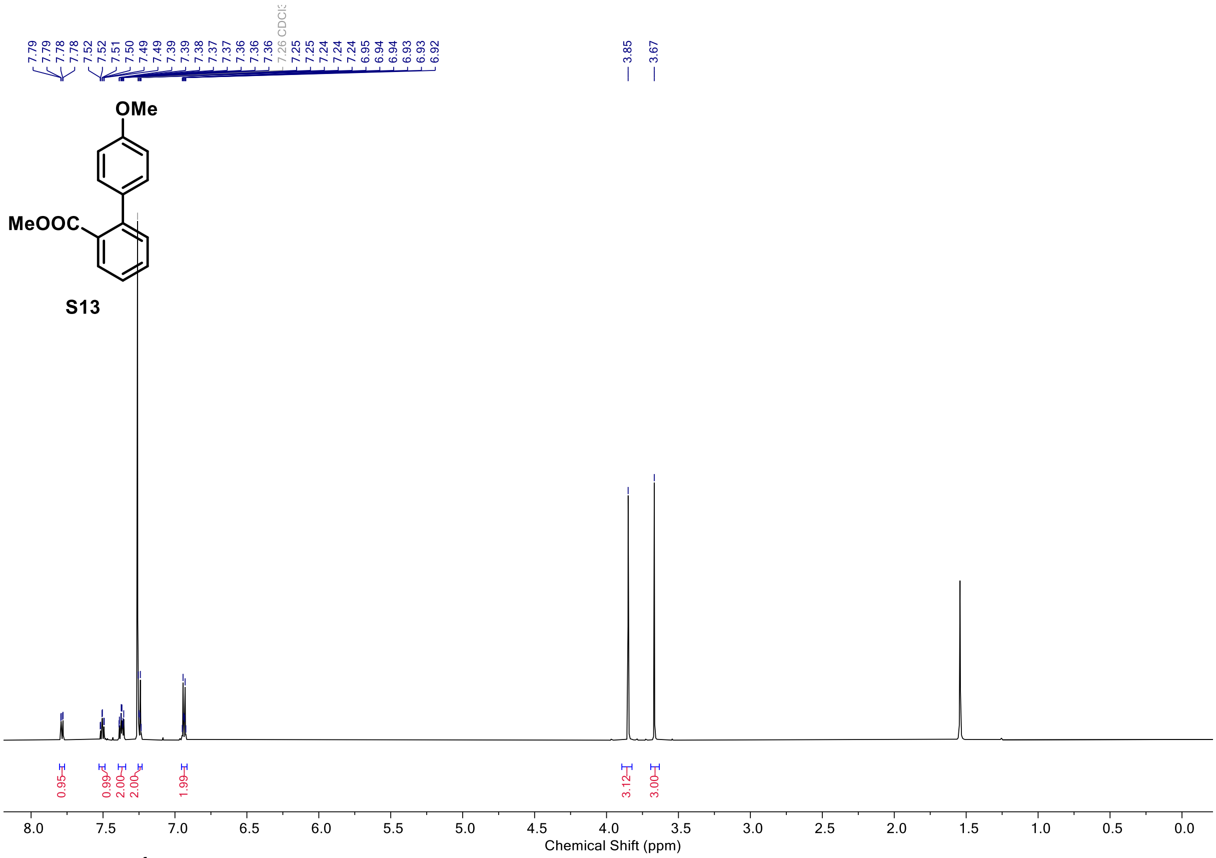

Figure S68: ^{1}H NMR spectrum of methyl **S13** (600 MHz, $CDCl_3$).

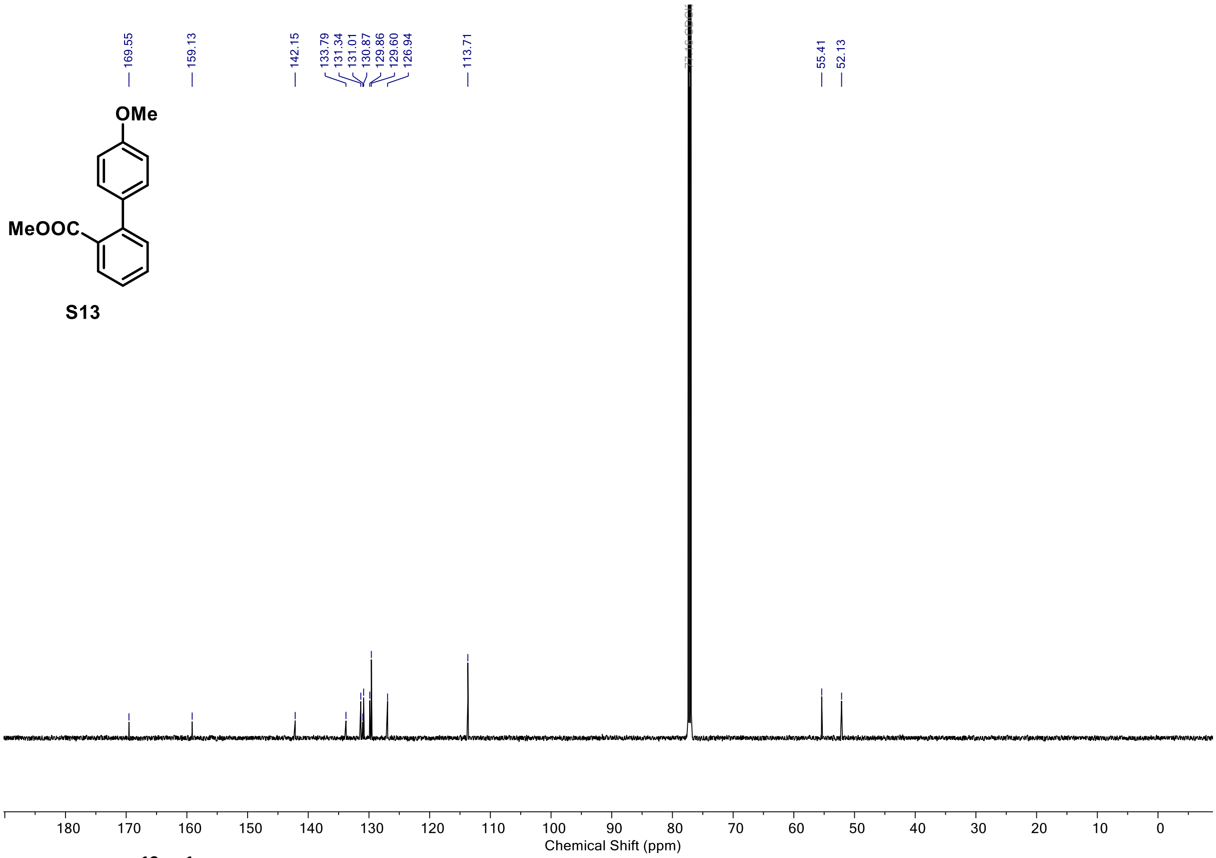

Figure S69: $^{13}C\{^{1}H\}$ **NMR** spectrum of **S13** (151 MHz, $CDCl_3$).

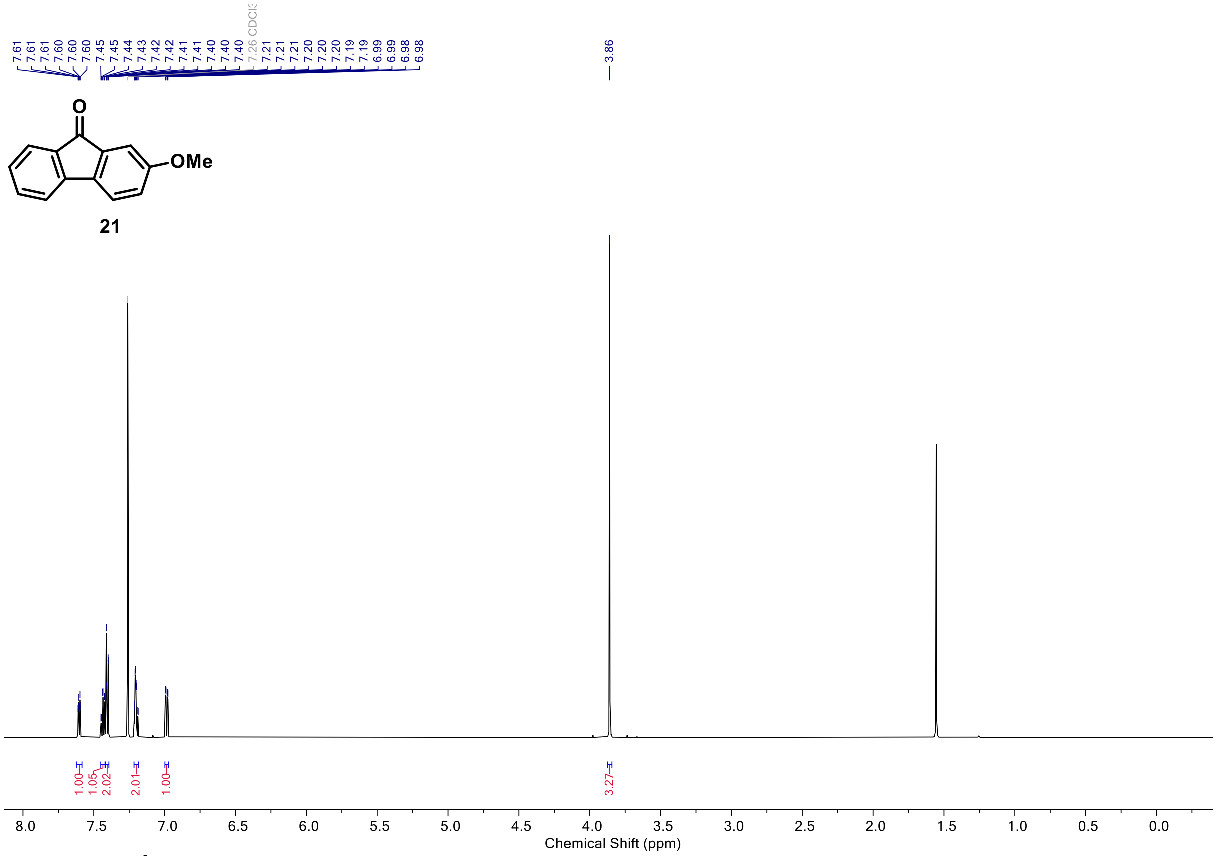

Figure S70: ^{1}**H NMR** spectrum of **21** (600 MHz, $CDCl_3$).

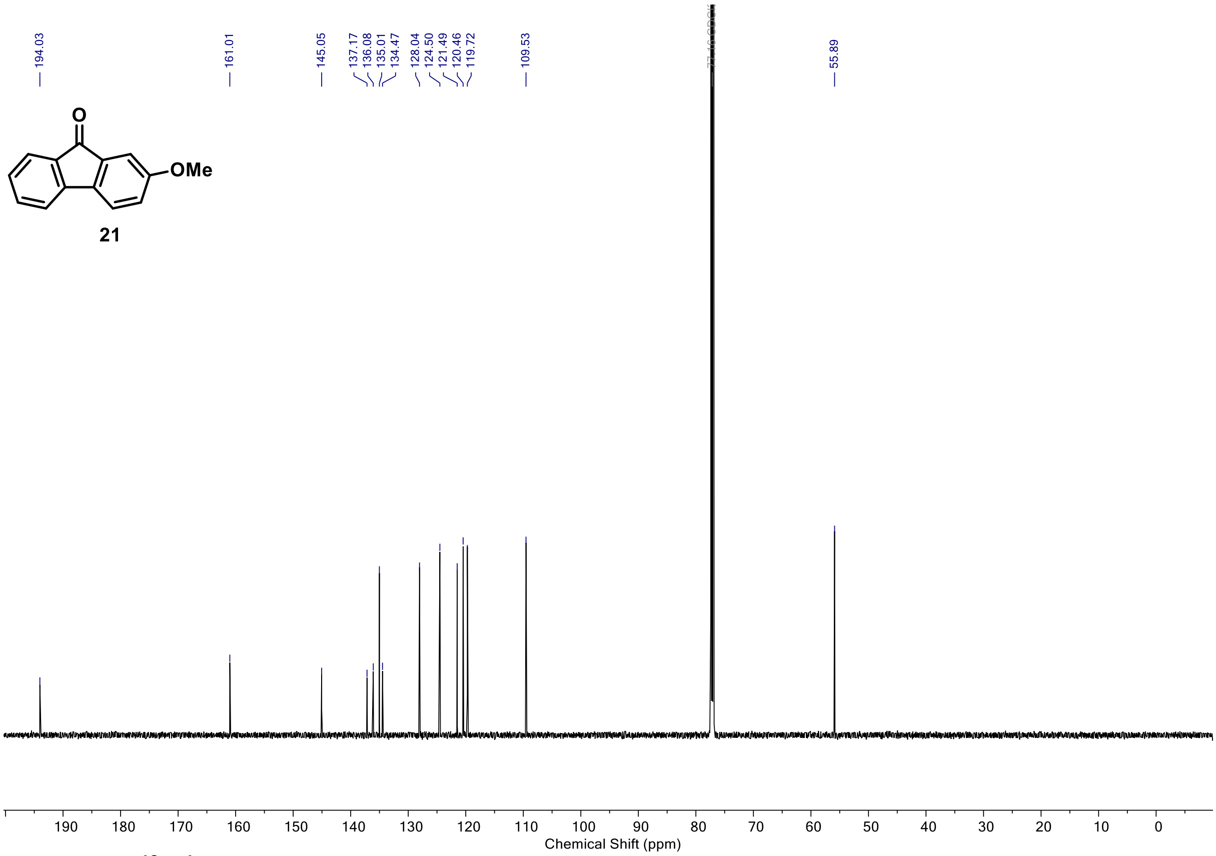

Figure S71: $^{13}C\{^{1}H\}$ NMR spectrum of **21** (151 MHz, $CDCl_3$).

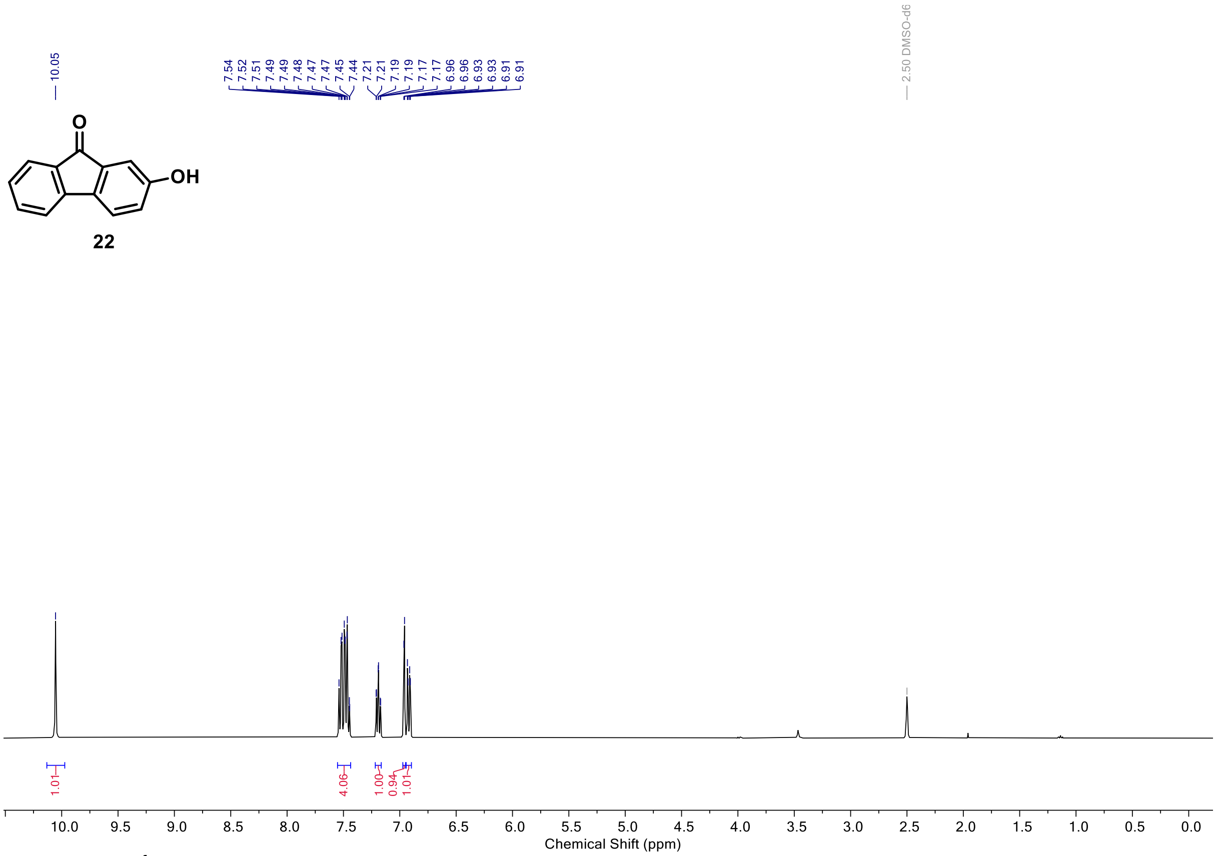

Figure S72: ^{1}H NMR spectrum of **22** (400 MHz, $(CD_3)_2SO$).

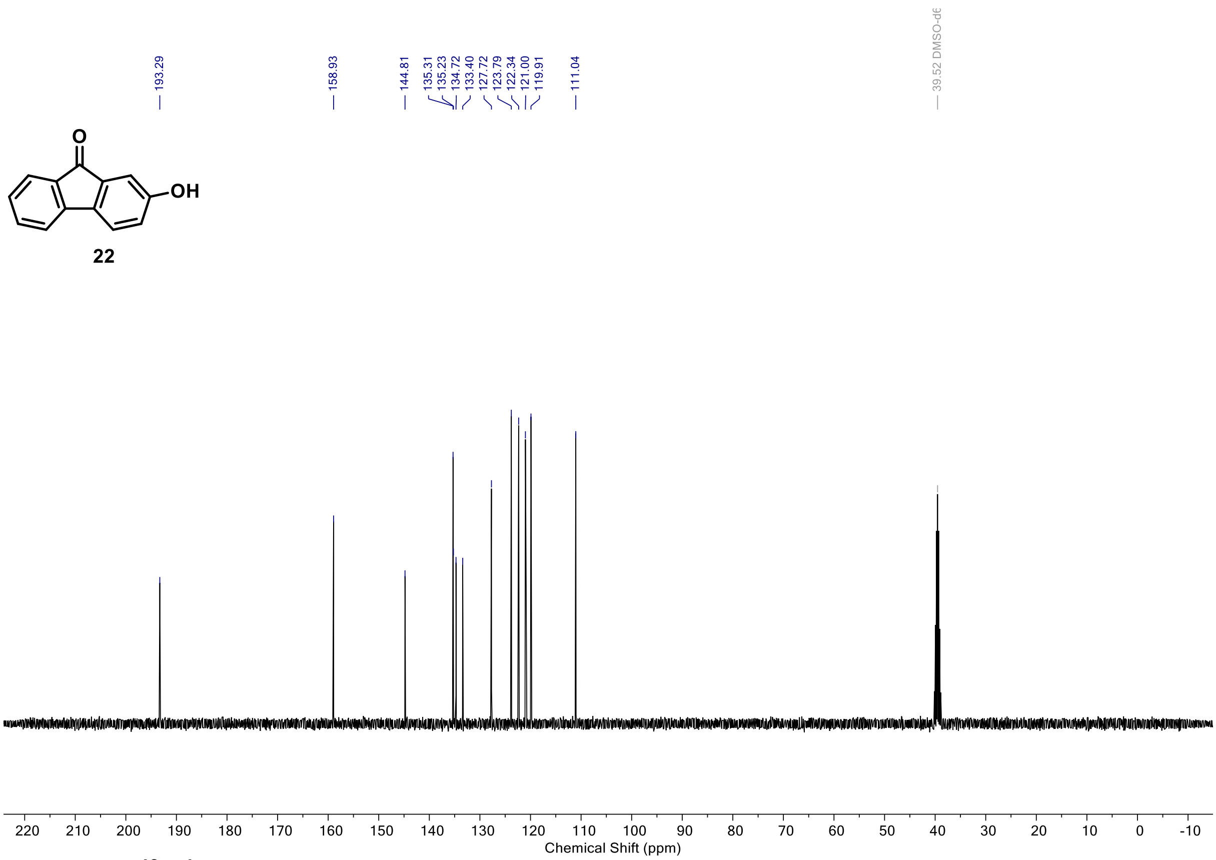

Figure S73: ^{13}C{^{1}H} NMR spectrum of **22** (101 MHz, $(CD_3)_2SO$).

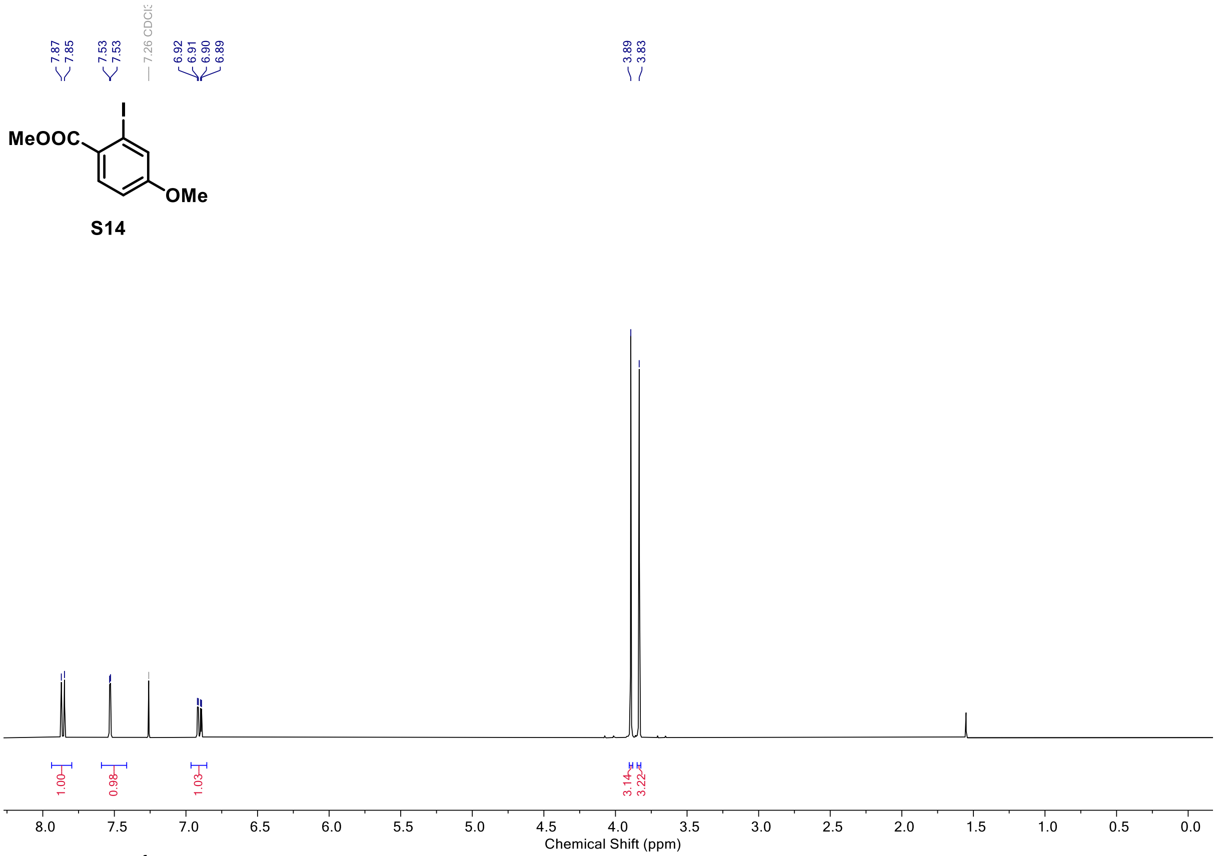

Figure S74: ^{1}H NMR spectrum of **S14** (400 MHz, $CDCl_3$).

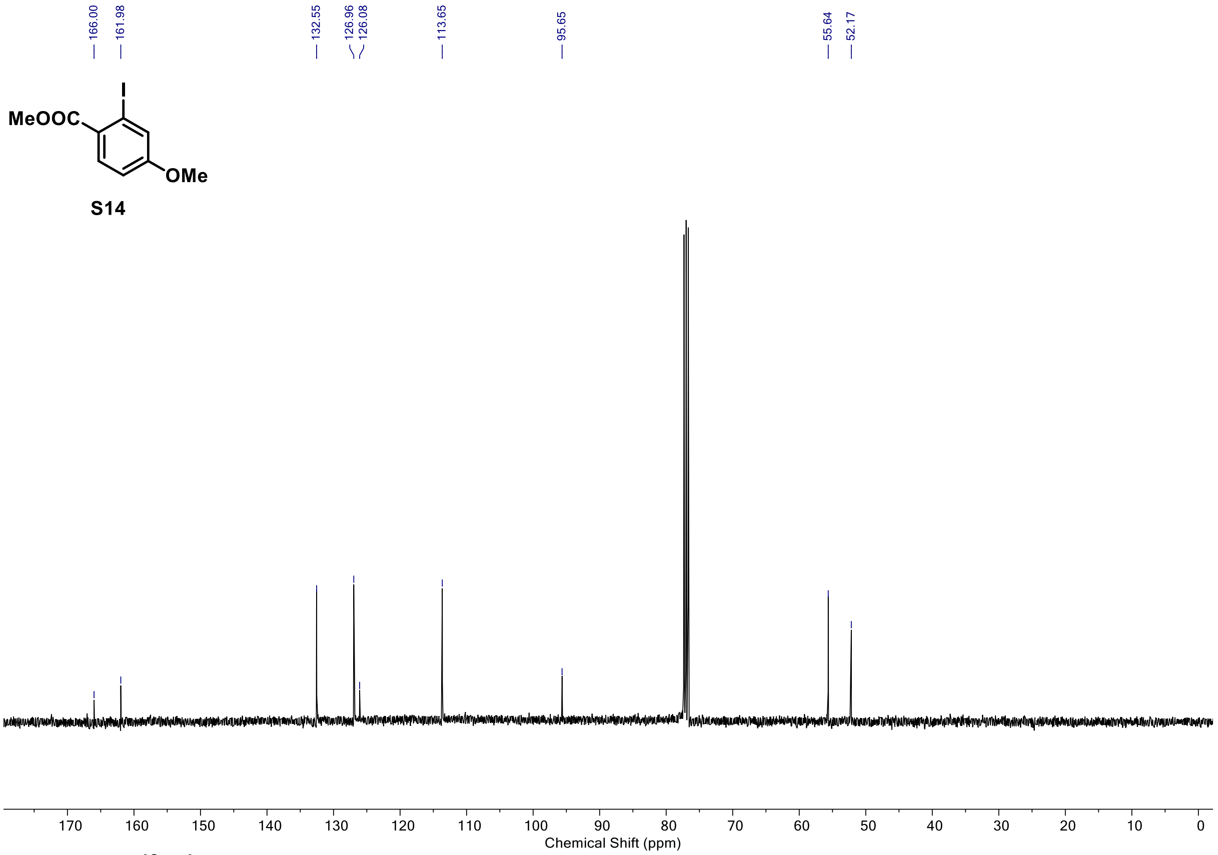

Figure S75: $^{13}C\{^{1}H\}$ NMR spectrum of **S14** (101 MHz, $CDCl_3$).

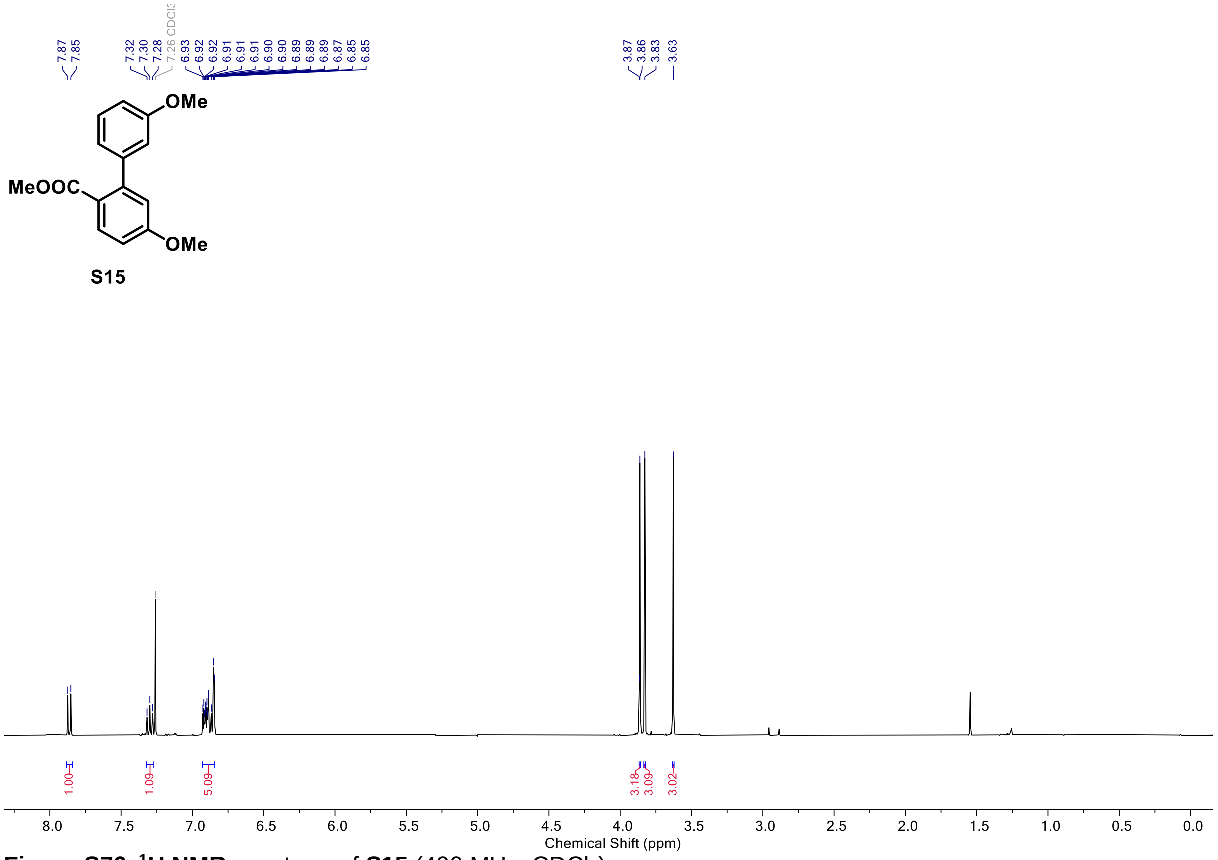

Figure S76: ^{1}H NMR spectrum of **S15** (400 MHz, $CDCl_3$).

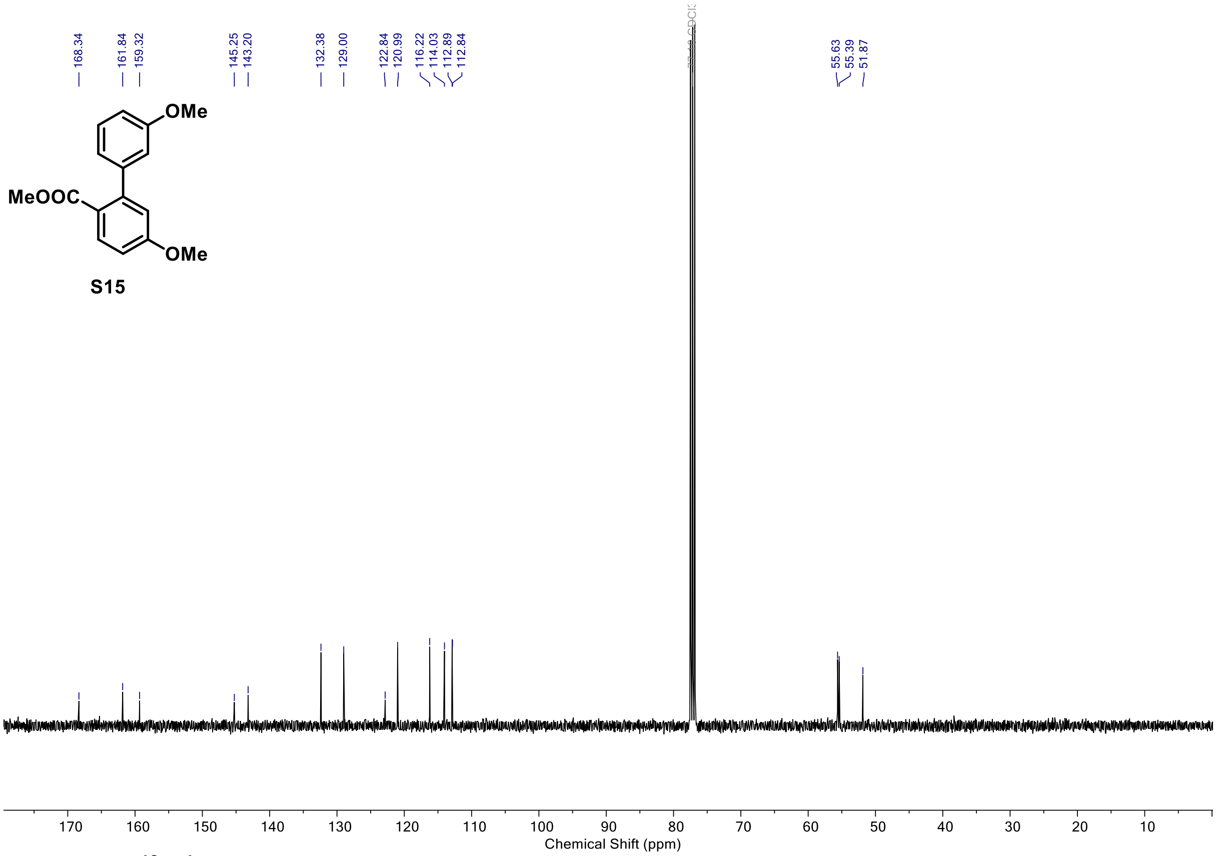

Figure S77: ^{13}C{^{1}H} NMR spectrum of **S15** (101 MHz, $CDCl_3$).

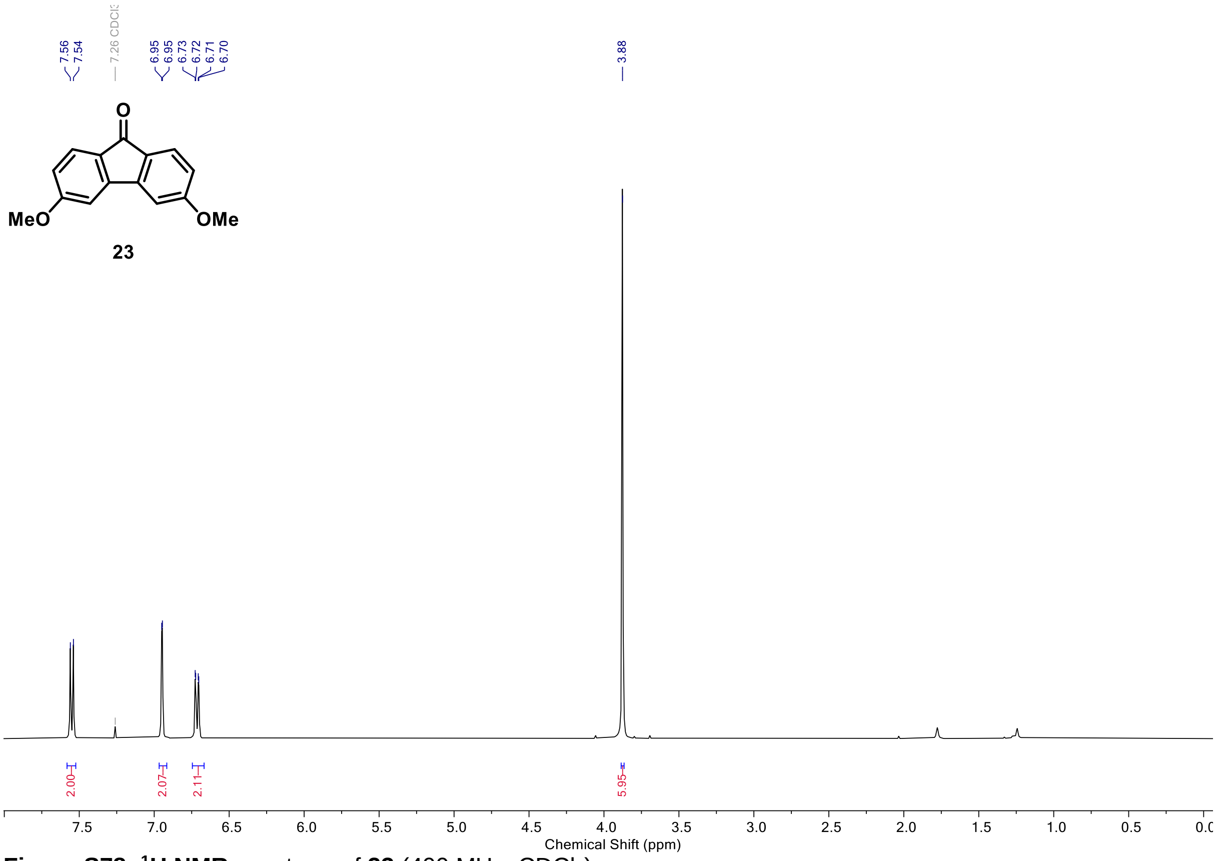

Figure S78: ^{1}H NMR spectrum of **23** (400 MHz, $CDCl_3$).

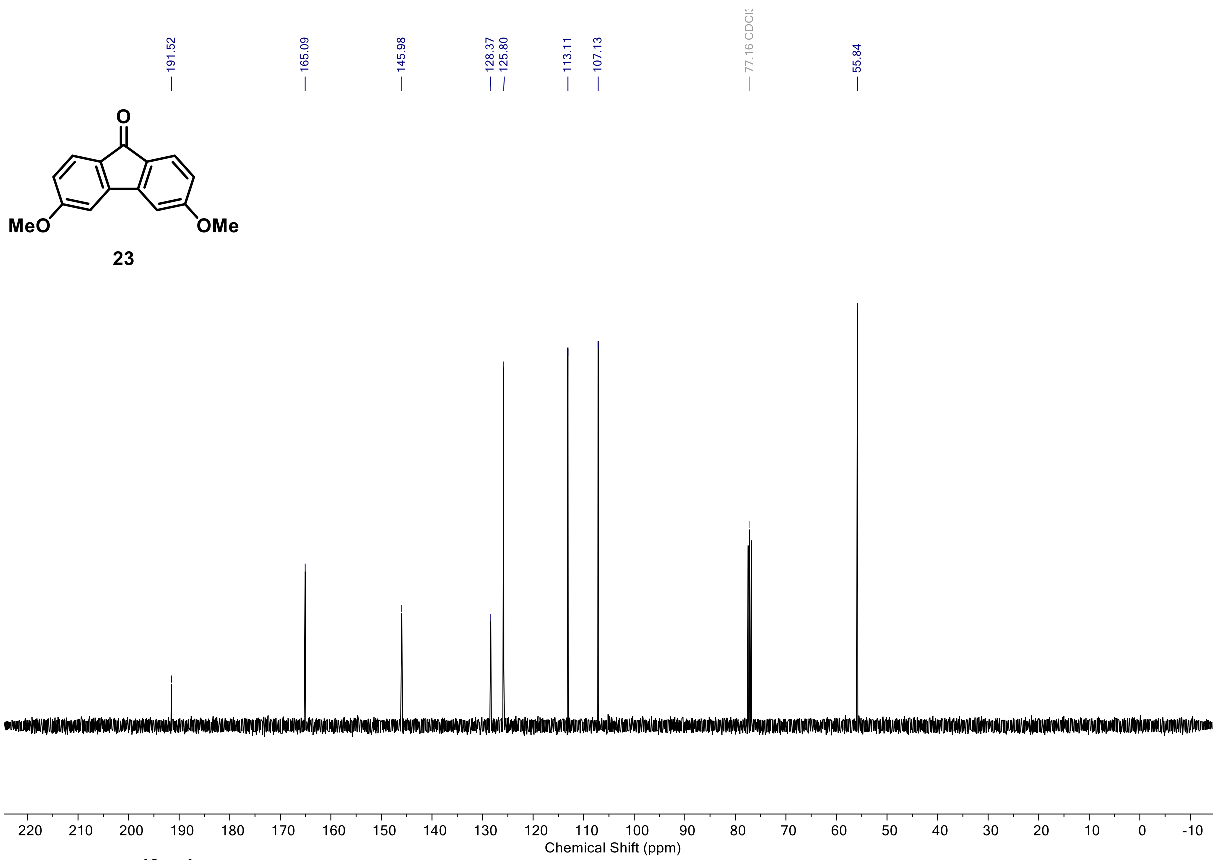

Figure S79: $^{13}C\{^1H\}$ NMR spectrum of **23** (101 MHz, $CDCl_3$).

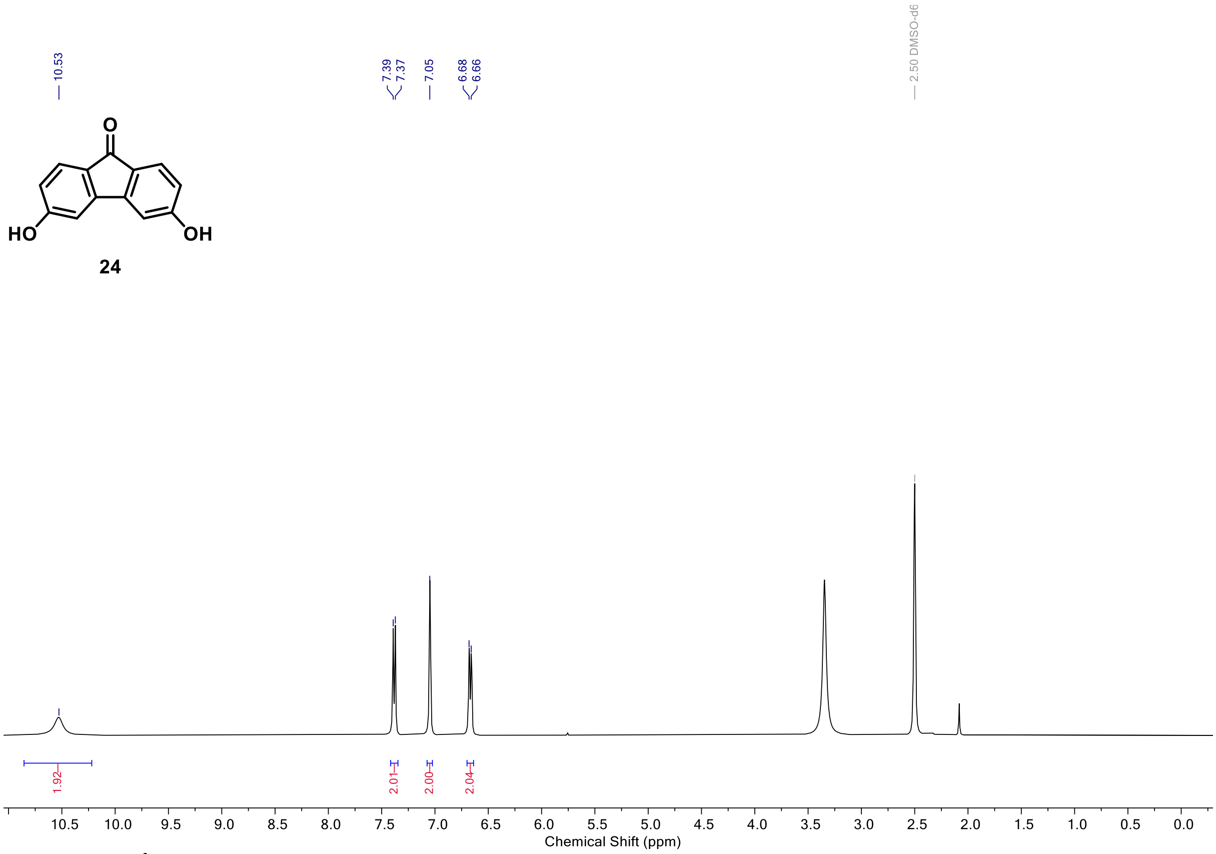

Figure S80: ^1H NMR spectrum of **24** (400 MHz, $(CD_3)_2SO$).

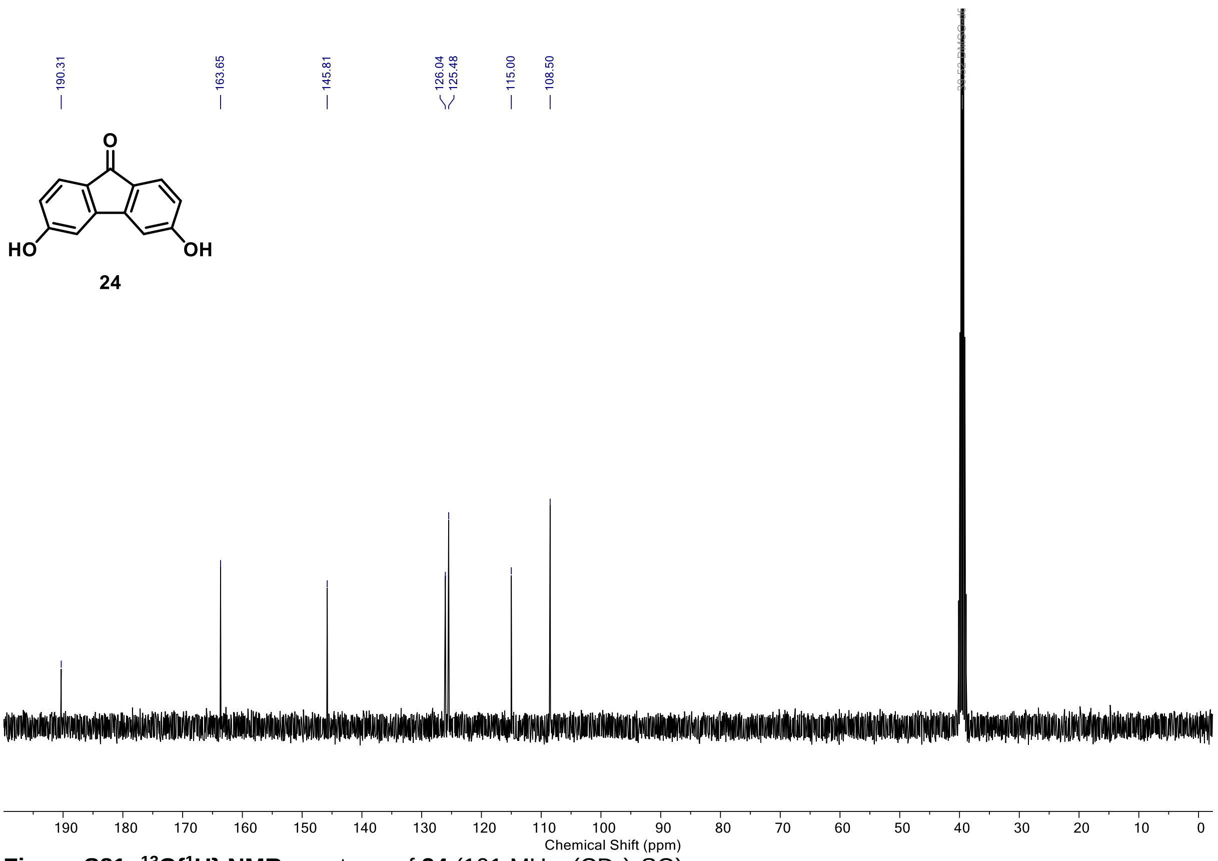

Figure S81: $^{13}C\{^1H\}$ NMR spectrum of **24** (101 MHz, $(CD_3)_2SO$).

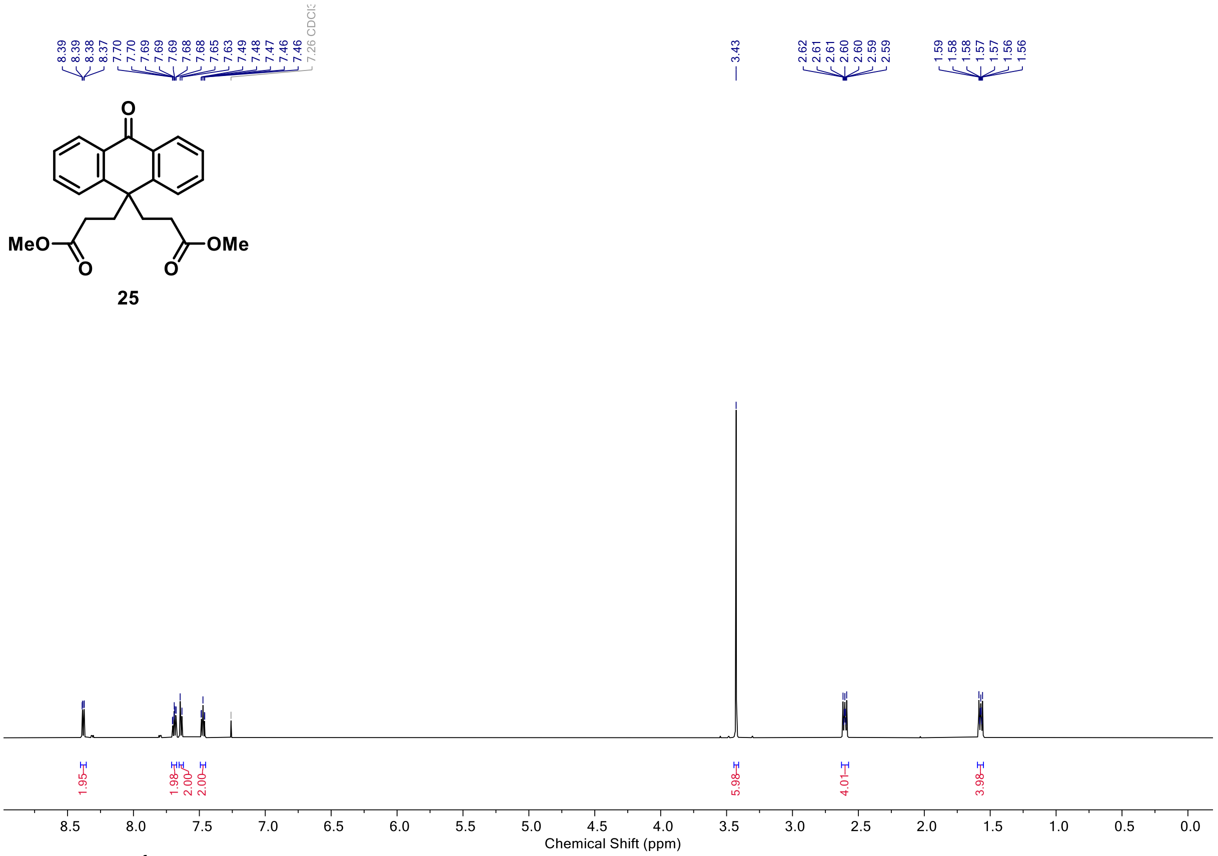

Figure S82: ^{1}H NMR spectrum of **25** (600 MHz, $CDCl_3$).

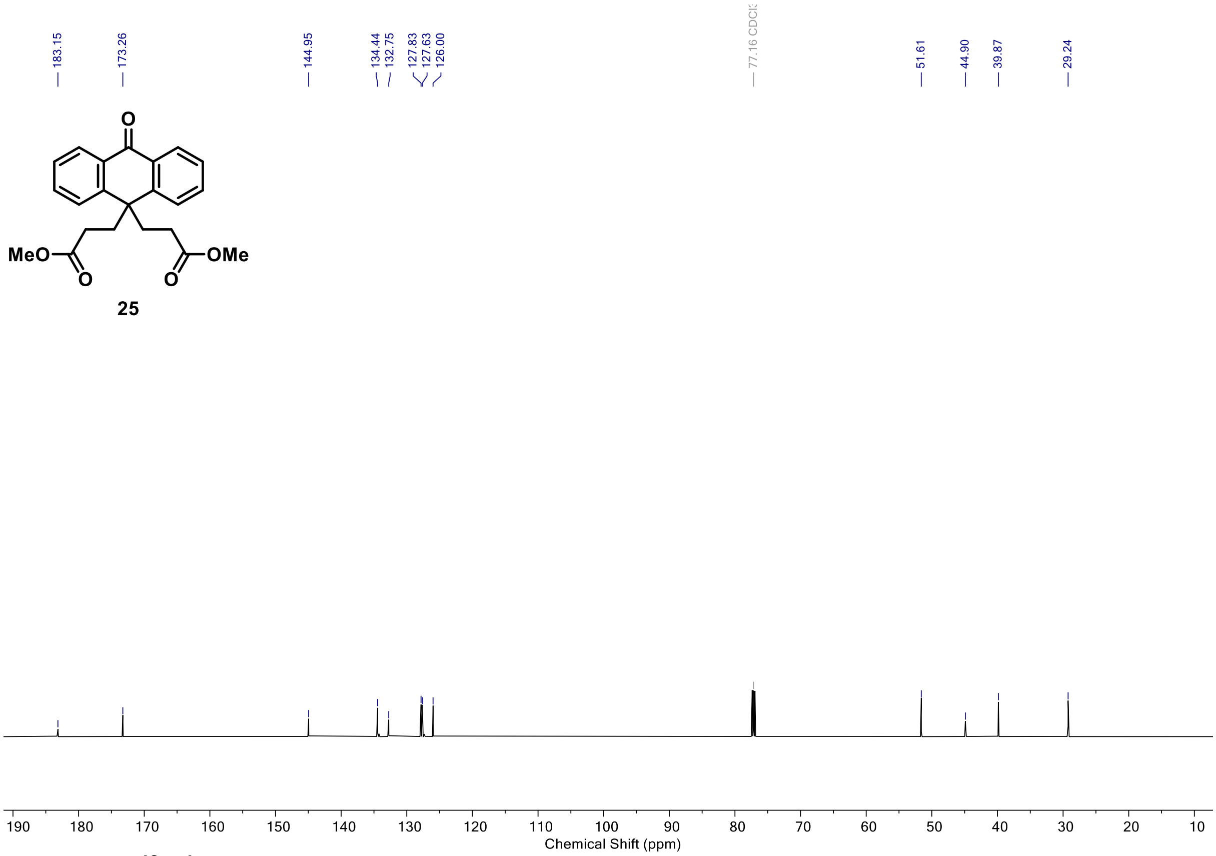

Figure S83: ^{13}C{^{1}H} NMR spectrum of **25** (151 MHz, $CDCl_3$).

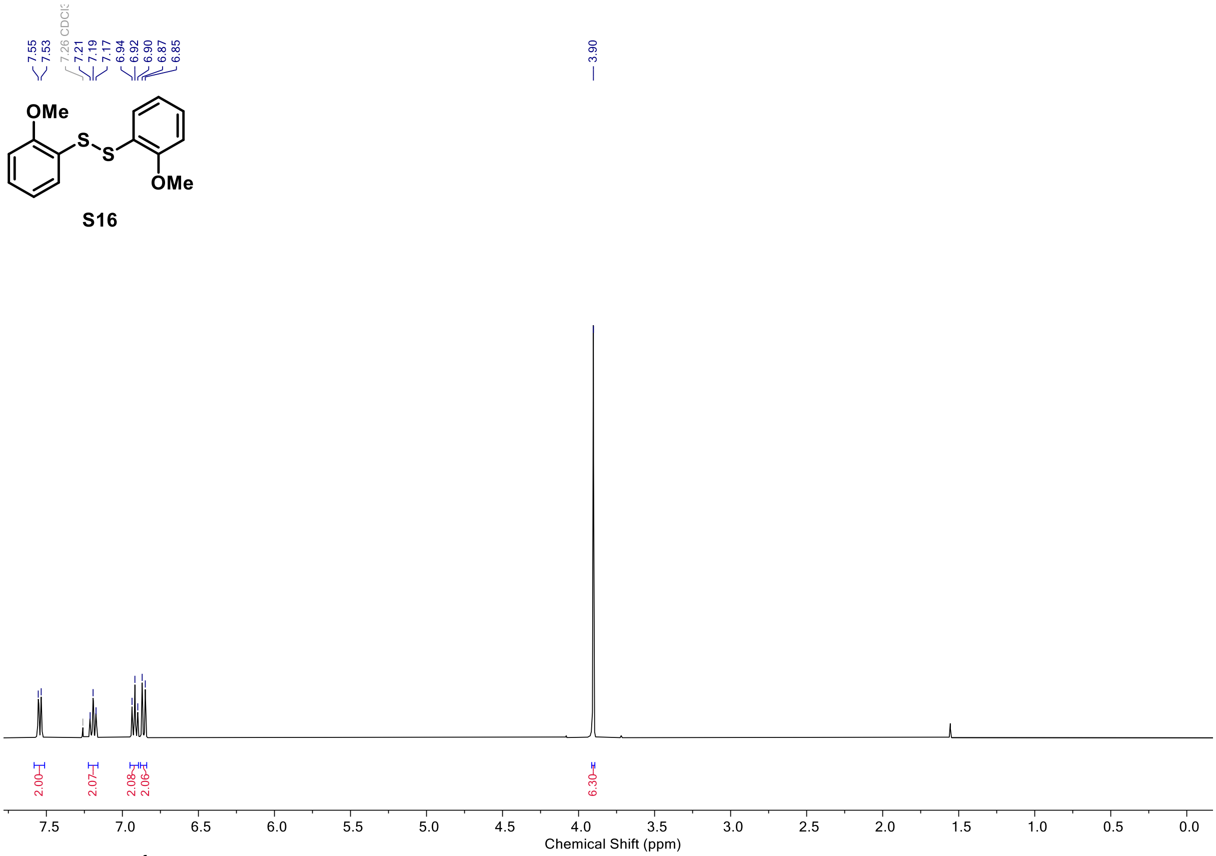

Figure S84: ^{1}H NMR spectrum of **S16** (400 MHz, $CDCl_3$).

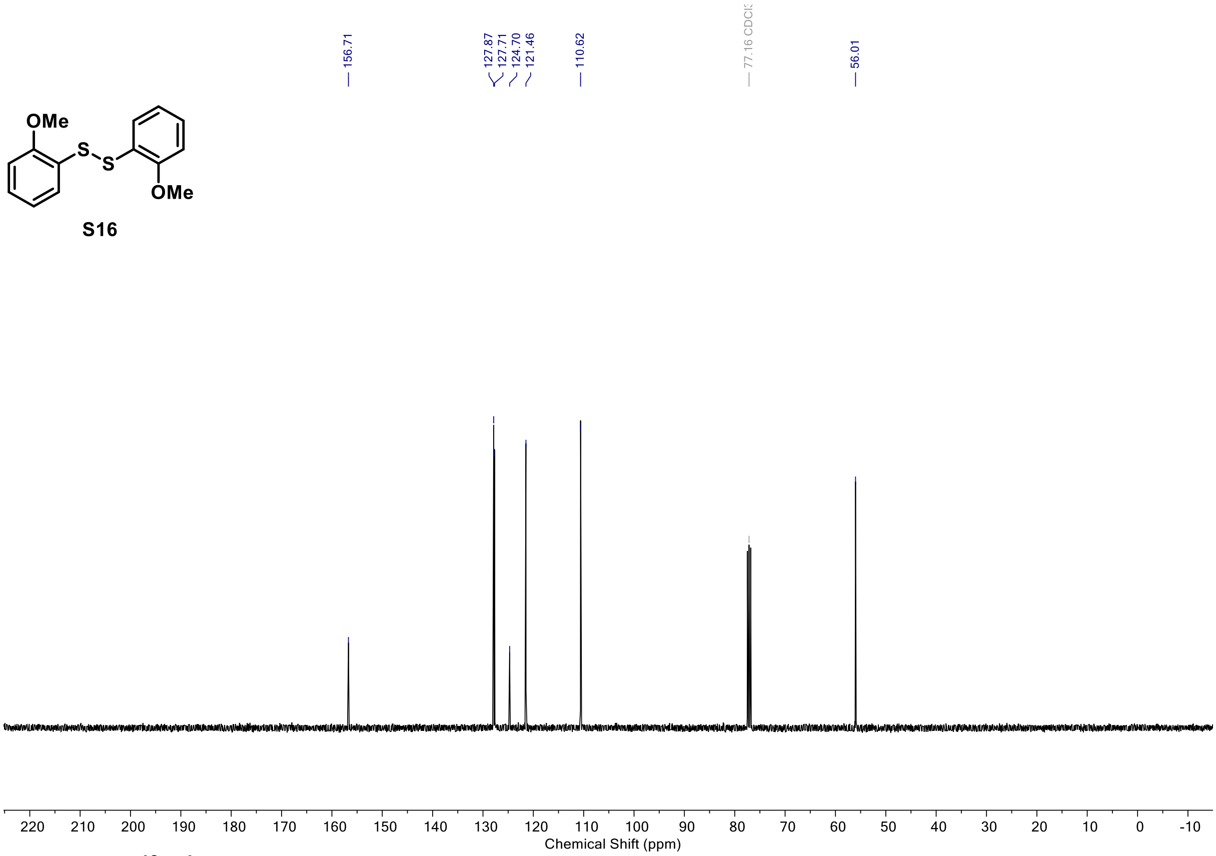

Figure S85: $^{13}C\{^1H\}$ NMR spectrum of **S16** (101 MHz, $CDCl_3$).

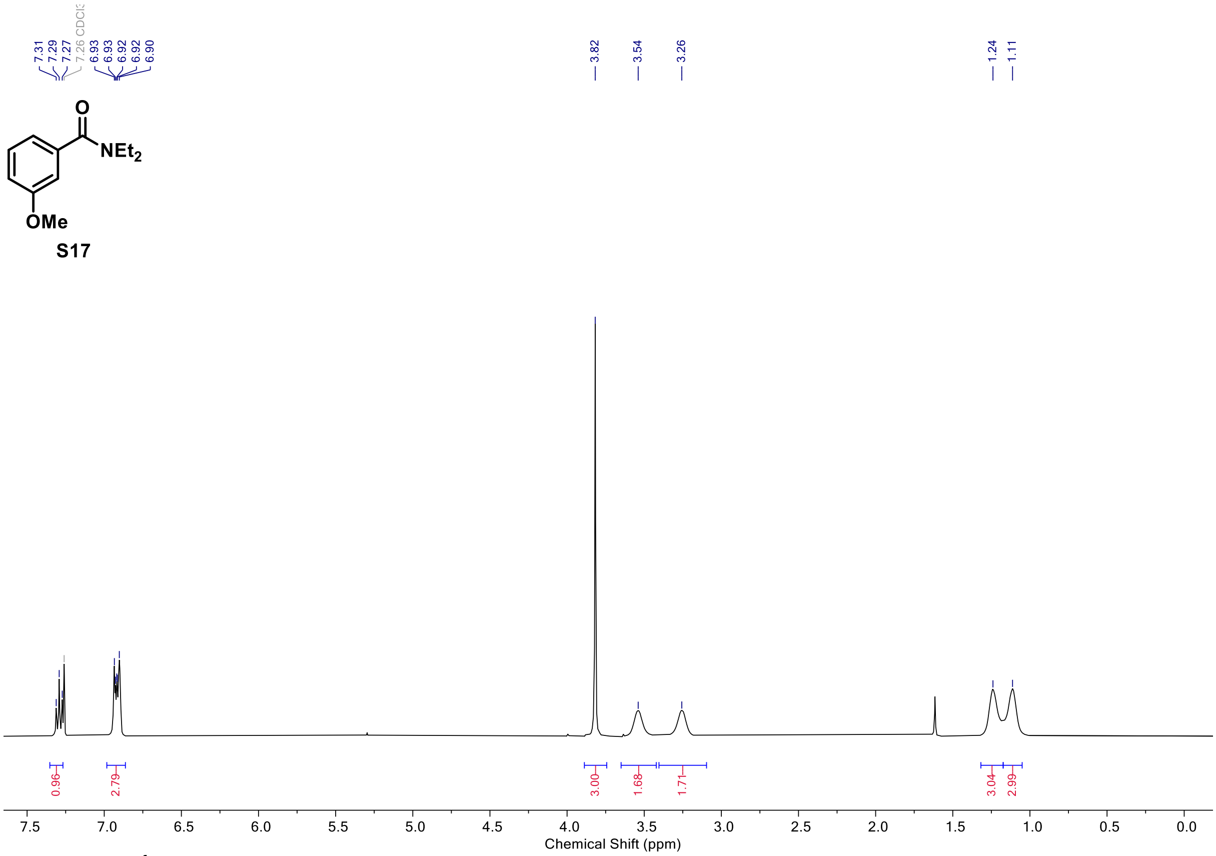

Figure S86: ^1H NMR spectrum of **S17** (400 MHz, $CDCl_3$).

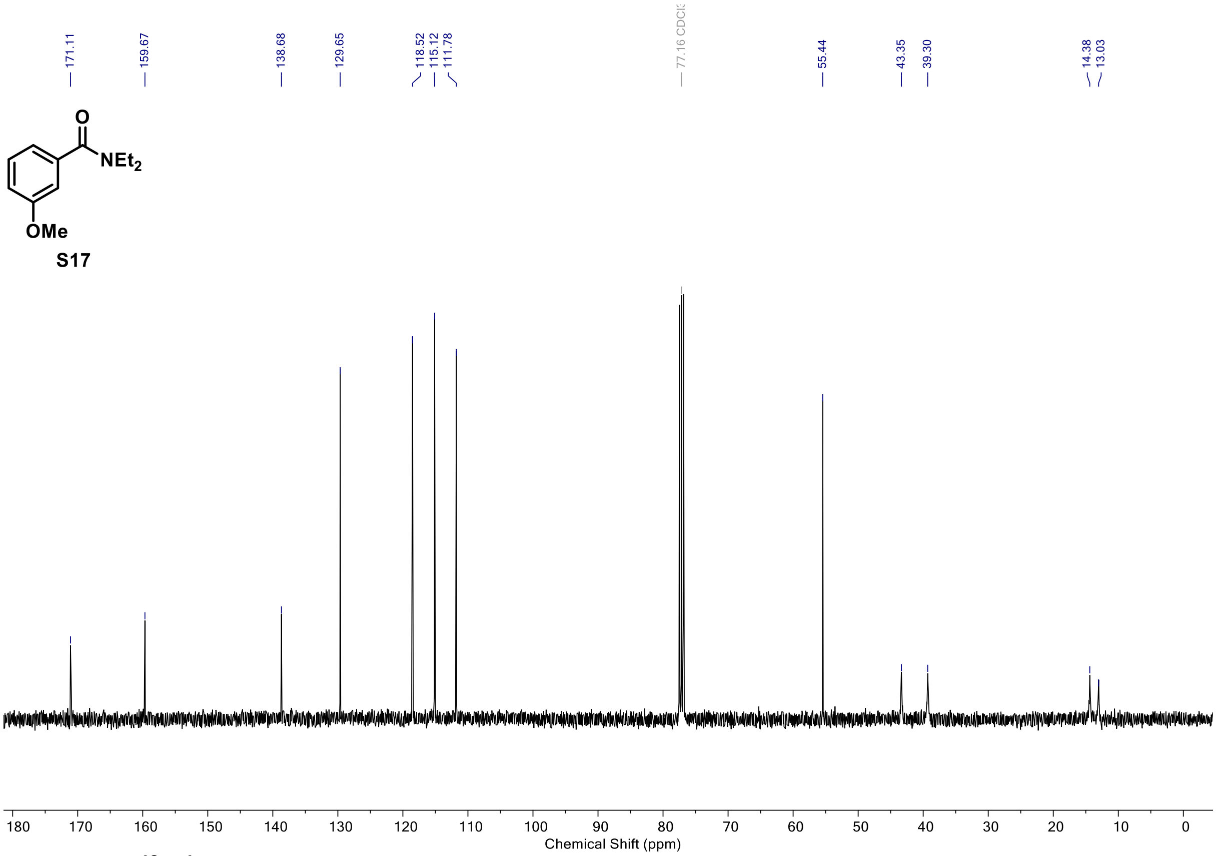

Figure S87: $^{13}C\{^{1}H\}$ NMR spectrum of **S17** (101 MHz, $CDCl_3$).

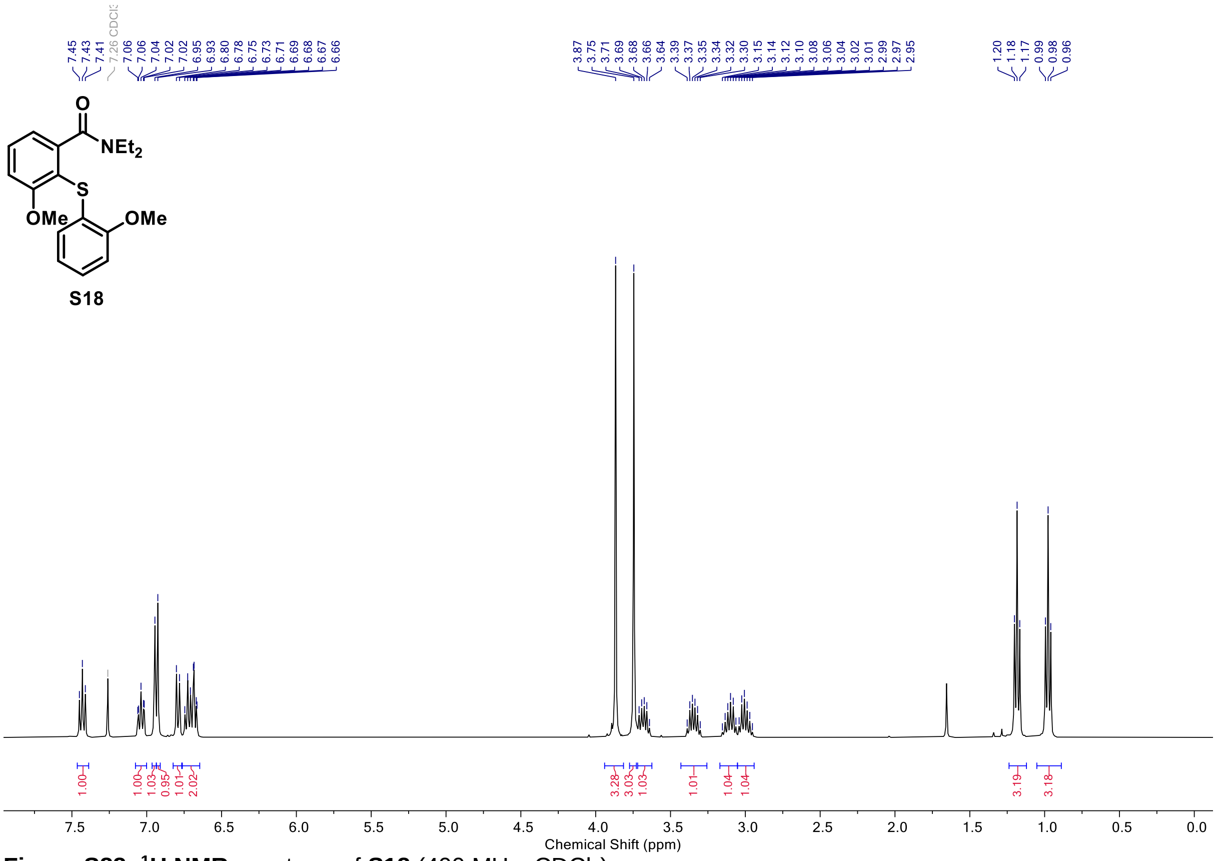

Figure S88: ^{1}H NMR spectrum of **S18** (400 MHz, $CDCl_3$).

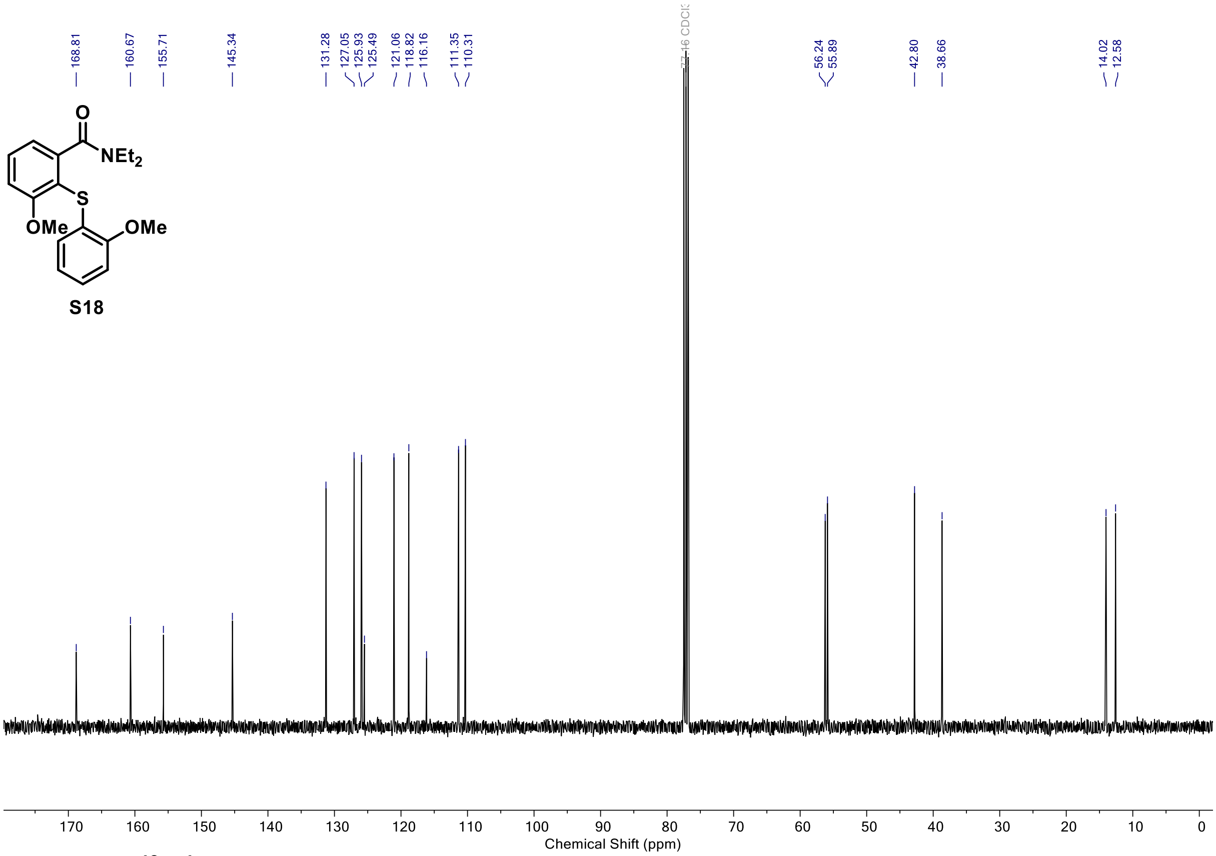

Figure S89: $^{13}C\{^{1}H\}$ NMR spectrum of **S18** (101 MHz, $CDCl_3$).

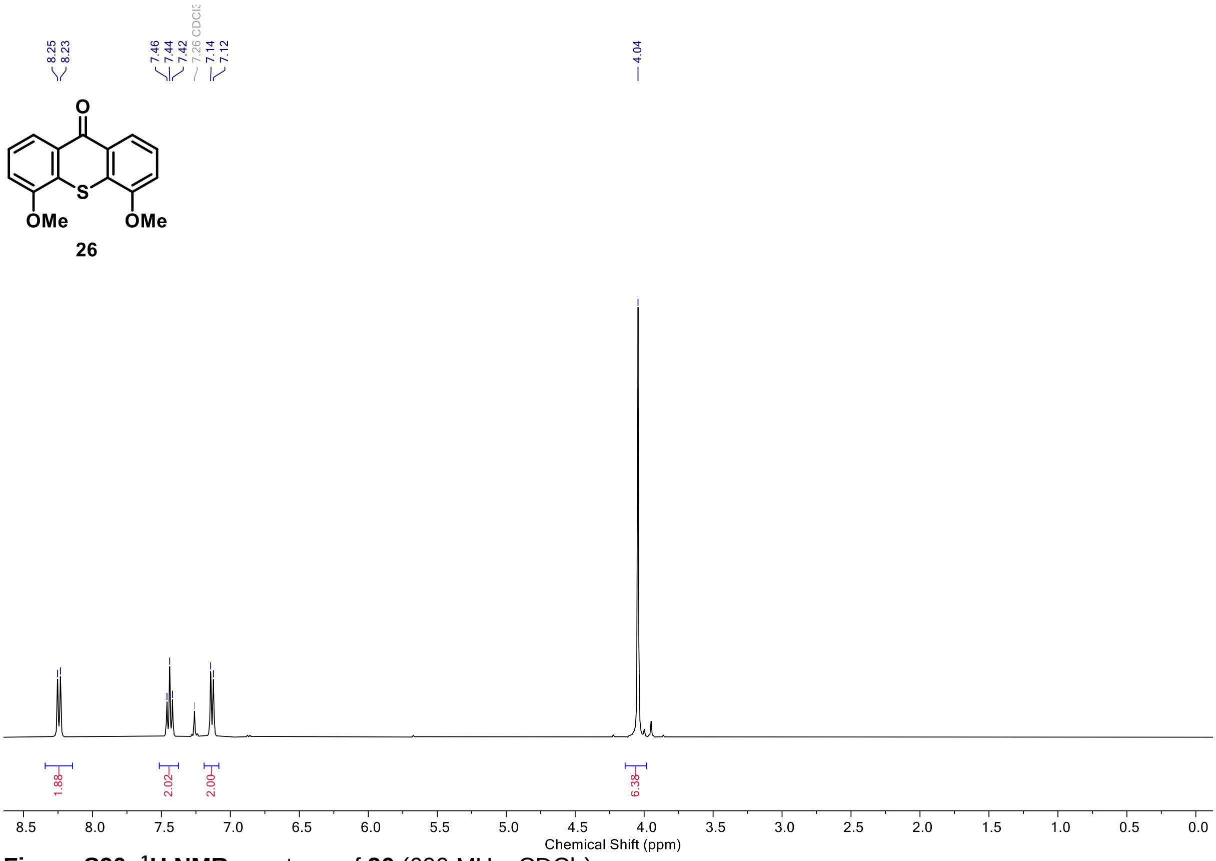

Figure S90: ^{1}H NMR spectrum of **26** (600 MHz, $CDCl_3$).

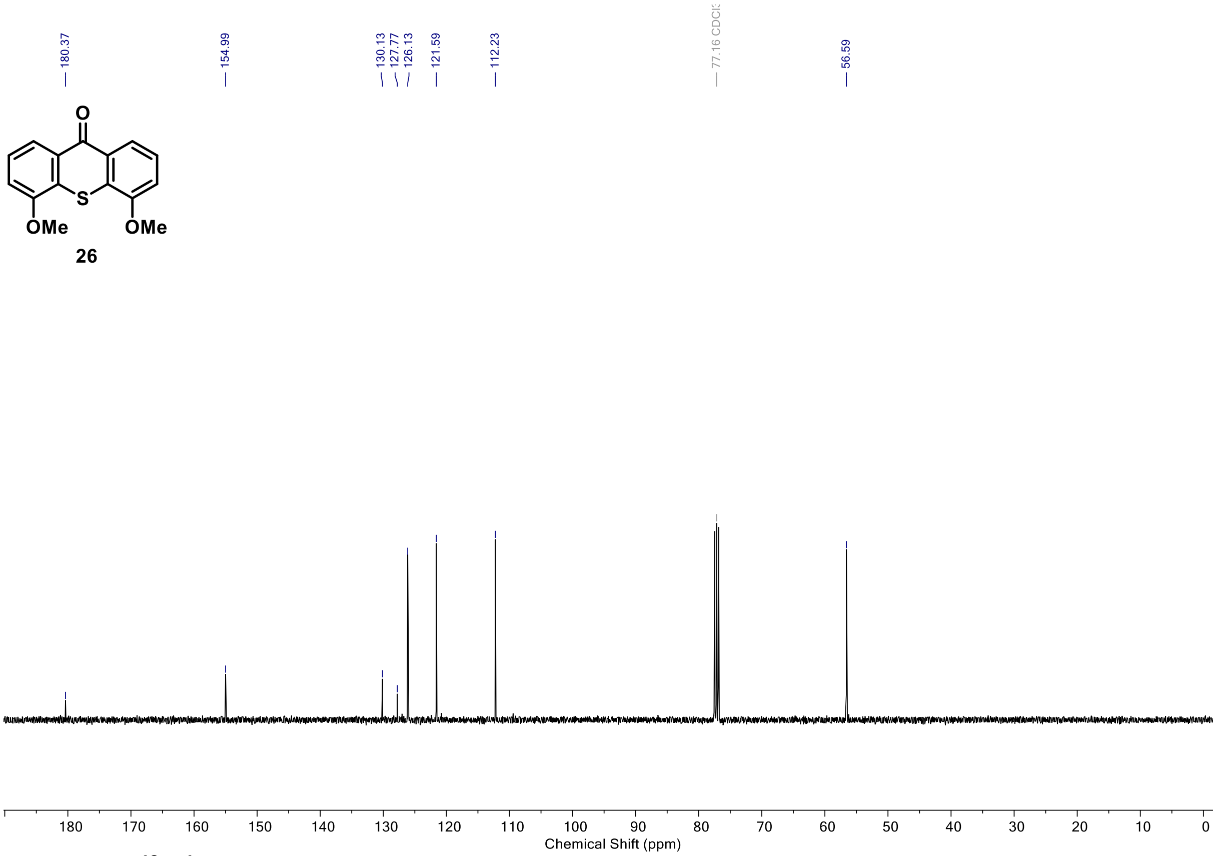

Figure S91: ^{13}**C{**^{1}**H} NMR** spectrum of **26** (151 MHz, $CDCl_3$).

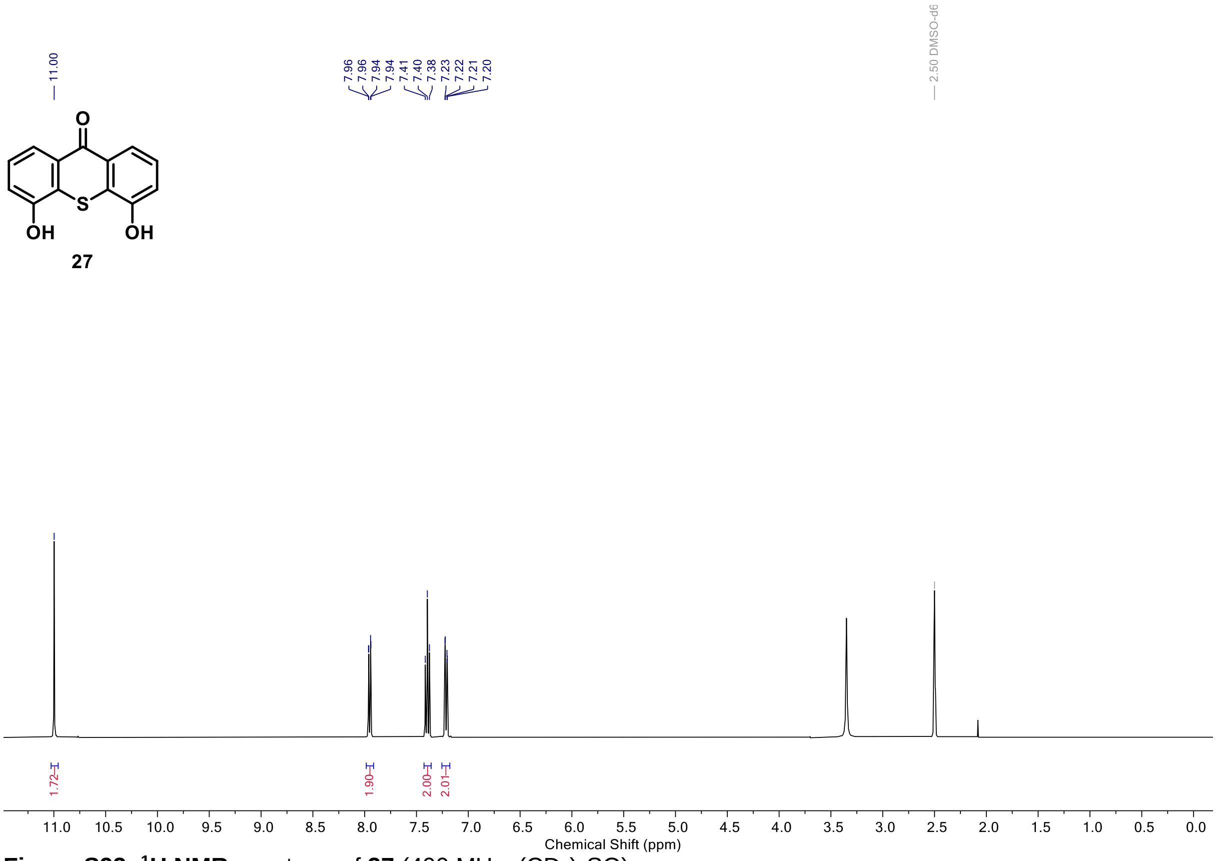

Figure S92: ^{1}**H NMR** spectrum of **27** (400 MHz, $(CD_3)_2SO$).

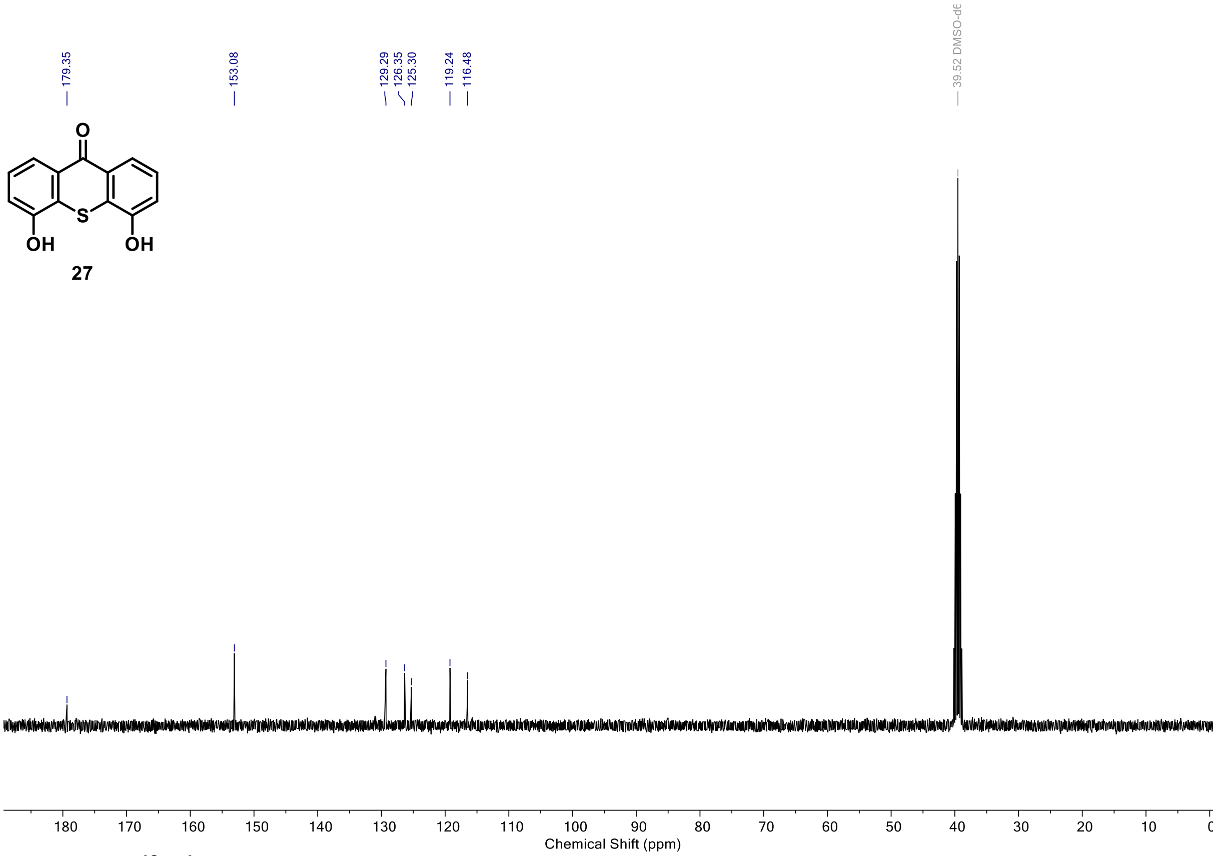

Figure S93: $^{13}C\{^{1}H\}$ **NMR** spectrum of **27** (101 MHz, $(CD_3)_2SO$).

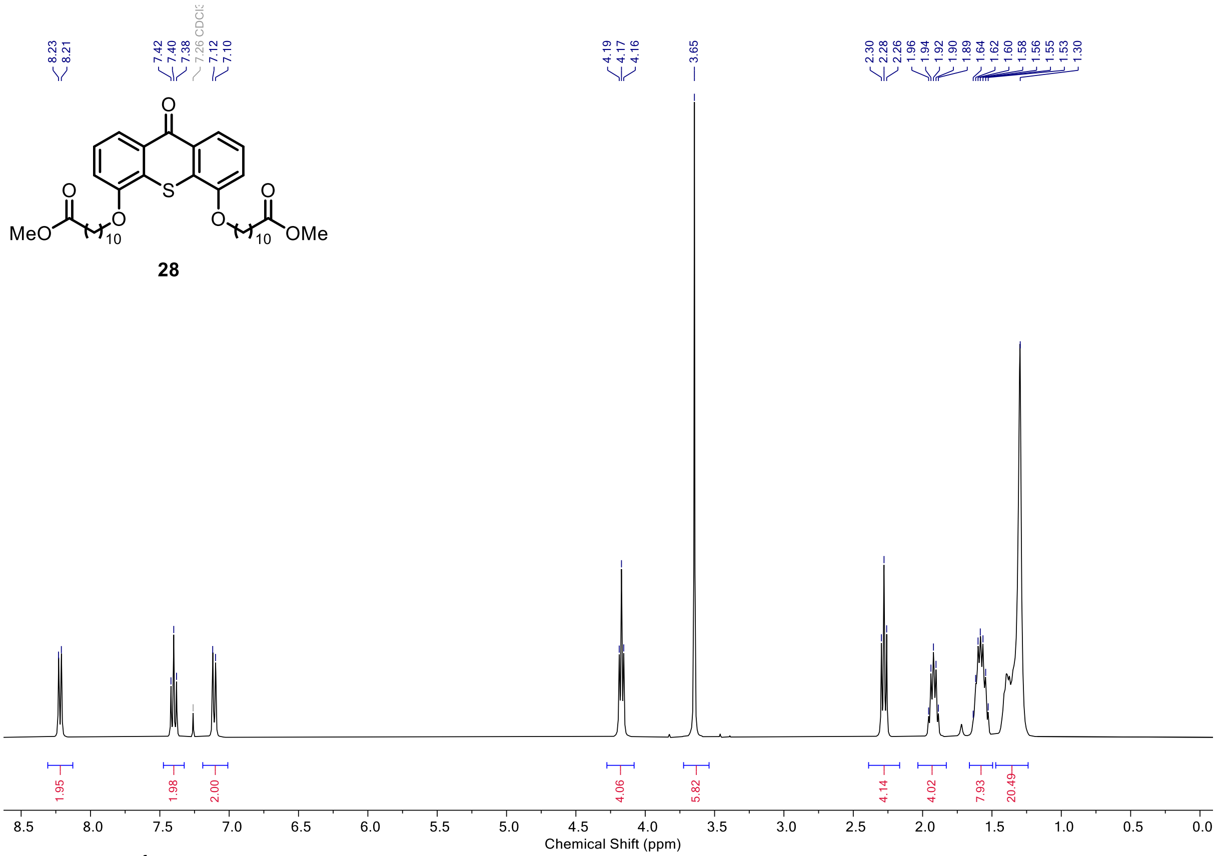

Figure S94: ^{1}**H NMR** spectrum of **28** (400 MHz, $CDCl_3$).

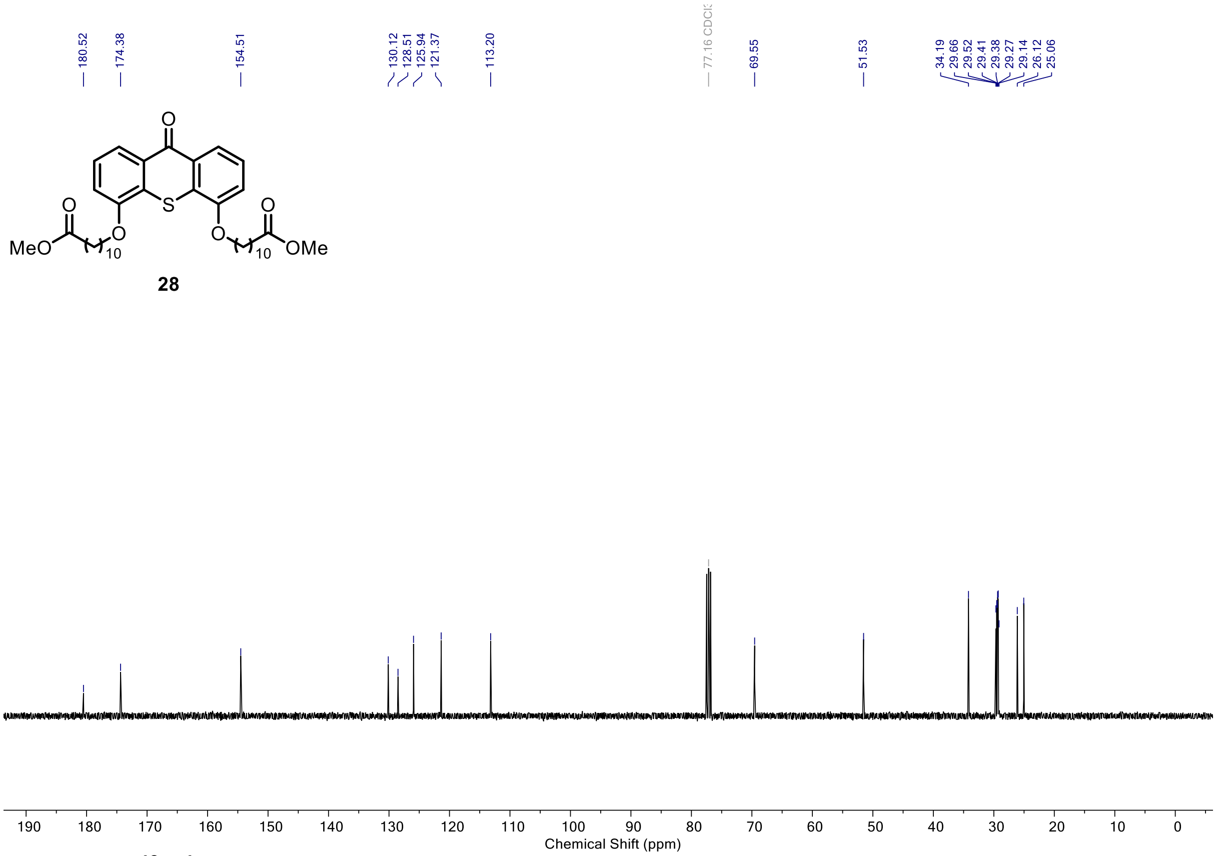

Figure S95: $^{13}C\{^{1}H\}$ NMR spectrum of **28** (101 MHz, $CDCl_3$).
